# Psychosocial and pharmacologic interventions for problematic methamphetamine use: Findings from a scoping review of the literature

**DOI:** 10.1371/journal.pone.0292745

**Published:** 2023-10-11

**Authors:** Mona Hersi, Kim Corace, Candyce Hamel, Leila Esmaeilisaraji, Danielle Rice, Nicole Dryburgh, Becky Skidmore, Gary Garber, Amy Porath, Melanie Willows, Paul MacPherson, Beth Sproule, Jorge Flores-Aranda, Chandlee Dickey, Brian Hutton

**Affiliations:** 1 Ottawa Hospital Research Institute, Ottawa, Canada; 2 Substance Use and Concurrent Disorders Program, The Royal Ottawa Mental Health Centre, Ottawa, Ontario Canada; 3 Department of Psychiatry, Faculty of Medicine, University of Ottawa, Ottawa, Ontario, Canada; 4 Institute of Mental Health Research, University of Ottawa, Ottawa, Ontario, Canada; 5 Department of Psychology, McGill University, Montreal, Quebec, Canada; 6 School of Epidemiology, Public Health and Preventive Medicine, University of Ottawa, Ottawa, ON, Canada; 7 Division of Infectious Diseases, The Ottawa Hospital, Ottawa, ON, Canada; 8 Faculty of Medicine, University of Ottawa, Ottawa, ON, Canada; 9 Knowledge Institute on Child and Youth Mental Health and Addictions, Ottawa, Canada; 10 Department of Family Medicine, Faculty of Medicine, University of Ottawa, Ottawa, ON, Canada; 11 Department of Pharmacy, Centre for Addiction and Mental Health, Toronto, ON, Canada; 12 Leslie Dan Faculty of Pharmacy and Department of Psychiatry, University of Toronto, Toronto, ON, Canada; 13 Université du Quebec à Montréal (UQÀM), Montreal, QC, Canada; 14 Schulich School of Medicine & Dentistry, Western University, London, ON, Canada; Universita degli Studi di Milano-Bicocca, ITALY

## Abstract

**Rationale:**

Methamphetamine use and related harms have risen at alarming rates. While several psychosocial and pharmacologic interventions have been described in the literature, there is uncertainty regarding the best approach for the management of methamphetamine use disorder (MUD) and problematic methamphetamine use (PMU). We conducted a scoping review of recent systematic reviews (SR), clinical practice guidelines (CPG), and primary controlled studies of psychosocial and pharmacologic treatments for MUD/PMU.

**Methods:**

Guided by an a priori protocol, electronic database search updates (e.g., MEDLINE, Embase) were performed in February 2022. Screening was performed following a two-stage process, leveraging artificial intelligence to increase efficiency of title and abstract screening. Studies involving individuals who use methamphetamine, including key subgroups (e.g. those with mental health comorbidities; adolescents/youths; gay, bisexual, and other men who have sex with men) were sought. We examined evidence related to methamphetamine use, relapse, use of other substances, risk behaviors, mental health, harms, and retention. Figures, tables and descriptive synthesis were used to present findings from the identified literature.

**Results:**

We identified 2 SRs, one CPG, and 54 primary studies reported in 69 publications that met our eligibility criteria. Amongst SRs, one concluded that psychostimulants had no effect on methamphetamine abstinence or treatment retention while the other reported no effect of topiramate on cravings. The CPG strongly recommended psychosocial interventions as well as self-help and family support groups for post-acute management of methamphetamine-related disorders. Amongst primary studies, many interventions were assessed by only single studies; contingency management was the therapy most commonly associated with evidence of potential effectiveness, while bupropion and modafinil were analogously the most common pharmacologic interventions. Nearly all interventions showed signs of potential benefit on at least one methamphetamine-related outcome measure.

**Discussion:**

This scoping review provides an overview of available interventions for the treatment of MUD/PMU. As most interventions were reported by a single study, the effectiveness of available interventions remains uncertain. Primary studies with longer durations of treatment and follow-up, larger sample sizes, and of special populations are required for conclusive recommendations of best approaches for the treatment of MUD/PMU.

## Background

Methamphetamine is a highly addictive synthetic psychostimulant, and is one of the most commonly used illicit substances [1]. In Canada, for example, nearly 4% of individuals over the age of 15 reported using methamphetamine at least once in their lifetime with rates higher in males (5.4%) than females (2.2%) [1]. Data from recent years suggest increasing use of harm reduction services for methamphetamines, as well as considerable increases in related hospitalizations, emergency department visits and mortality from overdoses. These data demonstrate the continued emergence of a public health crisis [2, 3]. In the United States (US), 2021 data indicate 0.6% of the population had been diagnosed with methamphetamine use disorder (MUD) in the past year, while 0.9% of individuals aged 12 years or older had used methamphetamine in the past year [4]; increases in risky use of methamphetamines and the diversity of individuals using methamphetamines were reported between 2015–2019, with the number of individuals using methamphetamine 100 or more days per year rising by 66% during that period [5]. In Europe where methamphetamine use has varied historically across regions and been higher in certain countries such as Czhechia and Slovakia, rises in first-time entries to specialised drug treatment programs and monitoring of wastewater data suggest an increase in methamphetamine use has emerged in recent years [6].

Regular methamphetamine use is associated with various harms and symptoms including psychosis, mood swings, insomnia, skin lesions, depression, malnutrition, respiratory illness, gum disease and severe tooth decay, risky behaviours (e.g., criminal behaviours, injection of other illicit substances, sexual practices that increase risk of sexually transmitted infections), and endocarditis [1, 7–18]. Specific patterns of methamphetamine use may indicate problematic use or meet the criteria for a psychiatric diagnosis. MUD, diagnosed by meeting at least two of 11 DSM-5 criteria, is defined as use “leading to clinically significant impairment or distress” [19]. The criteria for problematic methamphetamine use (PMU) is less well defined; in the literature, it has been defined according to thresholds of use (e.g., daily, monthly), consequences of use (e.g., substance use-related emergency department visit), or measures taken to provide treatment (e.g., in-treatment or treatment seeking) [20, 21].

Although various pharmacologic (e.g., bupropion [22, 23], mirtazapine [24, 25], varenicline [26]) and psychosocial (e.g., cognitive behavioural therapy [27], contingency management [28, 29], Matrix model [30]) therapies for MUD/PMU have been explored in the literature, the best approach to treatment remains unclear. Several past reviews have focused upon treatments for stimulant use disorders generally, however focused reviews specific to MUD/PMU may provide helpful information for clinicians. Assessments of the comparative benefits and harms of different treatments for MUD/PMU using evidence synthesis techniques such as network meta-analysis (NMA) may also provide new insights. Additionally, it remains uncertain whether specific treatment considerations for key subpopulations of individuals who use methamphetamines is required; this includes adolescents, those with mental health comorbidities, gay, bisexual, and other men who have sex with men (gbMSM), both cisgender and transgender people, individuals in corrections services, and pregnant women. We conducted a scoping review of recent systematic reviews (SR), clinical practice guidelines (CPG), and primary controlled studies of psychosocial and pharmacologic treatments for MUD/PMU, taking into consideration potential nuances in treatment for specific subpopulations of interest.

### Objectives

This scoping review was designed to answer the following questions:

1. What are the key characteristics and findings/recommendations of recent SRs and CPGs that address psychosocial and pharmacologic treatment of MUD/PMU?
2. What are the key characteristics (e.g., types of patients enrolled, treatments compared, outcomes assessed) of available primary controlled studies evaluating the clinical benefits of psychosocial and pharmacologic interventions for MUD/PMU?
3. What are the clinical benefits and harms of psychosocial and pharmacologic interventions for MUD/PMU?

## Methods

The protocol for this scoping review was published [31] and registered on the Open Science Framework (https://osf.io/9wy8p). This systematic scoping review was guided by established scoping review methodology [32]. We report the review in accordance with the PRISMA Extension Statement for Scoping Reviews [33] (**S1 Text**). Protocol amendments incurred during the review are detailed in **S2 Text** along with corresponding rationale.

### Eligibility criteria

SRs, CPGs, and primary controlled studies were selected for inclusion according to the following criteria, with additional details provided in **S3 Text.**

#### Population

SRs had to include at least one primary study involving participants with MUD/PMU and CPGs had to provide at least one recommendation specific to individuals with MUD/PMU (additional criteria for CPGs are provided below related to study design). Criteria for MUD and PMU were as follows:

Methamphetamine use Disorder: defined by DSM-5 (Box 1 in **S3 Text**), or dependence, abuse, or addiction defined by earlier editions of the DSM, ICD-10, or earlier ICD versions.

Problematic methamphetamine use: participants with no formal diagnosis of MUD, but who are described to be using methamphetamines nearly daily, and/or are seeking treatment for methamphetamine use, and/or are experiencing negative consequences related to methamphetamine use.

Primary studies had to include individuals using methamphetamine. Among those that did not restrict inclusion to those with MUD/PMU, they were included if: (i) ≥67% of the included participants met criteria for MUD/PMU, or (ii) subgroup data were reported for those with MUD/PMU. Additionally, primary studies with individuals who use various drugs (e.g., individuals who use stimulants) were included if subgroup data were available for those with MUD/PMU. Primary studies recruiting those in remission were excluded.

#### Concept

*Interventions*. Relevant interventions include individual and group-based psychosocial interventions (e.g., cognitive behavioural therapy, contingency management, and support programs) and pharmacologic interventions (e.g., dopamine agonists, opioid antagonists) and combinations of psychosocial and pharmacological interventions. Studies of relevant interventions were included if the intent was to address drug use or another condition (e.g., HIV risk behaviours, depression), and where the intent was relapse prevention. Other types of interventions including transcranial magnetic stimulation, exercise, and art-type therapies (e.g., art, music, drama) were excluded. Readers may review details provided in **S3 Text** to acquire additional information regarding the types of psychosocial interventions (e.g., cognitive behavioral therapy, acceptance and commitment therapy, contingency management) that were of interest for this review.

*Comparators*. In the SRs and primary studies, comparators were placebo, no treatment, treatment as usual, another active therapy, or the same intervention but at different frequency/ intensity/ settings.

*Outcomes*. Methamphetamine and other (e.g., alcohol, cannabis, illicit substances) substance use, including measures of abstinence as well as measures of change in consumption over time; mental health outcomes (e.g., depression, quality of life); risk behaviours (e.g., sexual risk behaviors, injection risk practices); harms (e.g., mortality, sexually transmitted infections, withdrawals due to adverse events); and retention/withdrawal.

*Study designs*. SRs had to meet the following criteria: (i) ≥2 databases searched; (ii) selection criteria reported; (iii) quality appraisal of included studies reported; and (iv) a list and synthesis of included studies reported [34, 35]. CPGs had to meet the following criteria: (i) diverse and relevant stakeholders included in guideline development panel; (ii) consensus procedure and methods for guideline development were reported; (iii) funding and the conflicts of interest (financial and non-financial) disclosed for members of the panel; (iv) systematic review methods were used to identify and evaluate evidence; and (v) guideline recommendations were clearly stated with each recommendation accompanied by information of potential benefits and harms and a rating of the level of confidence or strength of the evidence [36, 37]. Evidence syntheses reports not meeting the above criteria, overviews of published SRs, and overviews of published CPGs were excluded.

Included primary studies had to be randomized controlled trials, non-randomized controlled trials, or comparative cohort studies. All other primary study designs (e.g., case-control, mixed-methods, qualitative, cross-sectional, interrupted time series, before-and-after, editorials, letters, commentaries, case series, case reports) were excluded.

#### Context

There were no restrictions on setting of the study or geographic location. Primary studies had to report outcomes at ≥3 months for all outcomes excluding treatment retention. In addition to MUD/PMU populations, additional key subpopulations were included: individuals with mental health co-morbidities; adolescents/youth; gay, bisexual, and other men who have sex with men (gbMSM) (cisgender); gbMSM (transgender); individuals with other substance use problems (i.e., alcohol, cannabis, and illicit substances; excludes tobacco); pregnant people; individuals in correction services; other subgroups (i.e., individuals living with human immunodeficiency virus (HIV), categories of baseline methamphetamine use). For feasibility, only English and French language publications were included. There was no date restriction for publication of the primary studies, but for feasibility and to obtain the most recent information, only SRs and CPGs published in 2015 or later were sought.

### Data sources and search for studies

Search strategies were developed and tested through an iterative process by an experienced medical information specialist (BS) in consultation with the review team. The MEDLINE strategies were peer reviewed by another senior information specialist prior to execution using the PRESS Checklist. Using the OVID platform, we searched Ovid MEDLINE® ALL, Embase Classic+Embase, PsycINFO, Cochrane Database of Systematic Reviews and the Cochrane Central Register of Controlled Trials. Searches were performed on April 15, 2020. Updated database searches were performed in February 2022. Strategies utilized a combination of controlled vocabulary (e.g., “Amphetamine-Related Disorders”, “Methamphetamine”, “Central Nervous System Stimulants”) and keywords (e.g., “MUD”, “cocaine”, “CNS stimulant”). Research design filters were applied as appropriate, and vocabulary and syntax were adjusted across databases. There were no language restrictions on any of the searches but, when possible, conference abstracts, animal-only records and opinion pieces were removed from the results. The search for systematic reviews and clinical practice guidelines was limited to the publication years 2015 to February 2022 (the date of our final search update) to focus these types of data on recent evidence; no date limits were applied to the primary studies search. Specific details regarding the search strategies are provided in **S4 Text**. We also searched for unpublished reports using the Canadian Agency for Drugs and Technologies in Health (CADTH) Grey Matters checklist [38]. We scanned reference lists of relevant systematic reviews, overviews, and primary studies for reports not identified by the electronic database search. In addition, we considered references nominated by clinical experts.

### Study selection process

Search results were imported to EndNote (The EndNote Team; Philadelphia, USA) and duplicate records were excluded. References were imported into DistillerSR^®^ (Evidence Partners Inc; Ottawa, Canada) for study selection. Screening of SRs/CPGs and primary studies was performed separately following a two-stage process. In the first stage, titles and abstracts of SRs/CPGs were evaluated for relevancy using the liberal accelerated method (i.e., one reviewer was required to include a record, and two reviewers to exclude) [39]. In the second stage, full-text screening of potentially relevant SRs/CPGs was performed by two independent reviewers with all disagreements resolved by consensus or third-party arbitration. A calibration exercise was conducted by all reviewers before screening commenced (100 title and abstracts, 25 full-text records). Due to the large yield of the search strategy for primary studies (>16,000 citations), we leveraged artificial intelligence (AI) methods available within DistillerSR^®^ to increase efficiency of title and abstract screening. To pilot test the screening form and to train the AI reviewer, each reviewer independently screened 200 records and all disagreements were resolved. Once the AI reviewer was trained, two independent reviewers screened titles and abstracts until 95% of predicted relevant references were identified; disagreements were resolved regularly throughout the screening process (i.e., every 1–2 days).

All reviewers independently completed a calibration exercise of 25 full-text records, with conflicts resolved through discussion. Full texts of all primary studies were then evaluated by two reviewers, independently. Disagreements were resolved through discussion or third-party arbitration. Co-publications or multiple reports of the same study were treated as companion articles. Corresponding authors were contacted by email when study eligibility was unclear.

Updated searches for SRs/CPGs and primary studies were performed in February 2022. For feasibility, title and abstract as well as full-text screening was performed by a single reviewer. For primary studies, the DistillerSR^®^ AI prioritization tool was used to prioritize citations.

### Data extraction

Data extraction of SRs, CPGs, and primary studies was performed by one reviewer and verified by a second reviewer using DistillerSR^®^. Disagreements were resolved by consensus or third-party arbitration. See **S3 Text** for a list of the information extracted.

For SRs, we collected analyses (i.e., narrative or quantitative syntheses) as reported by review authors. No attempt was made to re-analyze studies (either narratively or quantitatively) in cases where a synthesis was only partially relevant. For example, if a synthesis combined studies of MUD/PMU with those of occasional methamphetamine users, we did not re-analyze the data to improve directness (i.e., by excluding the studies of occasional methamphetamine use). Instead, we limited data collection to analyses that were directly relevant to our eligibility criteria. We did not consult the included primary studies for missing data or to check the accuracy of the data reported within the review.

Where baseline population characteristics were reported by intervention group, we calculated and report combined data across groups [40]. For studies with multiple timepoints of follow-up, we limited data charting to 12-week follow-up, end-of-treatment (if at a point before or after 12 weeks), and the last follow-up (for studies that followed participants beyond the treatment duration).

### Quality appraisal of systematic reviews and practice guidelines

We assessed the quality of SRs using the AMSTAR-2 (A MeaSurement Tool to Assess systematic Reviews) instrument [41]. We assigned an overall rating of quality according to the fulfillment of critical and non-critical items. We used the seven critical items suggested by Shea et al. [41]; all other items were treated as non-critical. Reviews with one or more critical flaws (and with or without non-critical weaknesses) were considered to be of low or critically low quality, respectively. Reviews with no critical flaws and more than one non-critical weakness were rated as moderate quality. High quality reviews had no critical flaws and up to one non-critical weakness.

We used the AGREE-2 (Appraisal of Guidelines for REsearch & Evaluation) instrument to assess the quality of CPGs [42]. We rated each of the 23 items and a total score was calculated for each of the six domains. Two overall assessments of the guideline were also performed. First, we provided an overall assessment of the quality of the guideline on a 7-point scale. Secondly, we assessed whether we would recommend the guideline.

AMSTAR-2 and AGREE-2 assessments were completed by one reviewer and verified by a second. All disagreements were resolved by consensus. We did not assess the quality of primary studies, as is common in scoping reviews.

### Charting of data

Characteristics and findings from SRs and CPGs have been summarized in tables and figures; key findings have also been summarized descriptively. Characteristics of primary research studies have been summarized in tables.

To synthesize primary study results, where possible, we followed Cochrane guidance and performed vote counting based on the direction of the estimate of effect with no consideration of statistical significance [43]. Using this approach, for each effect estimate, other reported measure (e.g., rate difference) or narrative statement, we categorized each finding as ‘favours intervention’, ‘favours comparison’, ‘no difference between groups’, or ‘unclear’ (e.g., where only p-values reported). For harms or other negative outcomes (e.g., methamphetamine-positive urine samples, dropout), “favors intervention” was interpreted as fewer or a decrease of events with the intervention relative to the comparison group.

For outcomes (e.g., percentage of methamphetamine-negative urine screens, depressive symptoms, risk behaviours) reported cross-sectionally (i.e., at a specific follow-up timepoint such as week 16) rather than as a change from baseline, we limited data charting to those controlling for baseline differences, irrespective of the magnitude or statistical significance of those baseline differences. Where unadjusted and adjusted effect estimates were reported, we prioritized adjusted estimates; where multiple adjusted estimates were reported, we relied on the estimate adjusted for baseline differences. If results were only reported as rates per arm, we did not calculate relative effect estimates; decisions regarding direction of effects relied upon the reported rates. Where the difference in rates between groups was less than or equal to 0.5%, we interpreted this as ‘no difference’ between groups. Where outcome data were inadequately reported in text (e.g., limited to a statement regarding statistical significance), for the purpose of determining the direction of effects, we considered data reported in tables and figures (e.g., GEE fitted-lines) unless there was a lack of confidence in the interpretation of the data reported therein. We relied on authors’ reporting of outcomes at face-value; for example, where it appeared that the authors’ intent was to report outcome data cross-sectionally rather than as a change from baseline, we did not use data reported in tables or figures to determine the degree of change from baseline. However, where authors intended to report the outcome as a change from baseline, but results were inadequately reported in text, we did rely on data (i.e., baseline and follow-up) reported in tables for the purpose of determining the direction of effects.

### Synthesis

For a high-level approach to the synthesis, where feasible, we combined similar outcome measures. For example, we combined studies reporting the longest period of uninterrupted methamphetamine abstinence reported as mean days, weeks, and number of negative urine samples.

Due to the variation in intervention and comparisons in the included primary studies, we performed vote counting and report only high-level narrative summaries for each population or subgroup of interest. Narratives are grouped by population or subgroup, and then by intervention comparison. We changed the effect direction description of ‘positive health impact’ or ‘negative health impact’ to ‘favours intervention’ or ‘favours comparison’. Effect direction plots are reported by outcome category and separately for each population or subgroup of interest. Data related to the gbMSM subpopulation as well as individuals with mental health comorbidities are presented within the main text of this review, while data regarding additional subpopulations (e.g., those with light/heavy baseline methamphetamine use) are presented in data supplements in consideration of brevity.

### Feasibility assessment for network meta-analysis

An additional objective of the review was to assess the feasibility of performing network meta-analyses (NMA) [44] that would allow for syntheses of trial data to inform simultaneous comparisons amongst pharmacologic and psychosocial interventions based upon all available evidence. Such investigations are informative toward ensuring both the presence of sufficient data as well as avoidance of threats to validity that can arise through systematic differences amongst studies in the network. We followed guidance from Cope et al. [45] and inspected the studies with regard to network connectivity, the similarity of interventions (e.g. comparability of implementation and nature of grouped psychosocial interventions, and dose and schedule of pharmacologic interventions), the distribution of characteristics of study populations (e.g. age, percent male, lifetime duration of methamphetamine use, number of days of methamphetamine use in the past 14–30 days, history of self-help or treatment program), and the potential to address heterogeneity using techniques such as meta-regression and subgroup analysis. We prepared evidence tables of study characteristics and bar plots of study-level characteristics to examine the extent of methodologic and clinical differences between studies; the availability of outcomes across studies was also assessed, and collectively all information was discussed by the study team. Findings from this assessment are described.

## Results

### Search results

**Fig 1:**
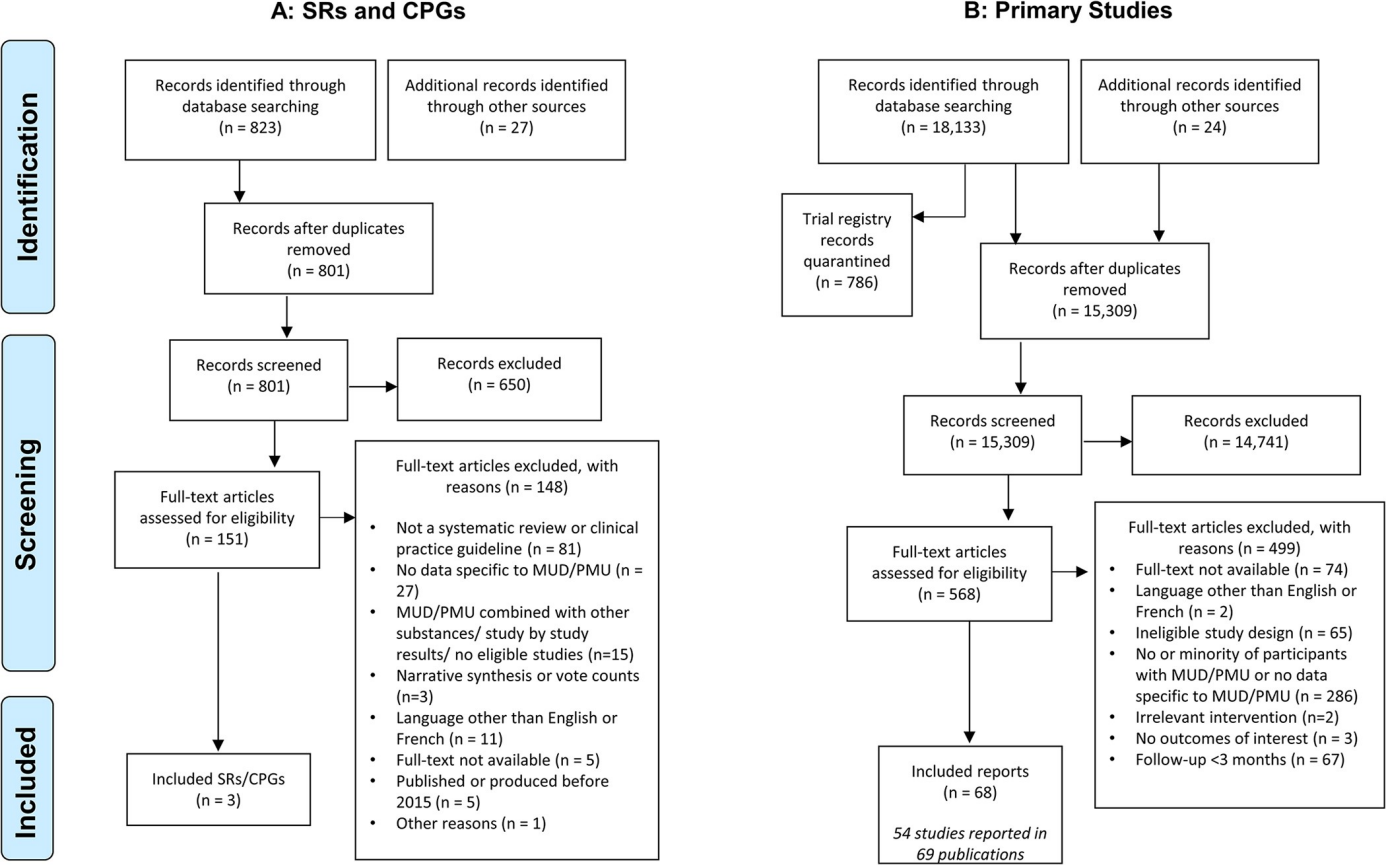
PRISMA diagrams.

In the search for SRs and CPGs, we identified 823 records through database searching and an additional 27 from the grey literature. One CPG and two SRs were included (**Fig 1, Panel A**). A list of excluded records at full-text, sorted by reason for exclusion, is provided in **S5 Text**.

In the search for primary studies, we identified 18,133 records through database searching and an additional 24 from grey literature searching (**Fig 1, Panel B**). After trial registries and duplicates were removed, 15,309 records remained. Using DistillerSR’s^®^ prioritized screening capabilities 95% (518/546) of predicted relevant references were identified after screening 4334 records. The highest rank score (i.e., likelihood of study inclusion) amongst remaining records was 0.0768. At this point, the remaining records that were not fully reviewed (i.e., reviewed by 2 reviewers) were excluded using the AI reviewer tool (n = 9,832). To perform a quality control check, a human reviewer screened 600 records that were excluded by the AI reviewer tool. No new relevant records were identified. The highest rank score of remaining studies was 0.0739. We ran the audit tool to verify if any records may have been incorrectly excluded (i.e., identified by an inclusion score of 0.85 or greater). Fifty records were identified by the audit, and re-reviewed, of which nine were deemed potentially relevant and included by a human reviewer. Additional screening and auditing were performed two more times, at which point no more records where included. All other records were excluded, and title/abstract screening was complete.

Sixty-nine publications [22–30, 46–104] reporting fifty-four studies [22–30, 46–83, 98–104] met our eligibility criteria and were included. One companion article [105] was a long-term follow-up of a study [46], while other companion articles primarily presented data on additional outcomes, subgroups or additional analyses of outcomes.

### Objective 1: What are the key characteristics and recommendations/findings of recent CPGs and SRs that address psychosocial and pharmacologic treatment of MUD/PMU?

#### Clinical practice guidelines

One clinical practice guideline, commissioned by the German Federal Ministry of Health (BMG) and German Medical Association (BÄK), was included [106, 107] (Table 1). The guideline provided 63 recommendations covering treatment planning, acute therapy in emergency settings, withdrawal management, post-acute therapy, relapse prevention, and treatment of co-occurring mental health disorders and treatment for specific subpopulations (i.e., gbMSM, pregnant persons). Using the AGREE-II instrument, the overall quality rating was 4 (7-point scale) (see **S6 Text** for more details).

**Table 1:** Clinical practice guideline and systematic review details.

Guidelines		
Details	Target profession; Target population	Assessing levels of evidence or grading of recommendations
**Braunwarth 2016** [106, 107] **Title**: S3 practice guideline methamphetamine-related disorders **Authoring Group**: German Federal Ministry of Health (BMG) and German Medical Association (BÄK) **Objective**: To develop evidence-based recommendations for the treatment of methamphetamine-related disorders. **Search date**: 2000-June 2015 **# of included studies**: 103 SRs/primary studies, 9 clinical practice guidelines **Country**: Germany **Funding**: Not reported	Physicians, psychotherapists, professionals working in outpatient and inpatient addiction services, caregivers in aftercare and rehabilitation settings, self-help organizations, partners to the medical profession (e.g., other occupations involved in healthcare, payer organizations); Individuals with methamphetamine-related disorders	Levels of evidence: Oxford Centre for Evidence-Based Medicine 2011 (CEBM); Grading of recommendations: Using a three-category ranking system (i.e., strong recommendation, recommendation, open)
**Systematic Reviews**		
**Details**	**Population, Substance use criteria, Intervention(s) & Comparator(s)**	**Outcome(s)**
**Bhatt 2016** [108] **Search details:** database inception to August 2016 **# of included studies**: 14 randomized controlled trials **Funding**: Non-industry funding	**Population**: Adolescents and adults ≥14 years of age, Amphetamine or methamphetamine abuse, dependence, or use disorder, **Interventions**: Psychostimulants^1^ **Comparator**: Placebo	• Abstinence from amphetamines and methamphetamines • Retention in treatment • Serious adverse events
**Nourredine 2021** [109] **Search details:** run on May 2020 **# of included studies**: 62 randomized controlled trials, non-randomized studies, meta-analyses **Funding**: No funding	**Population:** Unrestricted, Addictive and eating disorders including substance use disorders and dependence **Intervention:** Topiramate **Comparator:** Not specified as part of eligibility criteria. Single arm as well as placebo-controlled studies included.	• Cumulative abstinence • Abstinence rates • Number of drinking days • Time to first relapse • Number of heavy drinking days • Drinks per drinking day • Binge eating frequency • Gamma-glutamyl transferase • Quality of life • Obsession • Craving

^1^ Including “psychoanaleptics with mild stimulant effects (bupropion, modafinil) as well as classical stimulants (dextroamphetamine, methylphenidate, and dexmethylphenidate”[108].

Briefly, recommendations for post-acute treatment that were associated with a strong level of recommendation, which included the following:

1. regarding care delivery structures in post-acute management, there was strong support that the use of self-help and family support groups for post-acute management as well as offering motivation-based psychotherapeutic interventions be a component of care;
2. there was strong recommendation against use of sertraline, PROMETA^TM^, and dopamine analog treatments with narcotic-classified substances outside of registered clinical trial settings; and
3. there were strong recommendations that disease specific evidence-based treatment and psychoeducation for co-occurring mental health and substance use conditions be provided to individuals with MUD/PMU.

#### Systematic reviews

The two included SRs were published in 2016 and 2021, with the most recent database search dates ranging from November 2015 to May 2020 (**Table 1**). Results from these SRs [108, 109] were extracted, and are briefly described. Review authors from both SRs concluded that there was no evidence of an effect of psychostimulants on terminal abstinence (i.e., maintaining abstinence from methamphetamine at the end of the trial) and treatment retention in the context of amphetamine and methamphetamine use disorder [108], and no difference between topiramate and placebo with regard to changes in methamphetamine craving [109]. Using the AMSTAR-2 instrument (assessments provided in S6 Text), one review was rated to be of low quality [108] due to the presence of one critical flaw (i.e., did not provide a list of studies evaluated at full-text that were excluded) and two non-critical weaknesses (i.e., did not describe for inclusion of study designs, did not perform data extraction in duplicate);the other was rated to be of critically low quality [109] due to weaknesses in three of the four domains identified as critical (i.e., no explicit statement of methods being established prior to the conduct of the review, did not provide a list of studies evaluated at full-text that were excluded, did not account for risk of bias in individual studies when interpreting/discussing the results of the review), and four non-critical weaknesses (i.e., did not describe for inclusion of study designs, did not perform data extraction in duplicate, did not report the source of funding for the primary studies included in the review, did not provide an explanation for or discuss heterogeneity observed in the results of the review).

### Objective 2: What are the key characteristics (e.g., types of patients enrolled, treatments compared, outcomes assessed) of available primary controlled studies evaluating the clinical benefits of psychosocial and pharmacologic treatment of MUD/PMU?

A summary of study and population characteristics is presented in **Table 2**, with individual characteristics summarized per study in tabular form in **S7 Text** and summarized graphically in **S8 Text** (including plots of mean age, % males, % Caucasian, lifetime duration of methamphetamine use, and number of days of methamphetamine use in the last 14–30 days).

**Table 2:** Characteristics of included primary studies.

	Descriptor	n (%) [N = 54]
*Study characteristics*		
Study design	RCT	51 (94.4%)
	Comparative cohort	3 (5.6%)
Country of conduct	United States	33 (61.1%)
	Iran	11 (20.4%)
	Australia	6 (11.1%)
	China; Germany; South Africa; Thailand	1 (1.9%) (each)
Funding	Non-industry	46 (85.2%)^1^
	Industry	1 (1.9%)
	No funding	1 (1.9%)
	Not reported	6 (11.1%)
*Population characteristics*		
Population	Adults only	52 (96.3%)
	Adolescents and adults	2 (3.7%)
Age	Mean age range (n = 53 studies)	23.7 to 43.3 years
	Mean	35.1 years
	Median	35.9 year
Sex/gender	Male and female	36 (66.7%)
	Male born/identifying only	16 (29.6%)
	Female only	2 (3.7%)
Substance use	MUD, dependence and/or abuse	48 (88.9%)
	[Diagnostic criteria: DSM; ICD; Not reported]	[41; 1; 6]
	Individuals who use methamphetamines; not restricted to MUD	4 (7.4%)
	Stimulant use disorder; stimulant and/or alcohol use disorder (reporting subgroup data)	2 (3.7%)
Treatment status	Individuals seeking treatment	26 (48.1%)
	Non-daily methamphetamine use	2 (3.7%)
	Methadone treatment	2 (3.7%)
	Moderate to severe MUD; Not seeking treatment; Inpatient rehabilitation centres; inpatients who received detox services	1 (1.9%) (each)
	Not reported	20 (37.0%)
Subpopulations	“Unrestricted” (no subpopulation)	42 (77.8%)
	Transgender and/or cisgender gbMSM	9 (16.7%)
	Patients with mental health comorbidities	3 (5.6%)
*Intervention characteristics*		
Intervention categories	Pharmacologics^2^ *[Behavioural therapy co-intervention]*	30 (55.6%) *[29]*
	Psychosocial therapies^3^	22 (40.7%)
	Combination pharmacologic/psychosocial	2 (3.7%)

^1^ 7 (15.2%) interventions supplied by pharmaceutical companies

^2^ 20 different pharmacological interventions (e.g., bupropion, modafinil, mirtazapine) (see **S10 Text**)

^3^ 14 different psychosocial therapies (e.g., contingency management, cognitive behavioural therapy, Matrix model), with some studies offering more than one type of therapy (see **S10 Text**)

**Abbreviations**: DSM = Diagnostic and Statistical Manual of Mental Disorders; gbMSM = gay, bisexual and other men who have sex with men; ICD = International Classification of Diseases; MUD = Methamphetamine use disorder

#### Study characteristics

Study sample size and duration ranged from 18 to 978 participants and from 12 to 156 weeks, respectively. Setting varied across studies and included outpatient substance use treatment facilities, research clinic sites, primary health care centers, and inpatient rehabilitation centers. Among studies reporting race (n = 34), the majority of patients were Caucasian in all but four studies [23, 69, 73, 102]. Measures of duration of methamphetamine use varied across studies and included age of onset, years since first-time use, years of continuous or regular use, lifetime duration of use, number of days of methamphetamine use in the past 14 to 30 days, and frequency of use reported categorically. Data for commonly reported duration measures along with baseline use severity and craving are presented in **S9 Text**.

#### Outcomes reported

**Fig 2:**
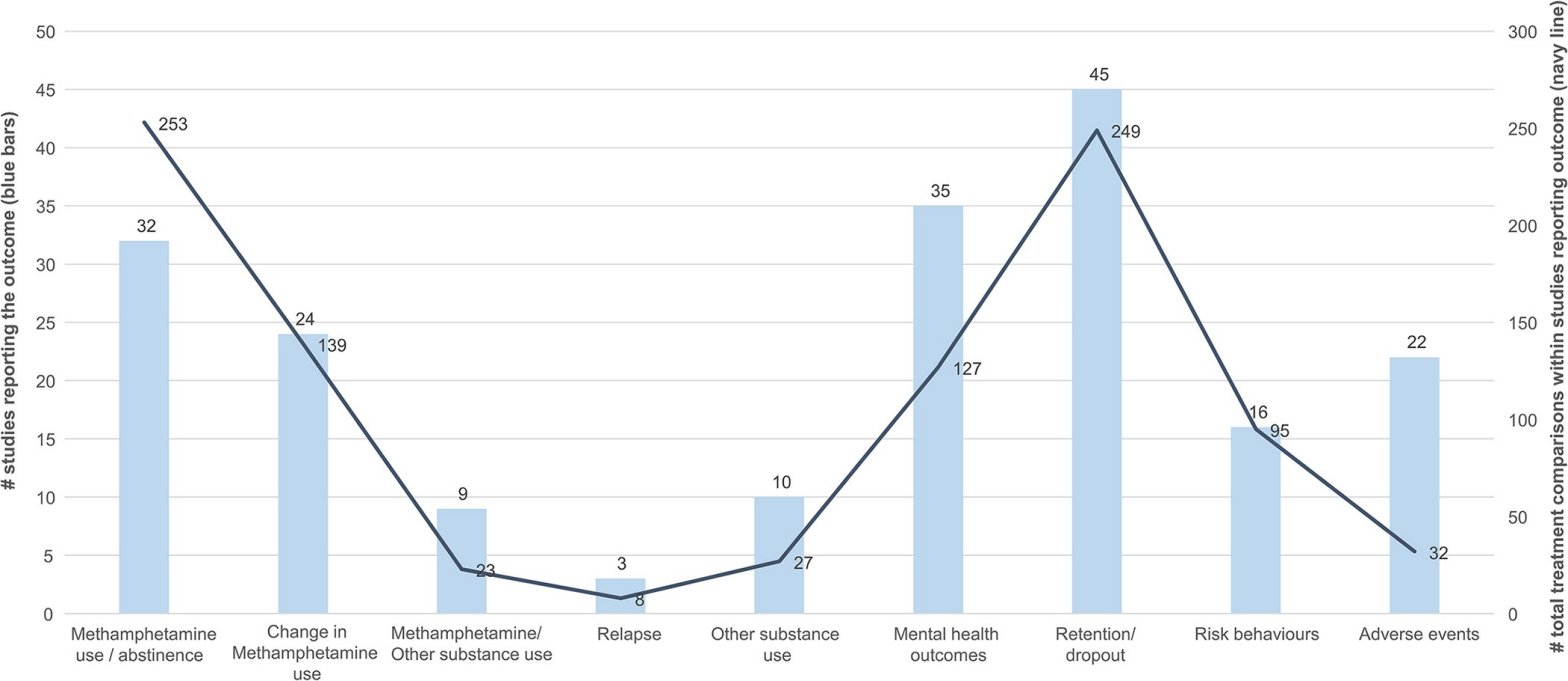
Summary of outcomes reported in primary studies. Methamphetamine use, abstinence: 195 of 253 estimates included in synthesis, Change in methamphetamine use: 90 of 139 estimates included in synthesis.

We determined the direction of treatment effects for 952 estimates reported across 69 publications (54 studies). As indicated in **Fig 2**, the most commonly reported outcomes were retention/dropout (45 studies, 249 estimates), methamphetamine use and abstinence (32 studies, 253 estimates), and change in methamphetamine use (34 studies, 139 estimates). Several outcomes were sparsely reported, including other substance use (i.e., alcohol, cannabis, and illicit substances excluding methamphetamine), relapse, and adverse events. The outcomes reported by each study are shown in **S11 Text.**

Methamphetamine outcomes were reported in various ways across studies. For example, after combining similar measures, *change in methamphetamine use* was reported in terms of 19 unique outcome measures across studies, while *methamphetamine use/abstinence* was reported in terms of 18 different measures. As noted in the methods and within our summary of protocol amendments for this review (**S2 Text**), for reasons of feasibility, syntheses (i.e., vote counting or narrative summaries) of methamphetamine outcomes were limited to the most commonly reported measures across studies (counting for less common measures was captured in summary as ‘all additional outcomes’, with their directions of effect captured). As such, 195 of the 253 estimates on methamphetamine use and abstinence and 90 of the 139 estimates of change in methamphetamine use were included. All reported substance use outcome domains, and measures have been outlined in **S11 Text**.

### Objective 3: What are the clinical benefits and harms of psychosocial and pharmacologic interventions for MUD/PMU?

#### Intervention details

Several behavioral and pharmacological interventions were evaluated, and most intervention comparisons were examined by a single study. Behavioral interventions included cognitive behavioral therapy (CBT), various contingency management (CM) programs, counselling, motivational interviewing, community-based programs, group therapy, and text-based and web-based interventions. Some studies compared and/or combined a behavioral intervention with a pharmacological intervention. Most pharmacological interventions (i.e., aripiprazole, baclofen, buprenorphine, bupropion, dexamphetamine, gabapentin, ibudilast, imipramine HCl, methylphenidate, modafinil, N-acetylcysteine, PROMETA^TM^ protocol (consisting of flumazenil, gabapentin and hydroxyzine), riluzole, topiramate, valproate, varenicline) were compared to placebo, while some studies compared two different pharmacological therapies, and some compared different dosages of the same pharmacological therapy. Pharmacotherapies used in the studies specific to the gbMSM population and participants with mental health comorbidities often differed from those in the studies with the unrestricted sample of methamphetamine users. Studies which included participants with mental health comorbidities often only evaluated pharmacological therapies. Most studies that reported on harms evaluated pharmacological therapies.

Given the considerable quantity, intricacy, heterogeneity, and sparsity of the available data, brief descriptions of the intervention and comparator and statements of the overall findings are provided in **Table 3** for unrestricted samples of methamphetamine users, **Table 4** for gbMSM populations, and **Table 5** for individuals with mental health comorbidities. Within these tables, high level descriptions pertaining to the relative benefits of treatments within different studies are provided, and we refer readers seeking further details of the collected data to **Figs A1-A37 in S1 Data** to view all extracted information including study design, time point of follow-up, and method of outcome ascertainment for each of the 952 estimates in the effect direction plots. For a more comprehensive descriptive summary, we also refer readers to **S12 Text** which provides a descriptive account of findings from primary studies. As noted earlier, data related to the gbMSM subpopulation as well as individuals with mental health comorbidities are presented within the tables of this review, while data regarding additional subpopulations (e.g., those with light/heavy baseline methamphetamine use) are presented in **S12 Text** and **S1 Data**, respectively, in consideration of brevity.

**Table 3:** Descriptive synthesis of overall results for unrestricted sample of methamphetamine users.

Intervention and Comparators	Overall results statement
**Contingency Management (CM) (n = 5 studies)**	
1. Continuous; Intermittent predictable; Intermittent unpredictable CM; No TRT [52] 2. 1-, 2-, 4-month CM + SPT; SPT alone [29] 3. 5 CM schedules compared to each other [74] 4. Escalating with reset CM; Escalating without reset CM [28] CM + treatment as usual; Treatment as usual [72]	**•** CM performed better than no treatment or standard treatment alone [29, 52, 72] **•** More CM sessions or longer periods of session outperformed fewer sessions and shorter periods [29] **•** Escalating with reset CM outperformed escalating without reset CM for methamphetamine use abstinence [28]
**Cognitive behavioural therapy (CBT) (n = 2 studies)**	
1. 1-, 2-, 3- and 4-session CBT; Self-help booklet [27, 87, 88] 2. Acceptance and Commitment Therapy vs CBT [81]	More CBT sessions over fewer sessions, and CBT over self-help booklet with the exception of self-help booklet outperforming 1-session of CBT [27, 87, 88] **•** Variation in outcome results between ACT and CBT with no difference in harms [81]
**Other behavioural interventions (n = 7 studies; 13 articles)**	
1. C-B RR; C-B detox groups; Individual counselling during C-B RR; No individual counselling during C-B RR; Outpatient counselling; No treatment [53, 91, 92] 2. Matrix model; Treatment as usual [70, 94, 95] 3. Inpatient residential rehabilitation (FAST model); Outpatient matrix model treatment [69] 4. CGT + 10 hours of group therapy focusing on stimulant use; CGT [46, 105] 5. Intensive MI; Standard MI [30, 86] 6. Blended imaging desensitisation + MI; Treatment as usual [102] 7. Educational intervention; Treatment as usual [60]	**•** Matrix model outperformed both treatment as usual [70, 94, 95] and inpatient residential rehabilitation [69] with the exception of retention/dropout, where inpatient residential rehabilitation was favored [69] **•** Other interventions (i.e., outpatient counseling, community-based residential rehabilitation and community-based detox [53, 91, 92], blended imaging desensitisation [102], educational intervention [60]) outperformed treatment as usual/no treatment for most outcomes (e.g., meth use/abstinence, change in meth use, mental health), but less often for retention/dropouts [53, 91, 92, 102] **•** Other comparisons provided results where the more effective treatment depended on the outcome evaluated (e.g., intensive versus standard motivational interviewing [30, 86]) or unclear results due to poor reporting [46, 105]
**Behavioural + Pharmacotherapy interventions (n = 7 studies; 13 articles)**	
1. Sertraline; Sertraline + CM; Placebo + CM; Placebo [78, 97] 2. Modafinil; CBT [100] 3. Matrix model; Methylphenidate; Matrix model + Methylphenidate; No treatment [104]	**•** Among studies that evaluated pharmacological and behavioural interventions combined, theextent of benefits varied by outcome measure [78, 97, 104] **•** When comparing a behavioral intervention to a pharmacotherapy intervention, CBT tended to outperform Modafinil [100]
**Pharmacotherapy interventions (n = 23 studies; 25 articles)**	
1. Aripiprazole; Placebo [54] 2. Baclofen; Gabapentin; Placebo [61] 3. Bupropion; Placebo [22, 23, 50, 80, 84, 90] 4. Ibudilast; Placebo [63] 5. Methylphendiate; Placebo [66, 103] 6. Modafinil 200 mg; Modafinil 400 mg; Placebo [49, 62, 76, 96] 7. PROMETA protocol; Placebo [65] 8. Riluzole; Placebo [58] 9. Valproate; Placebo [64] 10. Buprenorphine; Placebo [75] 11. N-acetyl cysteine; Placebo [101] 12. Dexamphetamine; Placebo [67] 13. Topiramate; Placebo [57, 89] 14. 150 mg Imipramine HCI; 10 mg Imipramine HCI [59] 15. Varenicline; Placebo [26]	**•** Several outcomes could not be assessed due to limitations in reporting **•** For many methamphetamine use outcomes, mental health outcomes and risk behaviors, many pharmacological therapies (i.e., Baclofen [61], Riluzole [58], Valproate [64], Buprenorphine [75], Dexamphetamine [67], Varenicline [26]), were favored to placebo, with the exception of Gabapentin [61] and Ibudilast [63]. **•** Aripiprazole [54], Bupropion [22, 23, 50, 80, 84, 90], Methylphenidate [66, 103], Modafinil (200 mg and 400 mg) [49, 62, 76, 96], PROMETA protocol [65], and N-acetyl cysteine [101] provided mixed results (i.e., related to degree of benefit) when compared to placebo depending on the outcome **•** Unclear reporting of results in the Topiramate study did not allow for determination of which was favored [57, 89] **•** Approximately half of the pharmacological therapy studies reported on harms often favoring placebo or no difference between, with the exception of Modafinil 200 mg [76, 96], PROMETA protocol [65], and Dexamphetamine [67] **•** Retention/dropout varied across studies **•** In studies that compared two pharmacological therapies, Baclofen was favored to Gabapentin (except for harms) [61], Modafinil 400 mg was favored to 200 mg although several outcomes were unclear [49], and results for 150 mg versus 10 mg of Imipramine HCI were mixed [59]

Available treatment comparisons are presented alongside high-level synopses of clinical findings across outcomes and studies. Readers interested in study-specific and outcome-specific results are referred to S1 Data and S12 Text for additional information.

ACT: Acceptance and Commitment Therapy; C-B RR: Community-based residential rehabilitation; CBT: Cognitive behavioural therapy; CGT: Conventional group therapy; CM: Contingency management; MI: Motivational interviewing; SPT: standard psychosocial therapy

**Table 4:** Descriptive synthesis of overall results for gay and bisexual and other men who have sex with men.

Intervention and Comparators	Overall results statement
**Other behavioural interventions (n = 5 studies; 7 articles)**	
1. Voucher-based reinforcement CM; gb men-specific CBT; CBT + Voucher-based reinforcement CM; CBT [77, 85, 93] 2. Gay-specific CBT; Gay social support therapy [79] 3. Combined (counselling + Mobile app Ecological momentary assessments + Web-based dashboard); Mobile+ (Mobile app Ecological momentary assessments + Web-based dashboard); Matched historical control [71] 4. Combined (Interactive text message with peer health educator + Automated gay-specific text message + Weekly self-monitoring text-based assessment); Automated+ (Automated gay-specific text message + Weekly self-monitoring text-based assessment); Weekly self-monitoring text-based assessment [73] 5. Behavioural activation + Sexual risk reduction counselling; Sexual risk reduction counselling [68]	**•** CM (voucher based), gay and bisexual men-specific CBT alone or with CM were all favored to CBT alone [77, 85, 93], and interventions involving CM (voucher based) were favored to gb men-specific CBT [77, 85, 93] **•** Gay specific CBT was favored to Gay social support therapy for both meth use/abstinence and change in meth use outcomes [79]. When comparing multi-faceted interventions (e.g., incorporating self-monitoring), results were mixed depending on the outcome [71, 73] **•** Retention/dropout was favored for the Behavioural Activation + Sexual Risk Reduction counselling group over Sexual risk reduction counselling alone [68] **•** No studies reported on harms
**Pharmacotherapy interventions (n = 4 studies)**	
1. Mirtazapine; Placebo [24, 25] 2. Naltrexone; Placebo [55] 3. Bupropion; Placebo [56]	**•** Outcomes for Mirtazapine [24, 25], Naltrexone [55], and Bupropion [56] were mixed depending on the outcome, with several results unclear due to limitations in reporting. **•** For example, Mirtazapine was favoured to placebo for meth use/abstinence and risk behaviour, however placebo was favoured to Mirtazapine for mental health outcome, with no difference in retention/dropout [24, 25].

Available treatment comparisons are presented alongside high-level synopses of clinical findings across outcomes and studies. Readers interested in study-specific and outcome-specific results are referred to S1 Data and S12 Text for additional information.

CBT: Cognitive behavioural therapy; CM: Contingency management

**Table 5:** Descriptive synthesis of overall results for participants with mental health comorbidities*.

Intervention and Comparators	Overall results statement
**Behavioural interventions (n = 2 studies; 4 articles)**	
1. 1-session, 2-session, 3 to 4-session CBT; Self help booklet [27, 87, 88] 2. Self-compassion training; Control [99]	**•** Self-help booklet was favored to single session CBT, however 2- and 3 to 4-session CBT were favored to self-help booklet. When comparing number of session, 2-session CBT was favored when compared to the others [27, 87, 88]. **•** Self-compassion training vs control (not further described) reported only on retention/dropout and there was no difference [99] **•** No studies reported on harms
**Pharmacotherapy interventions (n = 4 studies; 5 articles)**	
1. Paliperidone vs Placebo [83] 2. Bupropion; Placebo [22, 50, 90] 3. Citicoline; Placebo [51]	**•** Paliperidone [83] and Citicoline [51] was mostly favored to placebo, and Bupropion [22, 50, 90] had unclear reporting of results.

Available treatment comparisons are presented alongside high-level synopses of clinical findings across outcomes and studies. Readers interested in study-specific and outcome-specific results are referred to S1 Data and S12 Text for additional information.

Mental health comorbidities varied between studies and included diagnoses such depression, attention deficit hyperactivity disorder and bipolar depression. A detailed account by study is provided within the effect direction plots provided in S1 Data.

CBT: Cognitive behavioural therapy

### Feasibility assessment: Network meta-analysis

Detailed information reviewed during the feasibility assessment for NMA are provided in **S8 Text** (bar plots of population baseline characteristics), **S9 Text** (measures of population baseline methamphetamine use and severity), **S10 Text** (intervention characteristics) and **S11 Text** (availability of outcomes by study), respectively. Review of these information identified variability between study populations in terms of several effect modifiers (e.g., age, % male, ethnicity, measures of methamphetamine use) as well as a lack of reporting of certain characteristics, making it difficult for our clinical team members to judge the degree of homogeneity of populations from study to study. The sparse nature of the evidence base was noted, along with variability in outcome definitions and timing of measurement of the outcomes of interest. Variability in the implementation of behavioral interventions were noted, representing a challenge to the formulation of homogeneous treatment nodes for NMA. In consideration of all these factors, our team felt that currently available evidence related to pharmacologic and behavioral interventions is not amenable to the development of robust NMAs.

## Discussion

Given the increasing rate of methamphetamine use in many countries and the uncertainty regarding best approaches to treatment, we conducted a scoping review to identify, characterize, and summarize the findings of available studies of pharmacologic and psychosocial treatments for MUD/PMU. We also sought to address current knowledge gaps with regard to potential nuances in treatment for special populations, seeking out information for priority groups that included adolescents, gbMSM, pregnant people, individuals with mental health comorbidities, other substance use problems, or individuals in correction services. Notably, the high degree of between-study variability in terms of the outcome measures used and approaches to intervention implementation complicates the ability to compare findings between studies and to draw robust conclusions regarding the benefits and harms of different intervention strategies.

While there are indications of potential benefits for certain treatments from both psychosocial (e.g., contingency management, Matrix model, CBT, ACT, blended imaginal desensitization plus motivational interviewing) and pharmacologic (baclofen, bupropion, gabapentin, dexamphetamine, imipramine, methylphenidate, modafinil, valproate, buprenorphine, riluzole, topiramate, N-acetylcysteine) approaches and certain combinations thereof, research for many approaches was sparse; many intervention strategies were assessed in only small, single studies. Evidence was also noted to be heterogeneous in terms of the outcome measures used (including 37 different approaches used to measure methamphetamine usage) as well as approaches to intervention implementation. Data for the subgroups of interest were also sparse. Some evidence was identified for interventions in the gbMSM population, however, data for other a priori subgroups including those with mental health comorbidities, pregnant individuals and incarcerated persons were largely unavailable. Many trials enrolled broad populations yet did not consistently report information pertaining to key subgroups of individuals, and there remains a high need for additional data to understand how well different interventions work in these populations. Such challenges in the evidence limit the conclusions that can be drawn from this scoping review and serve as a limiting factor toward formal quantitative analyses of the evidence such as meta-analysis.

### Limitations of the scoping review

Consistent with scoping review methodology, we took a high-level approach to synthesizing the evidence. This approach often necessitated consolidating similar outcome measures despite differences in the way the measures were defined or measured. For example, regarding substance use outcomes, studies varied with respect to the number of substance-negative drug screens required for satisfying the treatment success criteria. Although studies were consistent in requiring all submitted samples to be substance-negative, submission of at least one urine drug screen was sufficient in some studies while others required a minimum of two samples per week.

Our approach to synthesizing the evidence, involving extraction of the direction of effects and vote counting, did not account for the magnitude of effects, statistical significance, nor differences in sample sizes across studies [43]. Extracting the direction of effect for each estimate was conducted at face-value and did not consider whether the magnitudes of between-group differences were appreciable or clinically meaningful. For example, a difference of 0.02 points on the Addiction Severity Index may not indicate an appreciable difference in alcohol or drug use between groups.

For pragmatic purposes, we limited syntheses (i.e., narrative summaries) to the most commonly reported methamphetamine outcome measures across studies. While this protocol adjustment occurred after full-text screening, outcome measures were selected objectively (i.e., based on frequency of reporting). With regard to CPGs, we required articles presenting recommendations to meet a very thorough set of methods in their design, and we acknowledge the availability of other CPGs related to PMU/MUD which may also be of clinical interest to readers [110]. Lastly, as we limited inclusion to studies reported in English and French, it is unknown whether the studies excluded due to language would have otherwise met the eligibility criteria.

### Future research

Limited evidence could be collected from the identified systematic reviews due to inadequate reporting of findings as well as the decision to assess treatment effects across different types of stimulant use disorders; future systematic reviews should consider performing separate analyses for participants with MUD/PMU (as opposed to grouping of all stimulant use disorders). Primary studies with longer durations of treatment and follow-up, larger sample sizes, and of special populations of individuals who use methamphetamines are required. The considerable variation in outcome measures across studies should be addressed by the development of a standardized set of outcomes for MUD/PMU treatment studies, in accordance to the Core Outcome Measures in Effectiveness Trials (COMET) methodology [111]. Future studies should adhere to the core outcome measures to ensure consistency in reporting across studies, thereby allowing for quantitative syntheses. Endpoints that capture changes in methamphetamine use, mental health status, risk behaviors, retention in treatment and other measures were most readily available in our review and all warrant consideration as core outcomes, while the advantages and limitations of different ways to measure these endpoints warrant discussion amongst experts in the development process.

## Conclusions

This scoping review provides readers with an overview of available interventions as well as an awareness of those which show potential benefits when treating individuals with MUD/PMU. Unfortunately, as many interventions were evaluated in a single study only, the effectiveness of available interventions remains uncertain. Primary studies with longer durations of treatment and follow-up, larger sample sizes, and of special populations of those who use methamphetamine are required for conclusive recommendations of best approaches for the treatment of MUD/PMU. Improved consistency in outcome selection as well as improvements in the description of intervention implementation should be goals for the conduct of future trials. There remains an important need for additional research into effective therapies for problematic methamphetamine use and methamphetamine use disorder. This review can provide people with lived experience, care providers, policy and decision makers the latest evidence regarding available interventions for MUD/PMU, and thus can be informative toward clinical practice, education/training, public health and awareness.

## Supporting information

S1 Text PRISMA ScR checklist. (DOCX)

S2 Text Protocol amendments. (DOCX)

S3 Text Additional eligibility criteria and methods details. (DOCX)

S4 Text Search strategies. (DOCX)

S5 Text List of excluded studies. (DOCX)

S6 Text Quality assessment of systematic reviews and meta-analyses. (DOCX)

S7 Text Primary study characteristics. (DOCX)

S8 Text Bar plots of baseline population characteristics. (DOCX)

S9 Text Baseline methamphetamine use and severity. (DOCX)

S10 Text Intervention characteristics. (DOCX)

S11 Text Outcomes per study and substance use outcomes/measures. (DOCX)

S12 Text Narrative summary of primary studies. (DOCX)

S1 Data Effect direction plots. (XLSX)
