# Supplementary material for: Psychosocial and pharmacologic interventions for problematic methamphetamine use: Findings from a scoping review of the literature
Source: PLoS One. 2023 Oct 11;18(10):e0292745. doi: 10.1371/journal.pone.0292745 (PMC10566716; doi:10.1371/journal.pone.0292745)
Supplement: S2 Text — (DOCX) [file pone.0292745.s002.docx]

**S2 Text. Protocol amendments**

*Systematic reviews*

A majority of reviews analyzed MUD/PMU studies with those of methamphetamine-using participants not meeting MUD/PMU criteria (e.g., occasional users) or with studies of other substance users (e.g., amphetamines and other stimulants). Studies that did provide MUD- or PMU-specific data often reported study-by-study descriptions of findings rather than performing a synthesis across studies. This study-by-study data was redundant given our efforts to include and map primary studies as part of this scoping review. As such, we limited data mapping to syntheses reported within SRs that were specific to MUD/PMU and which included at least two studies.

A few SRs met the criteria for including analyses (i.e., MUD/PMU-specific and >1 study), however, the narrative syntheses were poorly reported limiting our ability to accurately map the data. In particular, when authors reported a narrative synthesis or vote count, the number of studies contributing to the analysis for a given comparison and outcome (i.e., denominator) was often unclear. For example, where a single narrative synthesis (or vote count) included multiple outcomes, it was unclear whether all of the mentioned studies provided data for each of the outcomes or alternatively, if only one study reported on each of the outcomes (e.g., ‘none of the 3 studies reported significant findings for methamphetamine use, adherence, and craving’). Regarding the outcome data, the direction of effect was also often inadequately reported (i.e., vote counting based on statistical significance). Cross-checking narrative summaries with data reported in tables and figures was often also limited by inadequate reporting in this supplementary information. To avoid errors and misrepresentation of data, we decided to exclude narrative and vote counting syntheses.

Reviews that met the eligibility criteria of the scoping review but did not report usable syntheses were also excluded.

*Primary studies*

- To increase prioritization accuracy, instead of liberal accelerated screening, two independent reviewers screened titles and abstracts until 95% of predicted relevant references were identified. Also, once a recall of 95% was achieved, all remaining studies were excluded by the AI reviewer; for feasibility, only a subset were screened by a human reviewer.
- We considered participants receiving treatment at baseline as an indicator of PMU. Also pertaining to substance use criteria, we decided to include studies of MUD even if the use of diagnostic criteria (i.e., DSM/ICD) was not explicitly stated (n=6).
- Regarding data extraction, for feasibility, we did not extract quantitative results data (e.g., event rates, effect estimates) from included primary studies. Therefore, we did not synthesize data per outcome as median values and treatment effect ranges for each comparison as stated in the protocol.
- Due to the substantive number of outcomes and outcome measures reported within studies, we reduced the number of relevant outcomes. Prioritization of outcomes was performed by members of the authorship team with clinical expertise. The final list of included outcomes included: methamphetamine and other substance use (e.g., abstinence, change/reduction in use, relapse), mental health (e.g., depression, quality of life), risk behaviours (e.g., sexual risk behaviours, injection risk practices), harms (e.g., adverse events, sexually transmitted infections mortality, study withdrawal due to adverse events), retention/withdrawal (i.e., study retention/dropout, treatment retention/dropout).

Methamphetamine outcomes were reported in various ways across studies. After combining similar measures, change in methamphetamine use was reported as 19 unique outcome measures across studies while methamphetamine use/abstinence was reported as 18 different measures. This corresponded to a total of 392 estimates of methamphetamine use, abstinence, or change in use. For feasibility, we limited syntheses (i.e., vote counting or narrative summaries) to the most commonly reported methamphetamine outcome measures across studies. This resulted in the inclusion of six measures of methamphetamine use/abstinence and four measures of change in methamphetamine use for a total of 285 estimates included in the synthesis. Less frequently reported outcome measures (corresponding to 107 estimates) were excluded from the synthesis but have been consolidated and presented in effect direction plots.
