# Supplementary material for: Psychosocial and pharmacologic interventions for problematic methamphetamine use: Findings from a scoping review of the literature
Source: PLoS One. 2023 Oct 11;18(10):e0292745. doi: 10.1371/journal.pone.0292745 (PMC10566716; doi:10.1371/journal.pone.0292745)
Supplement: S5 Text — (DOCX) [file pone.0292745.s005.docx]

# S5 Text. Exclusion at full-text by reason

## Systematic reviews and Clinical Practice Guidelines

### Full-text not available

Gupta, S., Jhanjee, S., Dhawan, A. Effectiveness of Interventions Based on Yogic Breathing Practices (IB-YBP) on Substance Use Disorders-A Systematic Review of the Randomized Control Trials and Quasi-Experimental Trials. 2021. 56 (11): 1624-1641.

Ivanov, Iliyan, Pearson, Andrew, Kaplan, Gabriel, and Newcorn, Jeffrey. Attention deficit hyperactivity disorder and comorbid substance abuse. Clinical aspects of psychopharmacology in childhood and adolescence., 2nd ed. 2017: 61-80.

Mathew, S.. Amphetamine Withdrawal: Management. Joanna Briggs Institute 2020. JBI ID: JBI436

Smout, MS, Krasnikow, S, and Longo, M. QUICKFIX: Identify and intervene in psychostimulant use in primary health care. Drug and Alcohol Services South Australia 2015.

Knapp, W. P., Soares, B. G., Farrell, M. F., and Silva de, Lima M. Psychosocial interventions for cocaine and psychostimulant amphetamines related disorders. Cochrane Database of Systematic Reviews 2015. 2015 (4): CD003023.

### Language other than English/French

Arranz, B., Garriga, M., Bernardo, M., Gonzalez-Pinto, A., Arrojo, M., Torrens, M., Tirado-Munoz, J., Fonseca, F., Saiz, P. A., Florez, G., Goikolea, J. M., Zorrilla, I., Cunill, R., Castells, X., Becona, E., Lopez, A., San, L. Clinical Practice Guideline on Pharmacological and Psychological management of adult patients with Schizophrenia Spectrum Disorders and a comorbid substance use. 2021. 0 (0): 1504.

Crunelle, C. L., Van Den Brink, W., Schellekens, A., Van De Glind, G., and Matthys, F. International consensus for the screening, diagnosis and treatment of adult patients with substance use disorder and ADHD. Tijdschrift voor Psychiatrie 2019. 61 (7): 477-487.

Cunill, R., Castells, X., Gonzalez-Pinto, A., Arrojo, M., Bernardo, M., Saiz, P. A., Florez, G., Torrens, M., Tirado-Munoz, J., Fonseca, F., Arranz, B., Garriga, M., Goikolea, J. M., Zorrilla, I., Becona, E., Lopez, A., San, L.. Clinical practice guideline on pharmacological and psychological management of adult patients with attention deficit and hyperactivity disorder and comorbid substance use. 2021. 0 (0): 1569.

Gonzalez-Pinto, A., Goikolea, J. M., Zorrilla, I., Bernardo, M., Arrojo, M., Cunill, R., Castell, X., Becona, E., Lopez, A., Torrens, M., Tirado-Munoz, J., Fonseca, F., Arranz, B., Garriga, M., Saiz, P. A., Florez, G., San, L.. Clinical practice guideline on pharmacological and psychological management of adult patients with bipolar disorder and comorbid substance use. 2021. 0 (0): 1528.

Li, F., Yin, L., Yang, H., and Chen, L.. Efficacy of different drugs for patients with methamphetamine-induced psychotic disorders: A network meta-analysis. Chinese Journal of Evidence-Based Medicine 2017. 17 (7): 835-842.

Luderer, Mathias, Kiefer, Falk, Reif, Andreas, and Moggi, Franz. [ADHD in adult patients with substance use disorders]. Der Nervenarzt 2019. 90 (9): 926-931.

Mende, A.. Crystal meth: New S3 guideline presented. Pharmazeutische Zeitung 2016. 161 (49).

Neumann, S., Franke, A. G., and Soyka, M.. Methamphetamine addiction in Germany: A selective review about epidemiology, phenomenology and therapy. Nervenheilkunde 2016. 35 (11): 742-747.

Ozgen, H., Spijkerman, R., Noack, M., Holtmann, M., Schellekens, A. S. A., van de Glind, G., Banaschewski, T., Barta, C., Begeman, A., Casas, M., Crunelle, C. L., Daigre Blanco, C., Dalsgaard, S., Demetrovics, Z., den Boer, J., Dom, G., Eapen, V., Faraone, S. V., Franck, J., Gonzalez, R. A., Grau-Lopez, L., Groenman, A. P., Hemphala, M., Icick, R., Johnson, B., Kaess, M., Kapitany-Foveny, M., Kasinathan, J. G., Kaye, S. S., Kiefer, F., Konstenius, M., Levin, F. R., Luderer, M., Martinotti, G., Matthys, F. I. A., Meszaros, G., Moggi, F., Munasur-Naidoo, A. P., Post, M., Rabinovitz, S., Ramos-Quiroga, J. A., Sala, R., Shafi, A., Slobodin, O., Staal, W. G., Thomasius, R., Truter, I., van Kernebeek, M. W., Velez-Pastrana, M. C., Vollstadt-Klein, S., Vorspan, F., Young, J. T., Yule, A., van den Brink, W., Hendriks, V.. [International Consensus Statement for the Screening, Diagnosis, and Treatment of Adolescents with Concurrent Attention-Deficit/Hyperactivity Disorder and Substance Use Disorder]. 2021. 50 (1): 54-67.

Torrens, M., Tirado-Munoz, J., Fonseca, F., Farre, M., Gonzalez-Pinto, A., Arrojo, M., Bernardo, M., Arranz, B., Garriga, M., Saiz, P. A., Florez, G., Goikolea, J. M., Zorrilla, I., Cunill, R., Castells, X., Becona, E., Lopez, A., San, L.. Clinical practice guideline on pharmacological and psychological management of adult patients with depression and a comorbid substance use disorder. 2021. 0 (0): 1559.

Wodarz, N., Christ, M., Fleischmann, H., Looser, W., Schoett, K., Vilsmeier, F., Scheidler, A., Schaefer, C., Bothe, L., Muhlig, S., and Gouzoulis-Mayfrank, E.. Selected Recommendations from the S3-Guidelines on Pharmacological Management of Acute Methamphetamine-Related Mental Disorders Toxicity. Sucht 2019. 65 (4): 273-285.

### Produced or published before 2015

Substance misuse in adolescents: alcohol, cannabis, and other drugs.. Best Practise Journal 2012. 42: 28.

Centre for Reviews and Dissemination. Citicoline in addictive disorders: a review of the literature (Provisional abstract). Database of Abstracts of Reviews of Effects 2015. (2).

Centre for Reviews and Dissemination. Antipsychotics for cocaine or psychostimulant dependence: systematic review and meta-analysis of randomized, placebo-controlled trials (Provisional abstract). Database of Abstracts of Reviews of Effects 2015. (2).

Centre for Reviews and Dissemination. A systematic review of behavioral and treatment outcome studies among HIV-infected men who have sex with men who abuse crystal methamphetamine (Provisional abstract). Database of Abstracts of Reviews of Effects 2015. (2).

Centre for Reviews and Dissemination. Antipsychotic drug treatment of schizophrenic patients with substance abuse disorders (Provisional abstract). Database of Abstracts of Reviews of Effects 2015. (2).

### Failed to meet a priori criteria for systematic reviews or clinical practice guidelines

Medi-Cal Contingency Management Pilot Program Policy Design. 2022: 1-34.

Methamphetamine Treatment Guidelines: Practice Guidelines for Health Professionals. [Internet]. https://www.turningpoint.org.au/sites/default/files/2019-05/Turning-Point-Methamphetamine-Treatment-Guidelines.pdf 2018. (2nd. ed.).

Peer support interventions for substance use disorder: clinical effectiveness, cost-effectiveness, and guidelines. Canadian Agency for Drugs and Technologies in Health 2020.

Addressing methamphetamine use in primary care. https://bpac.org.nz/2018/meth.aspx 2018.

Management of acute presentations related to methamphetamine use: Clinical guidelines for adults and adolescents. https://cracksintheice.org.au/pdf/download/acute-presentations-related-to-methamphetamine-use-clinical-guideline-for-adults-and-adolesce.pdf 2017.

Psychostimulant early intervention flow chart: combined. Insight Clinical Support Services 2016.

Clinical guidelines for assessment and management of psychostimulant users. NSW Health 2015.

Managing Methamphetamine Use: Evidence Review Report. https://cmnalberta.com/wp-content/uploads/2019/09/Methamphetamine-Evidence-Review_08_28_2019.pdf 2019.

Ahmed, Saeed, Bachu, Ramya, Kotapati, Padma, Adnan, Mahwish, Ahmed, Rizwan, Farooq, Umer, Saeed, Hina, Khan, Ali Mahmood, Zubair, Aarij, Qamar, Iqra, and Begum, Gulshan. Use of Gabapentin in the Treatment of Substance Use and Psychiatric Disorders: A Systematic Review. Frontiers in psychiatry 2019. 10: 228.

Al Hazzani, Saad A.. Use of Evidence-Based Decision-Making in Comprehensive Dental Treatment of a Patient with Meth Mouth-A Case Report. The journal of evidence-based dental practice 2017. 17 (2): 92-98.

Alam-mehrjerdi, Zahra, Mokri, Azarakhsh, and Dolan, Kate. Methamphetamine use and treatment in Iran: A systematic review from the most populated Persian Gulf country. Asian journal of psychiatry 2015. 16: 17-25.

Alice Springs, NT Centre for Remote Health. CARPA standard treatment manual. Alice Springs, NT: Centre for Remote Health 2020.

Baandrup, Lone, Ostrup Rasmussen, Jesper, Klokker, Louise, Austin, Stephen, Bjornshave, Thomas, Fuglsang Bliksted, Vibeke, Fink-Jensen, Anders, Hedegaard Fohlmann, Allan, Peter Hansen, Jens, Kristine Nielsen, Malene, Sandsten, Karl Erik, Schultz, Vilhelm, Voss-Knude, Susanne, and Nordentoft, Merete. Treatment of adult patients with schizophrenia and complex mental health needs - A national clinical guideline. Nordic journal of psychiatry 2016. 70 (3): 231-240.

Ballester, J., Valentine, G., and Sofuoglu, M.. Pharmacological treatments for methamphetamine addiction: current status and future directions. Expert Review of Clinical Pharmacology 2017. 10 (3): 305-314.

Barnes, T. R. E., Drake, R., Paton, C., Cooper, S. J., Deakin, B., Ferrier, I. N., Gregory, C. J., Haddad, P. M., Howes, O. D., Jones, I., Joyce, E. M., Lewis, S., Lingford-Hughes, A., MacCabe, J. H., Owens, D. C., Patel, M. X., Sinclair, J. M. A., Stone, J. M., Talbot, P. S., Upthegrove, R., Wieck, A., and Yung, A. R.. Evidence-based guidelines for the pharmacological treatment of schizophrenia: Updated recommendations from the British Association for Psychopharmacology. Journal of Psychopharmacology 2020. 34 (1): 3-78.

BCCSU. Risk Mitigation in the Context of Dual Public Health Emergencies. 2022.

Berlin, Rachel K., Butler, Paul M., and Perloff, Michael D.. Gabapentin Therapy in Psychiatric Disorders: A Systematic Review. The primary care companion for CNS disorders 2015. 17 (5).

Brown, H. D. and DeFulio, A.. Contingency management for the treatment of methamphetamine use disorder: A systematic review. Drug and alcohol dependence 9-21-2020. 216: 108307.

Carpentier, Pieter Jan and Levin, Frances R.. Pharmacological Treatment of ADHD in Addicted Patients: What Does the Literature Tell Us?. Harvard review of psychiatry 2017. 25 (2): 50-64.

Chen, Xi Jing, Wang, Dong Mei, Zhou, Li Dan, Winkler, Markus, Pauli, Paul, Sui, Nan, and Li, Yong Hui. Mindfulness-based relapse prevention combined with virtual reality cue exposure for methamphetamine use disorder: Study protocol for a randomized controlled trial. Contemporary clinical trials 2018. 70: 99-105.

Chiang, Mathew, Lombardi, Domenico, Du, Jiang, Makrum, Ursula, Sitthichai, Rangsun, Harrington, Amy, Shukair, Nawras, Zhao, Min, and Fan, Xiaoduo. Methamphetamine-associated psychosis: Clinical presentation, biological basis, and treatment options. Human psychopharmacology 2019. 34 (5): e2710.

Coles, A. S., Kozak, K., and George, T. P.. A review of brain stimulation methods to treat substance use disorders. American Journal on Addictions 2018. 27 (2): 71-91.

Coles, Alexandria S., Sasiadek, Julia, and George, Tony P.. Pharmacotherapies for co-occurring substance use and bipolar disorders: A systematic review. Bipolar disorders 2019. 21 (7): 595-610.

Connors, N. J., Alsakha, A., Larocque, A., Hoffman, R. S., Landry, T., and Gosselin, S.. Antipsychotics for the treatment of sympathomimetic toxicity: A systematic review. American Journal of Emergency Medicine 2019. 37 (10): 1880-1890.

Crunelle, Cleo L., van den Brink, Wim, Moggi, Franz, Konstenius, Maija, Franck, Johan, Levin, Frances R., van de Glind, Geurt, Demetrovics, Zsolt, Coetzee, Corne, Luderer, Mathias, Schellekens, Arnt, ICASA consensus group, and Matthys, Frieda. International Consensus Statement on Screening, Diagnosis and Treatment of Substance Use Disorder Patients with Comorbid Attention Deficit/Hyperactivity Disorder. European addiction research 2018. 24 (1): 43-51.

Davis, D. R., Kurti, A. N., Skelly, J. M., Redner, R., White, T. J., and Higgins, S. T.. A review of the literature on contingency management in the treatment of substance use disorders, 2009-2014. Preventive medicine 2016. 92: 36-46.

De, Giorgi R., D'Alo, G. L., and De, Crescenzo F.. Psychosocial interventions in stimulant use disorders: A focus on women. Current Opinion in Psychiatry 2017. 30 (4): 275-282.

Deepmala, Slattery, J., Kumar, N., Delhey, L., Berk, M., Dean, O., Spielholz, C., and Frye, R.. Clinical trials of N-acetylcysteine in psychiatry and neurology: A systematic review. Neuroscience and biobehavioral reviews 2015. 55: 294-321.

Department of Veterans Affairs.[U.S.] Department of Defence. VA/DoD Clinical Practice Guidelines: Clinical Practice Guideline for the Management of Substance Use Disorders.[Internet]. https://www.healthquality.va.gov/guidelines/MH/sud/ 2015.

DiClemente, Carlo C., Corno, Catherine M., Graydon, Meagan M., Wiprovnick, Alicia E., and Knoblach, Daniel J.. Motivational interviewing, enhancement, and brief interventions over the last decade: A review of reviews of efficacy and effectiveness. Psychology of addictive behaviors: journal of the Society of Psychologists in Addictive Behaviors 2017. 31 (8): 862-887.

Duailibi, Michel Silvio, Cordeiro, Quirino, Brietzke, Elisa, Ribeiro, Marcelo, LaRowe, Steve, Berk, Michael, and Trevizol, Alisson Paulino. N-acetylcysteine in the treatment of craving in substance use disorders: Systematic review and meta-analysis. The American journal on addictions 2017. 26 (7): 660-666.

Enokibara, M., Trevizol, A., Shiozawa, P., and Cordeiro, Q.. Establishing an Effective TMS Protocol for Craving in Substance Addiction: Is It Possible?. American Journal on Addictions 2016. 25 (1): 28-30.

Forster, S. E., DePhilippis, D., and Forman, S. D.. "I's" on the prize: A systematic review of individual differences in Contingency Management treatment response. Journal of substance abuse treatment 2019. 100: 64-83.

Gaur, N., Gautam, M., Singh, S., Venkatesh, Raju, V, and Sarkar, S.. Clinical practice guidelines on assessment and management of substance abuse disorder in children and adolescents. Indian Journal of Psychiatry 2019. 61 (8 Supplement 2): S333-S349.

Ha, Z. Y., Mathew, S., and Yeong, K. Y.. Butyrylcholinesterase: A multifaceted pharmacological target and tool. Current Protein and Peptide Science 2020. 21 (1): 99-109.

Hauer, Larissa, Sellner, Johann, Brigo, Francesco, Trinka, Eugen, Sebastianelli, Luca, Saltuari, Leopold, Versace, Viviana, Holler, Yvonne, and Nardone, Raffaele. Effects of Repetitive Transcranial Magnetic Stimulation over Prefrontal Cortex on Attention in Psychiatric Disorders: A Systematic Review. Journal of clinical medicine 2019. 8 (4).

Hellem, Tracy L., Lundberg, Kelly J., and Renshaw, Perry F.. A review of treatment options for co-occurring methamphetamine use disorders and depression. Journal of addictions nursing 2015. 26 (1): 14-E1.

Hone-Blanchet, Antoine, Ciraulo, Domenic A., Pascual-Leone, Alvaro, and Fecteau, Shirley. Noninvasive brain stimulation to suppress craving in substance use disorders: Review of human evidence and methodological considerations for future work. Neuroscience and biobehavioral reviews 2015. 59: 184-200.

Jones, Jennifer L., Mateus, Camilo F., Malcolm, Robert J., Brady, Kathleen T., and Back, Sudie E.. Efficacy of Ketamine in the Treatment of Substance Use Disorders: A Systematic Review. Frontiers in psychiatry 2018. 9: 277.

Kuppili, P. P., Manohar, H., and Menon, V.. Current status of vaccines in psychiatry-A narrative review. Asian journal of psychiatry 2018. 31: 112-120.

Lappan, Sara N., Brown, Andrew W., and Hendricks, Peter S.. Dropout rates of in-person psychosocial substance use disorder treatments: a systematic review and meta-analysis. Addiction (Abingdon, England) 2020. 115 (2): 201-217.

Lefaucheur, J.-P., Aleman, A., Baeken, C., Benninger, D. H., Brunelin, J., Di, Lazzaro, V, Filipovic, S. R., Grefkes, C., Hasan, A., Hummel, F. C., Jaaskelainen, S. K., Langguth, B., Leocani, L., Londero, A., Nardone, R., Nguyen, J.-P., Nyffeler, T., Oliveira-Maia, A. J., Oliviero, A., Padberg, F., Palm, U., Paulus, W., Poulet, E., Quartarone, A., Rachid, F., Rektorova, I., Rossi, S., Sahlsten, H., Schecklmann, M., Szekely, D., and Ziemann, U.. Evidence-based guidelines on the therapeutic use of repetitive transcranial magnetic stimulation (rTMS): An update (2014-2018). Clinical Neurophysiology 2020. 131 (2): 474-528.

Li, KX and Loshak, H. Treatment for methamphetamine addiction: a review of guidelines. Canadian Agency for Drugs and Technologies in Health 2019.

Lupi, Matteo, Martinotti, Giovanni, Santacroce, Rita, Cinosi, Eduardo, Carlucci, Maria, Marini, Stefano, Acciavatti, Tiziano, and Di Giannantonio, Massimo. Transcranial Direct Current Stimulation in Substance Use Disorders: A Systematic Review of Scientific Literature. The journal of ECT 2017. 33 (3): 203-209.

Ma, Tianye, Sun, Yurong, and Ku, Yixuan. Effects of Non-invasive Brain Stimulation on Stimulant Craving in Users of Cocaine, Amphetamine, or Methamphetamine: A Systematic Review and Meta-Analysis. Frontiers in neuroscience 2019. 13: 1095.

MacLean, Sarah, Harney, Angela, and Arabena, Kerry. Primary health-care responses to methamphetamine use in Australian Indigenous communities. Australian journal of primary health 2015. 21 (4): 384-390.

Makani, Ramkrishna, Pradhan, Basant, Shah, Umang, and Parikh, Tapan. Role of Repetitive Transcranial Magnetic Stimulation (rTMS) in Treatment of Addiction and Related Disorders: A Systematic Review. Current drug abuse reviews 2017. 10 (1): 31-43.

Manning, V, Arunogiri, S, and Frei, MR. Alcohol and other drug withdrawal: Practice guidelines. https://www.turningpoint.org.au/sites/default/files/inline-files/Alcohol-and-Drug-Withdrawal-Guidelines-2018.pdf 2018.

Martinotti, G., Chiappini, S., Pettorruso, M., Mosca, A., Miuli, A., Di Carlo, F., D'Andrea, G., Collevecchio, R., Di Muzio, I., Sensi, S. L., Di Giannantonio, M.. Therapeutic potentials of ketamine and esketamine in obsessive-compulsive disorder (Ocd), substance use disorders (sud) and eating disorders (ed): A review of the current literature. 2021. 11(7) (no pagination)

Masroor, A., Khorochkov, A., Prieto, J., Singh, K. B., Nnadozie, M. C., Abdal, M., Shrestha, N., Abe, R. A. M., Mohammed, L.. Unraveling the Association Between Schizophrenia and Substance Use Disorder-Predictors, Mechanisms and Treatment Modifications: A Systematic Review. 2021. 13 (7): e16722.

McGovern, R., Newham, J. J., Addison, M. T., Hickman, M., Kaner, E. F.. Effectiveness of psychosocial interventions for reducing parental substance misuse. 2021. 3: CD012823.

Motlagh, F. E., Ibrahim, F., Rashid, R. A., Seghatoleslam, T., and Habil, H.. Acupuncture therapy for drug addiction. Chinese Medicine (United Kingdom) 2016. 11 (1): 16.

Ozgen, H., Spijkerman, R., Noack, M., Holtmann, M., Schellekens, A. S. A., van de Glind, G., Banaschewski, T., Barta, C., Begeman, A., Casas, M., Crunelle, C. L., Daigre Blanco, C., Dalsgaard, S., Demetrovics, Z., den Boer, J., Dom, G., Eapen, V., Faraone, S. V., Franck, J., Gonzalez, R. A., Grau-Lopez, L., Groenman, A. P., Hemphala, M., Icick, R., Johnson, B., Kaess, M., Kapitany-Foveny, M., Kasinathan, J. G., Kaye, S. S., Kiefer, F., Konstenius, M., Levin, F. R., Luderer, M., Martinotti, G., Matthys, F. I. A., Meszaros, G., Moggi, F., Munasur-Naidoo, A. P., Post, M., Rabinovitz, S., Ramos-Quiroga, J. A., Sala, R., Shafi, A., Slobodin, O., Staal, W. G., Thomasius, R., Truter, I., van Kernebeek, M. W., Velez-Pastrana, M. C., Vollstadt-Klein, S., Vorspan, F., Young, J. T., Yule, A., van den Brink, W., Hendriks, V.. International Consensus Statement for the Screening, Diagnosis, and Treatment of Adolescents with Concurrent Attention-Deficit/Hyperactivity Disorder and Substance Use Disorder. 2020. 26 (4-5): 223-232.

Posadzki, P., Khalil, M. M. K., AlBedah, A. M. N., Zhabenko, O., and Car, J.. Complementary and alternative medicine for addiction: an overview of systematic reviews. Focus on Alternative and Complementary Therapies 2016. 21 (2): 69-81.

Prud'homme, Melissa, Cata, Romulus, and Jutras-Aswad, Didier. Cannabidiol as an Intervention for Addictive Behaviors: A Systematic Review of the Evidence. Substance abuse: research and treatment 2015. 9: 33-38.

Quednow, Boris B. and Herdener, Marcus. Human pharmacology for addiction medicine: From evidence to clinical recommendations. Progress in brain research 2016. 224: 227-250.

Rash, Carla J., Alessi, Sheila M., and Petry, Nancy M.. Substance Abuse Treatment Patients in Housing Programs Respond to Contingency Management Interventions. Journal of substance abuse treatment 2017. 72: 97-102.

Ray, L. A., Meredith, L. R., Kiluk, B. D., Walthers, J., Carroll, K. M., Magill, M.. Combined Pharmacotherapy and Cognitive Behavioral Therapy for Adults With Alcohol or Substance Use Disorders: A Systematic Review and Meta-analysis. 2020. 3 (6): e208279.

Reynoard, J., Schmitt, C., Torrents, R., Simon, N.. Toxicological considerations in the prescription of baclofen for the treatment of substance use disorders. 2020. 16(4): 309-317.

Richards, John R., Albertson, Timothy E., Derlet, Robert W., Lange, Richard A., Olson, Kent R., and Horowitz, B. Zane. Treatment of toxicity from amphetamines, related derivatives, and analogues: a systematic clinical review. Drug and alcohol dependence 2015. 150: 1-13.

Roche, AM, Ryan, K, Fischer, J, and Nicholas, R. A Review of Australian Clinical Guidelines for Methamphetamine Use Disorder. https://nccred.org.au/wp-content/uploads/2019/09/2019-01_NCCRED_A-review-of-the-Clinical-Guidelines-for-Methamphetamine-Use-Disorder.pdf 2019. (Technical Report Number: 2019/01).

Ronsley, C., Nolan, S., Knight, R., Hayashi, K., Klimas, J., Walley, A., Wood, E., Fairbairn, N.. Treatment of stimulant use disorder: A systematic review of reviews. 2020. 15 (6): e0234809.

Rubenis, Adam J., Baker, Amanda L., Arunogiri, Shalini. Methamphetamine use and technology-mediated psychosocial interventions: A mini-review. 2021. 121

Secades-Villa, R., Garcia-Rodriguez, O., and Fernandez-Hermida, J. R.. Contingency management for substance use disorders in Spain: Implications for research and practice. Preventive medicine 2015. 80: 82-88.

Segawa, Tomoyuki, Baudry, Thomas, Bourla, Alexis, Blanc, Jean Victor, Peretti, Charles Siegfried, Mouchabac, Stephane, and Ferreri, Florian. Virtual Reality (VR) in Assessment and Treatment of Addictive Disorders: A Systematic Review. Frontiers in neuroscience 2019. 13: 1409.

Sepede, Gianna, Lorusso, Marco, Spano, Maria Chiara, Di Nanno, Piero, Di Iorio, Giuseppe, and Di Giannantonio, Massimo. Efficacy and Safety of Atypical Antipsychotics in Bipolar Disorder With Comorbid Substance Dependence: A Systematic Review. Clinical neuropharmacology 2018. 41 (5): 181-191.

Sharma, D. and Kar, S. K.. Recent advances in pharmacological management of substance use disorders. International Journal of Pharmaceutical Investigation 2015. 5 (4): 118-127.

Simpson, S. A., Chwastiak, L. A., Andrews, S. R., Bienvenu, O. J., Adler Cohen, M. A., Cozza, K. L., DiMartini, A., Fernandez-Robles, C., Grimaldi, J. A. R., Isenberg-Grzeda, E., King, Iv F., Kontos, N., Luchsinger, W., Munjal, S., Pathare, A., Pereira, L. F., Philbrick, K., Rosen, J. H., Schmelzer, N. A., Zimbrean, P. C.. Updates in Consultation-Liaison Psychiatry: 2019. 2020. 61(5): 450-455.

Soares, E. and Pereira, F. C.. Pharmacotherapeutic strategies for methamphetamine use disorder: mind the subgroups. Expert Opinion on Pharmacotherapy 2019. 20 (18): 2273-2293.

Stockings, Emily, Hall, Wayne D., Lynskey, Michael, Morley, Katherine I., Reavley, Nicola, Strang, John, Patton, George, and Degenhardt, Louisa. Prevention, early intervention, harm reduction, and treatment of substance use in young people. The lancet.Psychiatry 2016. 3 (3): 280-296.

Stockings, Emily, Hall, Wayne D., Lynskey, Michael, Morley, Katherine I., Reavley, Nicola, Strang, John, Patton, George, and Degenhardt, Louisa. Substance use in young people 3: Prevention, early intervention, harm reduction, and treatment of substance use in young people. The Lancet Psychiatry 2016. 3 (3): 280-296.

Stone, J, Marsh, A, Dale, A, Willis, L, O'Toole, S, Helfgott, S, and et al. Counselling guidelines: Alcohol and drug issues. Western Australian Mental Health Commission 2019.

Stuart, Alexandra, Baker, Amanda L., Bowman, Jenny, McCarter, Kristen, Denham, Alexandra Mary Janice, Lee, Nicole, Colyvas, Kim, and Dunlop, Adrian. Protocol for a systematic review of psychological treatment for methamphetamine use: an analysis of methamphetamine use and mental health symptom outcomes. BMJ open 2017. 7 (9): e015383.

Tardelli, V. S., Lago, M. P. P. D., Mendez, M., Bisaga, A., and Fidalgo, T. M.. Contingency Management with pharmacologic treatment for Stimulant Use Disorders: A review. Behaviour Research and Therapy 2018. 111: 57-63.

The Royal Women's Hospital.. Drug and Alcohol - Management of Methamphetamine Dependence in Pregnancy. https://thewomens.r.worldssl.net/images/uploads/downloadable-records/clinical-guidelines/drug-and-alcohol-management-methamphetamine-dependence-in-pregnancy_160517.pdf 2017.

Tomko, R. L., Bountress, K. E., and Gray, K. M.. Personalizing substance use treatment based on pre-treatment impulsivity and sensation seeking: A review. Drug and alcohol dependence 2016. 167: 1-7.

Werner, F.-M. and Covenas, R.. Long-term administration of antipsychotic drugs in schizophrenia and influence of substance and drug abuse on the disease outcome. Current drug abuse reviews 2017. 10 (1): 19-24.

Wittenauer, J., Ascher, M., Briggie, A., Kreiter, A., and Chavez, J.. The role of complementary and alternative medicine in adolescent substance use disorders. Adolescent Psychiatry 2015. 5 (2): 96-104.

Yadollahpour, A. and Yuan, T.. Transcranial direct current stimulation for the treatment of addictions: A systematic review of clinical trials. Current Psychiatry Reviews 2018. 14 (4): 221-229.

Yatham, L. N., Kennedy, S. H., Parikh, S. V., Schaffer, A., Bond, D. J., Frey, B. N., Sharma, V., Goldstein, B. I., Rej, S., Beaulieu, S., Alda, M., MacQueen, G., Milev, R. V., Ravindran, A., O'Donovan, C., McIntosh, D., Lam, R. W., Vazquez, G., Kapczinski, F., McIntyre, R. S., Kozicky, J., Kanba, S., Lafer, B., Suppes, T., Calabrese, J. R., Vieta, E., Malhi, G., Post, R. M., and Berk, M.. Canadian Network for Mood and Anxiety Treatments (CANMAT) and International Society for Bipolar Disorders (ISBD) 2018 guidelines for the management of patients with bipolar disorder. Bipolar disorders 2018. 20 (2): 97-170.

Zaso, Michelle J., Park, Aesoon, and Antshel, Kevin M.. Treatments for Adolescents With Comorbid ADHD and Substance Use Disorder: A Systematic Review. Journal of attention disorders 2015.

### No data specific to methamphetamine use disorder or problematic methamphetamine use

Chan, B., Freeman, M., Ayers, C., Korthuis, P. T., Paynter, R., Kondo, K., Kansagara, D.. A systematic review and meta-analysis of medications for stimulant use disorders in patients with co-occurring opioid use disorders. 2020. 216: 108193.

Cook, Jon, Lloyd-Jones, Martyn, Arunogiri, Shalini, Ogden, Edward, and Bonomo, Yvonne. Managing attention deficit hyperactivity disorder in adults using illicit psychostimulants: A systematic review. The Australian and New Zealand journal of psychiatry 2017. 51 (9): 876-885.

De Crescenzo, Franco, Ciabattini, Marco, D'Alo, Gian Loreto, De Giorgi, Riccardo, Del Giovane, Cinzia, Cassar, Carolina, Janiri, Luigi, Clark, Nicolas, Ostacher, Michael Joshua, and Cipriani, Andrea. Comparative efficacy and acceptability of psychosocial interventions for individuals with cocaine and amphetamine addiction: A systematic review and network meta-analysis. PLoS medicine 2018. 15 (12): e1002715.

Dugdale, S., Elison-Davies, S., Semper, H., Ward, J., and Davies, G.. Are Computer-Based Treatment Programs Effective at Reducing Symptoms of Substance Misuse and Mental Health Difficulties Within Adults? A Systematic Review. Journal of Dual Diagnosis 2019. 15 (4): 291-311.

Harada, Takayuki, Tsutomi, Hiroshi, Mori, Rintaro, and Wilson, David B.. Cognitive-behavioural treatment for amphetamine-type stimulants (ATS)-use disorders. The Cochrane database of systematic reviews 2018. 12: CD011315.

Sheridan Rains, L., Steare, T., Mason, O., Johnson, S.. Improving substance misuse outcomes in contingency management treatment with adjunctive formal psychotherapy: A systematic review and meta-analysis. 2020. 10(10) (no pagination)

Tardelli, V. S., Bisaga, A., Arcadepani, F. B., Gerra, G., Levin, F. R., Fidalgo, T. M.. Prescription psychostimulants for the treatment of stimulant use disorder: a systematic review and meta-analysis. 2020. 237 (8): 2233-2255.

Ainscough, Tom S., McNeill, Ann, Strang, John, Calder, Robert, and Brose, Leonie S.. Contingency management interventions for non-prescribed drug use during treatment for opiate addiction: A systematic review and meta-analysis. Drug and alcohol dependence 2017. 178: 318-339.

Berkovitch, L., Romeo, B., Karila, L., Gaillard, R., Benyamina, A.. [Efficacy of psychedelics in psychiatry, a systematic review of the literature]. 2021. 47 (4): 376-387.

Bolivar, H. A., Klemperer, E. M., Coleman, S. R. M., DeSarno, M., Skelly, J. M., Higgins, S. T.. Contingency Management for Patients Receiving Medication for Opioid Use Disorder: A Systematic Review and Meta-analysis. 2021. 78 (10): 1092-1102.

Boumparis, Nikolaos, Karyotaki, Eirini, Schaub, Michael P., Cuijpers, Pim, and Riper, Heleen. Internet interventions for adult illicit substance users: a meta-analysis. Addiction (Abingdon, England) 2017. 112 (9): 1521-1532.

Brookfield, Samuel, Fitzgerald, Lisa, Selvey, Linda, and Maher, Lisa. Turning points, identity, and social capital: A meta-ethnography of methamphetamine recovery. The International journal on drug policy 2019. 67: 79-90.

Cunill, R., Castells, X., Tobias, A., and Capella, D.. Pharmacological treatment of attention deficit hyperactivity disorder with co-morbid drug dependence. Journal of psychopharmacology (Oxford, England) 2015. 29 (1): 15-23.

Davis, J. P., Smith, D. C., and Briley, D. A.. Substance use prevention and treatment outcomes for emerging adults in non-college settings: A meta-Analysis. Psychology of Addictive Behaviors 2017. 31 (3): 242-254.

Fluyau, D., Revadigar, N., Pierre, C. G.. Systematic Review and Meta-Analysis: Treatment of Substance Use Disorder in Attention Deficit Hyperactivity Disorder. 2021. 30(2): 110-121.

Hayhurst, Karen P., Leitner, Maria, Davies, Linda, Flentje, Rachel, Millar, Tim, Jones, Andrew, King, Carlene, Donmall, Michael, Farrell, Michael, Fazel, Seena, Harris, Rochelle, Hickman, Matthew, Lennox, Charlotte, Mayet, Soraya, Senior, Jane, and Shaw, Jennifer. The effectiveness and cost-effectiveness of diversion and aftercare programmes for offenders using class A drugs: a systematic review and economic evaluation. Health technology assessment (Winchester, England) 2015. 19 (6): 1-viii.

Jeal, N., MacLeod, J., Turner, K., and Salisbury, C.. Systematic review of interventions to reduce illicit drug use in female drug-dependent street sex workers. BMJ open 2015. 5 (11): e009238.

Kedzior, Karina Karolina, Gerkensmeier, Imke, and Schuchinsky, Maria. Can deep transcranial magnetic stimulation (DTMS) be used to treat substance use disorders (SUD)? A systematic review. BMC psychiatry 2018. 18 (1): 137.

Krause, Marc, Huhn, Maximilian, Schneider-Thoma, Johannes, Bighelli, Irene, Gutsmiedl, Katharina, and Leucht, Stefan. Efficacy, acceptability and tolerability of antipsychotics in patients with schizophrenia and comorbid substance use. A systematic review and meta-analysis. European neuropsychopharmacology: the journal of the European College of Neuropsychopharmacology 2019. 29 (1): 32-45.

Martinez-Vispo, Carmela, Martinez, Ursula, Lopez-Duran, Ana, Fernandez Del Rio, Elena, and Becona, Elisardo. Effects of behavioural activation on substance use and depression: a systematic review. Substance abuse treatment, prevention, and policy 2018. 13 (1): 36.

Morel, A., Lebard, P., Dereux, A., Azuar, J., Questel, F., Bellivier, F., Marie-Claire, C., Fatseas, M., Vorspan, F., Bloch, V.. Clinical Trials of Cannabidiol for Substance Use Disorders: Outcome Measures, Surrogate Endpoints, and Biomarkers. 2021. 12 (no pagination)

Morris, Linzette, Stander, Jessica, Ebrahim, Wardah, Eksteen, Stephanie, Meaden, Orissa Anna, Ras, Ane, and Wessels, Annemarie. Effect of exercise versus cognitive behavioural therapy or no intervention on anxiety, depression, fitness and quality of life in adults with previous methamphetamine dependency: a systematic review. Addiction science & clinical practice 2018. 13 (1): 4.

Ozgen, H., Spijkerman, R., Noack, M., Holtmann, M., Schellekens, A., Dalsgaard, S., van den Brink, W., Hendriks, V.. Treatment of adolescents with concurrent substance use disorder and attention-deficit/hyperactivity disorder: A systematic review. 2021. 10(17) (no pagination)

Perry, Amanda E., Martyn-St James, Marrissa, Burns, Lucy, Hewitt, Catherine, Glanville, Julie M., Aboaja, Anne, Thakkar, Pratish, Santosh Kumar, Keshava Murthy, Pearson, Caroline, and Wright, Kath. Interventions for female drug-using offenders. The Cochrane database of systematic reviews 2019. 12: CD010910.

Rose-Clarke, K., Bentley, A., Marston, C., and Prost, A.. Peer-facilitated community-based interventions for adolescent health in low- and middle-income countries: A systematic review. PloS one 2019. 14 (1): e0210468.

Sayegh, C. S., Huey, S. J., Zara, E. J., and Jhaveri, K.. Follow-up treatment effects of contingency management and motivational interviewing on substance use: A meta-analysis. Psychology of Addictive Behaviors 2017. 31 (4): 403-414.

Srisurapanont, M., Likhitsathian, S., Suttajit, S., Maneeton, N., Maneeton, B., Oon-arom, A., Suradom, C.. Efficacy and dropout rates of antipsychotic medications for methamphetamine psychosis: A systematic review and network meta-analysis. 2021. 219 (no pagination)

### MUD/PMU combined with other substances/ study by study results/ no eligible studies

Minozzi S, Saulle R, De Crescenzo F, Amato L. Psychosocial interventions for psychostimulant

misuse. Cochrane Database Syst Rev. 2016 Sep 29;9:CD011866.

Tofighi B, Nicholson JM, McNeely J, Muench F, Lee JD. Mobile phone messaging for illicit drug and alcohol dependence: A systematic review of the literature. Drug Alcohol Rev. 2017 Jul;36(4):477–91.

De Giorgi R, Cassar C, Loreto D’alò G, Ciabattini M, Minozzi S, Economou A, et al. Psychosocial interventions in stimulant use disorders: a systematic review and qualitative synthesis of randomized controlled trials. Riv Psichiatr. 2018 Oct;53(5):233-55.

Lee NK, Jenner L, Harney A, Cameron J. Pharmacotherapy for amphetamine dependence: A systematic review. Drug Alcohol Depend. 2018 Oct 1;191:309–37.

Klimas J, Fairgrieve C, Tobin H, Field C-A, O’Gorman CS, Glynn LG, et al. Psychosocial interventions to reduce alcohol consumption in concurrent problem alcohol and illicit drug users. Cochrane Database Syst Rev. 2018 Dec 5;12:CD009269.

Knight R, Karamouzian M, Carson A, Edward J, Carrieri P, Shoveller J, et al. Interventions to address substance use and sexual risk among gay, bisexual and other men who have sex with men who use methamphetamine: A systematic review. Drug Alcohol Depend. 2019 01;194:410–29.

Lam L, Anand S, Li X, Tse ML, Zhao JX, Chan EW. Efficacy and safety of naltrexone for amfetamine and methamfetamine use disorder: a systematic review of randomized controlled trials. Clin Toxicol (Phila). 2019 Apr;57(4):225–33.

Gray C, Argaez C. Residential treatment for substance use disorder. Ottawa: CADTH. 2019 CADTH rapid response report: summary with critical appraisal.

Fluyau D, Mitra P, Lorthe K. Antipsychotics for Amphetamine Psychosis. A Systematic Review.

Front Psychiatry. 2019;10:740.

Clarke M, Featherstone R. Management of Acute Withdrawal and Detoxification for Adults who Misuse Methamphetamine: A Review of the Clinical Evidence and Guidelines. Ottawa: CADTH. 2019 CADTH rapid response report: summary with critical appraisal.

Khoramizadeh M, Effatpanah M, Mostaghimi A, Rezaei M, Mahjoub A, Shishehgar S. Treatment of amphetamine abuse/use disorder: a systematic review of a recent health concern. Daru. 2019 Dec;27(2):743–53.

Chan B, Freeman M, Kondo K, Ayers C, Montgomery J, Paynter R, et al. Pharmacotherapy for methamphetamine/amphetamine use disorder-a systematic review and meta-analysis. Addiction. 2019 Dec;114(12):2122–36.

Wells C, Loshak H, Dulong C. Withdrawal management and treatment of crystal methamphetamine addiction in pregnancy: a review of clinical effectiveness and guidelines. Ottawa: CADTH. 2019 CADTH rapid response report: summary with critical appraisal.

Stuart AM, Baker AL, Denham AMJ, Lee NK, Hall A, Oldmeadow C, et al. Psychological treatment for methamphetamine use and associated psychiatric symptom outcomes: A systematic review. J Subst Abuse Treat. 2020 Feb;109:61–79.

Siefried KJ, Acheson LS, Lintzeris N, Ezard N. Pharmacological Treatment of Methamphetamine/Amphetamine Dependence: A Systematic Review. CNS Drugs. 2020 Apr;34(4):337–65.

Steele D, Becker S, Danko K, Balk E et al. Interventions for Substance Use Disorders in

Adolescents: A Systematic Review [Internet]. Agency for Healthcare Research and Quality; 2020. Report No.: Comparative Effectiveness Review # 225. Available from: https://www.ncbi.nlm.nih.gov/books/NBK557291/pdf/Bookshelf_NBK557291.pdf

AshaRani PV, Hombali A, Seow E, Ong WJ, Tan JH, Subramaniam M. Non-pharmacological interventions for methamphetamine use disorder: a systematic review. Drug Alcohol Depend. 2020 Jul 1;212:108060.

Stokes PRA, Jokinen T, Amawi S, Qureshi M, Husain MI, Yatham LN, et al. Pharmacological Treatment of Mood Disorders and Comorbid Addictions: A Systematic Review and Meta-Analysis: Traitement Pharmacologique des Troubles de L’humeur et des Dépendances Comorbides: Une Revue Systématique et une Méta-Analyse. Can J Psychiatry. 2020 Nov;65(11):749–69.

### Other reasons for exclusion (i.e., only MUD/PMU data from a review)

Banerjee, S and Spry, C. Concurrent treatment for substance use disorder and trauma-related comorbidities: a review of clinical effectiveness and guidelines. Canadian Agency for Drugs and Technologies in Health 2017.

## Primary studies

### Full-text not available

Aharonovich E, Hasin DS. Primary drug use types and intervention-related self-monitoring in HIV patients. Drug and alcohol dependence 2015. 146 (#Issue#): e203-#End Page#.

Aharonovich E, Hasin DS Stohl. HealthCall: technology use to reduce non-injection drug use in HIV primary care. Drug and alcohol dependence 2015. 156 (#Issue#): e3-#End Page#.

Alipour, M., Jafarian, M., Rastgoo, R., Mokri, A., Gorji, A., Zarrindast, M. R., Lorestani, F., Razaghi, E. M.. Cabergoline in Treatment of Methamphetamine-Dependent Patients and Its Effect on Serum Level of Glial Cell-Derived Neurotrophic Factor: A Randomized, Double-Blind, Placebo-Controlled Clinical Trial. 2021. 27 (6): 457-468-#End Page#.

Ang, A.. Methylphenidate for methamphetamine use disorders in participants with and without ADHD. Drug and alcohol dependence 2015. 156 (#Issue#): e7-#End Page#.

Arechiga, G.. Naltrexone implants improve therapeutic adherence with multiple substance use disorder. Journal of addiction medicine 2019. 13 (3): E13-#End Page#.

Batki SL, Moon J.. Amlodipine treatment of methamphetamine dependence,a controlled outpatient trial: preliminary analysis. Drug and alcohol dependence 2001. 63 Suppl 1 (#Issue#): 12-#End Page#.

Batki, SL, Moon, J, and Bradley, M. Fluoxetine in methamphetamine dependence - a controlled trial: a preliminary analysis. The 61st Annual Scientific Meeting of the College on Problems of Drug Dependence. 1999. #volume# (#Issue#): 235-#End Page#.

Braeckman, R.. Dose-Finding Study of Abuse-Related Effects of Intranasal D-Methylphenidate in Recreational Stimulant Abusers. Journal of the American Academy of Child and Adolescent Psychiatry 2018. 57 (10): S176-#End Page#.

Brensilver, M.. Relation of immediate treatment response and end-of-trial outcomes in placebo-treated methamphetamine-dependent patients. Proceedings of the 74th Annual Scientific Meeting of the College on Problems of Drug Dependence; 2012. #volume# (#Issue#): Abstract-#End Page#.

Briones, M.. Varenicline for the treatment of methamphetamine dependence. Drug and alcohol dependence 2015. 156 (#Issue#): e28-#End Page#.

Brown ES, Nejtek VA Perantie DC. Neuroleptics and quetiapine in psychiatric illnesses with comorbid stimulant abuse. International journal of neuropsychopharmacology (abstracts of the 23rd congress of the collegium internationale neuro-psychopharmacologicum; june 23-27 2002. 5 (Suppl 1): S157-#End Page#.

Caillard, I.. Trying to overcome alcoholism and drug addiction. Biofutur 2001. #volume# (211): 21-#End Page#.

Choo E, Zlotnick. BSAFER: A Web intervention for women in the ED with drug use and IPV. Drug and alcohol dependence 2015. 156 (#Issue#): e43-#End Page#.

Courtney KE, Ghahremani D.. The effects of naltrexone on neural responses to methamphetamine cues. Drug and alcohol dependence 2017. 171 (#Issue#): e47-#End Page#.

Das-Douglas, M.. Actively-using, non-treatment-seeking men who have sex with men can be successfully enrolled and retained in pharmacologic studies for methamphetamine dependence. Proceedings of the 71th annual scientific meeting of the college on problems of drug dependence; 2009. #volume# (#Issue#): 29-#End Page#.

De La Garza, R.. The acetylcholinesterase inhibitor rivastigmine reduces medthamphetamine-induced 'desire METH' and 'likely to use METH' in methamphetamine-dependent volunteers. Neuropsychopharmacology : official publication of the American College of Neuropsychopharmacology 2010. 35 (Suppl 1): S389-#End Page#.

De, Young D.. Cardiovascular safety of ibudilast treatment with intravenous methamphetamine administration. Drug and alcohol dependence 2015. 146 (#Issue#): e265-#End Page#.

DeHardt, T.. Psychosocial treatment dose and prospective addiction treatment outcomes in a stimulant-dependent sample. Proceedings of the 65th annual scientific meeting of the college on problems of drug dependence; 2003. #volume# (#Issue#): 39-#End Page#.

Delker E, Cannizzaro. Contrasting trajectories of intervention-related self-monitoring in HIV primary care patients. Drug and alcohol dependence 2015. 156 (#Issue#): e56-#End Page#.

Elkashef AM, Rawson RA Smith E Anderson. Buproprion for the treatment of methamphetamine dependence. Proceedings of the 68th annual scientific meeting of the college on problems of drug dependence; 2006. #volume# (#Issue#): -#End Page#.

Fletcher JB, Landovitz RJ Reback CJ. Contingency management vs. non-contingent rewards: Intervention response patterns among stimulant-using MSM. Drug and alcohol dependence 2015. 156 (#Issue#): e70-#End Page#.

Forcehimes AA, Bogenschutz M.. Race and ethnicity differences in a MI-based brief intervention delivered in an ED setting. Drug and alcohol dependence 2015. 146 (#Issue#): e280-#End Page#.

Fukuhara, T., Sakuta, T., and Onishi, K.. Study on methamphetamine dependence. International Medical Journal 1996. 3 (3): 229-235.

Galea S, Walters. Methamphetamine addiction-the practicalities of researching 'P' addiction. Australian and New Zealand Journal of Psychiatry 2011. 45 (#Issue#): A60-#End Page#.

Galloway GP, Fiske L.. Dextroamphetamine as a treatment for methamphetamine dependence. Proceedings of the 71th annual scientific meeting of the college on problems of drug dependence; 2009. #volume# (#Issue#): 44-#End Page#.

Galloway, G. P.. A randomized, placebo-controlled trial of vigabatrin for methamphetamine dependence. Clinical pharmacology and therapeutics 2011. 89 (#Issue#): S70-#End Page#.

Ghahremani DG, Tabibnia G.. Modafinil improves cognitive performance in methamphetamine abusers: evidence from human behavioral and FMRI studies. Proceedings of the 71th annual scientific meeting of the college on problems of drug dependence; 2009. #volume# (#Issue#): 47-#End Page#.

Ghahremani, D.. Modafinil-induced enhancement of learning and related fMRI activation in humans reflects individual differences in striatal dopamine D2/D3 receptor availability. Neuropsychopharmacology : official publication of the American College of Neuropsychopharmacology 2013. 38 (#Issue#): S200-#End Page#.

Glasner-Edwards S, Farabee. Effects of psychosocial treatment dose on outcomes in stimulant-dependent adults. Proceedings of the 71th annual scientific meeting of the college on problems of drug dependence; 2009. #volume# (#Issue#): 47-#End Page#.

Glasner-Edwards S, Mooney. Mindfulness based relapse prevention improves stimulant use among adults with major depression and generalized anxiety disorder. Drug and alcohol dependence 2015. 156 (#Issue#): e80-#End Page#.

Haile CN, Verrico CD Thompson-Lake DG Mahoney JJ Kosten. The angiotensin receptor blocker candesartan attenuates the subjective effects of methamphetamine in humans. Drug and alcohol dependence 2015. 156 (#Issue#): e88-#End Page#.

Haning III, William F.. Methamphetamine treatment: what psychiatrists need to know to: substance abuse and mental health services administration. 155th annual meeting of the american psychiatric association; 2002. #volume# (#Issue#): -#End Page#.

Heinzerling KG, Shoptaw S Swanson. Randomized, double-blinde trial of modafinil vs placebo for methamphetamine dependence. Proceedings of the 71th annual scientific meeting of the college on problems of drug dependence; 2009. #volume# (#Issue#): 58-#End Page#.

Hospital MM, Graziano JN Morris SL Wagner EF. Examining the effectiveness of a school-based motivational interviewing alcohol intervention with minority adolescents. Alcoholism: Clinical and Experimental Research 2012. 36 (#Issue#): 243A-#End Page#.

Huber, A.. SErtraline and contingency management as treatment for methamphetamine dependence. Proceedings of the 63rd annual scientific meeting of college on problems of drug dependence; 2001. #volume# (#Issue#): S95-#End Page#.

Jackson BJ, Kalechstein AD De La Garza. An evaluation of the effects of rivastigmine on neurocognition in methamphetamine-dependent volunteers. Proceedings of the 68th annual scientific meeting of the college on problems of drug dependence; 2006. #volume# (#Issue#): -#End Page#.

Jaffe JH, Mitchell SG Gryczynksi. IOP vs. OP with Buprenorphine: 6-month Treatment Outcomes. American Journal on Addictions 2013. 22 (3): 318-#End Page#.

Johnson BA, Rawson RA Elkashef. Ondansetron for the treatment of methamphetamine dependence. Proceedings of the 66th annual scientific meeting of college on problems of drug dependence; 2004. #volume# (#Issue#): -#End Page#.

Kalechstein AD, De La Garza. Effects of Bupropion SR on neurocognition in volunteers with methamphetamine dependence. Proceedings of the 68th annual scientific meeting of the college on problems of drug dependence; 2006. #volume# (#Issue#): -#End Page#.

Karami, F., Assarian, F., Ghoreishi, F. S., Sehat, M.. The efficacy of n-acetilcysteine for the treatment of the metamfetamin-addicted patients under methadon therapy: A double-blind clinical trial. 2020. 22(7) (no pagination) (#Issue#): -#End Page#.

Kim TW, Bernstein J.. Does screening and brief intervention for drug use in primary care increase receipt of substance use disorder treatment?. Drug and alcohol dependence 2015. 156 (#Issue#): e111-#End Page#.

Konstenius, M.. Pharmacological treatment of ADHD with amphetamine dependence. Acta Neuropsychiatrica 2013. 25 (1 SUPPL. 1): 13-#End Page#.

Leelahanaj, Thawatchai, Kongsakon, Ronnachai, and Netrakom, Pongsatorn. A 4-week, double-blind comparison of olanzapine with haloperidol in the treatment of amphetamine psychosis. Journal of the Medical Association of Thailand = Chotmaihet thangphaet 2005. 88 Suppl 3 (#Issue#): S43-S52.

Leickly E, McDonell. Successful contingency management for alcohol generalizes to stimulant use reduction. Journal of addiction medicine 2016. 10 (3): E14-#End Page#.

Ling, K.. Immediate rewards improve outcomes for methamphetamine addiction: a behavioral economic analysis of a contingency management treatment program. Proceedings of the 73rd annual scientific meeting of the college on problems of drug dependence; 2011. #volume# (#Issue#): 102-#End Page#.

Ling, W.. Sustained-release methylphenidate for the treatment of methamphetamine dependence. Drug and alcohol dependence 2015. 146 (#Issue#): e180-#End Page#.

Mahoney JJ, De La Garza. Effects of rivastigmine treatment on intravenous self-administration of methamphetamine in methamphetamine-dependent volunteers. Proceedings of the 68th annual scientific meeting of the college on problems of drug dependence; 2006. #volume# (#Issue#): -#End Page#.

Mancino MJ, McGaugh J.. Efficacy and tolerability of doxazosin in psychostimulant-dependent patients. Drug and alcohol dependence 2015. 156 (#Issue#): e138-#End Page#.

Mancino MJ, McGaugh J.. D-amphetamine withdrawal paradigm in methamphetamine dependence. Proceedings of the 73rd annual scientific meeting of the college on problems of drug dependence; 2011. #volume# (#Issue#): 109-#End Page#.

Mancino MJ, Thostenson JD Guise JB McGaugh. Impact of lisdexamfetamine on retention of methamphetamine-dependent patients in a residential facility. Drug and alcohol dependence 2017. 171 (#Issue#): e128-#End Page#.

Meli S, Palfai. Screening and brief intervention for low risk drug use in primary care: a pilot randomized trial. Drug and alcohol dependence 2015. 156 (#Issue#): e149-#End Page#.

Mooney, L.. Methamphetamine dependence, psychiatric disorders, and treatment outcomes in individuals treated with methylphenidate. Drug and alcohol dependence 2015. 146 (#Issue#): e44-#End Page#.

Murphy SM, McDonell MG McPherson S Srebnik. Assessing the cost-effectiveness of a contingency-management intervention for stimulant use among community mental health patients with serious mental illness. Drug and alcohol dependence 2015. 156 (#Issue#): e160-#End Page#.

Naber, D.. Effects of aripiprazole once-monthly and paliperidone palmitate in patients with schizophrenia and concomitant substance use: A post-hoc analysis of QUALIFY, a head-to-head study. Neuropsychopharmacology : official publication of the American College of Neuropsychopharmacology 2015. 40 (#Issue#): S539-#End Page#.

Papaseit E, Perez-Mana. Human pharmacology of mephedrone: A dose-finding pilot study. Drug and alcohol dependence 2015. 146 (#Issue#): e61-#End Page#.

Peck, J.. Integrating substance abuse treatment with HIV prevention: treatment outcomes among gay male methamphetamine abusers in Hollywood, CA. Drug and alcohol dependence 2002. 66 Suppl 1 (#Issue#): -#End Page#.

Polcin DL, Korcha RA Nayak. Methamphetamine dependence and intensive motivational interviewing. Drug and alcohol dependence 2015. 146 (#Issue#): e72-#End Page#.

Prather, R.. Enhancing performance through increasing stress: Could nicotine help?. Alcohol (Fayetteville, N.Y.) 2011. 45 (3): 294-#End Page#.

Rashid RA, Habil MH Sulaiman AH Ahmad Zahari MM Hanif MA Seghatoleslam. Outcomes of aripirazole versus risperidone among methamphetamine dependence individuals seeking treatment in Malaysia. Alcohol and Alcoholism 2014. 49 (#Issue#): i36-#End Page#.

Reback CJ, Preston KL Scherer EA Liang. Technology-based interventions to addiction research. Journal of Neuroimmune Pharmacology 2017. 12 (2): S89-#End Page#.

Rush CR, Pike E Stoops WW. Influence of buspirone on the cardiovascular and subject-rated effects of methamphetamine. Drug and alcohol dependence 2015. 146 (#Issue#): e85-#End Page#.

Saldana, L.. Integrated treatment for mothers involved in child welfare for substance abuse. Drug and alcohol dependence 2015. 146 (#Issue#): e87-#End Page#.

Santos GM, Das M.. Adherence in pharmacotherapy trials among methamphetamine-dependent men who have sex with men (MSM). American journal of epidemiology 2011. 173 (#Issue#): S128-#End Page#.

Schottenfeld RS, Chawarski MC Sofuoglu. Pilot study of atomoxetine for BUP/NX-maintained patients with co-occurring opioid and ATS use disorder. Drug and alcohol dependence 2015. 156 (#Issue#): e200-#End Page#.

Shapiro HM, Smayda K.. Early prediction of high-risk patients is an opportunity for early intervention. Journal of addiction medicine 2019. 13 (3): E26-#End Page#.

Shoptaw S, Peck. A randomized, placebo-controlled trial of gabapentin or baclofen for methamphetamine dependence. Proceedings of the 67th annual scientific meeting of the college on problems of drug dependence; 2005. #volume# (#Issue#): -#End Page#.

Shoptaw S, Reback CJ Yang. Differential outcomes in a randomized trial of behavioral drug therapies for reducing drug use and sexual risk behaviors among gay and bisexual male methamphetamine abusers in Los Angeles. Los angeles, CA: friends research institute/ucla 2002. #volume# (#Issue#): -#End Page#.

Shoptaw, S.. Safety and early efficacy of ibudilast as a pharmacotherapy for methamphetamine addiction. Neuropsychopharmacology : official publication of the American College of Neuropsychopharmacology 2014. 39 (#Issue#): S105-#End Page#.

Shram, M.. An exploratory human abuse potential assessment of centanafadine, a novel triple reuptake inhibitor. Drug and alcohol dependence 2015. 156 (#Issue#): e203-#End Page#.

Stoops WW, Pike E Hays LR Glaser PE Rush CR. Influence of bupropion, naltrexone and bupropion + naltrexone on methamphetamine self-administration in humans. Drug and alcohol dependence 2015. 146 (#Issue#): e109-#End Page#.

Swanson, A, Shoptaw, S, and Heinzerling, KG. Varenicline for the treatment of methamphetamine dependence: a pilot study.. Proceedings of the 73rd Annual Scientific Meeting of the College on Problems of Drug Dependence 2011. #volume# (#Issue#): -#End Page#.

Urchel HC, Hanselka LL Baron. A controlled trial of flumazinil, gabapentin and hydroxyzine in treatment of methamphetamine dependence. Proceedings of the 70th annual scientific meeting of the college on problems of drug dependence; 2008. #volume# (#Issue#): -#End Page#.

Verrico CD, Mahoney JJ Bennett RS Newton TF De La Garza. Varenicline attenuates methamphetamine-induced subjective effects in methamphetamine-dependent volunteers. Neuropsychopharmacology : official publication of the American College of Neuropsychopharmacology 2012. 38 (#Issue#): S331-#End Page#.

Walker DD, Miller WR Ogle. The Influence of Significant Others in the Treatment of Cocaine and Methamphetamine Dependence: a Prospective Study. Drug and alcohol dependence 2002. 66 Suppl 1 (#Issue#): -#End Page#.

### Language other than English or French

Harada, Takayuki. [The randomized controlled trial of the prison-based Japanese Matrix Program (J-MAT) for methamphetamine abusers]. Nihon Arukoru Yakubutsu Igakkai zasshi = Japanese journal of alcohol studies & drug dependence 2012. 47 (6): 298-307.

Ikeda, Tomohiro, Umeno, Mitsuru, Morita, Nobuaki, Akiniwa, Hideki, and Nakatani, Yoji. [Two methods of support for methamphetamine concurrent disorder--comparison of "schizophrenia support" model and "dependence support" model]. Nihon Arukoru Yakubutsu Igakkai zasshi = Japanese journal of alcohol studies & drug dependence 2010. 45 (2): 92-103.

### Ineligible study design

Innovative approaches to drug abuse treatment. NIDA research monograph 1994. 140 (#Issue#): 61-64.

Anglin MD, Rawson RA. The CSAT Methamphetamine Treatment Project: what are we trying to accomplish?. Journal of psychoactive drugs 2000. 32 (2): 209-#End Page#.

Bachmann, K. M., Moggi, F., Hirsbrunner, H.-P., Donati, R., and Brodbeck, J.. An integrated treatment program for dually diagnosed patients. Psychiatric Services 1997. 48 (3): 314-316.

Bogenschutz, M. P.. It's time to take psilocybin seriously as a possible treatment for substance use disorders. American Journal of Drug and Alcohol Abuse 2017. 43 (1): 4-6.

Brady, K. T., Killeen, T., Baker, N. L.. Efficacy of mindfulness-based relapse prevention in veterans with substance use disorders: Design and methodology of a randomized clinical trial. 2021. 105 (no pagination).

Carrico, Adam W., Jain, Jennifer, Discepola, Michael V., Olem, David, Andrews, Rick, Woods, William J., Neilands, Torsten B., Shoptaw, Steven, Gomez, Walter, Dilworth, Samantha E., and Moskowitz, Judith T.. A community-engaged randomized controlled trial of an integrative intervention with HIV-positive, methamphetamine-using men who have sex with men. BMC public health 2016. 16: 673-.

Carrico, Adam W., Flentje, Annesa, Gruber, Valerie A., Woods, William J., Discepola, Michael V., Dilworth, Samantha E., Neilands, Torsten B., Jain, Jennifer, and Siever, Michael D.. Community-based harm reduction substance abuse treatment with methamphetamine-using men who have sex with men. Journal of urban health: bulletin of the New York Academy of Medicine 2014. 91 (3): 555-567.

Chavkin, W., Paone, D., Friedmann, P., and Wilets, I.. Reframing the debate: toward effective treatment for inner city drug-abusing mothers. Bulletin of the New York Academy of Medicine 1993. 70 (1): 50-68.

Chen, I. Chun, Teng, Gloria, Chen, Chur Jen, Lan, Tsuo Hung, and Liu, Hung Jen. The Autonomic Progress Bar Motivates Treatment Completion for Patients of Stimulant Use Disorder and Cannabis Use Disorder. Frontiers in psychiatry 2019. 10 (#Issue#): 944-#End Page#.

Chen, Xi Jing, Wang, Dong Mei, Zhou, Li Dan, Winkler, Markus, Pauli, Paul, Sui, Nan, and Li, Yong Hui. Mindfulness-based relapse prevention combined with virtual reality cue exposure for methamphetamine use disorder: Study protocol for a randomized controlled trial. Contemporary clinical trials 2018. 70: 99-105.

Collins, B. J., Cuddy, K., and Martin, A. P. Assessing the effectiveness and cost-effectiveness of drug intervention programs: UK case study. Journal of addictive diseases 2017. 36 (1): 5-13.

Dean, Andy C., London, Edythe D., Sugar, Catherine A., Kitchen, Christina M. R., Swanson, Aimee Noelle, Heinzerling, Keith G., Kalechstein, Ari D., and Shoptaw, Steven. Predicting adherence to treatment for methamphetamine dependence from neuropsychological and drug use variables. Drug and alcohol dependence 2009. 105 (1-2): 48-55.

Doyle, Suzanne R. and Donovan, Dennis M.. Applying an ensemble classification tree approach to the prediction of completion of a 12-step facilitation intervention with stimulant abusers. Psychology of addictive behaviors: journal of the Society of Psychologists in Addictive Behaviors 2014. 28 (4): 1127-1143.

Ekhtiari, H., Rezapour, T., Sawyer, B., Yeh, H. W., Kuplicki, R., Tarrasch, M., Paulus, M. P., Aupperle, R. Neurocognitive Empowerment for Addiction Treatment (NEAT): study protocol for a randomized controlled trial. 2021. 22 (1): 330-#End Page#.

Elkashef, Ahmed, Rawson, Richard A., Smith, Edwina, Pearce, Valerie, Flammino, Frank, Campbell, Jan, Donovick, Roger, Gorodetzky, Charles, Haning, William, Mawhinney, Joseph, McCann, Michael, Weis, Dennis, Williams, Lorie, Ling, Walter, and Vocci, Frank. The NIDA Methamphetamine Clinical Trials Group: a strategy to increase clinical trials research capacity. Addiction (Abingdon, England) 2007. 102 Suppl 1 (#Issue#): 107-113.

Ellinwood, E. H. and Cohen, S.. Amphetamine abuse. Science (New York, N.Y.) 1971. 171 (3969): 420-421.

Ezard, N., Clifford, B., Dunlop, A., Bruno, R., Carr, A., Liu, Z., Siefried, K. J., Lintzeris, N.. Safety and tolerability of oral lisdexamfetamine in adults with methamphetamine dependence: a phase-2 dose-escalation study. 2021. 11 (5): e044696-#End Page#.

Ezard, Nadine, Dunlop, Adrian, Clifford, Brendan, Bruno, Raimondo, Carr, Andrew, Bissaker, Alexandra, and Lintzeris, Nicholas. Study protocol: a dose-escalating, phase-2 study of oral lisdexamfetamine in adults with methamphetamine dependence. BMC psychiatry 2016. 16 (1): 428-#End Page#.

Ezard, Nadine, Dunlop, Adrian, Hall, Michelle, Ali, Robert, McKetin, Rebecca, Bruno, Raimondo, Phung, Nghi, Carr, Andrew, White, Jason, Clifford, Brendan, Liu, Zhixin, Shanahan, Marian, Dolan, Kate, Baker, Amanda L., and Lintzeris, Nicholas. LiMA: a study protocol for a randomised, double-blind, placebo controlled trial of lisdexamfetamine for the treatment of methamphetamine dependence. BMJ open 2018. 8 (7): e020723-#End Page#.

Fletcher, Jesse B. and Reback, Cathy J.. Antisocial personality disorder predicts methamphetamine treatment outcomes in homeless, substance-dependent men who have sex with men. Journal of substance abuse treatment 2013. 45 (3): 266-272.

Fletcher, Jesse B., Dierst-Davies, Rhodri, and Reback, Cathy J.. Contingency management voucher redemption as an indicator of delayed gratification. Journal of substance abuse treatment 2014. 47 (1): 73-77.

Fletcher, Jesse B., Swendeman, Dallas, and Reback, Cathy J.. Associations Between Major Depressive Episode, Methamphetamine Use Disorder Severity, and Engagement in Sexual Risk-Taking Among Methamphetamine-Using Men Who Have Sex with Men. AIDS and behavior 2018. 22 (5): 1461-1466.

Galanter, M.. Network therapy for substance abuse: A clinical trial. Psychotherapy 1993. 30 (2): 251-258.

Galloway, G. P., Marinelli-Casey, P., Stalcup, J., Lord, R., Christian, D., Cohen, J., Reiber, C., and Vandersloot, D.. Treatment-as-usual in the methamphetamine treatment project. Journal of psychoactive drugs 2000. 32 (2): 165-175.

Garfield, J. B. B., Piercy, H., Arunogiri, S., Lubman, D. I., Campbell, S. C., Sanfilippo, P. G., Gavin, J., Hopwood, M., Kotler, E., George, S., Okedara, G., Piccoli, L. R., Manning, V.. Protocol for the methamphetamine approach-avoidance training (MAAT) trial, a randomised controlled trial of personalised approach bias modification for methamphetamine use disorder. 2021. 22 (1): 21-#End Page#.

Glasner-Edwards, S., Mooney, L. J., Ang, A., Hillhouse, M., and Rawson, R.. Does posttraumatic stress disorder affect post-treatment methamphetamine use?. Journal of dual diagnosis 2013. 9 (2): 123-128.

Gossop, M., Marsden, J., Stewart, D., and Rolfe, A.. Treatment retention and 1 year outcomes for residential programmes in England. Drug and alcohol dependence 1999. 57 (2): 89-98.

Heinzerling, Keith G., McCracken, James T., Swanson, Aimee Noelle, Ray, Lara A., and Shoptaw, Steven J.. COMT Vall58Met, BDNF Val66Met, and OPRM1 Asn40Asp and methamphetamine dependence treatment response: Preliminary investigation. Journal of clinical psychopharmacology 2012. 32 (1): 135-137.

Hellem, Tracy L., Sung, Young Hoon, Shi, Xian Feng, Pett, Marjorie A., Latendresse, Gwen, Morgan, Jubel, Huber, Rebekah S., Kuykendall, Danielle, Lundberg, Kelly J., and Renshaw, Perry F.. Creatine as a Novel Treatment for Depression in Females Using Methamphetamine: A Pilot Study. Journal of dual diagnosis 2015. 11 (3-4): 189-202.

Herrell, J. M., Taylor, J. A., Gallagher, C., and Dawud-Noursi, S.. A multisite study of the effectiveness of methamphetamine treatment: an initiative of the Center for Substance Abuse Treatment. Journal of psychoactive drugs 2000. 32 (2): 143-147.

Huber, A., Lord, R. H., Gulati, V., Marinelli-Casey, P., Rawson, R., and Ling, W.. The CSAT methamphetamine treatment program: research design accommodations for "real world" application. Journal of psychoactive drugs 2000. 32 (2): 149-156.

Juel, A., Kristiansen, C. B., Madsen, N. J., Munk-Jorgensen, P., and Hjorth, P.. Interventions to improve lifestyle and quality-of-life in patients with concurrent mental illness and substance use. Nordic journal of psychiatry 2017. 71 (3): 197-204.

Kleber, H. D., Weiss, R. D., Anton, Jr, George, T. P., Greenfield, S. F., Kosten, T. R., O'Brien, C. P., Rounsaville, B. J., Strain, E. C., Ziedonis, D. M., Hennessy, G., Connery, H. S., McIntyre, J. S., Charles, S. C., Anzia, D. J., Cook, I. A., Finnerty, M. T., Johnson, B. R., Nininger, J. E., Summergrad, P., Woods, S. M., and Yager, J.. Treatment of patients with substance use disorders: Second edition. American Journal of Psychiatry 2006. 163 (8 SUPPL.): 1-81.

Kotajima-Murakami, Hiroko, Takano, Ayumi, Ogai, Yasukazu, Tsukamoto, Shotaro, Murakami, Maki, Funada, Daisuke, Tanibuchi, Yuko, Tachimori, Hisateru, Maruo, Kazushi, Sasaki, Tsuyoshi, Matsumoto, Toshihiko, and Ikeda, Kazutaka. Study of effects of ifenprodil in patients with methamphetamine dependence: Protocol for an exploratory, randomized, double-blind, placebo-controlled trial. Neuropsychopharmacology reports 2019. 39 (2): 90-99.

Ling Murtaugh, Kimberly, Krishnamurti, Tamar, Davis, Alexander L., Reback, Cathy J., and Shoptaw, Steven. Spend today, clean tomorrow: predicting methamphetamine abstinence in a randomized controlled trial. Health psychology : official journal of the Division of Health Psychology, American Psychological Association 2013. 32 (9): 958-966.

Marsden, J.. The long road to pharmacotherapies for stimulant dependence. Addiction (Abingdon, England) 2009. 104 (2): 234-#End Page#.

Matsumoto, T., Imamura, F., Kobayashi, O., Wada, K., Ozaki, S., Takeuchi, Y., Hasegawa, M., Imamura, Y., Taniya, Y., and Adachi, Y.. Evaluation of a relapse-prevention program for methamphetamine-dependent inmates using a self-teaching workbook and group therapy. Psychiatry and clinical neurosciences 2014. 68 (1): 61-69.

Mau, Marjorie K., Asao, Karynna, Efird, Jimmy, Saito, Erin, Ratner, Robert, Hafi, Muhannad, and Seto, Todd. Risk factors associated with methamphetamine use and heart failure among native Hawaiians and other Pacific Island peoples. Vascular health and risk management 2009. 5 (1): 45-52.

McElhiney, Martin C., Rabkin, Judith G., Rabkin, Richard, and Nunes, Edward V.. Provigil (modafinil) plus cognitive behavioral therapy for methamphetamine use in HIV+ gay men: a pilot study. The American journal of drug and alcohol abuse 2009. 35 (1): 34-37.

McGlynn, Elizabeth A., Boynton, Julie, Morton, Sally C., Stecher, Brian M., Hayes, Charles, Vaccaro, Jerome V., and Burnam, M. Audrey. Treatment for the dually diagnosed homeless: Program models and implementation experience: Los Angeles. Alcoholism treatment quarterly 1993. 10 (3-4): 171-186.

McKetin, Rebecca, Dean, Olivia M., Turner, Alyna, Kelly, Peter J., Quinn, Brendan, Lubman, Dan I., Dietze, Paul, Carter, Gregory, Higgs, Peter, Baker, Amanda L., Sinclair, Barbara, Reid, David, Manning, Victoria, Te Pas, Nina, Liang, Wenbin, Thomas, Tamsin, Bathish, Ramez, Kent, Margaret, Raftery, Dayle, Arunogiri, Shalini, Cordaro, Frank, Hill, Harry, and Berk, Michael. A study protocol for the N-ICE trial: A randomised double-blind placebo-controlled study of the safety and efficacy of N-acetyl-cysteine (NAC) as a pharmacotherapy for methamphetamine ("ice") dependence. Trials 2019. 20 (1): 325-#End Page#.

McRae-Clark, Aimee L., Brady, Kathleen T., Hartwell, Karen J., White, Kathleen, and Carter, Rickey E.. Methylphenidate transdermal system in adults with past stimulant misuse: an open-label trial. Journal of attention disorders 2011. 15 (7): 539-544.

Mennick, F.. Preventing methamphetamine abuse in youths. The American journal of nursing 2007. 107 (2): 22-#End Page#.

Mimiaga, Matthew J., Pantalone, David W., Biello, Katie B., Glynn, Tiffany Rose, Santostefano, Christopher M., Olson, Jennifer, Pardee, Dana J., Hughto, Jaclyn M. W., Garcia Valles, Josibel, Carrico, Adam W., Mayer, Kenneth H., and Safren, Steven A.. A randomized controlled efficacy trial of behavioral activation for concurrent stimulant use and sexual risk for HIV acquisition among MSM: project IMPACT study protocol. BMC public health 2018. 18 (1): 914-#End Page#.

Moras, K.. Outcome measurement considerations: pharmacological treatments for substance abuse. NIDA research monograph 1997. 175 (#Issue#): 118-136.

Ngai, C. K. H., Wu, C. S. T., and Suen, L. K. P.. Structured Relapse Prevention Program for Chinese Patients in Hong Kong with Comorbidity of Substance Use and Mental Health Disorders: A Feasibility Study. Journal of psychoactive drugs 2020. 52 (1): 56-65.

Polcin, Douglas L., Korcha, Rachael, Bond, Jason, Galloway, Gantt, and Nayak, Madhabika. Changes in psychiatric symptoms among persons with methamphetamine dependence predicts changes in severity of drug problems but not frequency of use. Substance abuse 2016. 37 (1): 209-214.

Reback, Cathy J. and Shoptaw, Steven. Development of an evidence-based, gay-specific cognitive behavioral therapy intervention for methamphetamine-abusing gay and bisexual men. Addictive behaviors 2014. 39 (8): 1286-1291.

Schultz Fischer, Jessica R.. Treatment of co-morbid methamphetamine substance abuse and borderline personality disorder features using modified Dialectical Behavior Therapy. Dissertation Abstracts International: Section B: The Sciences and Engineering 2008. 68 (12-B): 8411-#End Page#.

Seivewright, N.. Treatment and outcome of drug dependence. Current opinion in psychiatry 1990. 3 (3): 403-407.

Shariatirad, S., Mahjoub, A., Haqiqi, A., Hemami, M. R., Tofighi, B., Ekhtiari, H., and Effatpanah, M.. Buprenorphine added on brief cognitive behavioral therapy for treatment of methamphetamine use disorder. Iranian journal of psychiatry and behavioral sciences 2018. 12 (2): e11529-#End Page#.

Shoptaw, S., Klausner, J. D., Reback, C. J., Tierney, S., Stansell, J., Hare, C. B., Gibson, S., Siever, M., King, W. D., Kao, U., and Dang, J.. A public health response to the methamphetamine epidemic: The implementation of contingency management to treat methamphetamine dependence. BMC public health 2006. 6 (#Issue#): 214-#End Page#.

Stauffer, Christopher S., Moschetto, Jenna M., McKernan, Scott M., Hsiang, Elaine, Borsari, Brian, and Woolley, Joshua D.. Oxytocin-enhanced motivational interviewing group therapy for methamphetamine use disorder in men who have sex with men: study protocol for a randomized controlled trial. Trials 2019. 20 (1): 145-#End Page#.

Stitzer, Maxine L., Petry, Nancy M., and Peirce, Jessica. Motivational incentives research in the National Drug Abuse Treatment Clinical Trials Network. Journal of substance abuse treatment 2010. 38 Suppl 1 (#Issue#): S61-S69.

Swadi, H.. Adolescent substance misuse. Current opinion in psychiatry 1993. 6 (4): 511-515.

Takano, Ayumi, Miyamoto, Yuki, Kawakami, Norito, Matsumoto, Toshihiko, Shinozaki, Tomohiro, and Sugimoto, Takashi. Web-based cognitive behavioral relapse prevention program with tailored feedback for people with methamphetamine and other drug use problems: protocol for a multicenter randomized controlled trial in Japan. BMC psychiatry 2016. 16 (#Issue#): 87-#End Page#.

Tait, Robert J., McKetin, Rebecca, Kay-Lambkin, Frances, Bennett, Kylie, Tam, Ada, Bennett, Anthony, Geddes, Jenny, Garrick, Adam, Christensen, Helen, and Griffiths, Kathleen M.. Breakingtheice: a protocol for a randomised controlled trial of an internet-based intervention addressing amphetamine-type stimulant use. BMC psychiatry 2012. 12 (#Issue#): 67-#End Page#.

Talebizadeh, M., Fathali, Lavasani F., Bastani, P., and Noroozi, A.. Cue exposure therapy for treatment of stimulant (methamphetamine) use disorder: study protocol for a randomized controlled trial. Journal of substance use 2020. #volume# (#Issue#): -#End Page#.

Thomas, McLellan A., Randall, M., Joseph, N., and Alterman, A. I.. Categorizing substance abusers using the ASI: Implications for evaluation and treatment. NIDA Research Monograph Series 1990. #volume# (105): 227-235.

Thompson, C. A.. Pulmonary arterial hypertension seen in methamphetamine abusers. American Journal of Health-System Pharmacy 2008. 65 (12): 1109-#End Page#.

Tompkins-Dobbs, Karen and Schiefelbein, Janis. Emergency department policies and procedures for treatment of patients abusing methamphetamine. Journal of emergency nursing: JEN : official publication of the Emergency Department Nurses Association 2011. 37 (5): 437-443.

Westergaard, Ryan P., Hull, Shawnika J., Merkow, Alana, Stephens, Laura K., Hochstatter, Karli R., Olson-Streed, Heidi K., Baker, Lisa M., and Hess, Timothy M.. Computerized Tailored Interventions to Enhance Prevention and Screening for Hepatitis C Virus Among People Who Inject Drugs: Protocol for a Randomized Pilot Study. JMIR research protocols 2016. 5 (1): e15-#End Page#.

Winhusen, Theresa M., Kropp, Frankie, Theobald, Jeff, and Lewis, Daniel F.. Achieving smoking abstinence is associated with decreased cocaine use in cocaine-dependent patients receiving smoking-cessation treatment. Drug and alcohol dependence 2014. 134 (#Issue#): 391-395.

Yamada, C., Siste, K., Hanafi, E., Ophinni, Y., Beatrice, E., Rafelia, V., Alison, P., Limawan, A., Shinozaki, T., Matsumoto, T., Sakamoto, R.. Relapse prevention group therapy via video-conferencing for substance use disorder: Protocol for a multicentre randomised controlled trial in Indonesia. 2021. 11(9) (no pagination) (#Issue#): -#End Page#.

Yoon, J. H., Weaver, M. T., De La Garza, R., Suchting, R., Nerumalla, C. S., Omar, Y., Brown, G. S., Haliwa, I., and Newton, T. F. Comparison of three measurement models of discounting among individuals with methamphetamine use disorder. American Journal on Addictions 2018. 27 (5): 425-432.

### No (or minority of) participants with methamphetamine use disorder or problematic methamphetamine use (or no data specific to these populations)

Aharonovich, E., Sarvet, A., Stohl, M., DesJarlais, D., Tross, S., Hurst, T., Urbina, A., and Hasin, D.. Reducing non-injection drug use in HIV primary care: A randomized trial of brief motivational interviewing, with and without HealthCall, a technology-based enhancement. Journal of substance abuse treatment 2017. 74 (#Issue#): 71-79.

Aharonovich, Efrat, Stohl, Malka, Cannizzaro, Daniela, and Hasin, Deborah. HealthCall delivered via smartphone to reduce co-occurring drug and alcohol use in HIV-infected adults: A randomized pilot trial. Journal of substance abuse treatment 2017. 83 (#Issue#): 15-26.

Alammehrjerdi, Zahra, Briggs, Nancy E., Biglarian, Akbar, Mokri, Azarakhsh, and Dolan, Kate. A Randomized Controlled Trial of Brief Cognitive Behavioral Therapy for Regular Methamphetamine Use in Methadone Treatment. Journal of psychoactive drugs 2019. 51 (3): 280-289.

Alfonso, J. P., Caracuel, A., Delgado-Pastor, L. C., and Verdejo-Garcia, A.. Combined goal management training and mindfulness meditation improve executive functions and decision-making performance in abstinent polysubstance abusers. Drug and alcohol dependence 2011. 117 (1): 78-81.

Alisauskiene, Renata, Loberg, Else Marie, Gjestad, Rolf, Kroken, Rune A., Jorgensen, Hugo A., and Johnsen, Erik. The influence of substance use on the effectiveness of antipsychotic medication: a prospective, pragmatic study. Nordic journal of psychiatry 2019. 73 (4-5): 281-287.

Alterman, A. I., Koppenhaver, J. M., Mulholland, E., Ladden, L. J., and Baime, M. J.. Pilot trial of effectiveness of mindfulness meditation for substance abuse patients. Journal of substance use 2004. 9 (6): 259-268.

Altice, F. L., Maru, D. S. R., Bruce, R. D., Springer, S. A., and Friedland, G. H.. Superiority of directly administered antiretroviral therapy over self-administered therapy among HIV-infected drug users: A prospective, randomized, controlled trial. Clinical Infectious Diseases 2007. 45 (6): 770-778.

Amaro, H., Black, D. S.. Mindfulness-based intervention effects on substance use and relapse among women in residential treatment: A randomized controlled trial with 8.5-month follow-up period from the moment-by-moment in women's recovery project. 2021. 83(6) (#Issue#): 528-538-#End Page#.

Armstrong, M. A., Gonzales, Osejo, V, Lieberman, L., Carpenter, D. M., Pantoja, P. M., and Escobar, G. J.. Perinatal substance abuse interventoin in obstetric clinics decreases adverse neonatal outcomes. Journal of Perinatology 2003. 23 (1): 3-9.

Azrin, N. H., McMahon, P. T., Donohue, B., Besalel, V. A., Lapinski, K. J., Kogan, E. S., Acierno, R. E., and Galloway, E.. Behavior therapy for drug abuse: a controlled treatment outcome study. Behaviour research and therapy 1994. 32 (8): 857-866.

Back, S. E., Gentilin, S., and Brady, K. T.. Cognitive-behavioral stress management for individuals with substance use disorders: A pilot study. Journal of Nervous and Mental Disease 2007. 195 (8): 662-668.

Baer JS, Garrett SB Beadnell. Brief motivational intervention with homeless adolescents: evaluating effects on substance use and service utilization. Psychology of Addictive Behaviors 2007. 21 (4): 582-#End Page#.

Bahrami, S. and Asghari, F.. A controlled trial of acceptance and commitment therapy for addiction severity in methamphetamine users: Preliminary study. Archives of Psychiatry and Psychotherapy 2017. 19 (2): 49-55.

Baker, A., Boggs, T. G., and Lewin, T. J.. Randomized controlled trial of brief cognitive-behavioural interventions among regular users of amphetamine. Addiction (Abingdon, England) 2001. 96 (9): 1279-1287.

Baker, A., Lewin, T., Reichler, H., Clancy, R., Carr, V., Garrett, R., Sly, K., Devir, H., and Terry, M.. Motivational interviewing among psychiatric in-patients with substance use disorders. Acta psychiatrica Scandinavica 2002. 106 (3): 233-240.

Baker, A., Lewin, T., Reichler, H., Clancy, R., Carr, V., Garrett, R., Sly, K., Devir, H., and Terry, M.. Evaluation of a motivational interview for substance use within psychiatric in-patient services. Addiction (Abingdon, England) 2002. 97 (10): 1329-1337.

Barrowclough, C., Haddock, G., Beardmore, R., Conrod, P., Craig, T., Davies, L., Dunn, G., Lewis, S., Moring, J., Tarrier, N., and Wykes, T.. Evaluating integrated MI and CBT for people with psychosis and substance misuse: Recruitment, retention and sample characteristics of the MIDAS trial. Addictive behaviors 2009. 34 (10): 859-866.

Bell, D. C., Montoya, I. D., Richard, A. J., and Dayton, C. A.. The motivation for drug abuse treatment: Testing cognitive and 12-step theories. American Journal of Drug and Alcohol Abuse 1998. 24 (4): 551-571.

Bennett, J. Adam, Stoops, William W., and Rush, Craig R.. Alternative reinforcer response cost impacts methamphetamine choice in humans. Pharmacology, biochemistry, and behavior 2013. 103 (3): 481-486.

Beutler, L. E., Moleiro, C., Malik, M., Harwood, T. M., Romanelli, R., Gallagher-Thompson, D., and Thompson, L.. A comparison of the Dodo, EST, and ATI factors among comorbid stimulant-dependent, depressed patients. Clinical Psychology and Psychotherapy 2003. 10 (2): 69-85.

Black, David S. and Amaro, Hortensia. Moment-by-Moment in Women's Recovery (MMWR): Mindfulness-based intervention effects on residential substance use disorder treatment retention in a randomized controlled trial. Behaviour research and therapy 2019. 120 (#Issue#): 103437-#End Page#.

Bleiberg, J. L., Devlin, P., Croan, J., and Briscoe, R.. Relationship between treatment length and outcome in a therapeutic community. International Journal of the Addictions 1994. 29 (6): 729-740.

Blondell, R. D., Behrens, T., Smith, S. J., Greene, B. J., and Servoss, T. J.. Peer support during inpatient detoxification and aftercare outcomes. Addictive Disorders and their Treatment 2008. 7 (2): 77-86.

Bogenschutz, M. P., Rice, S. L., Tonigan, J. S., Vogel, H. S., Nowinski, J., Hume, D., and Arenella, P. B.. 12-step facilitation for the dually diagnosed: A randomized clinical trial. Journal of substance abuse treatment 2014. 46 (4): 403-411.

Booth, R. E., Kwiatkowski, C. F., and Stephens, R. C.. Effectiveness of HIV/AIDS interventions on drug use and needle risk behaviors for out-of-treatment injection drug users. Journal of psychoactive drugs 1998. 30 (3): 269-278.

Botha, Ulla A., Coetzee, Marise, Koen, Liezl, and Niehaus, Dana J. H.. An Attempt to Stem the Tide: Exploring the Effect of a 90-Day Transitional Care Intervention on Readmissions to an Acute Male Psychiatric Unit in South Africa. Archives of psychiatric nursing 2018. 32 (3): 384-389.

Bowen, S., Witkiewitz, K., Dillworth, T. M., Chawla, N., Simpson, T. L., Ostafin, B. D., Larimer, M. E., Blume, A. W., Parks, G. A., and Marlatt, G. A.. Mindfulness meditation and substance use in an incarcerated population. Psychology of Addictive Behaviors 2006. 20 (3): 343-347.

Brochu, S., Bergeron, J., Landry, M., Germain, M., and Schneeberger, P.. The impact of treatment on criminalized substance addicts. Journal of addictive diseases 2002. 21 (3): 23-41.

Brooks, A. J. and Penn, P. E.. Comparing treatments for dual diagnosis: Twelve-step and self-management and recovery training. American Journal of Drug and Alcohol Abuse 2003. 29 (2): 359-383.

Brooks, Adam C., Ryder, Deanna, Carise, Deni, and Kirby, Kimberly C.. Feasibility and effectiveness of computer-based therapy in community treatment. Journal of substance abuse treatment 2010. 39 (3): 227-235.

Brown, B. S., O'Grady, K., Battjes, R. J., and Farrell, E. V.. Factors associated with treatment outcomes in an aftercare population. American Journal on Addictions 2004. 13 (5): 447-460.

Brown, E Sherwood, Nejtek, Vicki A., Perantie, Dana C., Rajan Thomas, Nancy, and Rush, A. John. Cocaine and amphetamine use in patients with psychiatric illness: a randomized trial of typical antipsychotic continuation or discontinuation. Journal of clinical psychopharmacology 2003. 23 (4): 384-388.

Brown, T. G., Seraganian, P., Tremblay, J., and Annis, H.. Matching substance abuse aftercare treatments to client characteristics. Addictive behaviors 2002. 27 (4): 585-604.

Brown, T. G., Seraganian, P., Tremblay, J., and Annis, H.. Process and outcome changes with relapse prevention versus 12-step aftercare programs for substance abusers. Addiction (Abingdon, England) 2002. 97 (6): 677-689.

Buchanan, A. L., Vermund, S. H., Friedman, S. R., and Spiegelman, D.. Assessing Individual and Disseminated Effects in Network-Randomized Studies. American journal of epidemiology 2018. 187 (11): 2449-2459.

Burlew, Ann Kathleen, Montgomery, LaTrice, Kosinski, Andrzej S., and Forcehimes, Alyssa A.. Does treatment readiness enhance the response of African American substance users to Motivational Enhancement Therapy?. Psychology of addictive behaviors : journal of the Society of Psychologists in Addictive Behaviors 2013. 27 (3): 744-753.

Burling, T. A., Burling, A. S., and Latini, D.. A controlled smoking cessation trial for substance-dependent inpatients. Journal of consulting and clinical psychology 2001. 69 (2): 295-304.

Burling, T. A., Marshall, G. D., and Seidner, A. L.. Smoking cessation for substance abuse inpatients. Journal of substance abuse 1991. 3 (3): 269-276.

Caldeiro, R. M., Malte, C. A., Calsyn, D. A., Baer, J. S., Nichol, P., Kivlahan, D. R., and Saxon, A. J.. The association of persistent pain with out-patient addiction treatment outcomes and service utilization. Addiction (Abingdon, England) 2008. 103 (12): 1996-2005.

Campbell, B. K., Wander, N., Stark, M. J., and Holbert, T.. Treating cigarette smoking in drug-abusing clients. Journal of substance abuse treatment 1995. 12 (2): 89-94.

Capone, C., Presseau, C., Saunders, E., Eaton, E., Hamblen, J., and McGovern, M.. Is Integrated CBT Effective in Reducing PTSD Symptoms and Substance Use in Iraq and Afghanistan Veterans? Results from a Randomized Clinical Trial. Cognitive Therapy and Research 2018. 42 (6): 735-746.

Carpentier, Pieter J., de Jong, Cor A. J., Dijkstra, Boukje A. G., Verbrugge, Cor A. G., and Krabbe, Paul F. M.. A controlled trial of methylphenidate in adults with attention deficit/hyperactivity disorder and substance use disorders. Addiction (Abingdon, England) 2005. 100 (12): 1868-1874.

Carrico, A. W., Nation, A., Gomez, W., Dilworth, S. E., Johnson, M. O., Moskowitz, J. T., Rose, C. D., and Sundberg, J.. Pilot Trial of an Expressive Writing Intervention with HIV-Positive Methamphetamine-Using Men Who Have Sex with Men. Psychology of Addictive Behaviors 2015. 29 (2): 277-282.

Carrico, Adam W., Gomez, Walter, Jain, Jennifer, Shoptaw, Steven, Discepola, Michael V., Olem, David, Lagana-Jackson, Justin, Andrews, Rick, Neilands, Torsten B., Dilworth, Samantha E., Evans, Jennifer L., Woods, William J., and Moskowitz, Judith T.. Randomized controlled trial of a positive affect intervention for methamphetamine users. Drug and alcohol dependence 2018. 192 (#Issue#): 8-15.

Carrico, Adam W., Nil, Ean, Sophal, Chhit, Stein, Ellen, Sokunny, Muth, Yuthea, Neak, Evans, Jennifer L., Ngak, Song, Maher, Lisa, and Page, Kimberly. Behavioral interventions for Cambodian female entertainment and sex workers who use amphetamine-type stimulants. Journal of behavioral medicine 2016. 39 (3): 502-510.

Carroll, K. M., Ball, S. A., Martino, S., Nich, C., Babuscio, T. A., Nuro, K. F., Gordon, M. A., Portnoy, G. A., and Rounsaville, B. J.. Computer-assisted delivery of cognitive-behavioral therapy for addiction: A randomized trial of CBT4CBT. American Journal of Psychiatry 2008. 165 (7): 881-888.

Carroll, K. M., Ball, S. A., Nich, C., Martino, S., Frankforter, T. L., Farentinos, C., Kunkel, L. E., Mikulich-Gilbertson, S. K., Morgenstern, J., Obert, J. L., Polcin, D., Snead, N., and Woody, G. E.. Motivational interviewing to improve treatment engagement and outcome in individuals seeking treatment for substance abuse: A multisite effectiveness study. Drug and alcohol dependence 2006. 81 (3): 301-312.

Chapple, P. A. and Gray, G.. One year's work at a centre for the treatment of addicted patients. Lancet (London, England) 1968. 1 (7548): 908-911.

Chermack, Stephen T., Bonar, Erin E., Goldstick, Jason E., Winters, Jamie, Blow, Frederic C., Friday, Steven, Ilgen, Mark A., Rauch, Sheila A. M., Perron, Brian E., Ngo, Quyen M., and Walton, Maureen A.. A randomized controlled trial for aggression and substance use involvement among Veterans: Impact of combining Motivational Interviewing, Cognitive Behavioral Treatment and telephone-based Continuing Care. Journal of substance abuse treatment 2019. 98 (#Issue#): 78-88.

Chermack, Stephen T., Bonar, Erin E., Ilgen, Mark A., Walton, Maureen A., Cunningham, Rebecca M., Booth, Brenda M., and Blow, Frederic C.. Developing an Integrated Violence Prevention for Men and Women in Treatment for Substance Use Disorders. Journal of interpersonal violence 2017. 32 (4): 581-603.

Chisolm, M. S., Brigham, E. P., Tuten, M., Strain, E. C., and Jones, H. E.. The relationship between antidepressant use and smoking cessation in pregnant women in treatment for substance abuse. American Journal of Drug and Alcohol Abuse 2010. 36 (1): 46-51.

Choo, Esther K., Zlotnick, Caron, Strong, David R., Squires, Daniel D., Tape, Chantal, and Mello, Michael J.. BSAFER: A Web-based intervention for drug use and intimate partner violence demonstrates feasibility and acceptability among women in the emergency department. Substance abuse 2016. 37 (3): 441-449.

Cochran, Gerald, Stitzer, Maxine, Campbell, Aimee N. C., Hu, Mei Chen, Vandrey, Ryan, and Nunes, Edward V.. Web-based treatment for substance use disorders: differential effects by primary substance. Addictive behaviors 2015. 45 (#Issue#): 191-194.

Cohen, L. R. and Hien, D. A.. Treatment outcomes for women with substance abuse and PTSD who have experienced complex trauma. Psychiatric Services 2006. 57 (1): 100-106.

Corby, N. H. and Wolitski, R. J.. Condom use with main and other sex partners among high-risk women: intervention outcomes and correlates of reduced risk. Drugs & society (New York, N.Y.) 1996. 9 (1-2): 75-96.

Cottler, L. B., Compton, W. M., Ben Abdallah, A., Cunningham-Williams, R., Abram, F., Fichtenbaum, C., and Dotson, W.. Peer-delivered interventions reduce HIV risk behaviors among out-of-treatment drug abusers. Public health reports (Washington, D.C.: 1974) 1998. 113 Suppl 1 (#Issue#): 31-41.

Cox, W. M., Heinemann, A. W., Vincent, Miranti S., Schmidt, M., Klinger, E., and Blount, J.. Outcomes of Systematic Motivational Counseling for substance use following traumatic brain injury. Journal of addictive diseases 2003. 22 (1): 93-110.

Danaee-Far, Morteza, Maarefvand, Masoomeh, and Rafiey, Hassan. Effectiveness of a Brief Home-Based Social Work Motivational Intervention for Male Methamphetamine Users in Tehran: A Randomized Clinical Trial. Substance use & misuse 2016. 51 (14): 1863-1869.

Davey-Rothwell, M. A., Kuramoto, S. J., and Latkin, C. A.. Social networks, norms, and 12-step group participation. American Journal of Drug and Alcohol Abuse 2008. 34 (2): 185-193.

Dawson-Rose, Carol, Draughon, Jessica E., Cuca, Yvette, Zepf, Roland, Huang, Emily, Cooper, Bruce A., and Lum, Paula J.. Changes in Specific Substance Involvement Scores among SBIRT recipients in an HIV primary care setting. Addiction science & clinical practice 2017. 12 (1): 34-#End Page#.

Donovan, D. M., Rosengren, D. B., Downey, L., Cox, G. B., and Sloan, K. L.. Attrition prevention with individuals awaiting publicly funded drug treatment. Addiction (Abingdon, England) 2001. 96 (8): 1149-1160.

Donovan, Dennis M., Daley, Dennis C., Brigham, Gregory S., Hodgkins, Candace C., Perl, Harold I., Garrett, Sharon B., Doyle, Suzanne R., Floyd, Anthony S., Knox, Patricia C., Botero, Christopher, Kelly, Thomas M., Killeen, Therese K., Hayes, Carole, Kau'i Baumhofer, Nicole, Seamans, Cindy, and Zammarelli, Lucy. Stimulant abuser groups to engage in 12-step: a multisite trial in the National Institute on Drug Abuse Clinical Trials Network. Journal of substance abuse treatment 2013. 44 (1): 103-114.

Dore-Gauthier, Virginie, Miron, Jean Philippe, Jutras-Aswad, Dider, Ouellet-Plamondon, Clairelaine, and Abdel-Baki, Amal. Specialized assertive community treatment intervention for homeless youth with first episode psychosis and substance use disorder: A 2-year follow-up study. Early intervention in psychiatry 2020. 14 (2): 203-210.

Drake, R. E., Yovetich, N. A., Bebout, R. R., Harris, M., and McHugo, G. J.. Integrated treatment for dually diagnosed homeless adults. The Journal of nervous and mental disease 1997. 185 (5): 298-305.

Dyba, Janina, Moesgen, Diana, Klein, Michael, Pels, Fabian, and Leyendecker, Birgit. Evaluation of a family-oriented parenting intervention for methamphetamine-involved mothers and fathers - The SHIFT Parent Training. Addictive behaviors reports 2019. 9 (#Issue#): 100173-#End Page#.

Easton, Caroline J., Crane, Cory A., and Mandel, Dolores. A Randomized Controlled Trial Assessing the Efficacy of Cognitive Behavioral Therapy for Substance-Dependent Domestic Violence Offenders: An Integrated Substance Abuse-Domestic Violence Treatment Approach (SADV). Journal of marital and family therapy 2018. 44 (3): 483-498.

Easton, Caroline J., Mandel, Dolores, Babuscio, Theresa, Rounsaville, Bruce J., and Carroll, Kathleen M.. Differences in treatment outcome between male alcohol dependent offenders of domestic violence with and without positive drug screens. Addictive behaviors 2007. 32 (10): 2151-2163.

Eldridge, G. D., St Lawrence, J. S., Little, C. E., Shelby, M. C., Brasfield, T. L., Service, J. W., and Sly, K.. Evaluation of the HIV risk reduction intervention for women entering inpatient substance abuse treatment. AIDS education and prevention : official publication of the International Society for AIDS Education 1997. 9 (1 Suppl): 62-76.

Ersche, Karen D., Bullmore, Edward T., Craig, Kevin J., Shabbir, Shaila S., Abbott, Sanja, Muller, Ulrich, Ooi, Cinly, Suckling, John, Barnes, Anna, Sahakian, Barbara J., Merlo-Pich, Emilio V., and Robbins, Trevor W.. Influence of compulsivity of drug abuse on dopaminergic modulation of attentional bias in stimulant dependence. Archives of general psychiatry 2010. 67 (6): 632-644.

Ersche, Karen D., Roiser, Jonathan P., Abbott, Sanja, Craig, Kevin J., Muller, Ulrich, Suckling, John, Ooi, Cinly, Shabbir, Shaila S., Clark, Luke, Sahakian, Barbara J., Fineberg, Naomi A., Merlo-Pich, Emilio V., Robbins, Trevor W., and Bullmore, Edward T.. Response perseveration in stimulant dependence is associated with striatal dysfunction and can be ameliorated by a D(2/3) receptor agonist. Biological psychiatry 2011. 70 (8): 754-762.

Ersche, Karen D., Roiser, Jonathan P., Lucas, Mark, Domenici, Enrico, Robbins, Trevor W., and Bullmore, Edward T.. Peripheral biomarkers of cognitive response to dopamine receptor agonist treatment. Psychopharmacology 2011. 214 (4): 779-789.

Estrada, Yannine, Lee, Tae Kyoung, Wagstaff, Rachel, Rojas, M., Tapia, Maria I., Velazquez, Maria Rosa, Sardinas, Krystal, Pantin, Hilda, Sutton, Madeline Y., and Prado, Guillermo. eHealth Familias Unidas: Efficacy Trial of an Evidence-Based Intervention Adapted for Use on the Internet with Hispanic Families. Prevention science : the official journal of the Society for Prevention Research 2019. 20 (1): 68-77.

Fals-Stewart, W. and Schafer, J.. The treatment of substance abusers diagnosed with obsessive-compulsive disorder: an outcome study. Journal of substance abuse treatment 1992. 9 (4): 365-370.

Farabee, David, Cousins, Sarah J., Brecht, Mary Lynn, Antonini, Valerie P., Lee, Anne B., Brummer, Julie, Hemberg, Jordana, Karno, Mitchell, and Rawson, Richard A.. A comparison of four telephone-based counseling styles for recovering stimulant users. Psychology of addictive behaviors : journal of the Society of Psychologists in Addictive Behaviors 2013. 27 (1): 223-229.

Farnia, Vahid, Shakeri, Jalal, Tatari, Faezeh, Juibari, Toraj Ahmadi, Yazdchi, Katayoun, Bajoghli, Hafez, Brand, Serge, Abdoli, Nasrin, and Aghaei, Abbas. Randomized controlled trial of aripiprazole versus risperidone for the treatment of amphetamine-induced psychosis. The American journal of drug and alcohol abuse 2014. 40 (1): 10-15.

Ferrari, J. R., Groh, D. R., Rulka, G., Jason, L. A., and Davis, M. I.. Coming to terms with reality: Predictors of self-deception within substance abuse recovery. Addictive Disorders and their Treatment 2008. 7 (4): 210-218.

Fletcher, J. B., Clark, K. A., Reback, C. J.. Depression and HIV Transmission Risk among Methamphetamine-Using Men who have Sex with Men. 2021. 29 (3): 263-270-#End Page#.

Fletcher, T. D., Cunningham, J. L., Calsyn, R. J., Morse, G. A., and Klinkenberg, W. D.. Evaluation of treatment programs for dual disorder individuals: Modeling longitudinal and mediation effects. Administration and Policy in Mental Health and Mental Health Services Research 2008. 35 (4): 319-336.

Galai, Noya, Sirirojn, Bangorn, Aramrattana, Apinun, Srichan, Kamolrawee, Thomson, Nicholas, Golozar, Asieh, Flores, Jose M., Willard, Nancy, Ellen, Jonathan M., Sherman, Susan G., and Celentano, David D.. A cluster randomized trial of community mobilization to reduce methamphetamine use and HIV risk among youth in Thailand: Design, implementation and results. Social science & medicine (1982) 2018. 211 (#Issue#): 216-223.

Gardner, L. I., Marks, G., Shahani, L., Giordano, T. P., Wilson, T. E., Drainoni, M.-L., Keruly, J. C., Batey, D. S., and Metsch, L. R.. Assessing efficacy of a retention-in-care intervention among HIV patients with depression, anxiety, heavy alcohol consumption and illicit drug use. AIDS (London, England) 2016. 30 (7): 1111-1119.

Garrett, Sharon B., Doyle, Suzanne R., Peavy, K. Michelle, Wells, Elizabeth A., Owens, Mandy D., Shores-Wilson, Kathy, DiCenzo, Jessica, and Donovan, Dennis M.. Age differences in outcomes among patients in the "Stimulant Abuser Groups to Engage in 12-Step" (STAGE-12) intervention. Journal of substance abuse treatment 2018. 84 (#Issue#): 21-29.

Geller, B., Cooper, T. B., Watts, H. E., Cosby, C. M., and Fox, L. W.. Early findings from a pharmacokinetically designed double-blind and placebo-controlled study of lithium for adolescents comorbid with biporal and substance dependency disorders. Progress in Neuro-Psychopharmacology and Biological Psychiatry 1992. 16 (3): 281-299.

Ghaleh Emamghaisi, Z., Atashpour, S. H.. Effectiveness of Acceptance and Commitment Therapy on self-criticism and feeling of shame in methamphetamine users. 2020. 25(4) (#Issue#): 372-376-#End Page#.

Glasner-Edwards, Suzette, Mooney, Larissa J., Ang, Alfonso, Garneau, Helene Chokron, Hartwell, Emily, Brecht, Mary Lynn, and Rawson, Richard A.. Mindfulness Based Relapse Prevention for Stimulant Dependent Adults: A Pilot Randomized Clinical Trial. Mindfulness 2017. 8 (1): 126-135.

Gotestam, K. G. and Gunne, L. M.. Subjective effects of two anorexigenic agents fenfluramine and AN 448 in amphetamine dependent subjects. British journal of addiction 1972. 67 (1): 39-44.

Granholm, Eric, Tate, Susan R., Link, Peter C., Lydecker, Katherine P., Cummins, Kevin M., McQuaid, John, Shriver, Chris, and Brown, Sandra A.. Neuropsychological functioning and outcomes of treatment for co-occurring depression and substance use disorders. The American journal of drug and alcohol abuse 2011. 37 (4): 240-249.

Green, Alan I., Tohen, Mauricio F., Hamer, Robert M., Strakowski, Stephen M., Lieberman, Jeffrey A., Glick, Ira, Clark, W. Scott, and HGDH Research Group. First episode schizophrenia-related psychosis and substance use disorders: acute response to olanzapine and haloperidol. Schizophrenia research 2004. 66 (2-3): 125-135.

Greenfield, Shelly F., Trucco, Elisa M., McHugh, R. Kathryn, Lincoln, Melissa, and Gallop, Robert J.. The Women's Recovery Group Study: a Stage I trial of women-focused group therapy for substance use disorders versus mixed-gender group drug counseling. Drug and alcohol dependence 2007. 90 (1): 39-47.

Grelotti, David J., Hammer, Gwendolyn P., Dilley, James W., Karasic, Dan H., Sorensen, James L., Bangsberg, David R., and Tsai, Alexander C.. Does substance use compromise depression treatment in persons with HIV? Findings from a randomized controlled trial. AIDS care 2017. 29 (3): 273-279.

Gryczynski, Jan, Mitchell, Shannon Gwin, Gonzales, Arturo, Moseley, Ana, Peterson, Thomas R., Ondersma, Steven J., O'Grady, Kevin E., and Schwartz, Robert P.. A randomized trial of computerized vs. in-person brief intervention for illicit drug use in primary care: outcomes through 12 months. Journal of substance abuse treatment 2015. 50 (#Issue#): 3-10.

Guterstam, Joar, Jayaram-Lindstrom, Nitya, Berrebi, Jonathan, Petrovic, Predrag, Ingvar, Martin, Fransson, Peter, and Franck, Johan. Cue reactivity and opioid blockade in amphetamine dependence: A randomized, controlled fMRI study. Drug and alcohol dependence 2018. 191 (#Issue#): 91-97.

Hagedorn, Hildi J., Noorbaloochi, Siamak, Simon, Alisha Baines, Bangerter, Ann, Stitzer, Maxine L., Stetler, Cheryl B., and Kivlahan, Daniel. Rewarding early abstinence in Veterans Health Administration addiction clinics. Journal of substance abuse treatment 2013. 45 (1): 109-117.

Hagedorn, Hildi J., Stetler, Cheryl B., Bangerter, Ann, Noorbaloochi, Siamak, Stitzer, Maxine L., and Kivlahan, Daniel. An implementation-focused process evaluation of an incentive intervention effectiveness trial in substance use disorders clinics at two Veterans Health Administration medical centers. Addiction science & clinical practice 2014. 9 (#Issue#): 12-#End Page#.

Hall, Elizabeth A., Prendergast, Michael L., Roll, John M., and Warda, Umme. Reinforcing Abstinence and Treatment Participation among Offenders in a Drug Diversion Program: Are Vouchers Effective?. Criminal justice and behavior 2009. 36 (9): 935-953.

Hammond, A. S., Antoine, D. G., Stitzer, M. L., Strain, E. C.. A Randomized and Controlled Acceptability Trial of an Internet-based Therapy among Inpatients with Co-occurring Substance Use and Other Psychiatric Disorders. 2020. #volume# (#Issue#): 1-8-#End Page#.

Han, Hui, Zhang, Jing Ying, Hser, Yih Ing, Liang, Di, Li, Xu, Wang, Shan Shan, Du, Jiang, and Zhao, Min. Feasibility of a Mobile Phone App to Support Recovery From Addiction in China: Secondary Analysis of a Pilot Study. JMIR mHealth and uHealth 2018. 6 (2): e46-#End Page#.

Hechanova, M. R. M., Reyes, J. C., Acosta, A. C., Tuliao, A. P.. Psychosocial treatment for incarcerated methamphetamine users: the Philippines experience. 2020. 16 (4): 343-358-#End Page#.

Hellerstein, D. J., Rosenthal, R. N., and Miner, C. R.. A prospective study of integrated outpatient treatment for substance-abusing schizophrenic patients. American Journal on Addictions 1995. 4 (1): 33-42.

Henggeler, S. W., Clingempeel, W. G., Brondino, M. J., and Pickrel, S. G.. Four-Year Follow-up of Multisystemic Therapy with Substance-Abusing and Substance-Dependent Juvenile Offenders. Journal of the American Academy of Child and Adolescent Psychiatry 2002. 41 (7): 868-874.

Hershberger, Scott L., Wood, Michele M., and Fisher, Dennis G.. A cognitive-behavioral intervention to reduce HIV risk behaviors in crack and injection drug users. AIDS and behavior 2003. 7 (3): 229-243.

Hien, Denise A., Morgan-Lopez, Antonio A., Campbell, Aimee N. C., Saavedra, Lissette M., Wu, Elwin, Cohen, Lisa, Ruglass, Lesia, and Nunes, Edward V.. Attendance and substance use outcomes for the Seeking Safety program: sometimes less is more. Journal of consulting and clinical psychology 2012. 80 (1): 29-42.

Hoffman, J. A., Klein, H., Crosby, H., and Clark, D. C.. Project neighborhoods in action: an HIV-related intervention project targeting drug abusers in Washington, DC. Journal of urban health : bulletin of the New York Academy of Medicine 1999. 76 (4): 419-434.

Horvath, Keith J., Lammert, Sara, MacLehose, Richard F., Danh, Thu, Baker, Jason V., and Carrico, Adam W.. A Pilot Study of a Mobile App to Support HIV Antiretroviral Therapy Adherence Among Men Who Have Sex with Men Who Use Stimulants. AIDS and behavior 2019. 23 (11): 3184-3198.

Huang, Ya Shune, Tang, Tze Chun, Lin, Chi Hung, and Yen, Cheng Fang. Effects of motivational enhancement therapy on readiness to change MDMA and methamphetamine use behaviors in Taiwanese adolescents. Substance use & misuse 2011. 46 (4): 411-416.

Humeniuk, Rachel, Ali, Robert, Babor, Thomas, Souza-Formigoni, Maria Lucia, de Lacerda, Roseli Boerngen, Ling, Walter, McRee, Bonnie, Newcombe, David, Pal, Hemraj, Poznyak, Vladimir, Simon, Sara, and Vendetti, Janice. A randomized controlled trial of a brief intervention for illicit drugs linked to the Alcohol, Smoking and Substance Involvement Screening Test (ASSIST) in clients recruited from primary health-care settings in four countries. Addiction (Abingdon, England) 2012. 107 (5): 957-966.

Iguchi, M. Y., Stitzer, M. L., Bigelow, G. E., and Liebson, I. A.. Contingency management in methadone maintenance: effects of reinforcing and aversive consequences on illicit polydrug use. Drug and alcohol dependence 1988. 22 (1-2): 1-7.

Jalali, Farzad and Hashemi, Seyedeh Fatemeh. The Effect of Saffron on Depression among Recovered Consumers of Methamphetamine Living with HIV/AIDS. Substance use & misuse 2018. 53 (12): 1951-1957.

James, W., Preston, N. J., Koh, G., Spencer, C., Kisely, S. R., and Castle, D. J.. A group intervention which assists patients with dual diagnosis reduce their drug use: A randomized controlled trial. Psychological medicine 2004. 34 (6): 983-990.

Jayaram-Lindstrom, Nitya, Hammarberg, Anders, Beck, Olof, and Franck, Johan. Naltrexone for the treatment of amphetamine dependence: a randomized, placebo-controlled trial. The American journal of psychiatry 2008. 165 (11): 1442-1448.

Jayaram-Lindstrom, Nitya, Konstenius, Maija, Eksborg, Staffan, Beck, Olof, Hammarberg, Anders, and Franck, Johan. Naltrexone attenuates the subjective effects of amphetamine in patients with amphetamine dependence. Neuropsychopharmacology : official publication of the American College of Neuropsychopharmacology 2008. 33 (8): 1856-1863.

Jittiwutikan, J., Srisurapanont, M., and Jarusuraisin, N.. Amineptine in the treatment of amphetamine withdrawal: a placebo-controlled, randomised, double-blind study. Journal of the Medical Association of Thailand = Chotmaihet thangphaet 1997. 80 (9): 587-592.

Joe, G. W., Simpson, D. D., Dansereau, D. F., and Rowan-Szal, G. A.. Relationships between counseling rapport and drug abuse treatment outcomes. Psychiatric services (Washington, D.C.) 2001. 52 (9): 1223-1229.

Johnson, J. E. and Zlotnick, C.. Pilot study of treatment for major depression among women prisoners with substance use disorder. Journal of psychiatric research 2012. 46 (9): 1174-1183.

Joseph, A. M., Nichol, K. L., and Anderson, H.. Effect of treatment for nicotine dependence on alcohol and drug treatment outcomes. Addictive behaviors 1993. 18 (6): 635-644.

Kamara, S. G. and Van der Hyde, V. A.. Outcomes of regular vs. extended alcohol/drug outpatient treatment: I. Relapse, aftercare, and treatment re-entry. Medicine and law 1997. 16 (3): 607-620.

Karno, Mitchell, Farabee, David, Brecht, Mary Lynn, and Rawson, Richard. Patient reactance moderates the effect of directive telephone counseling for methamphetamine users. Journal of studies on alcohol and drugs 2012. 73 (5): 844-850.

Kashner, T. M., Rosenheck, R., Campinell, A. B., Suris, A., Crandall, R., Garfield, N. J., Lapuc, P., Pyrcz, K., Soyka, T., and Wicker, A.. Impact of work therapy on health status among homeless, substance-dependent veterans: A randomized controlled trial. Archives of general psychiatry 2002. 59 (10): 938-944.

Kay-Lambkin, Frances J., Baker, Amanda L., McKetin, Rebecca, and Lee, Nicole. Stepping through treatment: reflections on an adaptive treatment strategy among methamphetamine users with depression. Drug and alcohol review 2010. 29 (5): 475-482.

Kehle-Forbes, S. M., Drapkin, M. L., Foa, E. B., Koffel, E., Lynch, K. G., Polusny, M. A., Van Horn, D. H. A., Yusko, D. A., Charlesworth, M., Blasco, M., and Oslin, D. W.. Study design, interventions, and baseline characteristics for the Substance use and TRauma Intervention for VEterans (STRIVE) trial. Contemporary clinical trials 2016. 50 (#Issue#): 45-53.

Killeen, Therese, Carter, Rickey, Copersino, Marc, Petry, Nancy, and Stitzer, Maxine. Effectiveness of motivational incentives in stimulant abusing outpatients with different treatment histories. The American journal of drug and alcohol abuse 2007. 33 (1): 129-137.

Kiluk, B. D., Nich, C., Babuscio, T., and Carroll, K. M.. Quality versus quantity: acquisition of coping skills following computerized cognitive-behavioral therapy for substance use disorders. Addiction (Abingdon, England) 2010. 105 (12): 2120-2127.

Kim, Theresa W., Bernstein, Judith, Cheng, Debbie M., Lloyd-Travaglini, Christine, Samet, Jeffrey H., Palfai, Tibor P., and Saitz, Richard. Receipt of addiction treatment as a consequence of a brief intervention for drug use in primary care: a randomized trial. Addiction (Abingdon, England) 2017. 112 (5): 818-827.

Kirby, K. C., Benishek, L. A., Kerwin, M. E., Dugosh, K. L., Carpenedo, C. M., Bresani, E., Haugh, J. A., Washio, Y., and Meyers, R. J.. Analyzing components of Community Reinforcement and Family Training (CRAFT): Is treatment entry training sufficient?. Psychology of Addictive Behaviors 2017. 31 (7): 818-827.

Koblan, K. S., Hopkins, S. C., Sarma, K., Gallina, N., Jin, F., Levy-Cooperman, N., Schoedel, K. A., and Loebel, A.. Assessment of human abuse potential of dasotraline compared to methylphenidate and placebo in recreational stimulant users. Drug and alcohol dependence 2016. 159 (#Issue#): 26-34.

Kongsakon, Ronnachai, Papadopoulos, Konstantinos I., and Saguansiritham, Rapeepun. Mirtazapine in amphetamine detoxification: a placebo-controlled pilot study. International clinical psychopharmacology 2005. 20 (5): 253-256.

Konstenius, Maija, Jayaram-Lindstrom, Nitya, Beck, Olof, and Franck, Johan. Sustained release methylphenidate for the treatment of ADHD in amphetamine abusers: a pilot study. Drug and alcohol dependence 2010. 108 (1-2): 130-133.

Konstenius, Maija, Jayaram-Lindstrom, Nitya, Guterstam, Joar, Beck, Olof, Philips, Bjorn, and Franck, Johan. Methylphenidate for attention deficit hyperactivity disorder and drug relapse in criminal offenders with substance dependence: a 24-week randomized placebo-controlled trial. Addiction (Abingdon, England) 2014. 109 (3): 440-449.

Kraanen, F. L., Vedel, E., Scholing, A., and Emmelkamp, P. M. G.. The comparative effectiveness of Integrated treatment for Substance abuse and Partner violence (I-StoP) and substance abuse treatment alone: A randomized controlled trial. BMC psychiatry 2013. 13 (#Issue#): 189-#End Page#.

Kropp, F., Winhusen, T., Lewis, D., Hague, D., and Somoza, E.. Increasing prenatal care and healthy behaviors in pregnant substance users. Journal of psychoactive drugs 2010. 42 (1): 73-81.

Kurtz, S. P., Buttram, M. E., Pagano, M. E., and Surratt, H. L.. A randomized trial of brief assessment interventions for young adults who use drugs in the club scene. Journal of substance abuse treatment 2017. 78 (#Issue#): 64-73.

Landovitz, R. J., Fletcher, J. B., Shoptaw, S., and Reback, C. J.. Contingency management facilitates the use of postexposure prophylaxis among stimulant-using men who have sex with men. Open Forum Infectious Diseases 2015. 2 (1): ofu114-#End Page#.

Lash, S. J.. Increasing participation in substance abuse aftercare treatment. American Journal of Drug and Alcohol Abuse 1998. 24 (1): 31-36.

Lash, S. J., Burden, J. L., Parker, J. D., Stephens, R. S., Budney, A. J., Horner, R. D., Datta, S., Jeffreys, A. S., and Grambow, S. C.. Contracting, prompting and reinforcing substance use disorder continuing care. Journal of substance abuse treatment 2013. 44 (4): 449-456.

Ledgerwood, David M. and Petry, Nancy M.. Does contingency management affect motivation to change substance use?. Drug and alcohol dependence 2006. 83 (1): 65-72.

Li, L., Hien, N. T., Lin, C., Tuan, N. A., Tuan, L. A., Farmer, S. C., and Detels, R.. An intervention to improve mental health and family well-being of injecting drug users and family members in Vietnam. Psychology of Addictive Behaviors 2014. 28 (2): 607-613.

Lile, Joshua A., Stoops, William W., Vansickel, Andrea R., Glaser, Paul E. A., Hays, Lon R., and Rush, Craig R.. Aripiprazole attenuates the discriminative-stimulus and subject-rated effects of D-amphetamine in humans. Neuropsychopharmacology : official publication of the American College of Neuropsychopharmacology 2005. 30 (11): 2103-2114.

Little, Bertis B., Snell, Laura M., Van Beveren, Toosje T., Crowell, R. Becca, Trayler, Stacey, and Johnston, Walter L.. Treatment of substance abuse during pregnancy and infant outcome. American journal of perinatology 2003. 20 (5): 255-262.

Lott, David C. and Jencius, Simon. Effectiveness of very low-cost contingency management in a community adolescent treatment program. Drug and alcohol dependence 2009. 102 (1-3): 162-165.

Luderer, H. F., Campbell, A. N. C., Nunes, E. V., Enman, N. M., Xiong, X., Gerwien, R., Maricich, Y. A.. Engagement patterns with a digital therapeutic for substance use disorders: Correlations with abstinence outcomes. 2022. 132 (#Issue#): 108585-#End Page#.

Luoma, J. B., Kohlenberg, B. S., Hayes, S. C., and Fletcher, L.. Slow and steady wins the race: A randomized clinical trial of acceptance and commitment therapy targeting shame in substance use disorders. Journal of consulting and clinical psychology 2012. 80 (1): 43-53.

Luthar, Suniya S., Suchman, Nancy E., and Altomare, Michelle. Relational Psychotherapy Mothers' Group: a randomized clinical trial for substance abusing mothers. Development and psychopathology 2007. 19 (1): 243-261.

Lydecker, K. P., Tate, S. R., Cummins, K. M., McQuaid, J., Granholm, E., and Brown, S. A.. Clinical Outcomes of an Integrated Treatment for Depression and Substance Use Disorders. Psychology of Addictive Behaviors 2010. 24 (3): 453-465.

Machado, M. P. A., Fidalgo, T. M., Brasiliano, S., Hochgraf, P. B., Noto, A. R.. The contribution of mindfulness to outpatient substance use disorder treatment in Brazil: A preliminary study. 2020. 42(5) (#Issue#): 527-531-#End Page#.

Malow, R. M., West, J. A., Corrigan, S. A., Pena, J. M., and Cunningham, S. C.. Outcome of psychoeducation for HIV risk reduction. AIDS education and prevention : official publication of the International Society for AIDS Education 1994. 6 (2): 113-125.

Mamey, M. R., Burns, G. L., Barbosa-Leiker, C., Smith, C. L., McPherson, S.. Parallel growth modeling to better understand smoking with stimulant use outcomes during an integrated treatment trial. 2022. 30 (1): 51-58-#End Page#.

Marcus, M. T., Schmitz, J., Moeller, G., Liehr, P., Cron, S. G., Swank, P., Bankston, S., Carroll, D. D., and Granmayeh, L. K.. Mindfulness-based stress reduction in therapeutic community treatment: A stage 1 trial. American Journal of Drug and Alcohol Abuse 2009. 35 (2): 103-108.

Margolin, A., Schuman-Olivier, Z., Beitel, M., Arnold, R. M., Fulwiler, C. E., and Avants, S. K.. A preliminary study of spiritual self-schema (3-S+) therapy for reducing impulsivity in HIV positive drug users. Journal of clinical psychology 2007. 63 (10): 979-999.

Marks, Katherine R., Lile, Joshua A., Stoops, William W., Glaser, Paul E. A., Hays, Lon R., and Rush, Craig R.. Separate and Combined Effects of Naltrexone and Extended-Release Alprazolam on the Reinforcing, Subject-Rated, and Cardiovascular Effects of Methamphetamine. Journal of clinical psychopharmacology 2016. 36 (3): 213-221.

Martin, M., Vanichseni, S., Suntharasamai, P., Sangkum, U., Mock, P. A., Leethochawalit, M., Chiamwongpaet, S., Curlin, M. E., Na-Pompet, S., Warapronmongkholkul, A., Kittimunkong, S., Gvetadze, R. J., McNicholl, J. M., Paxton, L. A., Choopanya, K., Na Ayudhya, S. S., Kaewnil, K., Kitisin, P., Kukavejworakit, M., Natrujirote, P., Simakajorn, S., and Subhachaturas, W.. The impact of adherence to preexposure prophylaxis on the risk of HIV infection among people who inject drugs. AIDS (London, England) 2015. 29 (7): 819-824.

Martin, Michael, Vanichseni, Suphak, Suntharasamai, Pravan, Sangkum, Udomsak, Mock, Philip A., Leethochawalit, Manoj, Chiamwongpaet, Sithisat, Gvetadze, Roman J., Kittimunkong, Somyot, Curlin, Marcel E., Worrajittanon, Dararat, McNicholl, Janet M., Paxton, Lynn A., Choopanya, Kachit, and Bangkok Tenofovir Study Group. Risk behaviors and risk factors for HIV infection among participants in the Bangkok tenofovir study, an HIV pre-exposure prophylaxis trial among people who inject drugs. PloS one 2014. 9 (3): e92809-#End Page#.

Mausbach, Brent T., Semple, Shirley J., Strathdee, Steffanie A., Zians, Jim, and Patterson, Thomas L.. Efficacy of a behavioral intervention for increasing safer sex behaviors in HIV-negative, heterosexual methamphetamine users: results from the Fast-Lane Study. Annals of behavioral medicine : a publication of the Society of Behavioral Medicine 2007. 34 (3): 263-274.

Mausbach, Brent T., Semple, Shirley J., Strathdee, Steffanie A., Zians, Jim, and Patterson, Thomas L.. Efficacy of a behavioral intervention for increasing safer sex behaviors in HIV-positive MSM methamphetamine users: results from the EDGE study. Drug and alcohol dependence 2007. 87 (2-3): 249-257.

McDonell, Michael G., Srebnik, Debra, Angelo, Frank, McPherson, Sterling, Lowe, Jessica M., Sugar, Andrea, Short, Robert A., Roll, John M., and Ries, Richard K.. Randomized controlled trial of contingency management for stimulant use in community mental health patients with serious mental illness. The American journal of psychiatry 2013. 170 (1): 94-101.

McDonell, Michael, McPherson, Sterling, Vilardaga, Roger, Srebnik, Debra, Angelo, Frank N., Leickly, Emily, Saxon, Andrew J., Roll, John, and Ries, Richard. Preliminary findings: Contingency management targeting psycho-stimulant use results in secondary decreases in smoking for severely mentally ill adults. The American journal on addictions 2014. 23 (4): 407-410.

McHugo, G. J., Drake, R. E., Teague, G. B., and Xie, H.. Fidelity to assertive community treatment and client outcomes in the New Hampshire dual disorders study. Psychiatric services (Washington, D.C.) 1999. 50 (6): 818-824.

McLellan, A. T., Grissom, G. R., Zanis, D., Randall, M., Brill, P., and O'Brien, C. P.. Problem-service 'matching' in addiction treatment: A prospective study in 4 programs. Archives of general psychiatry 1997. 54 (8): 730-735.

Melnick, G., De, Leon G., Thomas, G., and Kressel, D.. A client-treatment matching protocol for therapeutic communities: First report. Journal of substance abuse treatment 2001. 21 (3): 119-128.

Menza, Timothy W., Jameson, Damon R., Hughes, James P., Colfax, Grant N., Shoptaw, Steven, and Golden, Matthew R.. Contingency management to reduce methamphetamine use and sexual risk among men who have sex with men: a randomized controlled trial. BMC public health 2010. 10 (#Issue#): 774-#End Page#.

Mertens JR, Ward CL Bresick GF Broder. Effectiveness of nurse-practitioner-delivered brief motivational intervention for young adult alcohol and drug use in primary care in South Africa: a randomized clinical trial. Alcohol and alcoholism (Oxford, Oxfordshire) 2014. 49 (4): 430-#End Page#.

Messina, N. P., Wish, E. D., and Nemes, S.. Therapeutic community treatment for substance abusers with antisocial personality disorder. Journal of substance abuse treatment 1999. 17 (1-2): 121-128.

Messina, N., Nemes, S., Wish, E., and Wraight, B.. Opening the black box. The impact of inpatient treatment services on client outcomes. Journal of substance abuse treatment 2001. 20 (2): 177-183.

Metsch, L. R., Feaster, D. J., Gooden, L., Matheson, T., Stitzer, M., Das, M., Jain, M. K., Rodriguez, A. E., Armstrong, W. S., Lucas, G. M., Nijhawan, A. E., Drainoni, M.-L., Herrera, P., Vergara-Rodriguez, P., Jacobson, J. M., Mugavero, M. J., Sullivan, M., Daar, E. S., McMahon, D. K., Ferris, D. C., Lindblad, R., Van, Veldhuisen P., Oden, N., Castellon, P. C., Tross, S., Haynes, L. F., Douaihy, A., Sorensen, J. L., Metzger, D. S., Mandler, R. N., Colfax, G. N., and Del, Rio C.. Effect of Patient navigation with or without financial incentives on viral suppression among hospitalized patients with HIV infection and substance use a randomized clinical trial. JAMA - Journal of the American Medical Association 2016. 316 (2): 156-170.

Miles, S Wayne, Sheridan, Janie, Russell, Bruce, Kydd, Rob, Wheeler, Amanda, Walters, Carina, Gamble, Greg, Hardley, Peta, Jensen, Maree, Kuoppasalmi, Kimmo, Tuomola, Pekka, Fohr, Jaana, Kuikanmaki, Outi, Vorma, Helena, Salokangas, Raimo, Mikkonen, Antti, Kallio, Mika, Kauhanen, Jussi, Kiviniemi, Vesa, and Tiihonen, Jari. Extended-release methylphenidate for treatment of amphetamine/methamphetamine dependence: a randomized, double-blind, placebo-controlled trial. Addiction (Abingdon, England) 2013. 108 (7): 1279-1286.

Miller, N. S. and Hoffmann, N. G.. Addictions treatment outcomes. Alcoholism treatment quarterly 1995. 12 (2): 41-55.

Moore, T. M., Seavey, A., Ritter, K., McNulty, J. K., Gordon, K. C., and Stuart, G. L.. Ecological momentary assessment of the effects of craving and affect on risk for relapse during substance abuse treatment. Psychology of Addictive Behaviors 2014. 28 (2): 619-624.

Morabbi, Mohammad Javad, Razaghi, Emran, Moazen-Zadeh, Ehsan, Safi-Aghdam, Hamideh, Zarrindast, Mohamad R., Vousoghi, Nasim, and Akhondzadeh, Shahin. Pexacerfont as a CRF1 antagonist for the treatment of withdrawal symptoms in men with heroin/methamphetamine dependence: a randomized, double-blind, placebo-controlled clinical trial. International clinical psychopharmacology 2018. 33 (2): 111-119.

Moreno, A., Perez-Elias, M. J., Casado, J. L., Munoz, V., Antela, A., Dronda, F., Navas, E., and Moreno, S.. Long-term outcomes of protease inhibitor-based therapy in antiretroviral treatment-naive HIV-infected injection drug users on methadone maintenance programmes. AIDS (London, England) 2001. 15 (8): 1068-1070.

Morgan, T. J., Morgenstern, J., Blanchard, K. A., Labouvie, E., and Bux, D. A.. Health-related quality of life for adults participating in outpatient substance abuse treatment. American Journal on Addictions 2003. 12 (3): 198-210.

Mueser, K. T., Glynn, S. M., Cather, C., Zarate, R., Fox, L., Feldman, J., Wolfe, R., and Clark, R. E.. Family intervention for co-occurring substance use and severe psychiatric disorders: Participant characteristics and correlates of initial engagement and more extended exposure in a randomized controlled trial. Addictive behaviors 2009. 34 (10): 867-877.

Murphy, C. M., Martin, R. A., Tidey, J. W., Colby, S. M., and Rohsenow, D. J.. Smoking outcome expectancies predict smoking during voucher-based treatment for smokers with substance use disorders. Journal of substance abuse treatment 2018. 90 (#Issue#): 73-78.

Murphy, S. M., Campbell, A. N. C., Ghitza, U. E., Kyle, T. L., Bailey, G. L., Nunes, E. V., and Polsky, D.. Cost-effectiveness of an internet-delivered treatment for substance abuse: Data from a multisite randomized controlled trial. Drug and alcohol dependence 2016. 161 (#Issue#): 119-126.

Murphy, Sean M., McDonell, Michael G., McPherson, Sterling, Srebnik, Debra, Angelo, Frank, Roll, John M., and Ries, Richard K.. An economic evaluation of a contingency-management intervention for stimulant use among community mental health patients with serious mental illness. Drug and alcohol dependence 2015. 153 (#Issue#): 293-299.

Najavits, L. M., Enggasser, J., Brief, D., and Federman, E.. A randomized controlled trial of a gender-focused addiction model versus 12-step facilitation for women veterans. American Journal on Addictions 2018. 27 (3): 210-216.

Nejtek, Vicki A.. Do atypical antipsychotics effectively treat co-occurring bipolar disorder and stimulant dependence? A randomized, double-blind trial: religious affiliation and psychiatric morbidity in Brazil: higher rates among evangelicals and spiritists. International journal of social psychiatry 2008. 54 (#Issue#): 562-#End Page#.

Nejtek, Vicki A., Avila, Matthew, Chen, Li Ann, Zielinski, Tanya, Djokovic, Marija, Podawiltz, Alan, Kaiser, Kathryn, Bae, Sejong, and Rush, A. John. Do atypical antipsychotics effectively treat co-occurring bipolar disorder and stimulant dependence? A randomized, double-blind trial. The Journal of clinical psychiatry 2008. 69 (8): 1257-1266.

Nicolosi, A., Leite, M. L. C., Molinari, S., Musicco, M., Saracco, A., and Lazzarin, A.. Incidence and prevalence trends of HIV infection in intravenous drug users attending treatment centers in Milan and Northern Italy, 1986-1990. Journal of acquired immune deficiency syndromes 1992. 5 (4): 365-373.

Nikoo, Mohammadali, Vogel, Marc, Choi, Fiona, Song, Michael J., Burghardt, Jensen, Zafari, Zafar, Tabi, Katarina, Frank, Anastasia, Barbic, Skye, Schutz, Christian, Jang, Kerry, and Krausz, Michael. Employment and paid work among participants in a randomized controlled trial comparing diacetylmorphine and hydromorphone. International Journal of Drug Policy 2018. 57 (#Issue#): 18-24.

Nyamathi, A., Reback, C. J., Shoptaw, S., Salem, B. E., Zhang, S., and Yadav, K.. Impact of Tailored Interventions to Reduce Drug Use and Sexual Risk Behaviors Among Homeless Gay and Bisexual Men. Am J Mens Health 2017. 11 (2): 208-220.

Nyamathi, Adeline M., Salem, Benissa E., Farabee, David, and Zhang, Sheldon. Differential reporting of drug use among gay, bisexual and transgender stimulant-using homeless adults post intervention. Journal of substance use 2017. 22 (2): 218-224.

O'Farrell, T. J., Murphy, M., Alter, J., and Fals-Stewart, W.. Behavioral family counseling for substance abuse: A treatment development pilot study. Addictive behaviors 2010. 35 (1): 1-6.

Olmstead, Todd A., Sindelar, Jody L., and Petry, Nancy M.. Cost-effectiveness of prize-based incentives for stimulant abusers in outpatient psychosocial treatment programs. Drug and alcohol dependence 2007. 87 (2-3): 175-182.

Olmstead, Todd A., Yonkers, Kimberly A., Ondersma, Steven J., Forray, Ariadna, Gilstad-Hayden, Kathryn, and Martino, Steve. Cost-effectiveness of electronic- and clinician-delivered screening, brief intervention and referral to treatment for women in reproductive health centers. Addiction (Abingdon, England) 2019. 114 (9): 1659-1669.

Page, Kimberly, Carrico, Adam W., Stein, Ellen, Evans, Jennifer, Sokunny, Muth, Maly, Phou, Sophal, Chhit, Neak, Yuthea, Ngak, Song, McCulloch, Charles, and Maher, Lisa. Cluster randomized stepped-wedge trial of a multi-level HIV prevention intervention to decrease amphetamine-type stimulants and sexual risk in Cambodian female entertainment and sex workers. Drug and alcohol dependence 2019. 196 (#Issue#): 21-30.

Parasrampuria, Dolly A., Schoedel, Kerri A., Schuller, Reinhard, Silber, Steven A., Ciccone, Patrick E., Gu, Joan, and Sellers, Edward M.. Do formulation differences alter abuse liability of methylphenidate? A placebo-controlled, randomized, double-blind, crossover study in recreational drug users. Journal of clinical psychopharmacology 2007. 27 (5): 459-467.

Parsons JT, Lelutiu Weinberger. A randomized controlled trial utilizing motivational interviewing to reduce HIV risk and drug use in young gay and bisexual men. Journal of consulting and clinical psychology 2014. 82 (1): 9-#End Page#.

Parsons, Jeffrey T., John, Steven A., Millar, Brett M., and Starks, Tyrel J.. Testing the Efficacy of Combined Motivational Interviewing and Cognitive Behavioral Skills Training to Reduce Methamphetamine Use and Improve HIV Medication Adherence Among HIV-Positive Gay and Bisexual Men. AIDS and behavior 2018. 22 (8): 2674-2686.

Peirce, Jessica M., Petry, Nancy M., Stitzer, Maxine L., Blaine, Jack, Kellogg, Scott, Satterfield, Frank, Schwartz, Marion, Krasnansky, Joe, Pencer, Eileen, Silva-Vazquez, Lolita, Kirby, Kimberly C., Royer-Malvestuto, Charlotte, Roll, John M., Cohen, Allan, Copersino, Marc L., Kolodner, Ken, and Li, Rui. Effects of lower-cost incentives on stimulant abstinence in methadone maintenance treatment: a National Drug Abuse Treatment Clinical Trials Network study. Archives of general psychiatry 2006. 63 (2): 201-208.

Petry, N. M., Weinstock, J., and Alessi, S. M.. A randomized trial of contingency management delivered in the context of group counseling. Journal of consulting and clinical psychology 2011. 79 (5): 686-696.

Petry, Nancy M., Alessi, Sheila M., Carroll, Kathleen M., Hanson, Tressa, MacKinnon, Stephen, Rounsaville, Bruce, and Sierra, Sean. Contingency management treatments: Reinforcing abstinence versus adherence with goal-related activities. Journal of consulting and clinical psychology 2006. 74 (3): 592-601.

Petry, Nancy M., Kolodner, Ken B., Li, Rui, Peirce, Jessica M., Roll, John M., Stitzer, Maxine L., and Hamilton, John A.. Prize-based contingency management does not increase gambling. Drug and alcohol dependence 2006. 83 (3): 269-273.

Piasecki, Melissa P., Antonuccio, David O., Steinagel, Gerri M., Kohlenberg, Barbara S., and Kapadar, Karen. Penetrating the blind in a study of an SSRI. Journal of behavior therapy and experimental psychiatry 2002. 33 (2): 67-71.

Piasecki, Melissa P., Steinagel, Gerri M., Thienhaus, Ole J., and Kohlenberg, Barbara S.. An exploratory study: the use of paroxetine for methamphetamine craving. Journal of psychoactive drugs 2002. 34 (3): 301-304.

Pitpitan, E. V., MacKinnon, D. P., Eaton, L. A., Smith, L. R., Wagman, J., Patterson, T. L.. Using Novel Approaches to Evaluate Behavioral Interventions: Overlooked Significant HIV Prevention Effects in the HPTN 015 Project EXPLORE. 2021. 87 (5): 1128-1135-#End Page#.

Pitre, U., Dansereau, D. F., Newbern, D., and Simpson, D. D.. Residential drug abuse treatment for probationers: Use of node-link mapping to enhance participation and progress. Journal of substance abuse treatment 1998. 15 (6): 535-543.

Poblete, Fernando, Barticevic, Nicolas A., Zuzulich, Maria Soledad, Portilla, Rodrigo, Castillo-Carniglia, Alvaro, Sapag, Jaime C., Villarroel, Luis, Sena, Brena F., and Galarce, Magdalena. A randomized controlled trial of a brief intervention for alcohol and drugs linked to the Alcohol, Smoking and Substance Involvement Screening Test (ASSIST) in primary health care in Chile. Addiction (Abingdon, England) 2017. 112 (8): 1462-1469.

Polak, K., Meyer, B. L., Neale, Z. E., Reisweber, J.. Program Evaluation of Group Transcending Self Therapy: An Integrative Modular Cognitive-Behavioral Therapy for Substance Use Disorders. 2020. 14 (no pagination): -#End Page#.

Pollastri, A. R., Pokrywa, M. L., Walsh, S. J., Kranzler, H. R., and Gelernter, J.. Incentive program decreases no-shows in nontreatment substance abuse research. Experimental and clinical psychopharmacology 2005. 13 (4): 376-380.

Rawson, R., Glasner, S., Brecht, M. L., Farabee, D.. A randomized comparison of 4 vs. 16 weeks of psychosocial treatment for stimulant users. 2021. 124 (#Issue#): 108274-#End Page#.

Rawson, Richard A., McCann, Michael J., Flammino, Frank, Shoptaw, Steven, Miotto, Karen, Reiber, Chris, and Ling, Walter. A comparison of contingency management and cognitive-behavioral approaches for stimulant-dependent individuals. Addiction (Abingdon, England) 2006. 101 (2): 267-274.

Reback, C. J., Grant, D. L., Fletcher, J. B., Branson, C. M., Shoptaw, S., Bowers, J. R., Charania, M., and Mansergh, G.. Text messaging reduces HIV risk behaviors among methamphetamine-using men who have sex with men. AIDS and behavior 2012. 16 (7): 1993-2002.

Reback, Cathy J., Fletcher, Jesse B., and Leibowitz, Arleen A.. Cost effectiveness of text messages to reduce methamphetamine use and HIV sexual risk behaviors among men who have sex with men. Journal of substance abuse treatment 2019. 100 (#Issue#): 59-63.

Reback, Cathy J., Peck, James A., Dierst-Davies, Rhodri, Nuno, Miriam, Kamien, Jonathan B., and Amass, Leslie. Contingency management among homeless, out-of-treatment men who have sex with men. Journal of substance abuse treatment 2010. 39 (3): 255-263.

Reif, S., Wechsberg, W. M., and Dennis, M. L.. Reduction of co-occurring distress and HIV risk behaviors among women substance abusers. Journal of Prevention and Intervention in the Community 2002. 22 (2): 61-80.

Reitzel, Lorraine R., Nguyen, Nga, Eischen, Sara, Thomas, Janet, and Okuyemi, Kolawole S.. Is smoking cessation associated with worse comorbid substance use outcomes among homeless adults?. Addiction (Abingdon, England) 2014. 109 (12): 2098-2104.

Reynolds, Anna R., Strickland, Justin C., Stoops, William W., Lile, Joshua A., and Rush, Craig R.. Buspirone maintenance does not alter the reinforcing, subjective, and cardiovascular effects of intranasal methamphetamine. Drug and alcohol dependence 2017. 181 (#Issue#): 25-29.

Riggs, Paula D., Winhusen, Theresa, Davies, Robert D., Leimberger, Jeffrey D., Mikulich-Gilbertson, Susan, Klein, Constance, Macdonald, Marilyn, Lohman, Michelle, Bailey, Genie L., Haynes, Louise, Jaffee, William B., Haminton, Nancy, Hodgkins, Candace, Whitmore, Elizabeth, Trello-Rishel, Kathlene, Tamm, Leanne, Acosta, Michelle C., Royer-Malvestuto, Charlotte, Subramaniam, Geetha, Fishman, Marc, Holmes, Beverly W., Kaye, Mary Elyse, Vargo, Mark A., Woody, George E., Nunes, Edward V., and Liu, David. Randomized controlled trial of osmotic-release methylphenidate with cognitive-behavioral therapy in adolescents with attention-deficit/hyperactivity disorder and substance use disorders. Journal of the American Academy of Child and Adolescent Psychiatry 2011. 50 (9): 903-914.

Rohsenow, D. J., Tidey, J. W., Martin, R. A., Colby, S. M., Sirota, A. D., Swift, R. M., and Monti, P. M.. Contingent Vouchers and Motivational Interviewing for Cigarette Smokers in Residential Substance Abuse Treatment. Journal of substance abuse treatment 2015. 55 (#Issue#): 29-38.

Rosen, M. I., Dieckhaus, K., McMahon, T. J., Valdes, B., Petry, N. M., Cramer, J., and Rounsaville, B.. Improved adherence with contingency management. AIDS patient care and STDs 2007. 21 (1): 30-39.

Rosenblum, A., Cleland, C., Magura, S., Mahmood, D., Kosanke, N., and Foote, J.. Moderators of effects of motivational enhancements to cognitive behavioral therapy. American Journal of Drug and Alcohol Abuse 2005. 31 (1): 35-58.

Rosenblum, Andrew, Magura, Stephen, Kayman, Deborah J., and Fong, Chunki. Motivationally enhanced group counseling for substance users in a soup kitchen: a randomized clinical trial. Drug and alcohol dependence 2005. 80 (1): 91-103.

Rotheram-Borus, Mary Jane, Rhodes, Fen, Desmond, Katherine, and Weiss, Robert E.. Reducing HIV risks among active injection drug and crack users: the safety counts program. AIDS and behavior 2010. 14 (3): 658-668.

Rubioz, G., Martine, I., Recio, A., Ponce, G., Lopez-Munoz, F., Alamo, C., Jimenez-Arriero, M. A., and Palomo, T.. Risperidone versus zuclopenthixol in the treatment of schizophrenia with substance abuse comorbidity: A long-term randomized, controlled, crossover study. European Journal of Psychiatry 2006. 20 (3): 133-146.

Ruglass, Lesia M., Hien, Denise A., Hu, Mei Chen, and Campbell, Aimee N. C.. Associations between post-traumatic stress symptoms, stimulant use, and treatment outcomes: a secondary analysis of NIDA's Women and Trauma study. The American journal on addictions 2014. 23 (1): 90-95.

Runarsdottir, Valgerdur, Hansdottir, Ingunn, Tyrfingsson, Thorarinn, Einarsson, Magnus, Dugosh, Karen, Royer-Malvestuto, Charlotte, Pettinati, Helen, Khalsa, Jag, and Woody, George E.. Extended-Release Injectable Naltrexone (XR-NTX) With Intensive Psychosocial Therapy for Amphetamine-Dependent Persons Seeking Treatment: A Placebo-Controlled Trial. Journal of addiction medicine 2017. 11 (3): 197-204.

Rush, Craig R., Stoops, William W., Lile, Joshua A., Glaser, Paul E. A., and Hays, Lon R.. Physiological and subjective effects of acute intranasal methamphetamine during atomoxetine maintenance. Pharmacology, biochemistry, and behavior 2011. 100 (1): 40-47.

Rush, Craig R., Stoops, William W., Lile, Joshua A., Glaser, Paul E. A., and Hays, Lon R.. Subjective and physiological effects of acute intranasal methamphetamine during d-amphetamine maintenance. Psychopharmacology 2011. 214 (3): 665-674.

Sadeghi, Hasan, Ebrahimi, Leyla, and Vatandoust, Leyla. Effectiveness of Hope Therapy Protocol on Depression and Hope in Amphetamine Users. International journal of high risk behaviors & addiction 2015. 4 (4): e21905-#End Page#.

Saitz, R., Cheng, D. M., Winter, M., Kim, T. W., Meli, S. M., Allensworth-Davies, D., Lloyd-Travaglini, C. A., and Samet, J. H.. Chronic care management for dependence on alcohol and other drugs: The AHEAD randomized trial. JAMA - Journal of the American Medical Association 2013. 310 (11): 1156-1167.

Saitz, Richard, Palfai, Tibor P. A., Cheng, Debbie M., Alford, Daniel P., Bernstein, Judith A., Lloyd-Travaglini, Christine A., Meli, Seville M., Chaisson, Christine E., and Samet, Jeffrey H.. Screening and brief intervention for drug use in primary care: the ASPIRE randomized clinical trial. JAMA 2014. 312 (5): 502-513.

Santisteban, Daniel A., Mena, Maite P., and McCabe, Brian E.. Preliminary results for an adaptive family treatment for drug abuse in Hispanic youth. Journal of family psychology : JFP : journal of the Division of Family Psychology of the American Psychological Association (Division 43) 2011. 25 (4): 610-614.

Santos, Glenn Milo. Substance use and alcohol among key populations at risk for hiv: Novel approaches in intervention development and evaluation. Dissertation Abstracts International: Section B: The Sciences and Engineering 2015. 75 (11-B(E)): No-Specified.

Santos, Glenn Milo, Coffin, Phillip O., Vittinghoff, Eric, DeMicco, Erin, Das, Moupali, Matheson, Tim, Raiford, Jerris L., Carry, Monique, Colfax, Grant, Herbst, Jeffrey H., and Dilley, James W.. Substance use and drinking outcomes in Personalized Cognitive Counseling randomized trial for episodic substance-using men who have sex with men. Drug and alcohol dependence 2014. 138 (#Issue#): 234-239.

Santos, Glenn Milo, Coffin, Phillip, Santos, Deirdre, Huffaker, Shannon, Matheson, Tim, Euren, Jason, DeMartini, Anna, Rowe, Christopher, Hahn, Judith A., Vlahov, David, Vittinghoff, Eric, and Batki, Steven L.. Feasibility, Acceptability, and Tolerability of Targeted Naltrexone for Nondependent Methamphetamine-Using and Binge-Drinking Men Who Have Sex with Men. Journal of acquired immune deficiency syndromes (1999) 2016. 72 (1): 21-30.

Schoedel, K. A., Meier, D., Chakraborty, B., Manniche, P. M., and Sellers, E. M.. Subjective and objective effects of the novel triple reuptake inhibitor tesofensine in recreational stimulant users. Clinical pharmacology and therapeutics 2010. 88 (1): 69-78.

Schuler, Maureen E., Nair, Prasanna, and Kettinger, Laurie. Drug-exposed infants and developmental outcome: effects of a home intervention and ongoing maternal drug use. Archives of pediatrics & adolescent medicine 2003. 157 (2): 133-138.

Schumacher, Joseph E., Mennemeyer, Stephen T., Milby, Jesse B., Wallace, Dennis, and Nolan, Kim. Costs and effectiveness of substance abuse treatments for homeless persons. The journal of mental health policy and economics 2002. 5 (1): 33-42.

Schwartz, Robert P., Gryczynski, Jan, Mitchell, Shannon Gwin, Gonzales, Arturo, Moseley, Ana, Peterson, Thomas R., Ondersma, Steven J., and O'Grady, Kevin E.. Computerized versus in-person brief intervention for drug misuse: a randomized clinical trial. Addiction (Abingdon, England) 2014. 109 (7): 1091-1098.

Schwinn, Traci Marie, Schinke, Steven Paul, Keller, Bryan, and Hopkins, Jessica. Two- and three-year follow-up from a gender-specific, web-based drug abuse prevention program for adolescent girls. Addictive behaviors 2019. 93 (#Issue#): 86-92.

Scott, C. K. and Dennis, M. L.. Results from two randomized clinical trials evaluating the impact of quarterly recovery management checkups with adult chronic substance users. Addiction (Abingdon, England) 2009. 104 (6): 959-971.

Shearer, J., Wodak, A., Mattick, R. P., Van Beek, I., Lewis, J., Hall, W., and Dolan, K.. Pilot randomized controlled study of dexamphetamine substitution for amphetamine dependence. Addiction (Abingdon, England) 2001. 96 (9): 1289-1296.

Sherman, Susan G., Sutcliffe, Catherine, Srirojn, Bangorn, Latkin, Carl A., Aramratanna, Apinun, and Celentano, David D.. Evaluation of a peer network intervention trial among young methamphetamine users in Chiang Mai, Thailand. Social science & medicine (1982) 2009. 68 (1): 69-79.

Shoptaw, Steven, Landovitz, Raphael J., and Reback, Cathy J.. Contingent Vs. Non-Contingent Rewards: Time-Based Intervention Response Patterns Among Stimulant-Using Men Who Have Sex With Men. Journal of substance abuse treatment 2017. 72 (#Issue#): 19-24.

Sindelar, Jody L., Olmstead, Todd A., and Peirce, Jessica M.. Cost-effectiveness of prize-based contingency management in methadone maintenance treatment programs. Addiction (Abingdon, England) 2007. 102 (9): 1463-1471.

Slesnick, Natasha and Zhang, Jing. Family systems therapy for substance-using mothers and their 8- to 16-year-old children. Psychology of addictive behaviors : journal of the Society of Psychologists in Addictive Behaviors 2016. 30 (6): 619-629.

Smith, T. L., Volpe, F. R., Hashima, J. N., and Schuckit, M. A.. Impact of a stimulant-focused enhanced program on the outcome of alcohol- and/or stimulant-dependent men. Alcoholism, clinical and experimental research 1999. 23 (11): 1772-1779.

Sobell, L. C., Sobell, M. B., and Agrawal, S.. Randomized Controlled Trial of a Cognitive-Behavioral Motivational Intervention in a Group Versus Individual Format for Substance Use Disorders. Psychology of Addictive Behaviors 2009. 23 (4): 672-683.

Spain, David, Crilly, Julia, Whyte, Ian, Jenner, Linda, Carr, Vaughan, and Baker, Amanda. Safety and effectiveness of high-dose midazolam for severe behavioural disturbance in an emergency department with suspected psychostimulant-affected patients. Emergency medicine Australasia : EMA 2008. 20 (2): 112-120.

Specka, Michael, Boning, Antje, Kluwig, Jurgen, Schifano, Fabrizio, Banger, Markus, Lange, Wolfgang, Lax, Hildegard, Marrziniak, Barbel, Schungel, Claudia, and Scherbaum, Norbert. Can reinforcement-based interventions to reduce drug use successfully be adapted to routine opioid maintenance treatment?. Annali dell'Istituto superiore di sanita 2013. 49 (4): 358-364.

Spoth, R., Redmond, C., Shin, C., Greenberg, M., Feinberg, M., and Schainker, L.. PROSPER community-university partnership delivery system effects on substance misuse through 6 1/2years past baseline from a cluster randomized controlled intervention trial. Preventive medicine 2013. 56 (3-4): 190-196.

Spoth, Richard, Redmond, Cleve, Shin, Chungyeol, Greenberg, Mark, Clair, Scott, and Feinberg, Mark. Substance-use outcomes at 18 months past baseline: the PROSPER Community-University Partnership Trial. American journal of preventive medicine 2007. 32 (5): 395-402.

Stahler, G. J., Shipley, T. F. J., Bartelt, D., DuCette, J. P., and Shandler, I. W.. Evaluating alternative treatments for homeless substance-abusing men: outcomes and predictors of success. Journal of addictive diseases 1995. 14 (4): 151-167.

Stallvik, M., Gastfriend, D. R., and Nordahl, H. M.. Matching patients with substance use disorder to optimal level of care with the ASAM Criteria software. Journal of substance use 2015. 20 (6): 389-398.

Stein, M. D., Charuvastra, A., Maksad, J., and Anderson, B. J.. A randomized trial of a brief alcohol intervention for needle exchangers (BRAINE). Addiction (Abingdon, England) 2002. 97 (6): 691-700.

Sterk, Claire E., Theall, Katherine P., Elifson, Kirk W., and Kidder, Daniel. HIV risk reduction among African-American women who inject drugs: a randomized controlled trial. AIDS and behavior 2003. 7 (1): 73-86.

Stoops, William W., Bennett, J. Adam, Lile, Joshua A., Sevak, Rajkumar J., and Rush, Craig R.. Influence of aripiprazole pretreatment on the reinforcing effects of methamphetamine in humans. Progress in neuro-psychopharmacology & biological psychiatry 2013. 47 (#Issue#): 111-117.

Strantz, I. H. and Welch, S. P.. Postpartum women in outpatient drug abuse treatment: correlates of retention/completion. Journal of psychoactive drugs 1995. 27 (4): 357-373.

Stuyt, Elizabeth B., Sajbel, Terrie A., and Allen, Michael H.. Differing effects of antipsychotic medications on substance abuse treatment patients with co-occurring psychotic and substance abuse disorders. The American journal on addictions 2006. 15 (2): 166-173.

Sutcliffe, Catherine G., Aramrattana, Apinun, Sherman, Susan G., Sirirojn, Bangorn, German, Danielle, Wongworapat, Kanlaya, Quan, Vu Minh, Keawvichit, Rassamee, and Celentano, David D.. Incidence of HIV and sexually transmitted infections and risk factors for acquisition among young methamphetamine users in northern Thailand. Sexually transmitted diseases 2009. 36 (5): 284-289.

Sutcliffe, Catherine G., German, Danielle, Sirirojn, Bangorn, Latkin, Carl, Aramrattana, Apinun, Sherman, Susan G., and Celentano, David D.. Patterns of methamphetamine use and symptoms of depression among young adults in northern Thailand. Drug and alcohol dependence 2009. 101 (3): 146-151.

Suvanchot, K. Sinsak, Somrongthong, Ratana, and Phukhao, Darunee. Efficacy of group motivational interviewing plus brief cognitive behavior therapy for relapse in amphetamine users with co-occurring psychological problems at Southern Psychiatric Hospital in Thailand. Journal of the Medical Association of Thailand = Chotmaihet thangphaet 2012. 95 (8): 1075-1080.

Swartz, M. S., Wagner, H. R., Swanson, J. W., Stroup, T. S., McEvoy, J. P., Reimherr, F., Miller, D. D., McGee, M., Khan, A., Canive, J. M., Davis, S. M., Hsiao, J. K., and Lieberman, J. A.. The effectiveness of antipsychotic medications in patients who use or avoid illicit substances: Results from the CATIE study. Schizophrenia research 2008. 100 (1-3): 39-52.

Tait, Robert J., McKetin, Rebecca, Kay-Lambkin, Frances, Carron-Arthur, Bradley, Bennett, Anthony, Bennett, Kylie, Christensen, Helen, and Griffiths, Kathleen M.. Six-month outcomes of a Web-based intervention for users of amphetamine-type stimulants: randomized controlled trial. Journal of medical Internet research 2015. 17 (4): e105-#End Page#.

Takano, Ayumi, Miyamoto, Yuki, Shinozaki, Tomohiro, Matsumoto, Toshihiko, and Kawakami, Norito. Effect of a web-based relapse prevention program on abstinence among Japanese drug users: A pilot randomized controlled trial. Journal of substance abuse treatment 2020. 111 (#Issue#): 37-46.

Thornton, C. C., Gottheil, E., Weinstein, S. P., and Kerachsky, R. S.. Patient-treatment matching in substance abuse: Drug addiction severity. Journal of substance abuse treatment 1998. 15 (6): 505-511.

Thornton, C. C., Patkar, A. A., Murray, H. W., Mannelli, P., Gottheil, E., Vergare, M. J., and Weinstein, S. P.. High- and low-structure treatments for substance dependence: Role of learned helplessness. American Journal of Drug and Alcohol Abuse 2003. 29 (3): 567-584.

Thornton, C., Gottheil, E., Patkar, A., and Weinstein, S.. Coping styles and response to high versus low-structure individual counseling for substance abuse. American Journal on Addictions 2003. 12 (1): 29-42.

Thylstrup, B. and Hesse, M.. Impulsive lifestyle counseling to prevent dropout from treatment for substance use disorders in people with antisocial personality disorder: A randomized study. Addictive behaviors 2016. 57 (#Issue#): 48-54.

Tiihonen, Jari, Krupitsky, Evgeny, Verbitskaya, Elena, Blokhina, Elena, Mamontova, Olga, Fohr, Jaana, Tuomola, Pekka, Kuoppasalmi, Kimmo, Kiviniemi, Vesa, and Zwartau, Edwin. Naltrexone implant for the treatment of polydrug dependence: a randomized controlled trial. The American journal of psychiatry 2012. 169 (5): 531-536.

Tiihonen, Jari, Kuoppasalmi, Kimmo, Fohr, Jaana, Tuomola, Pekka, Kuikanmaki, Outi, Vorma, Helena, Sokero, Petteri, Haukka, Jari, and Meririnne, Esa. A comparison of aripiprazole, methylphenidate, and placebo for amphetamine dependence. The American journal of psychiatry 2007. 164 (1): 160-162.

Timko, C., DeBenedetti, A., and Billow, R.. Intensive referral to 12-Step self-help groups and 6-month substance use disorder outcomes. Addiction (Abingdon, England) 2006. 101 (5): 678-688.

Tracy, Kathlene, Babuscio, Theresa, Nich, Charla, Kiluk, Brian, Carroll, Kathleen M., Petry, Nancy M., and Rounsaville, Bruce J.. Contingency Management to reduce substance use in individuals who are homeless with co-occurring psychiatric disorders. The American journal of drug and alcohol abuse 2007. 33 (2): 253-258.

Tucker JS, D'Amico EJ Ewing BA Miles JN Pedersen ER. A group-based motivational interviewing brief intervention to reduce substance use and sexual risk behavior among homeless young adults. Journal of substance abuse treatment 2017. 76 (#Issue#): 20-#End Page#.

Tuten, Michelle, Shadur, Julia M., Stitzer, Maxine, and Jones, Hendree E.. A Comparison of Reinforcement Based Treatment (RBT) versus RBT plus Recovery Housing (RBTRH). Journal of substance abuse treatment 2017. 72 (#Issue#): 48-55.

Vaglum, P. and Fossheim, I.. Differential treatment of young abusers: A quasi-experimental study of a 'therapeutic community' in a psychiatric hospital. Journal of drug issues 1980. 10 (4): 505-515.

van Emmerik-van, Oortmerssen K.. Cognitive behavioural therapy for patients with ADHD and substance use disorders. ADHD Attention Deficit and Hyperactivity Disorders 2015. 7 (#Issue#): S6-#End Page#.

Verachai, V., Rukngan, W., Chawanakrasaesin, K., Nilaban, S., Suwanmajo, S., Thanateerabunjong, R., Kaewkungwal, J., and Kalayasiri, R.. Treatment of methamphetamine-induced psychosis: a double-blind randomized controlled trial comparing haloperidol and quetiapine. Psychopharmacology 2014. 231 (16): 3099-3108.

Wachtel SR, Ortengren A.. The effects of acute haloperidol or risperidone on subjective responses to methamphetamine in healthy volunteers. Drug and alcohol dependence 2002. 68 (1): 23-#End Page#.

Wagoner, Joyce L. and Piazza, Nick J.. Group therapy for adult substance abusers on probation. Journal of Offender Rehabilitation 1993. 19 (3-4): 41-56.

Ward, Catherine L., Mertens, Jennifer R., Bresick, Graham F., Little, Francesca, and Weisner, Constance M.. Screening and brief intervention for substance misuse: Does it reduce aggression and HIV-related risk behaviours?. Alcohol and alcoholism (Oxford, Oxfordshire) 2015. 50 (3): 302-309.

Warden, Diane, Riggs, Paula D., Min, Sung joon, Mikulich-Gilbertson, Susan K., Tamm, Leanne, Trello-Rishel, Kathlene, and Winhusen, Theresa. Major depression and treatment response in adolescents with ADHD and substance use disorder. Drug and alcohol dependence 2012. 120 (1-3): 214-219.

Webster LR, Smith M.. Randomized, double-blind, placebo- and comparator controlled human abuse liability study of an experimental, triple monoamine reuptake inhibitor in recreational drug abusers. Clinical pharmacology in drug development 2016. 5 (#Issue#): 36-#End Page#.

Wechsberg, Wendee M., Luseno, Winnie K., Lam, Wendy K. K., Parry, Charles D. H., and Morojele, Neo K.. Substance use, sexual risk, and violence: HIV prevention intervention with sex workers in Pretoria. AIDS and behavior 2006. 10 (2): 131-137.

Wernette, G. T., Plegue, M., Kahler, C. W., Sen, A., and Zlotnick, C.. A Pilot Randomized Controlled Trial of a Computer-Delivered Brief Intervention for Substance Use and Risky Sex during Pregnancy. Journal of Women's Health 2018. 27 (1): 83-92.

Wesson, D. R., Smith, D. E., Lerner, S. E., and Kettner, V. R.. Treatment of polydrug users in San Francisco. AMER.J.DRUG.ALCOHOL ABUSE 1974. 1 (2): 159-179.

Westermeyer, J. and Lee, K.. Residential placement for veterans with addiction: American society of addiction medicine criteria vs. a veterans homeless program. Journal of Nervous and Mental Disease 2013. 201 (7): 567-571.

Winhusen, Theresa M., Brigham, Gregory S., Kropp, Frankie, Lindblad, Robert, Gardin, John G., Penn, Pat, Hodgkins, Candace, Kelly, Thomas M., Douaihy, Antoine, McCann, Michael, Love, Lee D., DeGravelles, Eliot, Bachrach, Ken, Sonne, Susan C., Hiott, Bob, Haynes, Louise, Sharma, Gaurav, Lewis, Daniel F., VanVeldhuisen, Paul, Theobald, Jeff, and Ghitza, Udi. A randomized trial of concurrent smoking-cessation and substance use disorder treatment in stimulant-dependent smokers. The Journal of clinical psychiatry 2014. 75 (4): 336-343.

Winhusen, Theresa, Stitzer, Maxine, Woody, George, Brigham, Gregory, Kropp, Frankie, Ghitza, Udi, Lindblad, Robert, Adinoff, Bryon, Green, Cindy, Sharma, Gaurav, and Somoza, Eugene. Design considerations for a study to evaluate the impact of smoking cessation treatment on stimulant use outcomes in stimulant-dependent individuals. Contemporary clinical trials 2012. 33 (1): 197-205.

Witbrodt, J. and Kaskutas, L. A.. Does diagnosis matter? Differential effects of 12-step participation and social networks on abstinence. American Journal of Drug and Alcohol Abuse 2005. 31 (4): 685-707.

Witkiewitz, K., Greenfield, B. L., and Bowen, S.. Mindfulness-based relapse prevention with racial and ethnic minority women. Addictive behaviors 2013. 38 (12): 2821-2824.

Worley MJ, Tate SR Brown SA. Mediational relations between 12-Step attendance, depression and substance use in patients with comorbid substance dependence and major depression. Addiction (Abingdon, England) 2012. 107 (11): 1974-#End Page#.

Wu, Q., Slesnick, N., and Zhang, J.. Understanding the role of emotion-oriented coping in women's motivation for change. Journal of substance abuse treatment 2018. 86 (#Issue#): 1-8.

Wu, Ying, Stanton, Bonita F., Galbraith, Jennifer, Kaljee, Linda, Cottrell, Lesley, Li, Xiaoming, Harris, Carole V., D'Alessandri, Dawn, and Burns, James M.. Sustaining and broadening intervention impact: a longitudinal randomized trial of 3 adolescent risk reduction approaches. Pediatrics 2003. 111 (1): e32-e38.

Xu, X., Yonkers, K. A., and Ruger, J. P.. Economic evaluation of a behavioral intervention versus brief advice for substance use treatment in pregnant women: Results from a randomized controlled trial. BMC pregnancy and childbirth 2017. 17 (1): 83-#End Page#.

Yokotani, Kenji and Tamura, Katsuhiro. Effects of Personalized Feedback Interventions on Drug-Related Reoffending: a Pilot Study. Prevention science : the official journal of the Society for Prevention Research 2015. 16 (8): 1169-1176.

Zawertailo LA, Busto UE Kaplan HL Greenblatt DJ Sellers EM. Comparative abuse liability and pharmacological effects of meprobamate, triazolam, and butabarbital. Journal of clinical psychopharmacology 2003. 23 (3): 269-#End Page#.

Zhang, S. X., Shoptaw, S., Reback, C. J., Yadav, K., and Nyamathi, A. M.. Cost-effective way to reduce stimulant-abuse among gay/bisexual men and transgender women: a randomized clinical trial with a cost comparison. Public health 2018. 154 (#Issue#): 151-160.

### Irrelevant intervention

Chinkijkarn, T., Kanato, M.. The Effects of Drug Camp in Treatment of Methamphetamine Use with a New Behavioral Change Model: A Quasi-Experimental Study. 2021. 51(1) (#Issue#): 57-65-#End Page#.

Marinelli-Casey, Patricia, Gonzales, Rachel, Hillhouse, Maureen, Ang, Alfonso, Zweben, Joan, Cohen, Judith, Hora, Peggy Fulton, Rawson, Richard A., and Methamphetamine Treatment Project Corp. Drug court treatment for methamphetamine dependence: treatment response and posttreatment outcomes. Journal of substance abuse treatment 2008. 34 (2): 242-248.

### Follow-up less than 3 months

Ahmadi, J. and Razeghian, Jahromi L. Comparing the effect of buprenorphine and methadone in the reduction of methamphetamine craving: a randomized clinical trial. Trials 6-6-2017. 18 (1): 259-#End Page#.

Ahmadi, Jamshid, Sahraian, Ali, and Biuseh, Mehdi. A randomized clinical trial on the effects of bupropion and buprenorphine on the reduction of methamphetamine craving. Trials 2019. 20 (1): 468-#End Page#.

Alizadehgoradel, J., Imani, S., Nejati, V., and Fathabadi, J.. Mindfulness-based substance abuse treatment (MBSAT) improves executive functions in adolescents with substance use disorders. Neurology Psychiatry and Brain Research 2019. 34 (#Issue#): 13-21.

Batki, S. L., Moon, J., Delucchi, K., Bradley, M., Hersh, D., Smolar, S., Mengis, M., Lefkowitz, E., Sexe, D., Morello, L., Everhart, T., Jones, R. T., and Jacob, P.. Methamphetamine quantitative urine concentrations during a controlled trial of fluoxetine treatment. Preliminary analysis. Annals of the New York Academy of Sciences 2000. 909 (#Issue#): 260-263.

Birath, J. Brandon, Briones, Marisa, Amaya, Stephanie, Shoptaw, Steven, Swanson, Aimee Noelle, Tsuang, John, Furst, Benjamin, Heinzerling, Keith, Obermeit, Lisa, Maes, Lauryn, McKay, Charles, and Wright, Matthew J.. Ibudilast may improve attention during early abstinence from methamphetamine. Drug and alcohol dependence 2017. 178 (#Issue#): 386-390.

Brooks, S. J., Wiemerslage, L., Burch, K., Maiorana, S., Cocolas, E., Schioth, H., Kamaloodien, K., and Stein, D.. The impact of cognitive training in substance use disorder: the effect of working memory training on impulse control in methamphetamine users. Psychopharmacology 2017. 234 (12): 1911-1921.

Casaletto, Kaitlin B., Moore, David J., Woods, Steven Paul, Umlauf, Anya, Scott, J. C., and Heaton, Robert K.. Abbreviated Goal Management Training Shows Preliminary Evidence as a Neurorehabilitation Tool for HIV-associated Neurocognitive Disorders among Substance Users. The Clinical neuropsychologist 2016. 30 (1): 107-130.

Courtney, Kelly E., Ghahremani, Dara G., and Ray, Lara A.. The Effects of Pharmacological Opioid Blockade on Neural Measures of Drug Cue-Reactivity in Humans. Neuropsychopharmacology : official publication of the American College of Neuropsychopharmacology 2016. 41 (12): 2872-2881.

Cruickshank, Christopher C., Montebello, Mark E., Dyer, Kyle R., Quigley, Allan, Blaszczyk, Jozef, Tomkins, Sally, and Shand, Diana. A placebo-controlled trial of mirtazapine for the management of methamphetamine withdrawal. Drug and alcohol review 2008. 27 (3): 326-333.

De La Garza, R. and Yoon, J. H.. Evaluation of the effects of rivastigmine on cigarette smoking by methamphetamine-dependent volunteers. Progress in neuro-psychopharmacology & biological psychiatry 2011. 35 (8): 1827-1830.

De La Garza, R., Mahoney, J. J., Culbertson, C., Shoptaw, S., and Newton, T. F.. The acetylcholinesterase inhibitor rivastigmine does not alter total choices for methamphetamine, but may reduce positive subjective effects, in a laboratory model of intravenous self-administration in human volunteers. Pharmacology, biochemistry, and behavior 2008. 89 (2): 200-208.

De La Garza, R., Newton, T. F., Haile, C. N., Yoon, J. H., Nerumalla, C. S., Mahoney, J. J., and Aziziyeh, A.. Rivastigmine reduces "Likely to use methamphetamine" in methamphetamine-dependent volunteers. Progress in neuro-psychopharmacology & biological psychiatry 2012. 37 (1): 141-146.

De La Garza, Richard, Zorick, Todd, Heinzerling, Keith G., Nusinowitz, Steve, London, Edythe D., Shoptaw, Steven, Moody, David E., and Newton, Thomas F.. The cardiovascular and subjective effects of methamphetamine combined with gamma-vinyl-gamma-aminobutyric acid (GVG) in non-treatment seeking methamphetamine-dependent volunteers. Pharmacology, biochemistry, and behavior 2009. 94 (1): 186-193.

Dean, Andy C., Nurmi, Erika L., Moeller, Scott J., Amir, Nader, Rozenman, Michelle, Ghahremani, Dara G., Johnson, Maritza, Berberyan, Robert, Hellemann, Gerhard, Zhang, Ziwei, and London, Edythe D.. No effect of attentional bias modification training in methamphetamine users receiving residential treatment. Psychopharmacology 2019. 236 (2): 709-721.

Dean, Andy C., Sevak, Rajkumar J., Monterosso, John R., Hellemann, Gerhard, Sugar, Catherine A., and London, Edythe D.. Acute modafinil effects on attention and inhibitory control in methamphetamine-dependent humans. Journal of studies on alcohol and drugs 2011. 72 (6): 943-953.

Galloway, G. P., Buscemi, R., Coyle, J. R., Flower, K., Siegrist, J. D., Fiske, L. A., Baggott, M. J., Li, L., Polcin, D., Chen, C. Y. A., and Mendelson, J.. A randomized, placebo-controlled trial of sustained-release dextroamphetamine for treatment of methamphetamine addiction. Clinical pharmacology and therapeutics 2011. 89 (2): 276-282.

Ghahremani, Dara G., Tabibnia, Golnaz, Monterosso, John, Hellemann, Gerhard, Poldrack, Russell A., and London, Edythe D.. Effect of modafinil on learning and task-related brain activity in methamphetamine-dependent and healthy individuals. Neuropsychopharmacology : official publication of the American College of Neuropsychopharmacology 2011. 36 (5): 950-959.

Ghasemi, Afsaneh, Rahimi Foroshani, Abbass, Kheibar, Nasrin, Latifi, Marziye, Khanjani, Narges, Eshagh Afkari, Mohammad, Taghdisi, Mohammad Hossein, Ghasemi, Faranak, Shojaeizadeh, Davoud, and Dastoorpour, Maryam. Effects of family-centered empowerment model based education program on quality of life in methamphetamine users and their families. Iranian Red Crescent medical journal 2014. 16 (3): e13375-#End Page#.

Grant, Jon E., Odlaug, Brian L., and Kim, Suck Won. A double-blind, placebo-controlled study of N-acetyl cysteine plus naltrexone for methamphetamine dependence. European neuropsychopharmacology : the journal of the European College of Neuropsychopharmacology 2010. 20 (11): 823-828.

Heinzerling, Keith G., Gadzhyan, Janette, van Oudheusden, Henry, Rodriguez, Felipe, McCracken, James, and Shoptaw, Steven. Pilot randomized trial of bupropion for adolescent methamphetamine abuse/dependence. The Journal of adolescent health : official publication of the Society for Adolescent Medicine 2013. 52 (4): 502-505.

Hester, Robert, Lee, Nicole, Pennay, Amy, Nielsen, Suzi, and Ferris, Jason. The effects of modafinil treatment on neuropsychological and attentional bias performance during 7-day inpatient withdrawal from methamphetamine dependence. Experimental and clinical psychopharmacology 2010. 18 (6): 489-497.

Jan, Reem K., Lin, Joanne C., McLaren, Donald G., Kirk, Ian J., Kydd, Rob R., and Russell, Bruce R.. The effects of methylphenidate on cognitive control in active methamphetamine dependence using functional magnetic resonance imaging. Frontiers in psychiatry 2014. 5 (#Issue#): 20-#End Page#.

Javdan, N. S., Ghaderi, A., Banafshe, H. R.. The effects of quetiapine on craving and withdrawal symptoms in methamphetamine abuse: A randomized, double-blind, placebo-controlled trial. 2020. 10(4) (no pagination) (#Issue#): -#End Page#.

Javdan, Najme Sadat, Ghoreishi, Fatemeh Sadat, Sehat, Mojtaba, Ghaderi, Amir, and Banafshe, Hamid Reza. Mental health and cognitive function responses to quetiapine in patients with methamphetamine abuse under methadone maintenance treatment. Journal of affective disorders 2019. 251 (#Issue#): 235-241.

Jeong, H., Yoon, S., Sung, Y. H., Kim, J., Lyoo, I. K., Yurgelun-Todd, D. A., Renshaw, P. F.. Effects of cytidine-5'-diphosphate choline on gray matter volumes in methamphetamine-dependent patients: A randomized, double-blind, placebo-controlled study. 2021. 143 (#Issue#): 215-221-#End Page#.

Johnson, Bankole A., Ait-Daoud, Nassima, Elkashef, Ahmed M., Smith, Edwina V., Kahn, Roberta, Vocci, Francis, Li, Shou Hua, Bloch, Daniel A., and Methamphetamine Study Group. A preliminary randomized, double-blind, placebo-controlled study of the safety and efficacy of ondansetron in the treatment of methamphetamine dependence. The international journal of neuropsychopharmacology 2008. 11 (1): 1-14.

Johnson, Bankole A., Roache, John D., Ait-Daoud, Nassima, Wallace, Christopher, Wells, Lynda, Dawes, Michael, and Wang, Yanmei. Effects of isradipine, a dihydropyridine-class calcium-channel antagonist, on d-methamphetamine's subjective and reinforcing effects. The international journal of neuropsychopharmacology 2005. 8 (2): 203-213.

Johnson, Bankole A., Roache, John D., Ait-Daoud, Nassima, Wells, Lynda T., Wallace, Christopher L., Dawes, Michael A., Liu, Lei, and Wang, Xin Qun. Effects of acute topiramate dosing on methamphetamine-induced subjective mood. The international journal of neuropsychopharmacology 2007. 10 (1): 85-98.

Johnson, Bankole A., Wells, Lynda T., Roache, John D., Wallace, Christopher L., Ait-Daoud, Nassima, Dawes, Michael A., Liu, Lei, Wang, Xin Qun, and Javors, Martin A.. Kinetic and cardiovascular effects of acute topiramate dosing among non-treatment-seeking, methamphetamine-dependent individuals. Progress in neuro-psychopharmacology & biological psychiatry 2007. 31 (2): 455-461.

Kalechstein, Ari D., Yoon, Jin H., Croft, Daniel E., Jaeggi, Susanne, Mahoney, James J., and De La Garza, Richard. Low dose, short-term rivastigmine administration does not affect neurocognition in methamphetamine dependent individuals. Pharmacology, biochemistry, and behavior 2011. 99 (3): 423-427.

Keoleian, Victoria, Stalcup, S Alex, Polcin, Douglas L., Brown, Michelle, and Galloway, Gantt. A cognitive behavioral therapy-based text messaging intervention for methamphetamine dependence. Journal of psychoactive drugs 2013. 45 (5): 434-442.

Kohno, M., Dennis, L. E., McCready, H., Schwartz, D. L., Hoffman, W. F., and Korthuis, P. T.. A preliminary randomized clinical trial of naltrexone reduces striatal resting state functional connectivity in people with methamphetamine use disorder. Drug and alcohol dependence 11-1-2018. 192 (#Issue#): 186-192.

Kohno, Milky, Morales, Angelica M., Dennis, Laura E., McCready, Holly, Hoffman, William F., and Korthuis, P. Todd. Effects of Naltrexone on Large-Scale Network Interactions in Methamphetamine Use Disorder. Frontiers in psychiatry 2019. 10 (#Issue#): 603-#End Page#.

Lee, Nicole, Pennay, Amy, Hester, Robert, McKetin, Rebecca, Nielsen, Suzi, and Ferris, Jason. A pilot randomised controlled trial of modafinil during acute methamphetamine withdrawal: feasibility, tolerability and clinical outcomes. Drug and alcohol review 2013. 32 (1): 88-95.

Li, Michael J., Briones, Marisa S., Heinzerling, Keith G., Kalmin, Mariah M., and Shoptaw, Steven J.. Ibudilast attenuates peripheral inflammatory effects of methamphetamine in patients with methamphetamine use disorder. Drug and alcohol dependence 2020. 206 (#Issue#): 107776-#End Page#.

Lim, A. C., Grodin, E. N., Green, R., Venegas, A., Meredith, L. R., Courtney, K. E., Moallem, N. R., Sayegh, P., London, E. D., Ray, L. A.. Executive function moderates naltrexone effects on methamphetamine-induced craving and subjective responses. 2020. 46 (5): 565-576-#End Page#.

Magee, Joshua C. and Winhusen, Theresa. The coupling of nicotine and stimulant craving during treatment for stimulant dependence. Journal of consulting and clinical psychology 2016. 84 (3): 230-237.

Magee, Joshua C., Lewis, Daniel F., and Winhusen, Theresa. Evaluating Nicotine Craving, Withdrawal, and Substance Use as Mediators of Smoking Cessation in Cocaine- and Methamphetamine-Dependent Patients. Nicotine & tobacco research : official journal of the Society for Research on Nicotine and Tobacco 2016. 18 (5): 1196-1201.

Mahoney, James J., Jackson, Brian J., Kalechstein, Ari D., De La Garza, Richard, Chang, Lee C., and Newton, Thomas F.. Acute modafinil exposure reduces daytime sleepiness in abstinent methamphetamine-dependent volunteers. The international journal of neuropsychopharmacology 2012. 15 (9): 1241-1249.

McGregor, Catherine, Srisurapanont, Manit, Mitchell, Amanda, Wickes, Wendy, and White, Jason M.. Symptoms and sleep patterns during inpatient treatment of methamphetamine withdrawal: a comparison of mirtazapine and modafinil with treatment as usual. Journal of substance abuse treatment 2008. 35 (3): 334-342.

Modarresi, A., Eslami, K., Kouti, L., Hassanvand, R., Javadi, M., and Sayyah, M.. Amantadine reduces persistent fatigue during post-acute withdrawal phase in methamphetamine abstained individuals: A randomized placebo-controlled trial. Journal of substance use 2018. 23 (6): 584-590.

Moore, D. J., Montoya, J. L., Blackstone, K., Rooney, A., Gouaux, B., Georges, S., Depp, C. A., Atkinson, J. H., and TMARC Group. Preliminary Evidence for Feasibility, Use, and Acceptability of Individualized Texting for Adherence Building for Antiretroviral Adherence and Substance Use Assessment among HIV-Infected Methamphetamine Users. AIDS Res Treat 2013. 2013 (#Issue#): 585143-#End Page#.

Moore, David J., Pasipanodya, Elizabeth C., Umlauf, Anya, Rooney, Alexandra S., Gouaux, Ben, Depp, Colin A., Atkinson, J. Hampton, and Montoya, Jessica L.. Individualized texting for adherence building (iTAB) for methamphetamine users living with HIV: A pilot randomized clinical trial. Drug and alcohol dependence 2018. 189 (#Issue#): 154-160.

Moosavi, Seyed Mohamad, Yazdani-Charati, Jamshid, and Amini, Fatemeh. Effects of Modafinil on Sleep Pattern during Methamphetamine Withdrawal: A Double-blind Randomized Controlled Trial. Addiction & health 2019. 11 (3): 165-172.

Mousavi, Seyed Ghafur, Sharbafchi, Mohammad Reza, Salehi, Mehrdad, Peykanpour, Mohammad, Karimian Sichani, Naeemeh, and Maracy, Mohammad. The efficacy of N-acetylcysteine in the treatment of methamphetamine dependence: a double-blind controlled, crossover study. Archives of Iranian medicine 2015. 18 (1): 28-33.

Newton, Thomas F., De La Garza, Richard, and Grasing, Ken. The angiotensin-converting enzyme inhibitor perindopril treatment alters cardiovascular and subjective effects of methamphetamine in humans. Psychiatry research 2010. 179 (1): 96-100.

Newton, Thomas F., Reid, Malcolm S., De La Garza, Richard, Mahoney, James J., Abad, Antonio, Condos, Rany, Palamar, Joseph, Halkitis, Perry N., Mojisak, Jurji, Anderson, Ann, Li, Shou Hua, and Elkashef, Ahmed. Evaluation of subjective effects of aripiprazole and methamphetamine in methamphetamine-dependent volunteers. The international journal of neuropsychopharmacology 2008. 11 (8): 1037-1045.

Newton, Thomas F., Roache, John D., De La Garza, Richard, Fong, Tim, Wallace, Christopher L., Li, Shou Hua, Elkashef, Ahmed, Chiang, Nora, and Kahn, Roberta. Safety of intravenous methamphetamine administration during treatment with bupropion. Psychopharmacology 2005. 182 (3): 426-435.

Newton, Thomas F., Roache, John D., De La Garza, Richard, Fong, Timothy, Wallace, Christopher L., Li, Shou Hua, Elkashef, Ahmed, Chiang, Nora, and Kahn, Roberta. Bupropion reduces methamphetamine-induced subjective effects and cue-induced craving. Neuropsychopharmacology : official publication of the American College of Neuropsychopharmacology 2006. 31 (7): 1537-1544.

Pike, E., Stoops, W. W., and Rush, C. R.. Acute buspirone dosing enhances abuse-related subjective effects of oral methamphetamine. Pharmacology Biochemistry and Behavior 2016. 150-151 (#Issue#): 87-93.

Pike, Erika, Stoops, William W., Hays, Lon R., Glaser, Paul E. A., and Rush, Craig R.. Methamphetamine self-administration in humans during D-amphetamine maintenance. Journal of clinical psychopharmacology 2014. 34 (6): 675-681.

Rabiey, Ali, Hassani-Abharian, Peyman, Farhad, Majid, Moravveji, Ali Reza, Akasheh, Goodarz, and Banafshe, Hamid Reza. Atomoxetine Efficacy in Methamphetamine Dependence during Methadone Maintenance Therapy. Archives of Iranian medicine 2019. 22 (12): 692-698.

Ray, Lara A., Bujarski, Spencer, Courtney, Kelly E., Moallem, Nathasha R., Lunny, Katy, Roche, Daniel, Leventhal, Adam M., Shoptaw, Steve, Heinzerling, Keith, London, Edythe D., and Miotto, Karen. The Effects of Naltrexone on Subjective Response to Methamphetamine in a Clinical Sample: a Double-Blind, Placebo-Controlled Laboratory Study. Neuropsychopharmacology : official publication of the American College of Neuropsychopharmacology 2015. 40 (10): 2347-2356.

Rezaei, Farzin, Emami, Maryam, Zahed, Shakiba, Morabbi, Mohammad Javad, Farahzadi, Mohammadhadi, and Akhondzadeh, Shahin. Sustained-release methylphenidate in methamphetamine dependence treatment: a double-blind and placebo-controlled trial. Daru : journal of Faculty of Pharmacy, Tehran University of Medical Sciences 2015. 23 (#Issue#): 2-#End Page#.

Rezaei, Farzin, Ghaderi, Ebrahim, Mardani, Roya, Hamidi, Seiran, and Hassanzadeh, Kambiz. Topiramate for the management of methamphetamine dependence: a pilot randomized, double-blind, placebo-controlled trial. Fundamental & clinical pharmacology 2016. 30 (3): 282-289.

Roche, Daniel J. O., Worley, Matthew J., Courtney, Kelly E., Bujarski, Spencer, London, Edythe D., Shoptaw, Steven, and Ray, Lara A.. Naltrexone moderates the relationship between cue-induced craving and subjective response to methamphetamine in individuals with methamphetamine use disorder. Psychopharmacology 2017. 234 (13): 1997-2007.

Samiei, Mercede, Vahidi, Mohammad, Rezaee, Omid, Yaraghchi, Azadeh, and Daneshmand, Reza. Methamphetamine-Associated Psychosis and Treatment With Haloperidol and Risperidone: A Pilot Study. Iranian journal of psychiatry and behavioral sciences 2016. 10 (3): e7988-#End Page#.

Solhi, Hassan, Jamilian, Hamid Reza, Kazemifar, Amir Mohammad, Javaheri, Javad, and Rasti Barzaki, Akram. Methylphenidate vs. resperidone in treatment of methamphetamine dependence: A clinical trial. Saudi pharmaceutical journal : SPJ : the official publication of the Saudi Pharmaceutical Society 2014. 22 (3): 191-194.

Srisurapanont, Manit, Sombatmai, Sangworn, and Boripuntakul, Theerarat. Brief intervention for students with methamphetamine use disorders: a randomized controlled trial. The American journal on addictions 2007. 16 (2): 111-116.

Stauffer, C. S., Moschetto, J. M., McKernan, S., Meinzer, N., Chiang, C., Rapier, R., Hsiang, E., Norona, J., Borsari, B., Woolley, J. D.. Oxytocin-enhanced group therapy for methamphetamine use disorder: Randomized controlled trial. 2020. 116 (#Issue#): 108059-#End Page#.

Sulaiman, Ahmad Hatim, Gill, Jesjeet Singh, Said, Mas Ayu, Zainal, Nor Zuraida, Hussein, Habil Mohamad, and Guan, Ng Chong. A randomized, placebo-controlled trial of aripiprazole for the treatment of methamphetamine dependence and associated psychosis. International journal of psychiatry in clinical practice 2013. 17 (2): 131-138.

Urschel, Harold C., Hanselka, Larry L., and Baron, Michael. A controlled trial of flumazenil and gabapentin for initial treatment of methylamphetamine dependence. Journal of psychopharmacology (Oxford, England) 2011. 25 (2): 254-262.

Verrico, Christopher D., Mahoney, James J., Thompson-Lake, Daisy G. Y., Bennett, Ryan S., Newton, Thomas F., and De La Garza, Richard. Safety and efficacy of varenicline to reduce positive subjective effects produced by methamphetamine in methamphetamine-dependent volunteers. The international journal of neuropsychopharmacology 2014. 17 (2): 223-233.

Wang, G., Ding, F., Chawarski, M. C., Hao, W., Liu, X., Deng, Q., Ouyang, X.. Randomized Controlled Trial of Paliperidone Extended Release Versus Risperidone for the Treatment of Methamphetamine-Associated Psychosis in Chinese Patients. 2020. 11 (no pagination) (#Issue#): -#End Page#.

Wang, G., Zhang, Y., Zhang, S., Chen, H., Xu, Z., Schottenfeld, R. S., Hao, W., and Chawarski, M. C.. Aripiprazole and Risperidone for Treatment of Methamphetamine-Associated Psychosis in Chinese Patients. Journal of substance abuse treatment 2016. 62 (#Issue#): 84-88.

Worley, Matthew J., Swanson, Aimee Noelle, Heinzerling, Keith G., Roche, Daniel J. O., and Shoptaw, Steve. Ibudilast attenuates subjective effects of methamphetamine in a placebo-controlled inpatient study. Drug and alcohol dependence 2016. 162 (#Issue#): 245-250.

Yazdanbakhsh, K., Dehghan, F., Mirzaei, S., Alikhani, M., Tatari, F., and Farnia, V.. The effectiveness of levinson-based cognitive-behavioral therapy on psychological well-being of methamphetamine-dependent patients. Acta Medica Mediterranea 2016. 32 (SpecialIssue5): 2001-2004.

### No outcomes of interest

Burduli, Ekaterina, Skalisky, Jordan, Hirchak, Katherine, Orr, Michael F., Foote, Albert, Granbois, Alexandria, Ries, Richard, Roll, John M., Buchwald, Dedra, McDonell, Michael G., and McPherson, Sterling M. Contingency management intervention targeting co-addiction of alcohol and drugs among American Indian adults: Design, methodology, and baseline data. Clinical trials (London, England) 2018. 15 (6): 587-599.

McPherson, Sterling, Orr, Michael, Lederhos, Crystal, McDonell, Michael, Leickly, Emily, Hirchak, Katherine, Oluwoye, Oladunni A., Murphy, Sean M., Layton, Matthew, and Roll, John M. Decreases in smoking during treatment for methamphetamine-use disorders: preliminary evidence. Behavioural pharmacology 2018. 29 (4): 370-374.

Niu, Tianhua, Li, Jingjing, Wang, Ju, Ma, Jennie Z., and Li, Ming D.. Identification of Novel Signal Transduction, Immune Function, and Oxidative Stress Genes and Pathways by Topiramate for Treatment of Methamphetamine Dependence Based on Secondary Outcomes. Frontiers in psychiatry 2017. 8: 271-.
