# Supplementary material for: Psychosocial and pharmacologic interventions for problematic methamphetamine use: Findings from a scoping review of the literature
Source: PLoS One. 2023 Oct 11;18(10):e0292745. doi: 10.1371/journal.pone.0292745 (PMC10566716; doi:10.1371/journal.pone.0292745)
Supplement: S6 Text — (DOCX) [file pone.0292745.s006.docx]

**S6 Text. Quality appraisal**

**Quality assessment of systematic reviews using AMSTAR-2**

| **Author Year** | **AMSTAR 2 Items** | | | | | | | | | | | | | | | | | | **Overall AMSTAR Rating** |
| --- | --- | --- | --- | --- | --- | --- | --- | --- | --- | --- | --- | --- | --- | --- | --- | --- | --- | --- | --- |
|  | **1** | **2*** | **3** | **4*** | **5** | **6** | **7*** | **8** | **9*** | | **10** | **11*** | | **12** | **13*** | **14** | **15*** | **16** |  |
|  |  |  |  |  |  |  |  |  | **a** | **b** |  | **a** | **b** |  |  |  |  |  |  |
| **Bhatt 2016** | Y | P | N | P | Y | N | N | P | Y | NA | Y | Y | NA | Y | Y | Y | Y | Y | Low |
| **Nourredine 2021** | Y | N | N | P | Y | N | N | P | Y | Y | N | NA | NA | NA | N | N | NA | Y | Critically low |

*Critical domains

NA = not applicable; N = No; P = Partial Yes;

Bhatt M, Zielinski L, Baker-Beal L, Bhatnagar N, Mouravska N, Laplante P, et al. Efficacy and safety of psychostimulants for amphetamine and methamphetamine use disorders: a systematic review and meta-analysis. Syst Rev. 2016 14;5(1):189.

Nourredine M, Jurek L, Angerville B, Longuet Y, de Ternay J, Derveaux A, et al. Use of Topiramate in the Spectrum of Addictive and Eating Disorders: A Systematic Review Comparing Treatment Schemes, Efficacy, and Safety Features. CNS Drugs. 2021 Feb;35(2):177–213.

**Critical appraisal of practice guidelines using AGREE II**

|  | **Domain scores (%)** | | | | | | **Overall assessment** | |
| --- | --- | --- | --- | --- | --- | --- | --- | --- |
| **Author (year)** | **Scope and purpose** | **Stakeholder involvement** | **Rigour of development** | **Clarity and presentation** | **Applicability** | **Editorial independence** | **Overall quality (1-7)** | **Overall recommendation** |
| **Braunwarth 2016** | 71 | 48 | 55 | 95 | 18 | 21 | 4 | Recommended with modifications |

Braunwarth WD, Christ M, Dirks H, et al. S3 practice guideline methamphetamine-related disorders. [Internet]. Berlin (DE): Arztliches Zentrum fur Qualitat in der Medizin; 2016. Available from: https://www.aezq.de/mdb/edocs/pdf/literatur/s3-gl-methamphetamine-relateddisorders-long.pdf

Härtel-Petri R, Krampe-Scheidler A, Braunwarth W-D, Havemann-Reinecke U, Jeschke P, Looser W, et al. Evidence-Based Guidelines for the Pharmacologic Management of Methamphetamine Dependence, Relapse Prevention, Chronic Methamphetamine-Related, and Comorbid Psychiatric Disorders in Post-Acute Settings. Pharmacopsychiatry. 2017 May;50(3):96–104.
