# Supplementary material for: Psychosocial and pharmacologic interventions for problematic methamphetamine use: Findings from a scoping review of the literature
Source: PLoS One. 2023 Oct 11;18(10):e0292745. doi: 10.1371/journal.pone.0292745 (PMC10566716; doi:10.1371/journal.pone.0292745)
Supplement: S7 Text — (DOCX) [file pone.0292745.s007.docx]

**S7 Text. Primary study characteristics**

| **Author Year (refID)**  **Country; Setting**  **Funding** | **Study design**  **Study duration**  **Number of participants** | **Subpopulations or specific participant characteristics** | **Methamphetamine use eligibility criteria**  **Participants with MUD^¥^ diagnosis according to DSM/ICD criteria (%)** | **Age (years)^Ɏ^** | **Male (%)** | **Majority race/ethnicity (%)** | **Substance co-use^§^ (%);**  **Other substance dependence^§^ (%)** | **Intervention comparison** |
| --- | --- | --- | --- | --- | --- | --- | --- | --- |
| **Abdoli 2019** (1)  Iran; Hospital  NR | RCT  12 weeks  80 | NA | MUD (NR)  NR | Range: 20-52 | 0% | NR | NR  NR | - CBT (Marlatt model for relapse prevention) - No treatment |
| **Abdoli 2021** (2)  Iran; Hospital  Non-industry | RCT  52 weeks  80 | Hospitalized for psychosis | MUD (TLC testing or DSM-5)  NR | 32.9 (7.0) | 0% | NR | NR  NR | - Self-compassion training - Control (not further described) |
| **Amiri 2016** (3)  Iran; Outpatient substance use treatment center  NR | RCT  12 weeks  24 | Treatment-seeking | MUD (DSM-IV-TR)  100% | 37.4 (NR) | 100% | NR | NR  NR^†^ | - MM (Regulated 12-session MM) - No treatment (wait list control) |
| **Anderson 2012** (4)  US; Outpatient drug treatment clinic sites  Non-industry | RCT  16 weeks  210 | Treatment-seeking | MUD (DSM-IV; SCID)  100% | 39.0 (8.6) | 59.1% | Caucasian: 70.5% | NR  NR^†^ | - Modafinil 200mg - Modafinil 400mg - Placebo |
| **Anderson 2015** (5)  US; Outpatient clinics  Non-industry | RCT  16 weeks  204 | Treatment-seeking, non-daily methamphetamine users (i.e., ≤29 of the 30 days prior to consent) | MUD (DSM-IV; MINI)  100% | 39.3 (NR) | 65.0% | Caucasian: 69.0% | NR  NR | - Bupropion - Placebo |
| **Aryan 2020** (6)  Iran; Hospital substance dependency clinic  Non-industry | RCT  12 weeks  100 | Treatment-seeking, receiving methadone treatment, at least weekly methamphetamine use during previous 3 months | MUD (DSM-IV)  100% | 30.8 (4.5) | 100% | NR | NR  NR | - Matrix model - Methylphenidate - Matrix model plus methylphenidate - No treatment |
| **Baker 2005** (7–9)  Australia; NR  Non-industry | RCT  26 weeks  214 | Regular methamphetamine users (at least once weekly for the 4 weeks prior to baseline) | Regular use of methamphetamines (at least weekly use)  Approximately 89.8% (DSM-IV; SCID) | 30.2 (7.8)* | 62.6%* | NR | NR  NR | - CBT (4 sessions) - CBT (2 sessions) - Self-help booklet |
| **Briones 2018** (10)  US; Outpatient  Non-industry | RCT  13 weeks  52 | Treatment-seeking | MUD (DSM-IV; SCID)  100% | 35.9 (10.5) | 63.5% | NR | NR  NR^†^ | - Varenicline - Placebo |
| **Brown 2012** (11)  US; Outpatient  Non-industry | RCT  12 weeks  60 | Bipolar depression or major depressive disorder | MUD (DSM-IV; SCID)  100% | 38.4 (9.6) | 54.2% | Caucasian: 77.1% | NR  NR | - Citicoline - Placebo |
| **Chudzynski 2015** (12)  US; Outpatient  Non-industry | RCT  28 weeks  119 | Treatment-seeking | MUD (DSM-IV)  100% | 32.7 (9.4) | 62.2% | Caucasian: 47.1% | NR  NR | - CM (continuous) - CM (intermittent predictable) - CM (intermittent unpredictable) - No treatment |
| **MATES study**  **Ciketic 2013** (13)  **McKetin 2012** (14)  **McKetin 2018** (15)  Australia; Residential rehabilitation facilities, detoxification units  Non-industry | Cohort  52 weeks  501 | NA | Methamphetamine or amphetamine recorded as the primary or secondary drug problem  Approximately 97.0% (DSM-IV; CIDI) | 31.6 (8.1)* | 73.1%* | NR | NR  NR | - Community-based residential rehabilitation - Inpatient or outpatient community-based detoxification - Outpatient counselling - No treatment |
|  | Cohort  156 weeks  461 | NA | Methamphetamine or amphetamine recorded as the primary or secondary drug problem  Approximately 97.0% (DSM-IV; CIDI) | 31.8 (7.9)* | 73.5%* | NR | Alcohol: 71.0%*  Cannabis: 78.0%*  Heroin: 26.0%*  Cocaine: 27.0%*;  NR | - Community-based residential rehabilitation - Inpatient or outpatient community-based detoxification - No treatment |
|  | Cohort  52 weeks  165 | NA | MUD (DSM-IV; CIDI)  100% | Median: 30 (25-35) | 74.0% | NR | Alcohol: 76.0%  Cannabis: 81.0%  Heroin: 19.0%  Cocaine: 25.0%  Ecstasy: 27.0%;  NR | - Individual counselling during residential rehabilitation - No individual counselling during residential rehabilitation |
| **Coffin 2013** (16)  US; Department of Public Health  Non-industry | RCT  12 weeks  90 | NA | MUD (DSM-IV-TR; SCID)  100% | 38.7 (10.8) | 87.8% | Caucasian: 50.0% | NR  NR | - Aripiprazole - Placebo |
| **Coffin 2018** (17)  US; Department of Public Health  Non-industry funding and intervention supplied by industry | RCT  12 weeks  100 | gbMSM | MUD (DSM-IV-TR; SCID)  100% | 43.2 (8.5) | Cisgender male: 96.0%  Transgender male: 1.0%  Transgender female: 3.0% | Caucasian: 55.0% | NR^†^  NR^†^ | - Naltrexone - Placebo |
| **Coffin 2020** (18)  US; Outpatient  Non-industry | RCT  36 weeks  120 | gbMSM | MUD (DSM-IV-TR; SCID)  100% | 43.3 (9.8) | Cisgender male: 95.8%  Transgender female: 4.2% | Caucasian: 50.8% | NR  NR | - Mirtazapine - Placebo |
| **Colfax 2011** (19)  US; Department of Public Health  Non-industry | RCT  12 weeks  60 | gbMSM | MUD (DSM-IV-TR; SCID)  100% | 40.5 (9.0) | 100% | Caucasian: 62.0% | NR  NR | - Mirtazapine - Placebo |
| **Das 2010** (20)  US; NR  Non-industry | RCT  12 weeks  30 | gbMSM | MUD (DSM; SCID)  100% | 36.5 (NR) | 100% | Caucasian: 53.3% | Cannabis: 63.0%  Poppers: 43.0%  Club drugs: 40.0%;  NR | - Bupropion - Placebo |
| **Elkashef 2008** (21,22)  US; Outpatient substance abuse treatment clinics  Non-industry | RCT  16 weeks  156 | Treatment-seeking | MUD (DSM-IV)  100% | 36.0 (8.8) | 66.9% | Caucasian: 74.2% | NR  NR | - Bupropion - Placebo |
| **Elkashef 2012** (23,24)  US; Medical centers  Non-industry | RCT  13 weeks  140 | NA | MUD (DSM-IV; SCID)  100% | 38.0 (8.6) | 63.6% | Caucasian: 82.9% | NR  NR | - Topiramate - Placebo |
| **Farahzadi 2019** (25)  Iran; Outpatient substance use disorder clinic  No funding | RCT  12 weeks  86 | NA | MUD (DSM-IV-TR)  100% | 35.9 (8.4) | 100% | NR | NR^†^  NR^†^ | - Riluzole - Placebo |
| **Fard 2020** (26)  Iran; Addiction treatment clinics  Non-industry funding | RCT  24 weeks  40 | Treatment-seeking, methamphetamine use at least 20 out of 28 days | MUD (DSM-5)  100% | 31.0 (7.9) | 100% | NR | NR  NR^†^ | - Modafinil - CBT |
| **Galloway 1996** (27)  US; Outpatient free drug detoxification and rehabilitation clinic  Non-industry | RCT  26 weeks  32 | NA | MUD (DSM-III-R)  100% | 33.0 (NR) | 91.0% | Caucasian: 78.1% | Alcohol: 31.3% Cannabis: 28.1% Heroin: 18.8% Cocaine: 6.3%;  NR^†^ | - Imipramine HCl 150 mg - Imipramine HCl 10 mg |
| **Ghasemi 2014** (28)  Iran; Substance abuse treatment centers  Non-industry | RCT  12 weeks  190 | Admitted to substance abuse treatment facilities, in recovery phase, and detoxified | MUD (NR)  NR | 31.1 (8.3) | 75.3% | NR | NR  NR | - Educational intervention - Treatment as usual |
| **Heinzerling 2006** (29)  US; Outpatient clinic  Non-industry | RCT  20 weeks  88 | Treatment-seeking | MUD (DSM-IV; SCID)  100% | 32.1 (7.8) | 69.3% | Caucasian: 59.1% | NR  NR^†^ | - Baclofen - Gabapentin - Placebo |
| **Heinzerling 2010** (30)  US; Clinical research sites  NR | RCT  16 weeks  71 | Treatment-seeking | MUD (DSM-IV-TR; SCID)  100% | 38.4 (10.5) | 70.4% | Caucasian: 50.7% | NR  NR^†^ | - Modafinil - Placebo |
| **Heinzerling 2014** (31)  US; Outpatient clinical research center  Non-industry | RCT  16 weeks  84 | Treatment-seeking, non-daily methamphetamine users (i.e., used on ≤29 of the 30 days prior to consent) | MUD (DSM-IV-TR; SCID)  100% | 38.3 (10.1) | 81.0% | Hispanic: 41.7% | NR  NR^†^ | - Bupropion - Placebo |
| **Heinzerling 2020** (32)  US; Outpatient clinic  Non-industry funding and intervention supplied by industry | RCT  16 weeks  125 | Treatment-seeking | MUD (DSM-IV-TR; SCID)  100% | 39.4 (10.0) | 73.6% | Caucasian: 47.2% | NR  NR^†^ | - Ibudilast - Placebo |
| **Kamp 2019** (33,34)  Germany; Inpatient rehabilitation centers  Non-industry | Cohort  78 weeks  108 enrolled (57 provided baseline data) | Admitted to inpatient rehabilitation centers | MUD (ICD-10 Diagnosis F15.2)  100% | 32.3 (8.0) | 78.9% | NR | NR  NR | - Conventional group therapy + 10 hours group therapy focusing on stimulant use - Conventional group therapy |
| **Kheirabadi 2016** (35)*******  Iran; Addiction treatment center  Non-industry | RCT  28 weeks  54 | NA | MUD (NR)  NR | 30.7 (5.4) | 100% | NR | NR  NR^†^ | - Valproate - Placebo |
| **Kheirabadi 2021** (36)  Iran; Addiction treatment center  Non-industry funding | RCT  24 weeks  40 | NA | MUD (DSM-5)  100% | 32.3 (7.0) | 75% | NR | NR  NR^†^ | - Buprenorphine - Placebo |
| **Ling 2012** (37)  US; Inpatient and outpatient substance abuse treatment clinics  Industry funded | RCT  15 weeks  120 | Treatment-seeking, methamphetamine use on at least 4 of the last 30 days | MUD (DSM-IV-TR or SCID-IV-TR)  100% | 38.4 (8.4) | 77.5% | Caucasian: 60.4% | NR  NR^†^ | - PROMETA TM protocol (flumazenil + gabapentin) - Placebo |
| **Ling 2014** (38)  US; Clinics  Non-industry funding and intervention supplied by industry | RCT  14 weeks  110 | NA | MUD (DSM-IV-TR)  100% | 39.1 (10.1) | 81.8% | Caucasian: 60.0% | NR  NR | - Methylphenidate - Placebo |
| **Longo 2009** (39)  Australia; Specialist drug and alcohol treatment services  Non-industry | RCT  26 weeks  49 | Methamphetamine use 3 or more days per week in previous 12 months | MUD (DSM-IV)  100% | 31.9 (5.1) | 61.0% | NR | NR  NR^†^ | - Dexamphetamine - Placebo |
| **McKetin 2021** (40)  Australia; NR  Non-industry funding and intervention supplied by industry | RCT  12 weeks  153 | Treatment-seeking | MUD (DSM-IV and Composite International Diagnostic Interview Version 3.0)  100% | 37.7 (8.1) | 59.0% | NR | Tobacco: 83.0%  Alcohol: 53.0%  Cannabis: 40.0%  Heroin: 4.0%  Other opioids: 8.0%  Cocaine: 4.0%  Ecstasy: 3.0% Hallucinogens: 3.0%  Inhalants: 9.0%;  NR | - N-acetylcysteine - Placebo |
| **Mimiaga 2019** (41)  US; Health institute  NR | RCT  26 weeks  41 | gbMSM | MUD (DSM-IV)  100% | 39.8 (11.6) | 100% | Caucasian: 78.0% | NR  NR | - Behavioural activation + Sexual risk reduction counselling - Sexual risk reduction counselling |
| **Noroozi 2020** (42)  Iran; Outpatient specialized drug treatment center  Non-industry funding | RCT  12 weeks  62 | Treatment-seeking | MUD (DSM-IV-TR)  100% | 32.1 (3.1) | 100% | NR | NR  NR^†^ | - Methylphenidate - Placebo |
| **Perngparn 2011** (43)  Thailand; Inpatient and outpatient drug dependent treatment centers  NR | Cohort  43 weeks  135 | NA | MUD (NR)  NR | 23.7 (5.7) | 100% | Thai: 98.5% | NR  NR | - Inpatient residential rehabilitation treatment (FAST model) - Outpatient MM treatment |
| **Polcin 2014** (44)  **Korcha 2014** (45)  US; Outpatient substance abuse treatment facility  Non-industry | RCT  26 weeks  217 | NA | MUD (DSM-IV)  100% | 38.0 (NR) | 50.7% | Caucasian: 67.3% | 75% with some level of alcohol problem;  NR | - Intensive MI - Standard MI |
|  | RCT  26 weeks  163 | Concurrent alcohol problem (alcohol ASI score >0 or reported ≥1 DSM-IV alcohol dependence symptom in last 12 months) | MUD (DSM-IV)  100% | 37.7 (10.4) | 46.6% | Caucasian: 67.5% | 100% concurrent alcohol problem;  DSM dependence other than MA: 58.9% |  |
| **Rawson 2004** (46–48)  US; Outpatient treatment sites  Non-industry | RCT  156 weeks  978 | Treatment-seeking | MUD (DSM-IV)  100% | 32.8 (NR) | 45.0% | Caucasian: 60.0% | NR  NR | - MM - Treatment as usual (best available option) |
| **Reback 2018** (49)  US; Methamphetamine abuse outpatient treatment program  Non-industry | RCT and matched historical control group  12 weeks  34 randomized, 102 matched historical controls | Treatment-seeking  gbMSM | Methamphetamine use in previous 12 months  NR | RCT: 40.6 (9.3);  Historical matches: 40.4 (8.6) | 100% | Caucasian, RCT: 35.3%;  Caucasian, Historical matches: 35.3% | NR  NR | - Counselling + Mobile app-based EMA + Web-based visualization dashboard - Mobile app-based EMA + Web-based dashboard - Matched historical control group |
| **Reback 2019** (50)  US; Community research center  Non-industry | RCT  39 weeks  286 | Not treatment-seeking gbMSM | Methamphetamine use in previous three months  89.2% (DSM-5; SCID MINI) | 41.5 (10.9)* | 100%* | African-American: 43.7%* | NR  NR | - Interactive TM with peer health educators + Theory-based, gay-specific TM transmitted by automation + Weekly self-monitoring text-based assessments - Theory-based, gay-specific, TM transmitted by automation + Weekly self-monitoring text-based assessments - Weekly self-monitoring text-based assessments |
| **Roll 2006** (51)  US; NR  Non-industry | RCT  12 weeks  18 | NA | MUD (DSM-IV)  100% | 32.0 (NR) | 66.7% | Caucasian: 77.8% | NR  NR | - CM (escalating with reset) - CM (escalating without reset) |
| **Roll 2006** (52)  US; Research clinic  NR | RCT  12 weeks  83 | Treatment-seeking | MUD (NR)  NR | 31.4 (7.3) | 60.2% | Caucasian: 62.7% | NR  NR | - Schedule 1 CM (flat magnitude of reinforcement schedule, no bonuses or resets) - Schedule 2 CM (slowly escalating magnitude of reinforcement, large bonuses and no resets) - Schedule 3 CM (high initial magnitude of reinforcement with slow escalation of voucher magnitude, no bonuses or resets) - Schedule 4 CM (high initial magnitude of reinforcement that decreased rapidly, moderate bonuses and no resets) - Schedule 5 CM (low initial magnitude of reinforcement with moderate escalation, moderate bonuses, and resets) |
| **Roll 2006** (53)  US; Community-based methadone maintenance drug abuse treatment clinics  Non-industry | RCT  26 weeks  402 randomized (subgroup of 113 with MUD) | Receiving methadone treatment | Stimulant abuse (DSM-IV)  28.1% of the 402 randomized (data reported for subgroup of participants with MUD) | 30.6 (8.1)** | 47.8%** | Caucasian: 69.0%** | Alcohol: 0%  Cannabis: 15.0%  Opioid: 5.3%**;  Alcohol: 22.1%  Cannabis: 25.7%  Opioid: 7.1%** | - CM + Treatment as usual - Treatment as usual |
| **Roll 2013** (54)  US; Outpatient clinic  Non-industry | RCT  52 weeks  118 | Treatment-seeking | MUD (DSM-IV)  100% | 32.0 (9.5) | 55.1% | Caucasian: 56.8% | NR  NR | - CM (1 month) + Standard psychosocial treatment - CM (2 month) + Standard psychosocial treatment - CM (4 month) + Standard psychosocial treatment - Standard psychosocial treatment |
| **Salehi 2015** (55)*******  Iran; Addiction treatment center  Non-industry | RCT  28 weeks  54 | NA | MUD (NR)  NR | 30.5 (5.4) | 100% | NR | NR  NR^†^ | - Buprenorphine - Placebo |
| **Shearer 2009** (56,57)  Australia; A primary health care center and a specialist drug and alcohol service  Non-industry funding and intervention supplied by industry | RCT  22 weeks  80 | Regular (i.e., 2–3 days or more) methamphetamine use per week | MUD (DSM-IV; CIDI)  100% | 36.0 (8.1) | 62.5% | NR | Alcohol (>6 standard drinks daily): 6.7%  Current opioid treatment: 12.5%;  NR | - Modafinil - Placebo |
| **Shoptaw 2005** (58–60)  US; Research clinic  Non-industry | RCT  52 weeks  162 | Treatment-seeking gbMSM | MUD (DSM-IV; SCID)  100% | 36.6 (6.4) | 100% | Caucasian: 77.2% | NR  NR | - CM (voucher-based reinforcement) - Gay and bisexual men-specific CBT - CBT - CBT + CM |
| **Shoptaw 2006** (61,62)  US; Clinical research site  Non-industry funding and intervention supplied by industry | RCT  14 weeks  229 | Treatment-seeking | MUD (DSM-IV, SCID)  100% | 33.1 (7.1) | 61.6% | Caucasian: 74.2% | NR  NR^†^ | - Sertraline - Placebo - Sertraline + CM - Placebo + CM |
| **Shoptaw 2008** (63)  US; Community clinics  Non-industry | RCT  52 weeks  128 (98 methamphet-amine abusers) | Treatment-seeking, gbMSM | Stimulant and/or alcohol abuse (NR)  NR | 37.1 (7.7)* | 100%* | Caucasian: 64.8%* | NR  NR^†^ | - Gay-specific CBT - Gay social support therapy |
| **Shoptaw 2008** (64,65)  US; Clinical research sites  Non-industry | RCT  12 weeks  73 | Treatment-seeking | MUD (DSM-IV-TR; SCID)  100% | 34.6 (10.2) | 64.4% | Caucasian: 56.2% | NR  NR^†^ | - Bupropion - Placebo |
| **Smout 2010** (66)  Australia; NR  Non-industry | RCT  24 weeks  104 | Treatment-seeking, methamphetamine use at least 2 days per week in the past 3 months | MUD (DSM-IV; MINI)  100% | 30.9 (6.5) | 60.0% | NR | NR  NR | - ACT - CBT |
| **Sorsdahl 2021** (67)  South Africa; NR  Non-industry funding | RCT  12 weeks  60 | NR | MUD (DSM-IV; MINI)  100% | 31.0 (6.5) | 53.3% | Caucasian: 3.3%  Black: 8.3%  Coloured: 88.3% | NR  NR | - Blended imaginal desensitization plus MI - Treatment as usual |
| **Trivedi 2021** (68)  US; Outpatient clinics  Non-industry funding and intervention supplied by industry | RCT (sequential parallel comparison design)  12 weeks (two stages consisting of 6 weeks each)  403 | Treatment-seeking, moderate or severe MUD, methamphetamine use on at least 18 of the 30 days before consent | MUD (DSM-5)  100% | 41.0 (10.1) | 68.7% | Caucasian: 71.2% | NR^†^;  Alcohol: 32.1%  Cannabis: 36.5%  Opioid: 7.3%  Cocaine: 8.5% | - Naltrexone + Bupropion - Placebo |
| **Wang 2019** (69)  China; Outpatient clinic  Non-industry | RCT  12 weeks  80 | Treatment-seeking, completed inpatient methamphetamine detoxification and discharged from hospital, history of methamphetamine-associated psychosis but without current psychotic symptoms | MUD (DSM-IV)  100% | 30.8 (7.0) | 88.8% | NR | NR;  Alcohol: 10.0%^†^ | - Paliperidone - Placebo |

Abbreviations: ACT = acceptance and commitment therapy; CBT = cognitive behavioural therapy; CIDI = Composite International Diagnostic Interview; CM = contingency management; EMA = ecological momentary assessments; gbMSM = gay and bisexual men who have sex with men; MATES = Methamphetamine Treatment Evaluation Study; MI = motivational interviewing; MINI = Mini-International Neuropsychiatric Interview; MM = Matrix Model; MUD = methamphetamine use disorder; SCID = Structured Clinical Interview; TM = text messages

**^¥^**Methamphetamine use disorder, dependence, or abuse

^Ɏ^Age is reported as mean (SD) unless otherwise specified

**^§^**Alcohol, cannabis and illicit substances (e.g., opioids, crack/cocaine)

^†^Exclusion of participants using or dependent on some or all other non-methamphetamine substances (substances varied across studies)

^‡^HIV testing was not assessed in the study; 9/32 had known HIV+ status.

*Baseline data reported for all participants (i.e., across all substances and not specific to those with methamphetamine use disorder or abuse)

**Baseline data reported for the subgroup of participants with MUD

***These studies are produced by the same authorship team and likely examine the same study participants.

**Reference List**

1. Abdoli N, Farnia V, Salemi S, Tatari F, Juibari TA, Alikhani M, et al. Efficacy of the Marlatt cognitive-behavioral model on decreasing relapse and craving in women with methamphetamine dependence: A clinical trial. Journal of Substance Use. 2019 Mar 4;24(2):229–32.

2. Abdoli N, Farnia V, Radmehr F, Alikhani M, Moradinazar M, Khodamoradi M, et al. The effect of self-compassion training on craving and self-efficacy in female patients with methamphetamine dependence: a one-year follow-up. Journal of Substance Use. 2021 Sep 3;26(5):491–6.

3. Amiri Z, Mirzaee B, Sabet M. Evaluating the efficacy of Regulated 12-Session Matrix Model in reducing susceptibility in methamphetamine-dependent individuals. International Journal of Medical Research & Health Sciences. 2016;5(2):77–85.

4. Anderson AL, Li S-H, Biswas K, McSherry F, Holmes T, Iturriaga E, et al. Modafinil for the treatment of methamphetamine dependence. Drug Alcohol Depend. 2012 Jan 1;120(1–3):135–41.

5. Anderson AL, Li S-H, Markova D, Holmes TH, Chiang N, Kahn R, et al. Bupropion for the treatment of methamphetamine dependence in non-daily users: a randomized, double-blind, placebo-controlled trial. Drug Alcohol Depend. 2015 May 1;150:170–4.

6. Aryan N, Banafshe HR, Farnia V, Shakeri J, Alikhani M, Rahimi H, et al. The therapeutic effects of methylphenidate and matrix-methylphenidate on addiction severity, craving, relapse and mental health in the methamphetamine use disorder. Subst Abuse Treat Prev Policy. 2020 Sep 25;15(1):72.

7. Baker A, Lee NK, Claire M, Lewin TJ, Grant T, Pohlman S, et al. Brief cognitive behavioural interventions for regular amphetamine users: a step in the right direction. Addiction. 2005 Mar;100(3):367–78.

8. Kay-Lambkin FJ, Baker AL, Lee NM, Jenner L, Lewin TJ. The influence of depression on treatment for methamphetamine use. Med J Aust. 2011 Aug 1;195(3):S38-43.

9. Lee NK, Pohlman S, Baker A, Ferris J, Kay-Lambkin F. It’s the thought that counts: craving metacognitions and their role in abstinence from methamphetamine use. J Subst Abuse Treat. 2010 Apr;38(3):245–50.

10. Briones M, Shoptaw S, Cook R, Worley M, Swanson A-N, Moody DE, et al. Varenicline treatment for methamphetamine dependence: A randomized, double-blind phase II clinical trial. Drug Alcohol Depend. 2018 Aug 1;189:30–6.

11. Brown ES, Gabrielson B. A randomized, double-blind, placebo-controlled trial of citicoline for bipolar and unipolar depression and methamphetamine dependence. J Affect Disord. 2012 Dec 20;143(1–3):257–60.

12. Chudzynski J, Roll JM, McPherson S, Cameron JM, Howell DN. Reinforcement Schedule Effects on Long-Term Behavior Change. Psychol Rec. 2015 Jun 1;65(2):347–53.

13. Ciketic S, McKetin R, Doran CM, Najman JM, Veerman JL, Hayatbakhsh RM. Health-related quality of life (HRQL) among methamphetamine users in treatment. Mental Health and Substance Use. 2013 Aug;6(3):250–61.

14. McKetin R, Najman JM, Baker AL, Lubman DI, Dawe S, Ali R, et al. Evaluating the impact of community-based treatment options on methamphetamine use: findings from the Methamphetamine Treatment Evaluation Study (MATES). Addiction. 2012 Nov;107(11):1998–2008.

15. McKetin R, Kothe A, Baker AL, Lee NK, Ross J, Lubman DI. Predicting abstinence from methamphetamine use after residential rehabilitation: Findings from the Methamphetamine Treatment Evaluation Study. Drug Alcohol Rev. 2018 Jan;37(1):70–8.

16. Coffin PO, Santos G-M, Das M, Santos DM, Huffaker S, Matheson T, et al. Aripiprazole for the treatment of methamphetamine dependence: a randomized, double-blind, placebo-controlled trial. Addiction. 2013 Apr;108(4):751–61.

17. Coffin PO, Santos G-M, Hern J, Vittinghoff E, Santos D, Matheson T, et al. Extended-release naltrexone for methamphetamine dependence among men who have sex with men: a randomized placebo-controlled trial. Addiction. 2018 Feb;113(2):268–78.

18. Coffin PO, Santos G-M, Hern J, Vittinghoff E, Walker JE, Matheson T, et al. Effects of Mirtazapine for Methamphetamine Use Disorder Among Cisgender Men and Transgender Women Who Have Sex With Men: A Placebo-Controlled Randomized Clinical Trial. JAMA Psychiatry. 2020 Mar 1;77(3):246–55.

19. Colfax GN, Santos G-M, Das M, Santos DM, Matheson T, Gasper J, et al. Mirtazapine to reduce methamphetamine use: a randomized controlled trial. Arch Gen Psychiatry. 2011 Nov;68(11):1168–75.

20. Das M, Santos D, Matheson T, Santos G-M, Chu P, Vittinghoff E, et al. Feasibility and acceptability of a phase II randomized pharmacologic intervention for methamphetamine dependence in high-risk men who have sex with men. AIDS. 2010 Apr 24;24(7):991–1000.

21. Elkashef AM, Rawson RA, Anderson AL, Li S-H, Holmes T, Smith EV, et al. Bupropion for the treatment of methamphetamine dependence. Neuropsychopharmacology. 2008 Apr;33(5):1162–70.

22. McCann DJ, Li S-H. A novel, nonbinary evaluation of success and failure reveals bupropion efficacy versus methamphetamine dependence: reanalysis of a multisite trial. CNS Neurosci Ther. 2012 May;18(5):414–8.

23. Elkashef A, Kahn R, Yu E, Iturriaga E, Li S-H, Anderson A, et al. Topiramate for the treatment of methamphetamine addiction: a multi-center placebo-controlled trial. Addiction. 2012 Jul;107(7):1297–306.

24. Ma JZ, Johnson BA, Yu E, Weiss D, McSherry F, Saadvandi J, et al. Fine-grain analysis of the treatment effect of topiramate on methamphetamine addiction with latent variable analysis. Drug Alcohol Depend. 2013 Jun 1;130(1–3):45–51.

25. Farahzadi M-H, Moazen-Zadeh E, Razaghi E, Zarrindast M-R, Bidaki R, Akhondzadeh S. Riluzole for treatment of men with methamphetamine dependence: A randomized, double-blind, placebo-controlled clinical trial. J Psychopharmacol. 2019 Mar;33(3):305–15.

26. Fard MT, Mansouri SS, Jafari A, Vousooghi N. Role of modafinil in the treatment of patients with methamphetamine dependence; An update on randomized, controlled clinical trial. Trop J Pharm Res. 2020 Nov 26;19(10):2179–85.

27. Galloway GP, Newmeyer J, Knapp T, Stalcup SA, Smith D. A controlled trial of imipramine for the treatment of methamphetamine dependence. J Subst Abuse Treat. 1996 Dec;13(6):493–7.

28. Ghasemi A, Estebsari F, Bastaminia A, Jamshidi E, Dastoorpoor M. Effects of Educational Intervention on Health-Promoting Lifestyle and Health-Related Life quality of Methamphetamine Users and Their Families: a Randomized Clinical Trial. Iran Red Crescent Med J. 2014 Nov;16(11):e20024.

29. Heinzerling KG, Shoptaw S, Peck JA, Yang X, Liu J, Roll J, et al. Randomized, placebo-controlled trial of baclofen and gabapentin for the treatment of methamphetamine dependence. Drug Alcohol Depend. 2006 Dec 1;85(3):177–84.

30. Heinzerling KG, Swanson A-N, Kim S, Cederblom L, Moe A, Ling W, et al. Randomized, double-blind, placebo-controlled trial of modafinil for the treatment of methamphetamine dependence. Drug Alcohol Depend. 2010 Jun 1;109(1–3):20–9.

31. Heinzerling KG, Swanson A-N, Hall TM, Yi Y, Wu Y, Shoptaw SJ. Randomized, placebo-controlled trial of bupropion in methamphetamine-dependent participants with less than daily methamphetamine use. Addiction. 2014 Nov;109(11):1878–86.

32. Heinzerling KG, Briones M, Thames AD, Hinkin CH, Zhu T, Wu YN, et al. Randomized, Placebo-Controlled Trial of Targeting Neuroinflammation with Ibudilast to Treat Methamphetamine Use Disorder. J Neuroimmune Pharmacol. 2020 Jun;15(2):238–48.

33. Kamp F, Proebstl L, Hager L, Schreiber A, Riebschläger M, Neumann S, et al. Effectiveness of methamphetamine abuse treatment: Predictors of treatment completion and comparison of two residential treatment programs. Drug Alcohol Depend. 2019 Aug 1;201:8–15.

34. Kamp F, Hager L, Proebstl L, Schreiber A, Riebschläger M, Neumann S, et al. 12- and 18-month follow-up after residential treatment of methamphetamine dependence: Influence of treatment drop-out and different treatment concepts. J Psychiatr Res. 2020 Oct;129:103–10.

35. Kheirabadi GR, Ghavami M, Maracy MR, Salehi M, Sharbafchi MR. Effect of add-on valproate on craving in methamphetamine depended patients: A randomized trial. Adv Biomed Res. 2016;5:149.

36. Kheirabadi GR, Bahrami M, Shariat A, Tarrahi M. The Effect of Add-on Buprenorphine to Matrix Program in Reduction of Craving and Relapse Among People With Methamphetamine Use Disorder: A Randomized Controlled Trial. J Clin Psychopharmacol. 2021 Feb 1;41(1):45–8.

37. Ling W, Shoptaw S, Hillhouse M, Bholat MA, Charuvastra C, Heinzerling K, et al. Double-blind placebo-controlled evaluation of the PROMETA^TM^ protocol for methamphetamine dependence. Addiction. 2012 Feb;107(2):361–9.

38. Ling W, Chang L, Hillhouse M, Ang A, Striebel J, Jenkins J, et al. Sustained-release methylphenidate in a randomized trial of treatment of methamphetamine use disorder. Addiction. 2014 Sep;109(9):1489–500.

39. Longo M, Wickes W, Smout M, Harrison S, Cahill S, White JM. Randomized controlled trial of dexamphetamine maintenance for the treatment of methamphetamine dependence. Addiction. 2010 Jan;105(1):146–54.

40. McKetin R, Dean OM, Turner A, Kelly PJ, Quinn B, Lubman DI, et al. N-acetylcysteine (NAC) for methamphetamine dependence: A randomised controlled trial. EClinicalMedicine. 2021 Aug;38:101005.

41. Mimiaga MJ, Pantalone DW, Biello KB, Hughto JMW, Frank J, O’Cleirigh C, et al. An initial randomized controlled trial of behavioral activation for treatment of concurrent crystal methamphetamine dependence and sexual risk for HIV acquisition among men who have sex with men. AIDS Care. 2019 Sep;31(9):1083–95.

42. Noroozi A, Motevalian SA, Zarrindast M-R, Alaghband-Rad J, Akhondzadeh S. Adding extended-release methylphenidate to psychological intervention for treatment of methamphetamine dependence: A double-blind randomized controlled trial. Med J Islam Repub Iran. 2020;34:137.

43. Perngparn U, Limanonda B, Aramrattana A, Pilley C, Areesantichai C, Taneepanichskul S. Methamphetamine dependence treatment rehabilitation in Thailand: a model assessment. J Med Assoc Thai. 2011 Jan;94(1):110–7.

44. Polcin DL, Bond J, Korcha R, Nayak MB, Galloway GP, Evans K. Randomized trial of intensive motivational interviewing for methamphetamine dependence. J Addict Dis. 2014;33(3):253–65.

45. Korcha RA, Polcin DL, Evans K, Bond JC, Galloway GP. Intensive motivational interviewing for women with concurrent alcohol problems and methamphetamine dependence. J Subst Abuse Treat. 2014 Feb;46(2):113–9.

46. Rawson RA, Marinelli-Casey P, Anglin MD, Dickow A, Frazier Y, Gallagher C, et al. A multi-site comparison of psychosocial approaches for the treatment of methamphetamine dependence. Addiction. 2004 Jun;99(6):708–17.

47. Rawson RA, Gonzales R, Pearce V, Ang A, Marinelli-Casey P, Brummer J, et al. Methamphetamine dependence and human immunodeficiency virus risk behavior. J Subst Abuse Treat. 2008 Oct;35(3):279–84.

48. Rawson RA, Gonzales R, Greenwell L, Chalk M. Process-of-care measures as predictors of client outcome among a methamphetamine-dependent sample at 12- and 36-month follow-ups. J Psychoactive Drugs. 2012 Oct;44(4):342–9.

49. Reback CJ, Rünger D, Fletcher JB, Swendeman D. Ecological momentary assessments for self-monitoring and counseling to optimize methamphetamine treatment and sexual risk reduction outcomes among gay and bisexual men. J Subst Abuse Treat. 2018 Sep;92:17–26.

50. Reback CJ, Fletcher JB, Swendeman DA, Metzner M. Theory-Based Text-Messaging to Reduce Methamphetamine Use and HIV Sexual Risk Behaviors Among Men Who Have Sex with Men: Automated Unidirectional Delivery Outperforms Bidirectional Peer Interactive Delivery. AIDS Behav. 2019 Jan;23(1):37–47.

51. Roll JM, Shoptaw S. Contingency management: schedule effects. Psychiatry Res. 2006 Sep 30;144(1):91–3.

52. Roll JM, Huber A, Sodano R, Chudzynski JE, Moynier E, Shoptaw S. A Comparison of Five Reinforcement Schedules for use in Contingency Management-Based Treatment of Methamphetamine Abuse. Psychol Rec. 2006 Jan;56(1):67–81.

53. Roll JM, Petry NM, Stitzer ML, Brecht ML, Peirce JM, McCann MJ, et al. Contingency management for the treatment of methamphetamine use disorders. Am J Psychiatry. 2006 Nov;163(11):1993–9.

54. Roll JM, Chudzynski J, Cameron JM, Howell DN, McPherson S. Duration effects in contingency management treatment of methamphetamine disorders. Addict Behav. 2013 Sep;38(9):2455–62.

55. Salehi M, Emadossadat A, Kheirabadi GR, Maracy MR, Sharbafchi MR. The Effect of Buprenorphine on Methamphetamine Cravings. J Clin Psychopharmacol. 2015 Dec;35(6):724–7.

56. Shearer J, Darke S, Rodgers C, Slade T, van Beek I, Lewis J, et al. A double-blind, placebo-controlled trial of modafinil (200 mg/day) for methamphetamine dependence. Addiction. 2009 Feb;104(2):224–33.

57. Shearer J, Shanahan M, Darke S, Rodgers C, van Beek I, McKetin R, et al. A cost-effectiveness analysis of modafinil therapy for psychostimulant dependence. Drug Alcohol Rev. 2010 May;29(3):235–42.

58. Shoptaw S, Reback CJ, Peck JA, Yang X, Rotheram-Fuller E, Larkins S, et al. Behavioral treatment approaches for methamphetamine dependence and HIV-related sexual risk behaviors among urban gay and bisexual men. Drug Alcohol Depend. 2005 May 9;78(2):125–34.

59. Peck JA, Reback CJ, Yang X, Rotheram-Fuller E, Shoptaw S. Sustained reductions in drug use and depression symptoms from treatment for drug abuse in methamphetamine-dependent gay and bisexual men. J Urban Health. 2005 Mar;82(1 Suppl 1):i100-108.

60. Jaffe A, Shoptaw S, Stein J, Reback CJ, Rotheram-Fuller E. Depression ratings, reported sexual risk behaviors, and methamphetamine use: latent growth curve models of positive change among gay and bisexual men in an outpatient treatment program. Exp Clin Psychopharmacol. 2007 Jun;15(3):301–7.

61. Shoptaw S, Huber A, Peck J, Yang X, Liu J, Jeff Dang null, et al. Randomized, placebo-controlled trial of sertraline and contingency management for the treatment of methamphetamine dependence. Drug Alcohol Depend. 2006 Oct 15;85(1):12–8.

62. Zorick T, Sugar CA, Hellemann G, Shoptaw S, London ED. Poor response to sertraline in methamphetamine dependence is associated with sustained craving for methamphetamine. Drug Alcohol Depend. 2011 Nov 1;118(2–3):500–3.

63. Shoptaw S, Reback CJ, Larkins S, Wang P-C, Rotheram-Fuller E, Dang J, et al. Outcomes using two tailored behavioral treatments for substance abuse in urban gay and bisexual men. J Subst Abuse Treat. 2008 Oct;35(3):285–93.

64. Shoptaw S, Heinzerling KG, Rotheram-Fuller E, Steward T, Wang J, Swanson A-N, et al. Randomized, placebo-controlled trial of bupropion for the treatment of methamphetamine dependence. Drug Alcohol Depend. 2008 Aug 1;96(3):222–32.

65. Brensilver M, Heinzerling KG, Swanson A-N, Telesca D, Furst BA, Shoptaw SJ. Cigarette smoking as a target for potentiating outcomes for methamphetamine abuse treatment. Drug Alcohol Rev. 2013 Jan;32(1):96–9.

66. Smout MF, Longo M, Harrison S, Minniti R, Wickes W, White JM. Psychosocial treatment for methamphetamine use disorders: a preliminary randomized controlled trial of cognitive behavior therapy and Acceptance and Commitment Therapy. Subst Abus. 2010 Apr;31(2):98–107.

67. Sorsdahl K, Stein DJ, Pasche S, Jacobs Y, Kader R, Odlaug B, et al. A novel brief treatment for methamphetamine use disorders in South Africa: a randomised feasibility trial. Addict Sci Clin Pract. 2021 Jan 7;16(1):3.

68. Trivedi MH, Walker R, Ling W, Dela Cruz A, Sharma G, Carmody T, et al. Bupropion and Naltrexone in Methamphetamine Use Disorder. N Engl J Med. 2021 Jan 14;384(2):140–53.

69. Wang G, Ma L, Liu X, Yang X, Zhang S, Yang Y, et al. Paliperidone Extended-Release Tablets for the Treatment of Methamphetamine Use Disorder in Chinese Patients After Acute Treatment: A Randomized, Double-Blind, Placebo-Controlled Exploratory Study. Front Psychiatry. 2019;10:656.
