# Supplementary material for: Psychosocial and pharmacologic interventions for problematic methamphetamine use: Findings from a scoping review of the literature
Source: PLoS One. 2023 Oct 11;18(10):e0292745. doi: 10.1371/journal.pone.0292745 (PMC10566716; doi:10.1371/journal.pone.0292745)
Supplement: S8 Text — (DOCX) [file pone.0292745.s008.docx]

# S8 Text. Bar plots of baseline population characteristics

## Figure A. Mean age across studies

Reported in 53 or 54 included studies.

## Figure B. Male born % across studies

Two studies were female only.

## Figure C. Reported as Caucasian across studies

## Figure D. Lifetime duration of methamphetamine use (years), mean/median

## Figure E. Number of days of methamphetamine use in the last 14-30 days, mean/median
