# Supplementary material for: Psychosocial and pharmacologic interventions for problematic methamphetamine use: Findings from a scoping review of the literature
Source: PLoS One. 2023 Oct 11;18(10):e0292745. doi: 10.1371/journal.pone.0292745 (PMC10566716; doi:10.1371/journal.pone.0292745)
Supplement: S10 Text — (DOCX) [file pone.0292745.s010.docx]

**S10 Text. Intervention characteristics**

1. **Pharmacotherapy treatments**

| **Author Year {refID}** | **Total daily dose** | **Intervention schedule** | **Route of administration;**  **Formulation** | **Treatment duration (weeks)** | **Co-interventions** | **Comparison** |  |
| --- | --- | --- | --- | --- | --- | --- | --- |
| **Antidepressants** | | | | | | | |
| **Bupropion** | | | | | | | |
| **Das 2010** (1) | 300 mg/day | 150 mg once a day (day 1-7); 150 mg BID (taken in the morning on weeks 2-12); 150 mg once a day (for 14 days after completion of the intervention) | oral;  XL | 12 | (1) Substance use counselling using CBT and MI techniques (weekly for 30-min)  (2) HIV risk-reduction counselling was repeated for HIV-negative participants at the final visit | Placebo |  |
| **Anderson 2015** (2) | 300 mg/day | 150 mg BID | oral;  sustained-release | 12 | Group cognitive-behavioral, relapse-prevention, manual-driven therapy three times weekly (90-min sessions) | Placebo |  |
| **Elkashef 2008** (3,4) | 300 mg/day | 150 mg once daily (days 1-3); 150 mg BID (for 11 weeks); dose reduced to 150 mg daily at end of treatment period (last 3 days) | oral;  sustained-release | 12 | Group standardized cognitive-behavioral therapy derived from the relapse prevention group component of the Matrix Model (90-minutes three times a week for 12 weeks) | Placebo |  |
| **Heinzerling 2014** (5) | 300 mg/day | 150 mg once a day (days 1-3); 150 mg BID (day 4 until the last three days of the treatment period); 150 mg once a day (the last three days of the treatment period) | oral;  sustained-release | 12 | Manual-based cognitive behavioral therapy (weekly for 12 weeks) | Placebo |  |
| **Shoptaw 2008** (6,7) | 300 mg/day | 150 mg once a day (days 1–3); 150 mg BID (day 4 to last 3 days of medication phase); 150 mg once a day (the last three days of week 12) | oral;  sustained-release | 12 | (1) SC consisting of weekly individual CBT for 12 weeks  (2) CM for 12 weeks: Participants received vouchers, redeemable for goods or services, for each urine sample that was negative for methamphetamine metabolites. Urine samples were submitted three times per week. Vouchers started at US$ 3.00 for the first metabolite-negative sample and increased in value by US$ 1.00 for each sample thereafter for the first 4 weeks of the study. Participants with methamphetamine-negative samples earned $15.00 from weeks 5 to 12. The value of the voucher was reset to $3.00 after relapse or a missed urine sample; the value was again reset to $15.00 after 3 methamphetamine-negative samples. Maximum cash value of vouchers was $537.00). | Placebo |  |
| **Mirtazapine** | | | | | | | |
| **Coffin 2020** (8) | 30 mg/day | 15 mg/day (week 1); 30 mg/day (week 2-24) | oral | 24 | (1) Substance use counselling using CBT and MI weekly for 24 weeks  (2) HIV counselling and testing every 12 weeks for individuals who initially tested negative for HIV | Placebo |  |
| **Colfax 2011** (9) | 30 mg/day | 15 mg once daily (week 1); 15 mg BID (weeks 2-12) | oral | 12 | (1) Weekly substance use counselling based on CBT and MI techniques (30-minute sessions)  (2) HIV risk reduction counselling repeated at 12-week visit. | Placebo |  |
| **Imipramine HCl** | | | | | | | |
| **Galloway 1996** (10) | 150 mg | 50 mg once a day (first week); 100 mg once a day (second week); 150 mg once a day (week three until the end of intervention period) | oral | 26 | (1) 3 to 5 hours of group counselling per week  (2) HIV risk-reduction education | Imipramine (10 mg/day) |  |
| **Antipsychotics** | | | | | | | |
| **Paliperidone** | | | | | | | |
| **Wang 2019** (11) | 3 mg/day | 3 mg once daily | oral;  extended-release | 12 | CBT counselling (30 minutes per week) | Placebo |  |
| **Aripiprazole** | | | | | | | |
| **Coffin 2013** (12) | 20 mg/day | 5 mg daily (week 1); 10 mg daily (week 2); 20 mg daily (week 3-12) | oral | 12 | (1) Weekly 30-minute substance use counselling using CBT and MI techniques  (2) HIV risk-reduction counselling was repeated for HIV- negative participants at the12-week visit | Placebo |  |
| **Cognitive enhancers** | | | | | | | |
| **Varenicline** | | | | | | | |
| **Briones 2018** (13) | 2 mg/day | 0.5 mg once a day (days 1–3); 0.5 mg BID (days 4–7); 1 mg BID (day 8 until completion of the medication phase) | oral;  over-encapsulated in a #1 size capsule with 25 mg riboflavin (daily total) | 9 | (1) SC program (one-hour weekly individual CBT sessions for 9-weeks)  (2) Comprehensive medication adherence counselling provided at every visit (three times per week)  (3) Inpatient detoxification and methamphetamine-abstinence initiation for four nights (day 8-11). Only 18 of the 52 participants in the trial received the inpatient stay intervention due to funding constraints; the inpatient stay was replaced with daily visits to the outpatient clinic to complete assessments during the second week of the study. | Placebo |  |
| **Modafinil** | | | | | | | |
| **Anderson 2012** (14) | 400mg/day | 400mg daily | oral | 12 | (1) Group standardized psychosocial therapy using the Matrix Institute's manual-guided CBT (90-minute sessions three times per week for 12 weeks)  (2) Referral for HIV counselling | Modafinil (200mg/day) |  |
| **Heinzerling 2010** (15) | 400 mg/day | 100 mg BID (taken in the morning on days 1-3); 100 mg QID (taken at one time in the morning on day 4 until the last three days of the trial); 200 mg per day (the last three days of the trial);  Permanent dose reduction to 200 mg/day allowed if AEs occurred. | oral | 12 | (1) SC of weekly individual CBT for 12 weeks  (2) CM during 2-week baseline period and 12- week medication phase: Participants received vouchers, redeemable for goods or services, for each urine sample that was negative for methamphetamine metabolites. Urine samples were submitted three times per week. Vouchers started at US $3.00 for the first metabolite-negative sample and increased in value by US$ 1.00 for each sample thereafter for the first 4 weeks of the study. Participants with methamphetamine-negative samples earned $15.00 from weeks 5 to 14. The value of the voucher was reset to $3.00 after relapse or a missed urine sample; the value was again reset to $15.00 after 3 methamphetamine-negative samples. Maximum cash value of vouchers was $537.00. | Placebo |  |
| **Shearer 2009** (16,17) | 200 mg/day | 200 mg/day (fixed dose) | oral | 10 | Brief four-session cognitive behavioural intervention developed specifically for methamphetamine users. | Placebo |  |
| **Fard 2020** (18) | 300 mg/day | 100mg/day (first day); 200mg (second day); 300mg (target dose) | oral | 12 | Standardized medication management (weekly, 15-20 minute session) consisting of intervention dose confirmation, side effects, cravings, and concomitant medication.  Unclear if control participants received standardized medication management. Participants in control group could receive medications other than modafinil. | CBT |  |
| **Gamma-aminobutyric acid agents** | | | | | | | |
| **Baclofen vs Gabapentin** | | | | | | | |
| **Heinzerling 2006** (19) | Baclofen: 60 mg/day | Baclofen: 10 mg TID (days 1–3); 20 mg TID (until week 16); dose decreased to 10 mg TID (last 3 days) | oral | 16 | Standard manual-driven psychosocial counselling program consisting of relapse prevention group sessions (90 minutes thrice-weekly for 16 weeks) | (1) Gabapentin (2400 mg/day)  [Treatment schedule: 400 mg TID (days 1–3); 800 mg TID (until week 16); dose decreased to 400 mg TID (last 3 days)];  (2) Placebo |  |
| **Valproate** | | | | | | | |
| **Kheirabadi 2016** (20) | 1000 mg/day | 250 mg daily (up to 10 days) then 1000 mg daily (remainder of treatment phase); dose tapered and discontinued over 10 days at end of treatment phase | oral | 16 | (1) Matrix program  (2) After the active treatment phase (16 weeks), patients were introduced to self-help groups and monitored regularly on a weekly basis over another 3 months. | Placebo |  |
| **Opioid antagonists** | | | | | | | |
| **Naltrexone** | | | | | | | |
| **Coffin 2018** (21) | 380 mg/month | 380 mg at 4-week intervals over 12 weeks (administered as 3 gluteal injections) | gluteal injections;  extended-release | 12 | (1) Substance use counselling using CBT and MI techniques (30-minute weekly)  (2) HIV risk-reduction counselling repeated for HIV- and HCV-negative participants at 12-week visits. | Placebo |  |
| **Psychostimulants** | | | | | | | |
| **Methylphenidate** | | | | | | | |
| **Ling 2014** (22) | 54 mg/day | 18 mg once daily (week 1); 36 mg once daily (week 2); 54 mg once daily (weeks 3-10) | oral;  sustained-release | 10 | (1) Placebo once daily (weeks 11-14)  (2) Motivational incentives twice weekly (participants earned draws for each MA-negative urine drug screen using a fishbowl method)  (3) Group CBT once weekly (1–1½ hour sessions). | Placebo |  |
| **Noroozi 2020** (23) | 60 mg/day | 20 mg/day (week 1); 20mg BID (week 2); 20mg TID (weeks 3-12) | oral;  extended-release | 12 | (1) Medication adherence and safety psychoeducation.  (2) Individual modified Matrix model treatment twice weekly (24 sessions total) consisting of "motivational enhancement, psychoeducational and cognitive behavioral treatment techniques". | Placebo |  |
| **Citicoline** | | | | | | | |
| **Brown 2012** (24) | 2000 mg/day | 500 mg once daily (week 1); 500 mg BID (week 2-3); 500 mg TID (week 4-5); 500 mg QID (week 6-12);  Dose decreased if needed due to side effects. | oral | 12 | CM model (drawing for a chance to win small prizes) for each of the urine samples provided. | Placebo |  |
| **Dexamphetamine** | | | | | | | |
| **Longo 2009** (25) | up to 110 mg/day | 20 mg once a day increased by 10 mg daily as required until stabilized or to a maximum of 110 mg/day (stabilization occurred over 2 weeks and the participants maintained on maximum dose of 110 mg/day for a maximum of 12 weeks). Medication tapered off over 1 month at end of treatment. | oral;  sustained-release | 14 (maximum 104 days: 14 days of stabilization and 90 days maintenance) | Standard psychotherapeutic care consisting of an introductory appointment plus cognitive behavioural model for amphetamine users (four sessions) | Placebo |  |
| **Other pharmacotherapies** | | | | | | | |
| **Topiramate** | | | | | | | |
| **Elkashef 2012** (26,27) | 200 mg/day or subject’s maximum tolerated dose | 25 mg/day then escalated over 35 days to 200 mg/day or to the subject’s maximum tolerated dose (dose maintained over weeks 6–12); at week 13, the dose was tapered to 100 mg/day for 3 days, 50 mg/day for 2 days, and then 25 mg/day for 2 days;  Dose decreased if needed due to side effects but had to be ≥50 mg/day to remain in the study. | oral | 13 | Weekly brief behavioral compliance enhancement treatment | Placebo |  |
| **Ibudilast** | | | | | | | |
| **Heinzerling 2020** (28) | 100 mg/day | 20 mg BID (day 1 to 3); 50 mg BID (day 4 to end of treatment) | oral | 12 | (1) 12.5 mg riboflavin twice daily (2) Medical Management counselling delivered weekly by study physician/nurse or counselor following a manual developed for the trial. | Placebo |  |
| **Riluzole** | | | | | | | |
| **Farahzadi 2019** (29) | 100 mg/day | 50 mg BID | oral | 12 | Weekly MM therapy sessions for 12 weeks | Placebo |  |
| **Buprenorphine** | | | | | | | |
| **Salehi 2015** (30) | 6 mg/day | 2 mg/day and then increased to 6 mg/day within 7 days; dose tapered and stopped over 10 days at end of treatment | sublingual | 16 | (1) Matrix program (daily)  (2) After the 16-week treatment phase, patients were introduced to the self-help groups and were regularly monitored every 2 weeks by a psychiatry resident for another 12 weeks. | Placebo |  |
| **Kheirabadi 2021** (31) | 4 mg/day | 2 mg/day (first 7 days) then 4 mg /day (6 weeks) then dose tapered and discontinued within 1 week. | sublingual | 8 | Matrix intervention for two months | Placebo |  |
| **PROMETA protocol: Flumazenil + Gabapentin** | | | | | | | |
| **Ling 2012** (32) | Flumazenil: 2 mg/day (on day of infusion)  Gabapentin: 1200 mg/day  Hydroxyzine: 50 mg/day | Flumazenil: 2 mg (days 1, 2, 3, 22 and 23)  Gabapentin: 300 mg (day 1); 600 mg (day 2); 900 mg (day 3); 1200 mg (day 4-37); down-titration (days 38-40)  Hydroxyzine: 50 mg dose before each flumazenil infusion (days 1, 2, 3, 22 and 23) and 50 mg take-home hydroxyzine to day 10 | Flumazenil: infusion  Gabapentin: oral  Hydroxyzine: oral | Flumazenil: Approximately 3 weeks (23 days)  Gabapentin: Approximately 6 weeks (40 days)  Hydroxyzine: Approximately 1.5 weeks (12 days) | CBT (1 session per week for 14 weeks) | Placebo (with active hydroxazine only) |  |
| **Naltrexone + Bupropion** | | | | | | | |
| **Trivedi 2021** (33) | Naltrexone: 380 mg every 3 weeks  Bupropion: 450 mg/day | Naltrexone: 380 mg every three weeks [on the day of randomization (or rerandomization) and in the third week of each stage];  Bupropion: escalating dose up to 450 mg/day (day 1-3); dose maintained at 450 mg/day (day 4 until the end of week 12); tapered over 4 days until discontinued | Naltrexone: intramuscular injection (extended-release)  Bupropion: oral (extended-release) | 12 (6 weeks per study stage) | Weekly counselling for reducing substance use provided by clinicians | Placebo |  |
| **N-acetylcysteine** | | | | | | | |
| **McKetin 2021** (34) | 2400 mg/day | 1200 mg BID | Oral | 12 | No co-interventions provided. Participants were allowed to receive treatments or seek support outside of the trial. Twenty-one percent of NAC participants received other drug treatment during the trial. | Placebo |  |

Abbreviations: BID = twice a day; CBT = cognitive behavioural therapy; HIV = human immunodeficiency virus; TID = three times a day; QID = four times a day; MI = motivational interviewing; MM = Matrix Model; SC = standard counselling

1. **Psychosocial treatments**

| **Author Year**  **Study arms** | **Total number of sessions** | **Format** | **Frequency per week;**  **Duration per session (min)** | **Duration (weeks)** | **Additional details** | **Co-interventions** |
| --- | --- | --- | --- | --- | --- | --- |
| **Chudzynski**  **2015** (35)   - Continuous CM - Intermittent predictable CM - Intermittent unpredictable CM - No treatment | 48 urine samples | Individual | Urine samples submitted:  3 days/week | 16 | *Continuous contingency management:* Participants received vouchers, redeemable for goods or services, for each methamphetamine-negative urine sample. Urine samples were submitted three times per week. Vouchers started at US$ 2.50 for the first methamphetamine-negative sample and increased in value by US$ 1.50 for each sample thereafter. For three consecutive methamphetamine-negative urine samples, a bonus voucher of US$ 10.00 was provided. For missed or methamphetamine-positive urine samples, no voucher was received, and a reset procedure was followed whereby the value of the next voucher for a methamphetamine-negative sample was reset to US$ 2.50. Maximum cash value of vouchers was $1155.00. | Group cognitive behavioral therapy based on the Matrix Model (three times per week for 16 weeks). |
|  | 48 urine samples | Individual | Urine samples submitted:  3 days/week | 16 | *Intermittent predictable contingency management:*  Participants received one voucher per week when they provided three consecutive methamphetamine-negative urine tests. The value of the vouchers increased with each three consecutive methamphetamine-negative samples provided; $22.00 for the first three metabolite negative samples, $35.50 for the next week of metabolite negative samples, $49.00 for the third week, and so on. For missed or methamphetamine-positive urine samples, a reset procedure was followed whereby the value of the next voucher following three consecutive methamphetamine-negative samples was reset to US$ 22.00. The maximum cash value of vouchers was $1155.00. |  |
|  | 48 urine samples | Individual | Urine samples submitted:  3 days/week | 16 | *Intermittent un­predictable contingency management:* Participants received one voucher per week of the same value and rate as the intermittent predictable condition. Participants received a voucher for $22.00 for the first three consecutive methamphetamine-negative urine samples. Thereafter, they received one voucher per week if all other urine tests since their last voucher were methamphetamine-negative. The voucher was given on a randomly selected day of the week unknown to participants until the urine sample was provided. For missed or methamphetamine-positive urine samples, a reset procedure was followed whereby the value of the next voucher following three consecutive methamphetamine-negative samples was reset to US$ 22.00. The maximum cash value of vouchers was $1155.00. |  |
|  | NA | NA | NA | 16 | *Standard condition:* Received no vouchers for the provision of methamphetamine-negative drug tests |  |
| **Roll 2006** (36)   - Escalating without reset CM - Escalating with reset CM | 36 urine samples | Individual | Urine samples submitted:  3 days/week | 12 | *Escalating without reset:* Participants received vouchers, redeemable for goods or services, for each urine sample that was negative for methamphetamine metabolites. Urine samples were submitted three times per week. Vouchers started at US$ 2.50 for the first metabolite-negative sample and increased in value by US$ 1.50 for each sample thereafter. The value of the voucher was not reset following relapse or a missed sample (i.e., did not change the level of escalation for the next urine sample that was negative for methamphetamine.) The total value of vouchers that could be earned was equal between the study groups. | Group cognitive behavioral therapy three times/week. |
|  | 36 urine samples | Individual | Urine samples submitted:  3 days/week | 12 | *Escalating with reset:* Participants received vouchers, redeemable for goods or services, for each urine sample that was negative for methamphetamine metabolites. Urine samples were submitted three times per week. Vouchers started at US$ 2.50 for the first metabolite-negative sample and increased in value by US$ 1.50 for each sample thereafter. The value of the voucher was reset to $2.50 following relapse or a missed sample. The total value of vouchers that could be earned was equal between the study groups. |  |
| **Roll 2006** (37)   - Schedule 1 CM: A flat magnitude of reinforcement schedule with no bonuses for continuous abstinence or resets for failure to abstain - Schedule 2 CM: Slowly escalating magnitude of reinforcement with large bonuses for blocks of abstinence and no resets for failure to abstain - Schedule 3 CM: High initial magnitude of reinforcement with slow escalation of voucher magnitude and no bonuses for continuous abstinence or resets for failure to abstain - Schedule 4 CM: High initial magnitude of reinforcement that decreased rapidly with moderate bonuses for blocks of abstinence and no resets for failure to abstain - Schedule 5 CM: Low initial magnitude of reinforcement, with moderate escalation, moderate bonuses for continuous abstinence and resets in voucher magnitude for failure to abstain | 24 urine samples | Individual | Urine samples submitted:  3 days/week | 8 | *Schedule 1 CM: A flat magnitude of reinforcement schedule with no bonuses for continuous abstinence or resets for failure to abstain:* "Each instance of abstinence during the first 8 weeks resulted in the delivery of a $25.00 voucher. Additionally, the provision of three consecutive methamphetamine negative urine tests resulted in the delivery of a $50.00 voucher. There were no reset contingencies. The total amount of reinforcement possible from this schedule was $1,000.00." No vouchers were provided if a metabolite-positive sample was submitted or if the participant failed to provide a urine sample. Vouchers were exchanged for goods and services approved by study/clinical staff. | Cognitive behavioral therapy group sessions based on Matrix Model (3 times/week). |
|  | 36 urine samples | Individual | Urine samples submitted:  3 days/week | 12 | *Schedule 2 CM: Slowly escalating magnitude of reinforcement with large bonuses for blocks of abstinence and no resets for failure to abstain:* "Each instance of abstinence during the first 6 weeks resulted in the delivery of a voucher that increased in value by $1.00 for consecutive instances of abstinence [initial voucher was $14.00]. Additionally, during the entire 12-week period, the provision of three consecutive methamphetamine negative urine tests resulted in the delivery of a $50.00 voucher. There were no reset contingencies. The total amount of reinforcement possible from this schedule was $1,005.00." No vouchers were provided if a metabolite-positive sample was submitted or if the participant failed to provide a urine sample. Vouchers were exchanged for goods and services approved by study/clinical staff. |  |
|  | 36 urine samples | Individual | Urine samples submitted:  3 days/week | 12 | *Schedule 3 CM: High initial magnitude of reinforcement with slow escalation of voucher magnitude and no bonuses for continuous abstinence or resets for failure to abstain:* "Each instance of abstinence during the 12-week period resulted in the delivery of a voucher, the value of which escalated by $1.00 for consecutive abstinences [initial voucher was $10.00]. There were no bonuses or reset contingencies. The total amount of reinforcement possible from this schedule was $990.00." No vouchers were provided if a metabolite-positive sample was submitted or if the participant failed to provide a urine sample. Vouchers were exchanged for goods and services approved by study/clinical staff. |  |
|  | 36 urine samples | Individual | Urine samples submitted:  3 days/week | 12 | *Schedule 4 CM: High initial magnitude of reinforcement that decreased rapidly with moderate bonuses for blocks of abstinence and no resets for failure to abstain:* "The magnitude of reinforcement started high [initial voucher was $75] and decreased by $5.00 for the first 3 weeks for each consecutive instance of abstinence. Following that all remaining abstinences earned vouchers worth $10.00. Throughout the entire 12-week period three consecutive instances of abstinence resulted in the delivery of a $20.00 bonus. There were no reset contingencies. The total amount of reinforcement available was $1,005.00."No vouchers were provided if a metabolite-positive sample was submitted or if the participant failed to provide a urine sample. Vouchers were exchanged for goods and services approved by study/clinical staff. |  |
|  | 36 urine samples | Individual | Urine samples submitted:  3 days/week | 12 | *Schedule 5 CM: Low initial magnitude of reinforcement, with moderate escalation, moderate bonuses for continuous abstinence and resets in voucher magnitude for failure to abstain:* “During the 12-week period each instance of abstinence resulted in the delivery of a reinforcer, which increased for consecutive abstinences by $1.25 [initial voucher was $2.50]. Additionally, each block of three consecutive abstinences resulted in the delivery of a $10.00 bonus. Finally, failure to abstain resulted in a [reset] in voucher value to the initial value from whence the escalation could begin again. The total amount of reinforcement available from this schedule was $997.50." |  |
| **Roll 2006** (38)   - CM + Treatment as usual - Treatment as usual | 24 urine samples | Individual | Urine samples submitted:  2 day/week | 12 | *CM + Treatment a usual:* Participants received contingency management in addition to the treatment as usual condition offered at the study site. Treatment as usual varied across study sites and consisted of Matrix model therapy in one site and a mix of cognitive behavior therapy and relapse prevention in the other two sites.  For the contingency management intervention, participants could earn the chance to win prizes each time they tested negative for the primary target drugs (methamphetamine, cocaine, amphetamine, and alcohol were considered the primary target drugs). Participants providing a metabolite-negative urine sample were eligible to draw between one and 12 chips from an opaque container containing 500 chips. Participants were eligible for one additional draw each week if all submitted samples were negative for the target drugs. A positive or missed urine sample resulted in a reset in the number of draws. Chips ranged in value from no monetary value to jumbo ($80-100). Fifty percent of chips were marked with "Good job" and had no monetary value; 41.8% were 'small' and worth $1.00-5.00; 8% were 'large' and worth $20, and 0.2% were 'jumbo' and worth $80-100. Participants exchanged their chips for prizes within each category. Participants were eligible for two bonus draws if they were also abstinent opioids and marijuana. A large prize was earned when participants first achieved two consecutive weeks of abstinence; this was to "offset the low rate of reinforcement early in the study when the number of draws was low". Maximum of 204 draws (~$400 in prizes) plus $20 for first two consecutive weeks of abstinence. | All sites encouraged participation in 12-step groups. |
|  | NR | NR | NR | NR | *Treatment as usual:* Varied across study sites and consisted of Matrix model therapy in one site and a mix of cognitive behavior therapy and relapse prevention in the other two sites. |  |
| **Roll 2013** (39)   - 1 month CM + Standard psychosocial treatment - 2 month CM + Standard psychosocial treatment - 4 month CM + Standard psychosocial treatment - Standard psychosocial treatment | Standard psychosocial intervention: 48,  CM: 12 urine samples | CM: individual,  Standard psychological treatment: group | Standard psychosocial intervention: 3 days/week,  CM (urine samples submitted):  3 days/week;  NR | Standard psychosocial treatment: 16,  CM: 4 | Standard psychosocial treatment intervention was manualized and based on the Matrix Model.  CM intervention was based on the variable magnitude reinforcement procedure (i.e., "fishbowl" technique). Participants were eligible for at least one draw for each methamphetamine-negative urine sample. An escalating schedule was used whereby the number of draws increased by one chip with consecutive weeks of abstinence. The number of draws was reset to one for the next eligible draw if participants missed a urine screen or had a methamphetamine-positive urine sample. Chips ranged in value from no monetary value to jumbo ($80-100). Fifty percent of chips were marked with "Good job" and had no monetary value; 41.8% were 'small' and worth about $1.00; 8% were 'large' and worth about $20, and 0.2% were 'jumbo' and worth about $80. Participants exchanged their chips for prizes within each category. Maximum a participant could receive was approximately $500; the actual maximum received averaged ≤$250 per participant. | NR |
|  | Standard psychosocial intervention: 48,  CM: 24 urine samples | CM: individual,  Standard psychological treatment: group | Standard psychosocial intervention: 3 days/week,  CM (urine samples submitted):  3 days/week;  NR | Standard psychosocial treatment: 16,  CM: 8 |  |  |
|  | Standard psychosocial intervention: 48,  CM: 48 urine samples | CM: individual,  Standard psychological treatment: group | Standard psychosocial intervention: 3 days/week,  CM (urine samples submitted):  3 days/week;  NR | Standard psychosocial treatment: 16,  CM: 16 |  |  |
|  | 48 | Group | 3 days/week;  NR | 16 | Standard psychosocial treatment intervention was manualized and based on the Matrix Model and delivered in a group setting (3 times/week for 16 weeks). |  |
| **Shoptaw 2005** (40–42)   - CM - CBT - CBT + CM - Gay and bisexual men-specific CBT | 48 urine samples | Individual | Urine samples submitted:  3 days/week | 16 | *Voucher-base reinforcement CM:* Participants received vouchers, redeemable for goods or services, for each urine sample that was negative for methamphetamine and cocaine metabolites. Urine samples were submitted three times per week. Vouchers started at US$ 2.50 for the first metabolite-negative sample and increased in value by US$ 2.50 for each sample thereafter. For three consecutive metabolite-negative urine samples, a bonus voucher of US$ 10.00 was provided. The value of the voucher was reset after relapse. Maximum cash value of vouchers was $1277.50. Intervention delivered by behavioral technician with bachelor’s level education and with an interest in studying behavioral therapy. | NR |
|  | 48 | Group | 3 day/week;  90 min | 16 | *CBT:* This intervention served as the standard of care. CBT was based on the Matrix Model. Participants taught about internal and external triggers, stages of recovery from methamphetamine dependence, identification of emotional states that can signal relapse, and cognitive skills (e.g., thought stopping, craving management, relapse analysis, healthy lifestyle behaviors). Intervention delivered by therapist with master’s level degrees or coursework and experience in conducting CBT. |  |
|  | CBT: 48  CM: 48 urine samples | *CBT:* Group  *CM:* Individual | *CBT:* 3 days/week  *CM* (urine samples submitted)*:* 3 days/week;  *CBT:* 90 min | 16 | *CBT + CM:* Received all components of both the CBT and contingency management interventions. |  |
|  | 48 | Group | 3 day/week;  90 min | 16 | *Gay and bisexual men-specific cognitive behavioral therapy:* Combined concepts of standard CBT (based on Matrix Model) with relevant behavioral and cultural aspects of methamphetamine use by gay and bisexual men. All topics used gay referents and sessions included discussions of "types of sexual behaviors engaged in when on and off drug, recognition of characteristics of sexual partners and important others who do and do not use methamphetamine, and recognition that revealing one’s drug problem is similar to the coming out process". Intervention delivered by therapist with master’s level degrees or coursework and experience in conducting CBT. |  |
| **Shoptaw 2008** (43)   - Gay-specific CBT - Gay social support therapy | 48 | Group | 3 days/week | 16 | *Gay-specific cognitive–behavioral therapy (GCBT):* Manualized program based on the matrix model with the aim of reducing substance use and high-risk sexual behaviours. The program incorporated "relevant cultural aspects of methamphetamine use by gay and bisexual men with CBT". Participants were taught skills to reduce drug use (e.g., thought stopping, craving management) and sexual risk behaviours (e.g., identifying triggers, thought stopping). Intervention delivered by therapists who had bachelor’s degrees, supervised experience in conducting CBT, and received 3 days training in GCBT. | NR |
|  | 48 | Combination (group and individual sessions) | 3 days/week | 16 | *Gay social support therapy (GSST):* The program incorporated components of peer-driven social model counselling with HIV health education/risk reduction. Participants attended a weekly HIV health education/risk reduction group, an open-ended social support group, and one-on-one discussion with a counselor (non-directive, participant-led). Intervention delivered by therapists with bachelor’s degrees, experience with social model recovery and received 3 days training in social support therapy. |  |
| **Smout 2010** (44)   - CBT - ACT | 12 | Individual | 1 day/week;  60 min | 12 | *CBT:* Sessions focused on building rapport; enhancing motivation to minimize use; developing coping skills for current and a coping plan for future high-risk situations; education about apparently irrelevant decisions; long-term relapse prevention skills training; and CBT for other psychosocial problems that impact drug use. Intervention delivered by two therapists; a doctoral-level psychologist with 4 years’ experience and a Masters-level psychologist with 1.5 years’ experience. | NR |
|  | 12 | Individual | 1 day/week;  60 min | 12 | *ACT:* Sessions focused on: values clarification and valued activity scheduling, acceptance and defusion exercises (to combat barriers to enacting valued activities as they arose), and discussions of the impact of methamphetamine use on performance of valued activities. Two therapists, a doctoral-level psychologist with 4 years’ experience and a Masters-level psychologist with 1.5 years’ experience, provided ACT. | NR |
| **Baker 2005** (45–47)   - 4 session CBT - 2 session CBT - Self-help booklet | 4 | Individual | NR;  45-60 min | 4 | *4 session CBT:* Treatment was guided by a manual and self-help booklet with a focus on developing skills to reduce amphetamine use. Sessions included role-playing and homework. Specific components included a motivational interview, cognitive–behavioural coping strategies, relapse prevention, management of craving, controlling thoughts about amphetamine use, and coping with lapses. The self-help booklet covered "amphetamine-related harms and suggestions for reducing amphetamine use". Intervention delivered by university graduates with clinical experience in the substance abuse field (3 psychologists and 1 social worker); received a week-long training session on the intervention delivery. | NR |
|  | 2 | Individual | NR;  45-60 min | 4 | *2 session CBT:* Same intervention components as 4-session CBT but with fewer sessions. |  |
|  | NA | NA | NA | 4 | *Self-help booklet:* Participants received the same self-help booklet as the intervention groups. |  |
| **Abdoli 2019** (48)   - CBT: Marlatt cognitive behavioral model for relapse prevention - No treatment | 12 | Group | once a week;  120 min | 12 | *Marlatt cognitive-behavioral model for relapse prevention* consisted of group sessions with brief lectures, discussions, role-playing, and assignments. | NR |
|  | NA | NA | NA | 12 | *No treatment:* Participants did not receive CBT |  |
| **Rawson 2004** (49–51)   - Matrix model - Treatment as usual (best available option) | CBT: 36,  Family education groups: 12,  Social support groups: 4,  Individual counselling: 4 | Matrix model consists of a combination of group, individual and family-based interventions | CBT: 3 times/week, NR for other intervention components;  NR | 16 | Manualized treatment protocol consisting cognitive behavioral therapy groups, family education groups, social support groups and individual counselling, weekly breath alcohol testing and urine testing for cocaine, methamphetamine, opiates, cannabis and benzodiazepines. Participants were also encouraged to attend 12-Step meetings (weekly or more). Intervention delivered by staff with 40 hours of didactic and experiential training; they also received booster training sessions at each site and feedback from site Matrix clinicians. | Encouraged to participate in continuing care activities following treatment completion. |
|  | Varied across sites | Individual and group sessions in majority of sites | NR;  1-13 hours per week | 8-16 (varied across sites) | Treatment as usual conditions followed a community-based treatment approach and the delivered interventions represented a ‘best available option’ and not a ‘minimal contact comparison’ condition. TAU varied widely across the eight sites (comprising of individual and group sessions and either encouragement or requirement of 12-step program involvement). Participants were required to receive treatment for 1 to 13 hours per week. TAU clinical staff were supervised by each program’s clinical director and were not involved in training or delivery of the Matrix Model intervention. |  |
| **Amiri 2016** (52)   - Matrix Model (Regulated 12-session MM) - No treatment (wait list control) | 12 | Unclear | 1 day/week;  60 min | 12 | The focus of the program was craving management. Sessions covered various topics including issues in remission, shame and guilt, motivation for abstinence, and relapse. The Matrix model is based on multiple components including behavioral therapy, relapse prevention research, motivational interviewing, psychoeducation information, and the 12-steps program. | NR |
|  | NA | NA | NA | 12 | Wait list control |  |
| **Polcin 2014** (53,54)   - Intensive motivational interviewing - Standard motivational interviewing | 9 | Individual | 1 day/week;  NR | 9 | *Intensive motivational interviewing* followed a manualized program; however, therapists were given flexibility regarding the problems and topics covered in the sessions. The goal of the first session was to identify the problems and motivation for change. The second and third sessions covered the advantages and disadvantages of making changes and developing a change plan, respectively. Implementation of the change plan was the focus of the remaining sessions. | Group cognitive behavioral therapy that emphasized craving management, three times per week for up to 12 weeks (eight weeks of active treatment and four weeks of aftercare). |
|  | 1 session of standard motivational interviewing,  8 sessions of nutrition education | Individual | Standard motivational interviewing was offered once, and nutrition education was offered 1 day/week over the remaining 8 weeks | 9 | *Standard motivational interviewing:* NR;  *Nutrition education:* The 8 nutrition education sessions were incorporated to achieve time equivalence with the intense motivational interviewing arm (9 sessions). Sessions covered various topics including weight management, exercise, cholesterol, nutritional content of food, and the food pyramid. |  |
| **Ghasemi 2014** (55)   - Educational intervention - Treatment as usual | 9 | Group | NR | Unclear | "The general principles of the intervention were as follows: acknowledgment, assurance, empathy, encouragement, and provision of chance to express emotions in order to obtain social support from others." | NR |
|  | NR | NR | NR | Unclear | NR |  |
| **Mimiaga 2019** (56)   - Behavioural activation + sexual risk reduction (BA-SRR) counselling - Sexual risk reduction counselling (SRR) | 13 | Individual | NR | 12 | Modules of the BA-SRR intervention were informed by motivational interviewing. The intervention included modules on sexual risk reduction, cognitive behaviour therapy for substance abuse, behavioural activation integrated with risk reduction counselling, and relapse prevention. In total, participants received 13 sessions: two sessions of sexual risk reduction, ten sessions of behavioural activation with sexual risk reduction, and one session of relapse prevention. | NR |
|  | 2 | Individual | NR | 12 | Participants in the control group received the same two sessions of sexual risk reduction offered to the intervention group. Control participants did not receive behavioural activation or relapse prevention sessions. The two sessions of sexual risk reduction counselling received were based on the IMB skills change approach to sexual risk reduction. |  |
| **Reback 2019** (57)   - Mobile-based text messaging: Interactive text-messaging conversations with peer health educators (PHE) + theory-based, gay-specific text messages transmitted by automation + weekly self-monitoring text-based assessments - Mobile-based text messaging: Theory-based, gay-specific, text messages transmitted by automation + weekly self-monitoring text-based assessments - Mobile-based text messaging: Weekly self-monitoring text-based assessments | 280 scripted gay-specific text messages,  NR for PHE interaction,  8 self-monitoring text-based assessments | Individual | 5 scripted gay-specific text messages per day;  Maximum 4 conversations of maximum 40 messages between PHE and participant per day;  1 day/week self-monitoring text-based assessments | 8 | *Interactive text-messaging conversations with peer health educators + theory-based, gay-specific text messages transmitted by automation + weekly self-monitoring text-based assessments:* Participants received five scripted theory-based gay-specific text messages per day (total of 280 over 8 week period). Participants assessed at baseline as having additional risk profiles (e.g., injection drug use, HIV status, ART adherence if HIV+) were given messages tailored to their specific risk profile in place of the general messages. Participants were able to respond to the scripted messages or send in questions to initiate text-messaging interaction with the PHE. The text messages sent by the PHE focused on health promotion (including HIV prevention text message) and referrals. A maximum of four conversations were allowed per day with a maximum of 40 messages total per conversation. Participants also received a brief weekly text-based assessment of methamphetamine use and HIV sexual behaviours (in previous week). | NR |
|  | 280 scripted gay-specific text messages,  8 self-monitoring text-based assessments | Individual | 5 scripted gay-specific text messages per day; 1 day/week self-monitoring text-based assessments | 8 | *Theory-based, gay-specific, text messages transmitted by automation + weekly self-monitoring text-based assessments:*  Participants received five scripted theory-based gay-specific text messages per day (total of 280 over 8 week period). Participants assessed at baseline as having additional risk profiles (e.g., injection drug use, HIV status, ART adherence if HIV+) were given messages tailored to their specific risk profile in place of the general messages. Participants also received a brief weekly text-based assessment of methamphetamine use and HIV sexual behaviours (in previous week). |  |
|  | 8 self-monitoring text-based assessments | Individual | 1 day/week | 8 | *Weekly self-monitoring text-based assessments:* Participants received a brief weekly text-based assessment of methamphetamine use and HIV sexual behaviours (in previous week). |  |
| **Reback 2018** (58)   - Counselling + mobile app-based ecological momentary assessments (EMA) + web-based visualization dashboard - Mobile app-based ecological momentary assessments (EMA) + web-based dashboard - Matched historical control group | Counselling: 8,  EMA surveys: 280 | Individual | Counselling: 1 day/week,  EMA surveys: 5 times/day;  Counselling: 30 min,  EMA surveys: less than three minutes (the sexual encounter questions: less than two minutes for each sexual encounter reported). | 8 | Participants received prompts to complete mobile app-based EMA surveys five time/day (every 3 hours). Surveys evaluated: internal and external triggers, substance use and craving, and sexual encounters. Participants could also complete a survey when an event occurred (e.g., experienced a craving).  Participants had access to a web-based dashboard which displayed EMA survey responses.  Participants received weekly counselling where urinalysis dashboard and EMA survey responses were discussed with a counsellor. Various topics were also covered including problem solving triggers, substance use and high-risk sexual behaviours. Counselling delivered by a certified substance abuse counselor. | Getting Off intensive outpatient methamphetamine abuse treatment service program (24 sessions over 8 weeks) which is a modified version of Gay-specific Cognitive Behavioral Therapy (GCBT). The program also includes a low-cost contingency management intervention for submission of methamphetamine-negative urine samples. |
|  | EMA surveys: 280 | Individual | EMA surveys: 5 times/day;  EMA surveys: less than three minutes (the sexual encounter questions: less than two minutes for each sexual encounter reported). | 8 | Participants received prompts to complete mobile app-based EMA surveys five time/day (every 3 hours). Surveys evaluated: internal and external triggers, substance use and craving, and sexual encounters. Participants could also complete a survey when an event occurred (e.g., experienced a craving).  Participants had access to a web-based dashboard which displayed EMA survey responses. |  |
|  | NA | NA | NA | NA | The matched historical control group was selected from a sample of participants that received the Getting Off outpatient treatment program (24 group sessions over 8 weeks) which is a modified version of Gay-specific Cognitive Behavioral Therapy (GCBT). The program also included a low-cost contingency management intervention for submission of methamphetamine-negative urine samples. *Not a randomized study arm. | NR |
| **Perngparn 2011** (59)   - Inpatient residential rehabilitation treatment (FAST model) - Outpatient Matrix Model treatment | NR | Combination (family, individual, group) | NR | 16 | *Residential rehabilitation:* Inpatients in a treatment rehabilitation program received the FAST Model (F – Family, A – Alternative treatment activities, S – Self-help and T – Therapeutic community). Intervention components not adequately described. | NR |
|  | NR | Unclear | NR | 16 | *Matrix Model:* Out-patients received the Matrix model (no description provided). |  |
| **Ciketic 2013** (60,61)   - Community-based residential rehabilitation - Community-based detoxification (inpatient or outpatient) - Outpatient counselling - No treatment | NR | NR | NR | Median (IQR): 62 days (29-98 days) | *Community-based residential rehabilitation:* Residential rehabilitation typically involved inpatient stays from several weeks to months and included "integrated services and therapeutic activities (e.g. behavioural treatment approaches, recreational activities, social and community living skills, group work and relapse prevention)". Some variation in the type of treatments provided across residential rehabilitation facilities was expected. | NR |
|  | NR | NR | NR | Median (IQR): 5 days (4–7 days) | *Community-based detoxification (inpatient or outpatient):* Inpatient detoxification stays were "typically...brief (e.g. 1 week)...with medical support to manage withdrawal symptoms." Some variation in the type of treatments provided across detoxification services was expected. |  |
|  | NR | NR | NR | NR | *Outpatient counselling:* Not further described. |  |
|  | NA | NA | NA | NR | Non-intervention comparison group |  |
| **McKetin 2018** (62)   - Individual counselling during community-based residential rehabilitation - No individual counselling during community-based residential rehabilitation | NR | Individual | NR | Median (IQR): 59 days (29–98 days) | Residential rehabilitation typically involved inpatient stays from several weeks to months and included "integrated services and therapeutic activities (e.g. behavioural treatment approaches, recreational activities, social and community living skills, group work and relapse prevention)". Some variation in the type of treatments provided across residential rehabilitation facilities was expected. For example, some participants received individual counselling while others did not. | NR |
|  | NR | Unclear (no individual counselling received but some could have received group counselling) | NR | Median (IQR): 59 days (29–98 days) |  |  |
| **Kamp 2019** (63,64)   - Conventional group therapy + 10 hours of group therapy focusing on stimulant use (amphetamine type stimulant (ATS) residential treatment program - Conventional group therapy only (treatment as usual) residential treatment program | Conventional group therapy component: approximately 72,  ATS group therapy: approximately 24 | Group | Conventional group therapy component: 3 times/week;  ATS group therapy: once per week | ~ 24 | All participants were in-patients at the Hochstadt residential rehabilitation facility.  Participants received conventional group therapy plus an additional 10 hours of group therapy focusing on stimulant use.  Conventional group therapy concepts not described for this institution.  The Amphetamine Type Stimulants (ATS) group therapy portion covered the following concepts: psychoeducation, effects of MA use on emotions, coping with avolition and lack of motivation after MA cessation, effects of MA use and changes in sleep, relapse prevention, sexuality and MA use, alcohol and MA use, other compulsive or impulsive behaviors than substance use and voiding relapse traps. | NR |
|  | approximately 120 | Group | 5 times/week | ~ 24 | All participants were in-patients at the Mecklenburg residential rehabilitation facility.  Conventional group therapy (5 times per week) at this institution consisted of the following concepts: relapse prevention, self-reflection, development of new behavioral strategies, and proving experiments. |  |
| **Abdoli 2021** (65)   - Self-compassion training - Control (not further described) | 8 | Group | Twice weekly;  60-90 min | 4 | Self-compassion training, delivered by an experienced clinical psychologist, covered various topics including empathy, self-compassion, forgiveness, acceptance of problems, and accountability development. | Routine therapies and counseling (not further described). |
|  | NR | NR | NR | NR | NR |  |
| **Sorsdahl 2021** (66)   - Blended imaginal desensitisation plus motivational interviewing - Treatment as usual | 6 | Individual | NR (likely once weekly);  NR |  | The six session blended imaginal desensitisation plus motivational interviewing intervention included a condensed version of motivational enhancement therapy, financial planning, a behavioural intervention for methamphetamine use triggers, exposure therapy, cognitive therapy, relapse prevention, and an optional session including family involvement. The intervention was delivered by two clinical psychologists with >5 years experience delivering CBT. | NR |
|  | NR | NR | NR | NR | Treatment as usual condition consisted of referral to a specialized, registered outpatient rehabilitation centre. All of the referral facilities provided individual and group-based motivational interviewing and cognitive behavioural counseling sessions. |  |

Abbreviations: ACT = acceptance and commitment therapy; CBT = cognitive behavioural therapy; CM: contingency management; NR = not reported; NA = not applicable

1. **Combination pharmacological and psychosocial interventions**

| **Author Year {refID}** | **Intervention arm** | **Pharmacology intervention schedule;**  **Total daily dose;**  **Route of administration;** | **Psychosocial intervention** | **Treatment duration (weeks)** | **Co-interventions** |
| --- | --- | --- | --- | --- | --- |
| **Shoptaw 2006** (67,68) | Sertraline | Sertraline 50 mg once a day (week 1); 50 mg BID (week 2-12);  100 mg/day  Oral | NA | 12 | Standardized and manual-driven MM relapse prevention group sessions (3 times/week for 90 minutes). |
|  | Placebo | NA | NA | 12 |  |
|  | Sertraline + CM | Sertraline 50 mg once a day (week 1); 50 mg BID (week 2-12);  100 mg/day  Oral | *Contingency management:* Participants received vouchers, redeemable for goods or services, for each methamphetamine-negative urine sample. Urine samples were submitted three times per week (total of 36 urine samples submitted). Vouchers started at US$ 2.50 for the first methamphetamine-negative sample and increased in value by US$ 1.25 for each sample thereafter. For three consecutive methamphetamine-negative urine samples, a bonus voucher of US$ 10.00 was provided. For missed or methamphetamine-positive urine samples, no voucher was received, and a reset procedure was followed whereby the value of the next voucher for a methamphetamine-negative sample was reset to US$ 2.50. | 12 |  |
|  | Placebo + contingency management | NA |  | 12 |  |
| **Aryan 2020** (69) | Methylphenidate | 10 mg/day (first month), 7.5 mg/day (second month), 5 mg/day (third month);  Dosage varied monthly;  Oral | NA | 12 | NR |
|  | Matrix model | NA | *Matrix model:* Matrix model treatment sessions (twice weekly for 45 min; total of 22 sessions) covered a variety of topics including external and internal triggers, relapse prevention, and motivation for recovery. | 12 |  |
|  | No treatment | NA | NA | 12 |  |
|  | Methylphenidate + Matrix model | 10 mg/day (first month), 7.5 mg/day (second month), 5 mg/day (third month);  Dosage varied monthly;  Oral | *Matrix model:* Matrix model treatment sessions (twice weekly for 45 min; total of 22 sessions) covered a variety of topics including external and internal triggers, relapse prevention, and motivation for recovery. | 12 |  |

**Reference List**

1. Das M, Santos D, Matheson T, Santos G-M, Chu P, Vittinghoff E, et al. Feasibility and acceptability of a phase II randomized pharmacologic intervention for methamphetamine dependence in high-risk men who have sex with men. AIDS. 2010 Apr 24;24(7):991–1000.

2. Anderson AL, Li S-H, Markova D, Holmes TH, Chiang N, Kahn R, et al. Bupropion for the treatment of methamphetamine dependence in non-daily users: a randomized, double-blind, placebo-controlled trial. Drug Alcohol Depend. 2015 May 1;150:170–4.

3. Elkashef AM, Rawson RA, Anderson AL, Li S-H, Holmes T, Smith EV, et al. Bupropion for the treatment of methamphetamine dependence. Neuropsychopharmacology. 2008 Apr;33(5):1162–70.

4. McCann DJ, Li S-H. A novel, nonbinary evaluation of success and failure reveals bupropion efficacy versus methamphetamine dependence: reanalysis of a multisite trial. CNS Neurosci Ther. 2012 May;18(5):414–8.

5. Heinzerling KG, Swanson A-N, Hall TM, Yi Y, Wu Y, Shoptaw SJ. Randomized, placebo-controlled trial of bupropion in methamphetamine-dependent participants with less than daily methamphetamine use. Addiction. 2014 Nov;109(11):1878–86.

6. Shoptaw S, Heinzerling KG, Rotheram-Fuller E, Steward T, Wang J, Swanson A-N, et al. Randomized, placebo-controlled trial of bupropion for the treatment of methamphetamine dependence. Drug Alcohol Depend. 2008 Aug 1;96(3):222–32.

7. Brensilver M, Heinzerling KG, Swanson A-N, Telesca D, Furst BA, Shoptaw SJ. Cigarette smoking as a target for potentiating outcomes for methamphetamine abuse treatment. Drug Alcohol Rev. 2013 Jan;32(1):96–9.

8. Coffin PO, Santos G-M, Hern J, Vittinghoff E, Walker JE, Matheson T, et al. Effects of Mirtazapine for Methamphetamine Use Disorder Among Cisgender Men and Transgender Women Who Have Sex With Men: A Placebo-Controlled Randomized Clinical Trial. JAMA Psychiatry. 2020 Mar 1;77(3):246–55.

9. Colfax GN, Santos G-M, Das M, Santos DM, Matheson T, Gasper J, et al. Mirtazapine to reduce methamphetamine use: a randomized controlled trial. Arch Gen Psychiatry. 2011 Nov;68(11):1168–75.

10. Galloway GP, Newmeyer J, Knapp T, Stalcup SA, Smith D. A controlled trial of imipramine for the treatment of methamphetamine dependence. J Subst Abuse Treat. 1996 Dec;13(6):493–7.

11. Wang G, Ma L, Liu X, Yang X, Zhang S, Yang Y, et al. Paliperidone Extended-Release Tablets for the Treatment of Methamphetamine Use Disorder in Chinese Patients After Acute Treatment: A Randomized, Double-Blind, Placebo-Controlled Exploratory Study. Front Psychiatry. 2019;10:656.

12. Coffin PO, Santos G-M, Das M, Santos DM, Huffaker S, Matheson T, et al. Aripiprazole for the treatment of methamphetamine dependence: a randomized, double-blind, placebo-controlled trial. Addiction. 2013 Apr;108(4):751–61.

13. Briones M, Shoptaw S, Cook R, Worley M, Swanson A-N, Moody DE, et al. Varenicline treatment for methamphetamine dependence: A randomized, double-blind phase II clinical trial. Drug Alcohol Depend. 2018 Aug 1;189:30–6.

14. Anderson AL, Li S-H, Biswas K, McSherry F, Holmes T, Iturriaga E, et al. Modafinil for the treatment of methamphetamine dependence. Drug Alcohol Depend. 2012 Jan 1;120(1–3):135–41.

15. Heinzerling KG, Swanson A-N, Kim S, Cederblom L, Moe A, Ling W, et al. Randomized, double-blind, placebo-controlled trial of modafinil for the treatment of methamphetamine dependence. Drug Alcohol Depend. 2010 Jun 1;109(1–3):20–9.

16. Shearer J, Darke S, Rodgers C, Slade T, van Beek I, Lewis J, et al. A double-blind, placebo-controlled trial of modafinil (200 mg/day) for methamphetamine dependence. Addiction. 2009 Feb;104(2):224–33.

17. Shearer J, Shanahan M, Darke S, Rodgers C, van Beek I, McKetin R, et al. A cost-effectiveness analysis of modafinil therapy for psychostimulant dependence. Drug Alcohol Rev. 2010 May;29(3):235–42.

18. Fard MT, Mansouri SS, Jafari A, Vousooghi N. Role of modafinil in the treatment of patients with methamphetamine dependence; An update on randomized, controlled clinical trial. Trop J Pharm Res. 2020 Nov 26;19(10):2179–85.

19. Heinzerling KG, Shoptaw S, Peck JA, Yang X, Liu J, Roll J, et al. Randomized, placebo-controlled trial of baclofen and gabapentin for the treatment of methamphetamine dependence. Drug Alcohol Depend. 2006 Dec 1;85(3):177–84.

20. Kheirabadi GR, Ghavami M, Maracy MR, Salehi M, Sharbafchi MR. Effect of add-on valproate on craving in methamphetamine depended patients: A randomized trial. Adv Biomed Res. 2016;5:149.

21. Coffin PO, Santos G-M, Hern J, Vittinghoff E, Santos D, Matheson T, et al. Extended-release naltrexone for methamphetamine dependence among men who have sex with men: a randomized placebo-controlled trial. Addiction. 2018 Feb;113(2):268–78.

22. Ling W, Chang L, Hillhouse M, Ang A, Striebel J, Jenkins J, et al. Sustained-release methylphenidate in a randomized trial of treatment of methamphetamine use disorder. Addiction. 2014 Sep;109(9):1489–500.

23. Noroozi A, Motevalian SA, Zarrindast M-R, Alaghband-Rad J, Akhondzadeh S. Adding extended-release methylphenidate to psychological intervention for treatment of methamphetamine dependence: A double-blind randomized controlled trial. Med J Islam Repub Iran. 2020;34:137.

24. Brown ES, Gabrielson B. A randomized, double-blind, placebo-controlled trial of citicoline for bipolar and unipolar depression and methamphetamine dependence. J Affect Disord. 2012 Dec 20;143(1–3):257–60.

25. Longo M, Wickes W, Smout M, Harrison S, Cahill S, White JM. Randomized controlled trial of dexamphetamine maintenance for the treatment of methamphetamine dependence. Addiction. 2010 Jan;105(1):146–54.

26. Elkashef A, Kahn R, Yu E, Iturriaga E, Li S-H, Anderson A, et al. Topiramate for the treatment of methamphetamine addiction: a multi-center placebo-controlled trial. Addiction. 2012 Jul;107(7):1297–306.

27. Ma JZ, Johnson BA, Yu E, Weiss D, McSherry F, Saadvandi J, et al. Fine-grain analysis of the treatment effect of topiramate on methamphetamine addiction with latent variable analysis. Drug Alcohol Depend. 2013 Jun 1;130(1–3):45–51.

28. Heinzerling KG, Briones M, Thames AD, Hinkin CH, Zhu T, Wu YN, et al. Randomized, Placebo-Controlled Trial of Targeting Neuroinflammation with Ibudilast to Treat Methamphetamine Use Disorder. J Neuroimmune Pharmacol. 2020 Jun;15(2):238–48.

29. Farahzadi M-H, Moazen-Zadeh E, Razaghi E, Zarrindast M-R, Bidaki R, Akhondzadeh S. Riluzole for treatment of men with methamphetamine dependence: A randomized, double-blind, placebo-controlled clinical trial. J Psychopharmacol. 2019 Mar;33(3):305–15.

30. Salehi M, Emadossadat A, Kheirabadi GR, Maracy MR, Sharbafchi MR. The Effect of Buprenorphine on Methamphetamine Cravings. J Clin Psychopharmacol. 2015 Dec;35(6):724–7.

31. Kheirabadi GR, Bahrami M, Shariat A, Tarrahi M. The Effect of Add-on Buprenorphine to Matrix Program in Reduction of Craving and Relapse Among People With Methamphetamine Use Disorder: A Randomized Controlled Trial. J Clin Psychopharmacol. 2021 Feb 1;41(1):45–8.

32. Ling W, Shoptaw S, Hillhouse M, Bholat MA, Charuvastra C, Heinzerling K, et al. Double-blind placebo-controlled evaluation of the PROMETA^TM^ protocol for methamphetamine dependence. Addiction. 2012 Feb;107(2):361–9.

33. Trivedi MH, Walker R, Ling W, Dela Cruz A, Sharma G, Carmody T, et al. Bupropion and Naltrexone in Methamphetamine Use Disorder. N Engl J Med. 2021 Jan 14;384(2):140–53.

34. McKetin R, Dean OM, Turner A, Kelly PJ, Quinn B, Lubman DI, et al. N-acetylcysteine (NAC) for methamphetamine dependence: A randomised controlled trial. EClinicalMedicine. 2021 Aug;38:101005.

35. Chudzynski J, Roll JM, McPherson S, Cameron JM, Howell DN. Reinforcement Schedule Effects on Long-Term Behavior Change. Psychol Rec. 2015 Jun 1;65(2):347–53.

36. Roll JM, Shoptaw S. Contingency management: schedule effects. Psychiatry Res. 2006 Sep 30;144(1):91–3.

37. Roll JM, Huber A, Sodano R, Chudzynski JE, Moynier E, Shoptaw S. A Comparison of Five Reinforcement Schedules for use in Contingency Management-Based Treatment of Methamphetamine Abuse. Psychol Rec. 2006 Jan;56(1):67–81.

38. Roll JM, Petry NM, Stitzer ML, Brecht ML, Peirce JM, McCann MJ, et al. Contingency management for the treatment of methamphetamine use disorders. Am J Psychiatry. 2006 Nov;163(11):1993–9.

39. Roll JM, Chudzynski J, Cameron JM, Howell DN, McPherson S. Duration effects in contingency management treatment of methamphetamine disorders. Addict Behav. 2013 Sep;38(9):2455–62.

40. Shoptaw S, Reback CJ, Peck JA, Yang X, Rotheram-Fuller E, Larkins S, et al. Behavioral treatment approaches for methamphetamine dependence and HIV-related sexual risk behaviors among urban gay and bisexual men. Drug Alcohol Depend. 2005 May 9;78(2):125–34.

41. Peck JA, Reback CJ, Yang X, Rotheram-Fuller E, Shoptaw S. Sustained reductions in drug use and depression symptoms from treatment for drug abuse in methamphetamine-dependent gay and bisexual men. J Urban Health. 2005 Mar;82(1 Suppl 1):i100-108.

42. Jaffe A, Shoptaw S, Stein J, Reback CJ, Rotheram-Fuller E. Depression ratings, reported sexual risk behaviors, and methamphetamine use: latent growth curve models of positive change among gay and bisexual men in an outpatient treatment program. Exp Clin Psychopharmacol. 2007 Jun;15(3):301–7.

43. Shoptaw S, Reback CJ, Larkins S, Wang P-C, Rotheram-Fuller E, Dang J, et al. Outcomes using two tailored behavioral treatments for substance abuse in urban gay and bisexual men. J Subst Abuse Treat. 2008 Oct;35(3):285–93.

44. Smout MF, Longo M, Harrison S, Minniti R, Wickes W, White JM. Psychosocial treatment for methamphetamine use disorders: a preliminary randomized controlled trial of cognitive behavior therapy and Acceptance and Commitment Therapy. Subst Abus. 2010 Apr;31(2):98–107.

45. Baker A, Lee NK, Claire M, Lewin TJ, Grant T, Pohlman S, et al. Brief cognitive behavioural interventions for regular amphetamine users: a step in the right direction. Addiction. 2005 Mar;100(3):367–78.

46. Kay-Lambkin FJ, Baker AL, Lee NM, Jenner L, Lewin TJ. The influence of depression on treatment for methamphetamine use. Med J Aust. 2011 Aug 1;195(3):S38-43.

47. Lee NK, Pohlman S, Baker A, Ferris J, Kay-Lambkin F. It’s the thought that counts: craving metacognitions and their role in abstinence from methamphetamine use. J Subst Abuse Treat. 2010 Apr;38(3):245–50.

48. Abdoli N, Farnia V, Salemi S, Tatari F, Juibari TA, Alikhani M, et al. Efficacy of the Marlatt cognitive-behavioral model on decreasing relapse and craving in women with methamphetamine dependence: A clinical trial. Journal of Substance Use. 2019 Mar 4;24(2):229–32.

49. Rawson RA, Marinelli-Casey P, Anglin MD, Dickow A, Frazier Y, Gallagher C, et al. A multi-site comparison of psychosocial approaches for the treatment of methamphetamine dependence. Addiction. 2004 Jun;99(6):708–17.

50. Rawson RA, Gonzales R, Pearce V, Ang A, Marinelli-Casey P, Brummer J, et al. Methamphetamine dependence and human immunodeficiency virus risk behavior. J Subst Abuse Treat. 2008 Oct;35(3):279–84.

51. Rawson RA, Gonzales R, Greenwell L, Chalk M. Process-of-care measures as predictors of client outcome among a methamphetamine-dependent sample at 12- and 36-month follow-ups. J Psychoactive Drugs. 2012 Oct;44(4):342–9.

52. Amiri Z, Mirzaee B, Sabet M. Evaluating the efficacy of Regulated 12-Session Matrix Model in reducing susceptibility in methamphetamine-dependent individuals. International Journal of Medical Research & Health Sciences. 2016;5(2):77–85.

53. Polcin DL, Bond J, Korcha R, Nayak MB, Galloway GP, Evans K. Randomized trial of intensive motivational interviewing for methamphetamine dependence. J Addict Dis. 2014;33(3):253–65.

54. Korcha RA, Polcin DL, Evans K, Bond JC, Galloway GP. Intensive motivational interviewing for women with concurrent alcohol problems and methamphetamine dependence. J Subst Abuse Treat. 2014 Feb;46(2):113–9.

55. Ghasemi A, Estebsari F, Bastaminia A, Jamshidi E, Dastoorpoor M. Effects of Educational Intervention on Health-Promoting Lifestyle and Health-Related Life quality of Methamphetamine Users and Their Families: a Randomized Clinical Trial. Iran Red Crescent Med J. 2014 Nov;16(11):e20024.

56. Mimiaga MJ, Pantalone DW, Biello KB, Hughto JMW, Frank J, O’Cleirigh C, et al. An initial randomized controlled trial of behavioral activation for treatment of concurrent crystal methamphetamine dependence and sexual risk for HIV acquisition among men who have sex with men. AIDS Care. 2019 Sep;31(9):1083–95.

57. Reback CJ, Fletcher JB, Swendeman DA, Metzner M. Theory-Based Text-Messaging to Reduce Methamphetamine Use and HIV Sexual Risk Behaviors Among Men Who Have Sex with Men: Automated Unidirectional Delivery Outperforms Bidirectional Peer Interactive Delivery. AIDS Behav. 2019 Jan;23(1):37–47.

58. Reback CJ, Rünger D, Fletcher JB, Swendeman D. Ecological momentary assessments for self-monitoring and counseling to optimize methamphetamine treatment and sexual risk reduction outcomes among gay and bisexual men. J Subst Abuse Treat. 2018 Sep;92:17–26.

59. Perngparn U, Limanonda B, Aramrattana A, Pilley C, Areesantichai C, Taneepanichskul S. Methamphetamine dependence treatment rehabilitation in Thailand: a model assessment. J Med Assoc Thai. 2011 Jan;94(1):110–7.

60. Ciketic S, McKetin R, Doran CM, Najman JM, Veerman JL, Hayatbakhsh RM. Health-related quality of life (HRQL) among methamphetamine users in treatment. Mental Health and Substance Use. 2013 Aug;6(3):250–61.

61. McKetin R, Najman JM, Baker AL, Lubman DI, Dawe S, Ali R, et al. Evaluating the impact of community-based treatment options on methamphetamine use: findings from the Methamphetamine Treatment Evaluation Study (MATES). Addiction. 2012 Nov;107(11):1998–2008.

62. McKetin R, Kothe A, Baker AL, Lee NK, Ross J, Lubman DI. Predicting abstinence from methamphetamine use after residential rehabilitation: Findings from the Methamphetamine Treatment Evaluation Study. Drug Alcohol Rev. 2018 Jan;37(1):70–8.

63. Kamp F, Proebstl L, Hager L, Schreiber A, Riebschläger M, Neumann S, et al. Effectiveness of methamphetamine abuse treatment: Predictors of treatment completion and comparison of two residential treatment programs. Drug Alcohol Depend. 2019 Aug 1;201:8–15.

64. Kamp F, Hager L, Proebstl L, Schreiber A, Riebschläger M, Neumann S, et al. 12- and 18-month follow-up after residential treatment of methamphetamine dependence: Influence of treatment drop-out and different treatment concepts. J Psychiatr Res. 2020 Oct;129:103–10.

65. Abdoli N, Farnia V, Radmehr F, Alikhani M, Moradinazar M, Khodamoradi M, et al. The effect of self-compassion training on craving and self-efficacy in female patients with methamphetamine dependence: a one-year follow-up. Journal of Substance Use. 2021 Sep 3;26(5):491–6.

66. Sorsdahl K, Stein DJ, Pasche S, Jacobs Y, Kader R, Odlaug B, et al. A novel brief treatment for methamphetamine use disorders in South Africa: a randomised feasibility trial. Addict Sci Clin Pract. 2021 Jan 7;16(1):3.

67. Shoptaw S, Huber A, Peck J, Yang X, Liu J, Jeff Dang null, et al. Randomized, placebo-controlled trial of sertraline and contingency management for the treatment of methamphetamine dependence. Drug Alcohol Depend. 2006 Oct 15;85(1):12–8.

68. Zorick T, Sugar CA, Hellemann G, Shoptaw S, London ED. Poor response to sertraline in methamphetamine dependence is associated with sustained craving for methamphetamine. Drug Alcohol Depend. 2011 Nov 1;118(2–3):500–3.

69. Aryan N, Banafshe HR, Farnia V, Shakeri J, Alikhani M, Rahimi H, et al. The therapeutic effects of methylphenidate and matrix-methylphenidate on addiction severity, craving, relapse and mental health in the methamphetamine use disorder. Subst Abuse Treat Prev Policy. 2020 Sep 25;15(1):72.
