# Supplementary material for: Psychosocial and pharmacologic interventions for problematic methamphetamine use: Findings from a scoping review of the literature
Source: PLoS One. 2023 Oct 11;18(10):e0292745. doi: 10.1371/journal.pone.0292745 (PMC10566716; doi:10.1371/journal.pone.0292745)
Supplement: S11 Text — (DOCX) [file pone.0292745.s011.docx]

# S11 Text. Reported outcomes by study and outcome domains

| **Study** | **Meth use/ abstinence** | **Change in meth use** | **Meth and other substance use*** | **Other substance use**** | **Relapse** | **Mental health outcomes** | **Risk behaviours** | **Adverse events** | **Retention/ Dropout** |
| --- | --- | --- | --- | --- | --- | --- | --- | --- | --- |
| **Abdoli 2019** (1) |  |  |  |  |  |  |  |  |  |
| **Abdoli 2021** (2) |  |  |  |  |  |  |  |  | 2 |
| **Amiri 2016** (3) |  |  |  |  |  |  |  |  |  |
| **Anderson 2012** (4) | 6 | 6 | 3 | 3 |  | 9 | 6 |  | 6 |
| **Anderson 2015** (5) | 7 |  |  |  |  |  |  |  | 2 |
| **Aryan 2020** (6) |  | 6 |  |  |  | 6 |  |  | 12 |
| **Baker 2005** (7–9) | 3 | 18 | 6 |  |  | 12 | 12 |  | 12 |
| **Briones 2018** (10) |  |  |  |  |  | 1 |  | 1 | 2 |
| **Brown 2012** (11) |  | 2 |  |  |  | 1 |  | 1 | 2 |
| **Chudzynski 2015** (12) | 6 | 12 |  |  |  |  |  |  |  |
| **MATES study** (13–15) | 3 | 8 |  |  |  | 12 |  |  | 6 |
| **Coffin 2013** (16) | 3 | 3 |  |  |  | 1 | 6 | 1 | 4 |
| **Coffin 2018** (17) | 3 | 1 |  |  |  | 2 | 6 | 1 | 4 |
| **Coffin 2020** (18) | 9 | 3 |  |  |  | 3 | 18 | 1 | 2 |
| **Colfax 2011** (19) |  | 1 |  |  |  | 2 | 6 | 1 | 4 |
| **Das 2010** (20) |  | 1 |  |  |  |  | 5 | 2 | 4 |
| **Elkashef 2008** (21,22) | 17 | 8 | 1 | 3 |  | 3 | 2 |  | 2 |
| **Elkashef 2012** (23,24) |  | 9 | 1 | 1 |  | 1 | 1 | 2 | 2 |
| **Farahzadi 2019** (25) | 1 |  |  |  |  | 1 |  |  | 4 |
| **Fard 2020** (26) |  | 2 |  |  |  | 1 |  |  | 2 |
| **Galloway 1996** (27) |  | 1 |  |  |  | 1 |  | 1 | 2 |
| **Ghasemi 2014** (28) |  |  |  |  |  | 2 |  |  | 2 |
| **Heinzerling 2006** (29) | 18 | 3 |  |  |  | 3 |  | 3 | 9 |
| **Heinzerling 2010** (30) | 12 | 1 |  |  |  | 4 |  | 1 | 9 |
| **Heinzerling 2014** (31) | 2 | 2 |  |  |  | 1 |  | 1 | 3 |
| **Heinzerling 2020** (32) | 9 | 3 |  |  |  | 2 |  | 1 | 3 |
| **Kamp 2019** (33,34) | 1 |  |  | 2 |  | 4 |  |  | 6 |
| **Kheirabadi 2016** (35) | 6 |  |  |  |  |  |  |  | 2 |
| **Kheirabadi 2021** (36) |  |  |  |  |  |  |  |  |  |
| **Ling 2012** (37) | 2 | 1 |  |  |  | 3 |  | 1 | 7 |
| **Ling 2014** (38) | 2 |  |  | 2 |  |  |  | 1 | 5 |
| **Longo 2009** (39) |  | 3 |  |  |  | 1 |  | 2 | 6 |
| **Mimiaga 2019** (40) |  |  |  |  |  |  |  |  | 4 |
| **McKetin 2021** (41) | 1 | 1 |  |  |  | 5 |  |  |  |
| **Noroozi 2020** (42) | 2 | 2 |  | 9 |  | 3 | 4 |  | 2 |
| **Perngparn 2011** (43) |  |  |  |  | 1 | 4 | 1 |  | 3 |
| **Polcin 2014** (44)  **Korcha 2014** (45) |  | 1 | 1 | 1 |  | 4 |  |  | 2 |
| **Rawson 2004** (46–48) | 3 | 2 | 1 | 1 |  |  | 2 |  | 2 |
| **Reback 2018** (49) | 3 | 2 |  |  |  |  | 4 |  | 8 |
| **Reback 2019** (50) |  | 2 |  |  |  |  | 10 |  | 12 |
| **Roll 2006** (51) | 2 |  |  |  |  |  |  |  |  |
| **Roll 2006** (52) | 40 |  |  |  | 6 |  |  |  |  |
| **Roll 2006** (53) |  |  | 6 | 1 |  |  |  |  | 5 |
| **Roll 2013** (54) | 18 | 12 |  |  |  |  |  |  | 24 |
| **Salehi 2015** (55) | 3 |  |  |  |  |  |  | 3 |  |
| **Shearer 2009** (56,57) |  |  | 3 |  |  | 2 |  | 1 | 4 |
| **Shoptaw 2005** (58–60) | 36 | 3 |  |  |  | 6 | 9 |  | 6 |
| **Shoptaw 2006** (61,62) | 8 | 8 |  |  |  | 8 |  |  | 18 |
| **Shoptaw 2008** (63) | 4 | 2 |  |  |  | 4 | 3 |  |  |
| **Shoptaw 2008** (64,65) | 19 | 5 |  |  |  |  |  | 1 | 18 |
| **Smout 2010** (66) |  | 4 |  | 4 |  | 4 |  | 1 | 5 |
| **Sorsdahl 2021** (67) |  | 1 |  |  |  | 2 |  |  | 2 |
| **Trivedi 2021** (68) | 2 |  |  |  |  | 5 |  | 2 | 2 |
| **Wang 2019** (69) | 2 |  |  |  | 1 | 4 |  | 3 | 6 |
| **Total number studies;**  **Total number estimates** | 32;  253 | 34;  139 | 8;  22 | 10;  27 | 3;  8 | 35;  127 | 16;  95 | 22;  32 | 45;  249 |

Abbreviations: Meth = methamphetamine

*Methamphetamine, alcohol, cannabis, and/or other illicit substances

**Alcohol, cannabis, and illicit substances (excluding methamphetamine)

Green cells indicate that the outcome is reported in the study; numbers indicate the total number of estimates per outcome reported in each study

# Substance use outcome domains and measures

1. Ability to initiate abstinence (number of tests that occurred prior to producing the first methamphetamine-negative test result) [n=1; N=10]
2. At least three negative urine screens out of a possible four screens [n=1; N=1]
3. **Longest period (days, weeks) or consecutive negative urine screens of uninterrupted/continuous abstinence (based on urine screens) [n=15; N=49]**
4. Consecutive weeks of continuous abstinence measured using NOBWOS – *Median NOBWOS* [n=3; N=5]
5. **Number (%) participants achieving consecutive weeks of abstinence in the final two weeks of treatment or study (based on urine screens) [n=7; N=17]**

- Consecutive weeks of continuous abstinence measured using NOBWOS - Number (%) participants with *NOBWOS value of 1 indicating 2 weeks of end of study abstinence*

1. **Number (%) participants achieving consecutive weeks of abstinence at any other period (i.e., not restricted to final 2 weeks) during the follow-up (based on urine screens) [n=11; N=59]**

- Number (%) participants with no positive urine screens throughout entire study (i.e., abstinence throughout entire study)
- Number (%) participants achieving consecutive weeks of abstinence at end of treatment/study but longer than two weeks (e.g., final 3 weeks; based on urine screens)
- Consecutive weeks of continuous abstinence measured using NOBWOS - Number (%) participants with *NOBWOS value of 2 to 11 indicating 3 to 12 weeks of end of study abstinence*
- Consecutive weeks of continuous abstinence measured using NOBWOS – Number (%) participants with *NOBWOS value of 0 (i.e., 0-1 weeks of end of study abstinence, indicating treatment failure)*

1. Number (%) participants with self-reported uninterrupted/continuous abstinence (i.e., self-reported abstinence throughout entire study) [n=1; N=3]
2. Number (%) participants that remained free of methamphetamine for at least 80% of treatment period/study (based on urine screens) [n=1; N=6]
3. Number (%) participants achieving methamphetamine abstinence at end of study (based on urine screens) [n=3; N=3]
4. Number (%) participants self-reporting abstinence [n=1; N=3]
5. Number (or %) of participants self-reporting use [n=1; N=1]
6. **Joint probability index [n=6; N=16]**
7. **Treatment effectiveness score: Mean number of negative urine drug screens [n=10; N=32]**
8. **Treatment effectiveness score: Percentage of negative urine drug screens (over total number of tests possible) [n=7; N=22]**
9. Number (%) participants with negative urine screen at a single timepoint [n=2; N=4]
10. Number (%) participants with positive urine screen at a single timepoint [n=4; N=8]
11. Number of days of self-reported use [n=2; N=13]
12. Percentage of positive oral fluid samples (over total number of samples submitted) [n=1; N=1]
13. **Overall change in proportion of negative urine screens [n=7; N=35]**
14. **Overall change in proportion of positive urine screens [n=10; N= 26]**
15. Change in proportion of participants with positive hair samples [n=1; N=2]
16. Proportion of participants that had a >15% decrease in methamphetamine use with treatment, assessed using urine screens [n=1; N=1]
17. Proportion of participants that had a >15% increase in methamphetamine use with treatment, assessed using urine screens [n=1; N=1]
18. Change in proportion of participants with a negative use week, *assessed using self-report* [n=1; N=1]
19. **Change in proportion of participants with a negative use week, *assessed using urine screens* [n=4; N=17]**
20. Change in median concentration of methamphetamine in hair sample [n=1; N=1]
21. Average weekly log10 median urine methamphetamine concentrations [n=2; N=5]
22. Proportion of participants that decreased the median quantitative urine methamphetamine concentration during the study period by ≥50% from baseline [n=1; N=1]
23. Proportion of participants that decreased the median quantitative urine methamphetamine concentration during the study period by ≥25% from baseline [n=1; N=2]
24. Change in mean percent days abstinent, self-report [n=1; N=1]
25. **Change in the mean/median number of self-reported days of use [n=9; N=12]**
26. Change in mean self-reported daily occasions of use [n=1; N=18]
27. Change in self-reported quantity (grams) of use [n=1; N=1]
28. Change in total use (number of days used multiplied by the amount used on each day), self-report [n=2; N=4]
29. Change in the frequency of self-reported use (measured as a categorical variable) [n=1; N=8]
30. Proportion of participants that decreased self-reported use days by ≥50% from baseline period [n=1; N=2]
31. Change in self-reported days since last use [n=1; N=1]

**Substance use outcomes**

**Change in methamphetamine use**

**Methamphetamine use, abstinence**

1. Number (or %) of participants with cocaine-positive urine screen (at a single timepoint (e.g., week 12) [n= 1; N=1]
2. Number (or %) of participants with opioid-positive urine screen (at a single timepoint (e.g., week 12) [n= 1; N=1]
3. Change in mean ASI Alcohol Score [n= 6; N=8]
4. Change in mean ASI Drug Score [n= 1; N=1]
5. Change in the mean number of self-reported days of alcohol use [n=2; N=2]
6. Change in the mean number of self-reported days of marijuana use [n= 2; N=2]
7. Change in the mean number of self-reported days of other drug use (opium, sedatives, polysubstance, heroin, alcohol use at intoxication level) [n=1; N=5]
8. Change in total substance use (number of days used multiplied by the amount used on each day) [n= 1; N=2]
9. Overall change in proportion of marijuana-negative urine screens [n=1; N=1]
10. Change in proportion of participants with hair samples positive for at least one other substance [n= 1; N=2]
11. Number (%) participants self-reporting alcohol use [n=1; N=1]
12. Number (%) participants self-reporting cannabinoid use [n=1; N=1]

**Other substances (use, abstinence and change)**

1. Abstinence throughout study (number or % of participants with no specimens positive for the substance throughout entire study) [n=1; N=1]
2. Longest period (days, weeks) of uninterrupted/continuous abstinence (based on urine screens) [n= 1; N=1]
3. Longest consecutive substance-negative screens (number of urine screens) [n= 1; N=1]
4. Treatment effectiveness score (TES) [n= 1; N=1]
5. Number of days of self-reported substance use [n= 1; N=1]
6. Quantity of substance use (mean quantity) [n= 1; N=1]
7. Spending on substances ($/day) [n= 1; N=1]
8. Change in mean ASI Drug Score [n=5; N=7]
9. Change in mean self-reported daily occasions of polydrug use [n=1; N=6]
10. Overall change in proportion of negative urine screens [n=1; N=2]

**Methamphetamines & other substances (use, abstinence, change)**

*n = number of studies; N = number of estimates

For select outcome domains, the most frequently reported measures are indicated in bold text.

1. Methamphetamine relapse (among those previously achieving abstinence) [n=2; N=7]
2. Illicit substance relapse (among those previously achieving abstinence) [n=1; N=1]

**Relapse**

# References

1. Abdoli N, Farnia V, Salemi S, Tatari F, Juibari TA, Alikhani M, et al. Efficacy of the Marlatt cognitive-behavioral model on decreasing relapse and craving in women with methamphetamine dependence: A clinical trial. Journal of Substance Use. 2019 Mar 4;24(2):229–32.

2. Abdoli N, Farnia V, Radmehr F, Alikhani M, Moradinazar M, Khodamoradi M, et al. The effect of self-compassion training on craving and self-efficacy in female patients with methamphetamine dependence: a one-year follow-up. Journal of Substance Use. 2021 Sep 3;26(5):491–6.

3. Amiri Z, Mirzaee B, Sabet M. Evaluating the efficacy of Regulated 12-Session Matrix Model in reducing susceptibility in methamphetamine-dependent individuals. International Journal of Medical Research & Health Sciences. 2016;5(2):77–85.

4. Anderson AL, Li SH, Biswas K, McSherry F, Holmes T, Iturriaga E, et al. Modafinil for the treatment of methamphetamine dependence. Drug Alcohol Depend. 2012 Jan 1;120(1–3):135–41.

5. Anderson AL, Li SH, Markova D, Holmes TH, Chiang N, Kahn R, et al. Bupropion for the treatment of methamphetamine dependence in non-daily users: a randomized, double-blind, placebo-controlled trial. Drug Alcohol Depend. 2015 May 1;150:170–4.

6. Aryan N, Banafshe HR, Farnia V, Shakeri J, Alikhani M, Rahimi H, et al. The therapeutic effects of methylphenidate and matrix-methylphenidate on addiction severity, craving, relapse and mental health in the methamphetamine use disorder. Subst Abuse Treat Prev Policy. 2020 Sep 25;15(1):72.

7. Baker A, Lee NK, Claire M, Lewin TJ, Grant T, Pohlman S, et al. Brief cognitive behavioural interventions for regular amphetamine users: a step in the right direction. Addiction. 2005 Mar;100(3):367–78.

8. Kay-Lambkin FJ, Baker AL, Lee NM, Jenner L, Lewin TJ. The influence of depression on treatment for methamphetamine use. Med J Aust. 2011 Aug 1;195(3):S38-43.

9. Lee NK, Pohlman S, Baker A, Ferris J, Kay-Lambkin F. It’s the thought that counts: craving metacognitions and their role in abstinence from methamphetamine use. J Subst Abuse Treat. 2010 Apr;38(3):245–50.

10. Briones M, Shoptaw S, Cook R, Worley M, Swanson AN, Moody DE, et al. Varenicline treatment for methamphetamine dependence: A randomized, double-blind phase II clinical trial. Drug Alcohol Depend. 2018 Aug 1;189:30–6.

11. Brown ES, Gabrielson B. A randomized, double-blind, placebo-controlled trial of citicoline for bipolar and unipolar depression and methamphetamine dependence. J Affect Disord. 2012 Dec 20;143(1–3):257–60.

12. Chudzynski J, Roll JM, McPherson S, Cameron JM, Howell DN. Reinforcement Schedule Effects on Long-Term Behavior Change. Psychol Rec. 2015 Jun 1;65(2):347–53.

13. Ciketic S, McKetin R, Doran CM, Najman JM, Veerman JL, Hayatbakhsh RM. Health-related quality of life (HRQL) among methamphetamine users in treatment. Mental Health and Substance Use. 2013 Aug;6(3):250–61.

14. McKetin R, Najman JM, Baker AL, Lubman DI, Dawe S, Ali R, et al. Evaluating the impact of community-based treatment options on methamphetamine use: findings from the Methamphetamine Treatment Evaluation Study (MATES). Addiction. 2012 Nov;107(11):1998–2008.

15. McKetin R, Kothe A, Baker AL, Lee NK, Ross J, Lubman DI. Predicting abstinence from methamphetamine use after residential rehabilitation: Findings from the Methamphetamine Treatment Evaluation Study. Drug Alcohol Rev. 2018 Jan;37(1):70–8.

16. Coffin PO, Santos GM, Das M, Santos DM, Huffaker S, Matheson T, et al. Aripiprazole for the treatment of methamphetamine dependence: a randomized, double-blind, placebo-controlled trial. Addiction. 2013 Apr;108(4):751–61.

17. Coffin PO, Santos GM, Hern J, Vittinghoff E, Santos D, Matheson T, et al. Extended-release naltrexone for methamphetamine dependence among men who have sex with men: a randomized placebo-controlled trial. Addiction. 2018 Feb;113(2):268–78.

18. Coffin PO, Santos GM, Hern J, Vittinghoff E, Walker JE, Matheson T, et al. Effects of Mirtazapine for Methamphetamine Use Disorder Among Cisgender Men and Transgender Women Who Have Sex With Men: A Placebo-Controlled Randomized Clinical Trial. JAMA Psychiatry. 2020 Mar 1;77(3):246–55.

19. Colfax GN, Santos GM, Das M, Santos DM, Matheson T, Gasper J, et al. Mirtazapine to reduce methamphetamine use: a randomized controlled trial. Arch Gen Psychiatry. 2011 Nov;68(11):1168–75.

20. Das M, Santos D, Matheson T, Santos GM, Chu P, Vittinghoff E, et al. Feasibility and acceptability of a phase II randomized pharmacologic intervention for methamphetamine dependence in high-risk men who have sex with men. AIDS. 2010 Apr 24;24(7):991–1000.

21. Elkashef AM, Rawson RA, Anderson AL, Li SH, Holmes T, Smith EV, et al. Bupropion for the treatment of methamphetamine dependence. Neuropsychopharmacology. 2008 Apr;33(5):1162–70.

22. McCann DJ, Li SH. A novel, nonbinary evaluation of success and failure reveals bupropion efficacy versus methamphetamine dependence: reanalysis of a multisite trial. CNS Neurosci Ther. 2012 May;18(5):414–8.

23. Elkashef A, Kahn R, Yu E, Iturriaga E, Li SH, Anderson A, et al. Topiramate for the treatment of methamphetamine addiction: a multi-center placebo-controlled trial. Addiction. 2012 Jul;107(7):1297–306.

24. Ma JZ, Johnson BA, Yu E, Weiss D, McSherry F, Saadvandi J, et al. Fine-grain analysis of the treatment effect of topiramate on methamphetamine addiction with latent variable analysis. Drug Alcohol Depend. 2013 Jun 1;130(1–3):45–51.

25. Farahzadi MH, Moazen-Zadeh E, Razaghi E, Zarrindast MR, Bidaki R, Akhondzadeh S. Riluzole for treatment of men with methamphetamine dependence: A randomized, double-blind, placebo-controlled clinical trial. J Psychopharmacol. 2019 Mar;33(3):305–15.

26. Fard MT, Mansouri SS, Jafari A, Vousooghi N. Role of modafinil in the treatment of patients with methamphetamine dependence; An update on randomized, controlled clinical trial. Trop J Pharm Res. 2020 Nov 26;19(10):2179–85.

27. Galloway GP, Newmeyer J, Knapp T, Stalcup SA, Smith D. A controlled trial of imipramine for the treatment of methamphetamine dependence. J Subst Abuse Treat. 1996 Dec;13(6):493–7.

28. Ghasemi A, Estebsari F, Bastaminia A, Jamshidi E, Dastoorpoor M. Effects of Educational Intervention on Health-Promoting Lifestyle and Health-Related Life quality of Methamphetamine Users and Their Families: a Randomized Clinical Trial. Iran Red Crescent Med J. 2014 Nov;16(11):e20024.

29. Heinzerling KG, Shoptaw S, Peck JA, Yang X, Liu J, Roll J, et al. Randomized, placebo-controlled trial of baclofen and gabapentin for the treatment of methamphetamine dependence. Drug Alcohol Depend. 2006 Dec 1;85(3):177–84.

30. Heinzerling KG, Swanson AN, Kim S, Cederblom L, Moe A, Ling W, et al. Randomized, double-blind, placebo-controlled trial of modafinil for the treatment of methamphetamine dependence. Drug Alcohol Depend. 2010 Jun 1;109(1–3):20–9.

31. Heinzerling KG, Swanson AN, Hall TM, Yi Y, Wu Y, Shoptaw SJ. Randomized, placebo-controlled trial of bupropion in methamphetamine-dependent participants with less than daily methamphetamine use. Addiction. 2014 Nov;109(11):1878–86.

32. Heinzerling KG, Briones M, Thames AD, Hinkin CH, Zhu T, Wu YN, et al. Randomized, Placebo-Controlled Trial of Targeting Neuroinflammation with Ibudilast to Treat Methamphetamine Use Disorder. J Neuroimmune Pharmacol. 2020 Jun;15(2):238–48.

33. Kamp F, Proebstl L, Hager L, Schreiber A, Riebschläger M, Neumann S, et al. Effectiveness of methamphetamine abuse treatment: Predictors of treatment completion and comparison of two residential treatment programs. Drug Alcohol Depend. 2019 Aug 1;201:8–15.

34. Kamp F, Hager L, Proebstl L, Schreiber A, Riebschläger M, Neumann S, et al. 12- and 18-month follow-up after residential treatment of methamphetamine dependence: Influence of treatment drop-out and different treatment concepts. J Psychiatr Res. 2020 Oct;129:103–10.

35. Kheirabadi GR, Ghavami M, Maracy MR, Salehi M, Sharbafchi MR. Effect of add-on valproate on craving in methamphetamine depended patients: A randomized trial. Adv Biomed Res. 2016;5:149.

36. Kheirabadi GR, Bahrami M, Shariat A, Tarrahi M. The Effect of Add-on Buprenorphine to Matrix Program in Reduction of Craving and Relapse Among People With Methamphetamine Use Disorder: A Randomized Controlled Trial. J Clin Psychopharmacol. 2021 Feb 1;41(1):45–8.

37. Ling W, Shoptaw S, Hillhouse M, Bholat MA, Charuvastra C, Heinzerling K, et al. Double-blind placebo-controlled evaluation of the PROMETA^TM^ protocol for methamphetamine dependence. Addiction. 2012 Feb;107(2):361–9.

38. Ling W, Chang L, Hillhouse M, Ang A, Striebel J, Jenkins J, et al. Sustained-release methylphenidate in a randomized trial of treatment of methamphetamine use disorder. Addiction. 2014 Sep;109(9):1489–500.

39. Longo M, Wickes W, Smout M, Harrison S, Cahill S, White JM. Randomized controlled trial of dexamphetamine maintenance for the treatment of methamphetamine dependence. Addiction. 2010 Jan;105(1):146–54.

40. Mimiaga MJ, Pantalone DW, Biello KB, Hughto JMW, Frank J, O’Cleirigh C, et al. An initial randomized controlled trial of behavioral activation for treatment of concurrent crystal methamphetamine dependence and sexual risk for HIV acquisition among men who have sex with men. AIDS Care. 2019 Sep;31(9):1083–95.

41. McKetin R, Dean OM, Turner A, Kelly PJ, Quinn B, Lubman DI, et al. N-acetylcysteine (NAC) for methamphetamine dependence: A randomised controlled trial. EClinicalMedicine. 2021 Aug;38:101005.

42. Noroozi A, Motevalian SA, Zarrindast MR, Alaghband-Rad J, Akhondzadeh S. Adding extended-release methylphenidate to psychological intervention for treatment of methamphetamine dependence: A double-blind randomized controlled trial. Med J Islam Repub Iran. 2020;34:137.

43. Perngparn U, Limanonda B, Aramrattana A, Pilley C, Areesantichai C, Taneepanichskul S. Methamphetamine dependence treatment rehabilitation in Thailand: a model assessment. J Med Assoc Thai. 2011 Jan;94(1):110–7.

44. Polcin DL, Bond J, Korcha R, Nayak MB, Galloway GP, Evans K. Randomized trial of intensive motivational interviewing for methamphetamine dependence. J Addict Dis. 2014;33(3):253–65.

45. Korcha RA, Polcin DL, Evans K, Bond JC, Galloway GP. Intensive motivational interviewing for women with concurrent alcohol problems and methamphetamine dependence. J Subst Abuse Treat. 2014 Feb;46(2):113–9.

46. Rawson RA, Marinelli-Casey P, Anglin MD, Dickow A, Frazier Y, Gallagher C, et al. A multi-site comparison of psychosocial approaches for the treatment of methamphetamine dependence. Addiction. 2004 Jun;99(6):708–17.

47. Rawson RA, Gonzales R, Pearce V, Ang A, Marinelli-Casey P, Brummer J, et al. Methamphetamine dependence and human immunodeficiency virus risk behavior. J Subst Abuse Treat. 2008 Oct;35(3):279–84.

48. Rawson RA, Gonzales R, Greenwell L, Chalk M. Process-of-care measures as predictors of client outcome among a methamphetamine-dependent sample at 12- and 36-month follow-ups. J Psychoactive Drugs. 2012 Oct;44(4):342–9.

49. Reback CJ, Rünger D, Fletcher JB, Swendeman D. Ecological momentary assessments for self-monitoring and counseling to optimize methamphetamine treatment and sexual risk reduction outcomes among gay and bisexual men. J Subst Abuse Treat. 2018 Sep;92:17–26.

50. Reback CJ, Fletcher JB, Swendeman DA, Metzner M. Theory-Based Text-Messaging to Reduce Methamphetamine Use and HIV Sexual Risk Behaviors Among Men Who Have Sex with Men: Automated Unidirectional Delivery Outperforms Bidirectional Peer Interactive Delivery. AIDS Behav. 2019 Jan;23(1):37–47.

51. Roll JM, Shoptaw S. Contingency management: schedule effects. Psychiatry Res. 2006 Sep 30;144(1):91–3.

52. Roll JM, Huber A, Sodano R, Chudzynski JE, Moynier E, Shoptaw S. A Comparison of Five Reinforcement Schedules for use in Contingency Management-Based Treatment of Methamphetamine Abuse. Psychol Rec. 2006 Jan;56(1):67–81.

53. Roll JM, Petry NM, Stitzer ML, Brecht ML, Peirce JM, McCann MJ, et al. Contingency management for the treatment of methamphetamine use disorders. Am J Psychiatry. 2006 Nov;163(11):1993–9.

54. Roll JM, Chudzynski J, Cameron JM, Howell DN, McPherson S. Duration effects in contingency management treatment of methamphetamine disorders. Addict Behav. 2013 Sep;38(9):2455–62.

55. Salehi M, Emadossadat A, Kheirabadi GR, Maracy MR, Sharbafchi MR. The Effect of Buprenorphine on Methamphetamine Cravings. J Clin Psychopharmacol. 2015 Dec;35(6):724–7.

56. Shearer J, Darke S, Rodgers C, Slade T, van Beek I, Lewis J, et al. A double-blind, placebo-controlled trial of modafinil (200 mg/day) for methamphetamine dependence. Addiction. 2009 Feb;104(2):224–33.

57. Shearer J, Shanahan M, Darke S, Rodgers C, van Beek I, McKetin R, et al. A cost-effectiveness analysis of modafinil therapy for psychostimulant dependence. Drug Alcohol Rev. 2010 May;29(3):235–42.

58. Shoptaw S, Reback CJ, Peck JA, Yang X, Rotheram-Fuller E, Larkins S, et al. Behavioral treatment approaches for methamphetamine dependence and HIV-related sexual risk behaviors among urban gay and bisexual men. Drug Alcohol Depend. 2005 May 9;78(2):125–34.

59. Peck JA, Reback CJ, Yang X, Rotheram-Fuller E, Shoptaw S. Sustained reductions in drug use and depression symptoms from treatment for drug abuse in methamphetamine-dependent gay and bisexual men. J Urban Health. 2005 Mar;82(1 Suppl 1):i100-108.

60. Jaffe A, Shoptaw S, Stein J, Reback CJ, Rotheram-Fuller E. Depression ratings, reported sexual risk behaviors, and methamphetamine use: latent growth curve models of positive change among gay and bisexual men in an outpatient treatment program. Exp Clin Psychopharmacol. 2007 Jun;15(3):301–7.

61. Shoptaw S, Huber A, Peck J, Yang X, Liu J, Jeff Dang null, et al. Randomized, placebo-controlled trial of sertraline and contingency management for the treatment of methamphetamine dependence. Drug Alcohol Depend. 2006 Oct 15;85(1):12–8.

62. Zorick T, Sugar CA, Hellemann G, Shoptaw S, London ED. Poor response to sertraline in methamphetamine dependence is associated with sustained craving for methamphetamine. Drug Alcohol Depend. 2011 Nov 1;118(2–3):500–3.

63. Shoptaw S, Reback CJ, Larkins S, Wang PC, Rotheram-Fuller E, Dang J, et al. Outcomes using two tailored behavioral treatments for substance abuse in urban gay and bisexual men. J Subst Abuse Treat. 2008 Oct;35(3):285–93.

64. Shoptaw S, Heinzerling KG, Rotheram-Fuller E, Steward T, Wang J, Swanson AN, et al. Randomized, placebo-controlled trial of bupropion for the treatment of methamphetamine dependence. Drug Alcohol Depend. 2008 Aug 1;96(3):222–32.

65. Brensilver M, Heinzerling KG, Swanson AN, Telesca D, Furst BA, Shoptaw SJ. Cigarette smoking as a target for potentiating outcomes for methamphetamine abuse treatment. Drug Alcohol Rev. 2013 Jan;32(1):96–9.

66. Smout MF, Longo M, Harrison S, Minniti R, Wickes W, White JM. Psychosocial treatment for methamphetamine use disorders: a preliminary randomized controlled trial of cognitive behavior therapy and Acceptance and Commitment Therapy. Subst Abus. 2010 Apr;31(2):98–107.

67. Sorsdahl K, Stein DJ, Pasche S, Jacobs Y, Kader R, Odlaug B, et al. A novel brief treatment for methamphetamine use disorders in South Africa: a randomised feasibility trial. Addict Sci Clin Pract. 2021 Jan 7;16(1):3.

68. Trivedi MH, Walker R, Ling W, Dela Cruz A, Sharma G, Carmody T, et al. Bupropion and Naltrexone in Methamphetamine Use Disorder. N Engl J Med. 2021 Jan 14;384(2):140–53.

69. Wang G, Ma L, Liu X, Yang X, Zhang S, Yang Y, et al. Paliperidone Extended-Release Tablets for the Treatment of Methamphetamine Use Disorder in Chinese Patients After Acute Treatment: A Randomized, Double-Blind, Placebo-Controlled Exploratory Study. Front Psychiatry. 2019;10:656.
