# Supplementary material for: Psychosocial and pharmacologic interventions for problematic methamphetamine use: Findings from a scoping review of the literature
Source: PLoS One. 2023 Oct 11;18(10):e0292745. doi: 10.1371/journal.pone.0292745 (PMC10566716; doi:10.1371/journal.pone.0292745)
Supplement: S12 Text — (DOCX) [file pone.0292745.s012.docx]

# S12 Text. Narrative summary of primary studies

#### Methamphetamine use and abstinence

##### Longest period of continuous abstinence, unrestricted sample of methamphetamine users

**Contingency management**

Four studies (1–4) reported a total of 23 unique comparisons. Chudzynski 2015 (1) examined different contingency management interventions including continuous, intermittent predictable, and intermittent unpredictable reinforcement. The direction of effect for each reported comparison is unclear as data were inadequately reported. Roll 2013 (3) compared different durations of contingency management. In comparison to standard psychosocial treatment alone, all durations of contingency management were associated with longer periods of continuous abstinence. Compared to those assigned to shorter durations of contingency management (i.e., 1 or 2 months), those receiving contingency management for 4 months experienced a longer period of continuous abstinence. One study (4) examined the effect of resetting the value of vouchers following relapse or a missed sample. The escalating with reset intervention was associated with a longer period of continuous abstinence as compared to the escalating without reset condition. The final study (2) compared five contingency management schedules. As compared to all other schedules, results favoured the schedule comprised of low initial magnitude of reinforcement, moderate escalation, moderate bonuses for continuous abstinence and resets in voucher.

**Matrix model**

A single study (5–7) reported on the effect of the Matrix model on continuous abstinence. Results favoured the Matrix model intervention as compared to treatment as usual.

**Pharmacotherapies**

Two trials reported on the effect of modafinil (8,9) and one trial each reported on the effects of aripiprazole (10), baclofen (11), gabapentin (11), bupropion (12,13), and methylphenidate (14). Of the two studies that compared modafinil 400 mg to placebo, one reported findings that favored the modafinil arm (9) while the direction of effect was unclear in the second study (8). Anderson 2012 (8) also reported that modafinil 200 mg was associated with a longer period of continuous abstinence as compared to placebo.

Compared to placebo, baclofen (11), bupropion (12,13), and methylphenidate (14), were associated with longer periods of continuous abstinence. Aripiprazole (10) and gabapentin (11) were associated with shorter periods of continuous abstinence as compared to placebo.

##### Treatment effectiveness score (TES), unrestricted sample of methamphetamine users

The treatment effectiveness score (TES) was reported as the mean number of methamphetamine-negative urine drugs screens or as a percentage of methamphetamine-negative drug screens.

**Contingency management**

In one study (4), those receiving the escalating with reset intervention submitted more methamphetamine-negative urines compared to those in the escalating without reset condition. Another trial (2) compared five contingency management schedules; as compared to all other schedules, results favoured the schedule consisting of high initial magnitude of reinforcement, slow escalation of voucher management, no bonuses for continuous abstinence, and no resets.

**Matrix model**

One study (5–7) reported that participants assigned to the Matrix model intervention submitted more methamphetamine-negative urine samples as compared to those receiving treatment as usual.

**Pharmacotherapies**

In a three-arm trial (11), individuals receiving baclofen submitted more methamphetamine-negative urines than both the placebo and gabapentin arms. Both studies comparing bupropion to placebo reported a higher treatment effectiveness score amongst those receiving bupropion (12,13,15). In the remaining three trials, compared to placebo, results favoured modafinil (9) but not ibidulast (16) or the PROMETA ^TM^ protocol (17).

##### Joint Probability Index, unrestricted sample of methamphetamine users

Joint probability index, defined as the probability of a methamphetamine-negative urine specimen at a given timepoint (18), was reported in four studies.

**Pharmacotherapies**

Heinzerling 2006 (11) reported that at 16 weeks, those assigned to baclofen had a greater probability of submitting a methamphetamine-negative urine sample as compared to those in the placebo and gabapentin arms. In one study each, results favoured bupropion (12,13) and modafinil (9) over placebo. In the remaining study, results favoured placebo over ibidulast (16).

##### Consecutive weeks of abstinence in final two weeks of treatment/study, unrestricted sample of methamphetamine users

Six studies reported the proportion of participants in each study arm that attained consecutive weeks of abstinence, confirmed by urine drug screen, in the final two weeks of the treatment period (i.e., weeks 11 and 12).

**Pharmacotherapies**

Two (15,19,20) of the three studies comparing bupropion to placebo reported results favouring bupropion. In the remaining study (21), a higher proportion of placebo participants achieved consecutive weeks of abstinence in the final two weeks of the treatment period. Heinzerling 2020 (16) and Anderson 2012 (8) reported results favouring placebo over ibudilast and modafinil 200 mg, respectively. Results varied across the two studies that compared modafinil 400 mg to placebo with one reporting results favouring placebo (8) and the other (9) reporting no difference between groups. In the modafinil dose comparison (400 mg vs 200 mg) reported by Anderson 2012 (8), more participants receiving the 400 mg dose achieved two weeks of consecutive abstinence at the end of treatment.

##### Consecutive weeks of abstinence at any other period during follow-up, unrestricted sample of methamphetamine users

Several studies reported the proportion of participants achieving consecutive weeks of abstinence at any other period during the study (i.e., not limited to the final two weeks of treatment or study). The duration of abstinence (i.e., the number of consecutive weeks) varied across studies. For example, some studies required two or more weeks of abstinence while others required longer durations.

**Contingency management**

Roll 2013 (3) examined the effect of varying durations of contingency management. Compared to standard psychosocial treatment, all durations of contingency management (i.e., 1, 2 and 4 month) were associated with a higher proportion of participants achieving 12 consecutive weeks of methamphetamine abstinence. Comparing the contingency management conditions, results favoured longer durations of contingency management in each of the comparisons. In a second study, Roll 2006 (2) compared the proportion of participants achieving four or more weeks of consecutive abstinence across five contingency management schedules. As compared to all other schedules, results favoured the schedule comprised of low initial magnitude of reinforcement, moderate escalation, moderate bonuses for continuous abstinence and resets in voucher.

**Matrix model**

One study (5–7) reported that a higher proportion of participants assigned to the Matrix model intervention submitted three consecutive negative urine samples (equivalent to three consecutive weeks of abstinence) as compared to those receiving treatment as usual.

**Pharmacotherapies**

A higher proportion of participants assigned to baclofen achieved two and three weeks of consecutive abstinence as compared to placebo and gabapentin (11). Another study reported that a lower percentage of participants receiving the PROMETA ^TM^ protocol achieved three weeks of consecutive abstinence as compared to placebo (17).

Three studies compared bupropion to placebo. Of the two studies (12,13,19,20) that reported data for two or more weeks of consecutive abstinence, both reported results favouring bupropion. Two studies reported data for four and eight weeks of consecutive abstinence with varying results; one reported no difference between groups (19,20) for both measures while the other reported results favouring placebo (21). Inconsistent findings were also reported in two studies providing data on consecutive abstinence for all 12 weeks of the trial with one favouring bupropion and the other placebo (19–21). Additional data from the bupropion trials, corresponding to different durations of consecutive abstinence, is reported in **S1 Data.**

In the four-arm trial reported by Shoptaw 2006 (22,23), sertraline plus contingency management outperformed sertraline alone but not the contingency management plus placebo condition. When the intervention arms were combined, a lower percentage of participants receiving sertraline achieved three or more weeks of consecutive abstinence as compared to the combined placebo arms. In the analysis combining the contingency management conditions, results favoured contingency management over no contingency management.

##### Longest period of continuous abstinence, gbMSM

**Cognitive behavioural therapy**

In the four-arm trial reported by Shoptaw 2005 (24–26), those receiving the combination intervention of voucher-based reinforcement plus CBT achieved a longer period of continuous abstinence as compared to both interventions alone as well as the gay and bisexual men-specific CBT condition.

**Pharmacotherapy**

One study (27) reported on the effect of mirtazapine on the longest period of continuous abstinence. Results favoured the mirtazapine arm at 24 and 36 weeks, but not at 12 weeks. In another study (28), naltrexone was associated with a shorter period of continuous abstinence as compared to placebo.

##### Treatment effectiveness score (TES), gbMSM

**Cognitive behavioural therapy**

Shoptaw 2005 (24–26) reported that the combination intervention of voucher-based reinforcement plus CBT outperformed both interventions alone as well as the gay and bisexual men-specific CBT condition. However, at longer follow-up (i.e., 52 weeks), the combination intervention only outperformed the gay and bisexual men-specific CBT. Compared to the CBT condition, those receiving gay and bisexual men-specific CBT submitted more methamphetamine-negative urine samples at the short-term (16 week) but not long-term (52 week) follow-up.

In a second study, Shoptaw 2008 (29) reported that participants receiving gay-specific CBT submitted more methamphetamine-negative urine samples as compared to those assigned to gay social support therapy.

##### Joint Probability Index, gbMSM

**Cognitive behavioural therapy**

Shoptaw 2008 (29) reported that compared to gay social support therapy, those receiving the gay-specific CBT intervention had a greater probability of submitting a methamphetamine-negative urine sample.

**Ecological momentary assessments**

In the trial reported by Reback 2018 (30), participants were randomized to mobile app-based ecological momentary assessments plus a web-based visualization dashboard with or without counselling. The study also included a third group of matched historical controls. Both intervention groups outperformed the matched historical control group. Comparing the two intervention arms, results favoured the intervention without the counselling component.

##### Consecutive weeks of abstinence, gbMSM

**Cognitive behavioural therapy**

Shoptaw 2008 (29) reported that compared to gay social support therapy, a higher proportion of participants assigned to the gay-specific CBT intervention achieved two consecutive weeks of abstinence.

**Pharmacotherapies**

As compared to placebo, a higher proportion of participants receiving mirtazapine achieved two consecutive weeks of abstinence during the 11^th^ and 12^th^ weeks of the trial reported by Coffin 2020 (27). Results were consistent for the final two weeks of treatment (i.e., weeks 23 and 24) as well as the final two weeks of the study (i.e., weeks 35 and 36).

##### Longest period of continuous abstinence, participants with mental health co-morbidities

Wang 2019 (31) recruited participants with a history of methamphetamine-associated psychosis but without current psychotic symptoms. Compared to placebo, participants receiving paliperidone achieved a longer period of continuous abstinence.

##### Treatment effectiveness score (TES), participants with mental health co-morbidities

Among participants with a history of methamphetamine-associated psychosis but without current psychotic symptoms, those receiving paliperidone submitted more methamphetamine-negative urine samples as compared to placebo (31).

##### Consecutive weeks of abstinence, participants with mental health co-morbidities

Anderson 2015 (21) reported subgroup data for participants with greater severity of depression as well as those with adult ADHD. The direction of effect for both analyses is unclear, as results were reported inadequately.

##### Longest period of continuous abstinence, participants with heavy baseline methamphetamine use

In a subgroup analysis restricted to those with three or more methamphetamine-positive urine samples during the lead-in period, participants assigned to bupropion achieved a longer period of continuous abstinence as compared to placebo (12,13). In a second study (9), participants with heavy baseline methamphetamine use (>18 days of past 30) assigned to modafinil achieved a longer period of continuous abstinence as compared to placebo.

##### Treatment effectiveness score (TES), participants with heavy baseline methamphetamine use

Four studies reported the proportion or mean number of methamphetamine-negative urine samples submitted by participants with heavy baseline methamphetamine use. One study each reported results favouring bupropion (12,13), modafinil (9), and combination naltrexone and bupropion (32) over placebo. The remaining study reported no difference between ibudilast and placebo (16).

##### Joint Probability Index, participants with heavy baseline methamphetamine use

Bupropion (12,13) and modafinil (9) outperformed placebo in one study each, while the remaining study favoured placebo over ibudilast (16).

##### Consecutive weeks of abstinence, participants with heavy baseline methamphetamine use

In two separate studies reported by the same author, a greater proportion of participants receiving ibudilast (16) and modafinil (9) achieved two weeks of consecutive abstinence in the final weeks of treatment (weeks 11 and 12) as compared to placebo.

Two studies reported the effect of bupropion toward achieving two or more consecutive weeks of abstinence among participants with heavy baseline use. Results favoured placebo in one of the studies (12,13) whereby heavy methamphetamine use was defined as three or more positive urine samples during the lead-in. The second study (19,20) reported results favouring bupropion for those who used methamphetamine on 19 to 29 days of 30 days at baseline, but not amongst those who used on all 30 days.

##### Longest period of continuous abstinence, participants with light baseline methamphetamine use

One study (12,13) reported results favouring bupropion over placebo amongst light methamphetamine users (0-2 methamphetamine-positive urine screens during lead-in). Another trial (9) favoured placebo over modafinil for light users (≤18 days of past 30 days).

##### Treatment effectiveness score (TES) and Joint Probability Index, participants with light baseline methamphetamine use

Shoptaw 2008 (12,13) reported that light baseline methamphetamine users receiving bupropion submitted more methamphetamine-negative urine drugs screens than those assigned to placebo. One study each reported results favouring placebo over ibudilast (16) and modafinil (9). Similar results were reported for the joint probability index **(see S1 Data, Figure A5**).

##### Consecutive weeks of abstinence, participants with light baseline methamphetamine use

Among light baseline methamphetamine users, one study each reported results favouring placebo over bupropion (21), ibudilast (16), and modafinil (9) regarding the proportion of participants achieving consecutive weeks of abstinence in the final two weeks of treatment (weeks 11 and 12).

Both studies (12,13,21) reporting the proportion of participants that achieved two or more weeks of consecutive abstinence during the trial reported results favouring bupropion. One study (19,20) reported that more placebo participants achieved three or more weeks of abstinence as compared to bupropion.

#### Change in methamphetamine use

##### Change in proportion of methamphetamine-negative urine samples, unrestricted sample of methamphetamine users

**Contingency management**

Two studies reported the effect of contingency management on the change in methamphetamine abstinence over time using generalized estimation equations (GEE) (1,3). The two studies reported 12 unique comparisons. Chudzynski 2015 (1) reported that over the 16-week treatment period, all contingency management conditions outperformed the no treatment condition. Compared to the intermittent unpredictable contingency management condition, those receiving continuous contingency management and those receiving intermittent predictable contingency management were more likely to submit a negative methamphetamine urine sample. Results favoured the intermittent predictable contingency management condition over continuous contingency management.

Roll 2013 (3) reported that both during the treatment period (baseline to 16 weeks) and longer follow-up (26 to 52 weeks), all durations of contingency management outperformed standard psychosocial treatment. Comparing the contingency management conditions, those assigned to 4 months of contingency management were more likely to submit a negative methamphetamine urine sample as compared to those in the 2-month and 1-month contingency management arms.

**Pharmacotherapies**

Two studies (11,16) reported results regarding change in proportion of methamphetamine-negative urine samples, however, the direction of effect for each reported comparison is unclear as results were reported inadequately. One study reported that placebo participants were more likely to submit a methamphetamine-negative urine sample as compared to those receiving modafinil 400 mg (9).

##### Change in proportion of methamphetamine-positive urine samples, unrestricted sample of methamphetamine users

**Pharmacotherapies**

Coffin 2013 (10) reported that over the course of the trial, participants receiving aripiprazole were less likely to submit methamphetamine-positive urine samples as compared to placebo. Shoptaw 2006 (22,23) reported that compared to sertraline alone, those receiving combination sertraline and contingency management were less likely to submit methamphetamine-positive urine samples over the 14-week follow-up. Results also favoured placebo and the placebo plus contingency management conditions over the sertraline-only condition. Fard 2020 (33) reported results favouring modafinil over CBT. One study (15) examined the effect of bupropion, however, the direction of effect was unclear.

Two studies reported on the effect of methylphenidate. Compared to placebo, the methylphenidate group in one of the studies (34) experienced a greater reduction in the percentage of positive weekly urine samples. In the second study (35), participants were randomized to receive methylphenidate, Matrix model treatment, methylphenidate plus Matrix model treatment, or no treatment. Results favoured methylphenidate over Matrix model and no treatment. However, the combination intervention outperformed all conditions.

##### Change in proportion of participants with a negative methamphetamine use week, unrestricted sample of methamphetamine users

**Pharmacotherapies**

Four studies reported the effect of pharmacotherapies on the change in proportion of participants with a negative methamphetamine use week over the course of the trial. A negative methamphetamine use week was defined as a week in which all available urine screens were methamphetamine-metabolite free.

Elkashef 2008 (19,20) reported that the bupropion group had a greater rate of increase in methamphetamine-free study weeks over the 12-week treatment period as compared to the placebo group. Results were inadequately reported in a second study of bupropion (12,13). Anderson 2012 (8) reported results favouring lower dose modafinil (200 mg) as compared to a higher dose (400 mg). Finally, results favoured topimirate over placebo during weeks 6 to 12 of the trial reported by Elkashef 2012 (36,37).

##### Change in number of self-reported days of methamphetamine use, unrestricted sample of methamphetamine users

**Matrix model**

During the treatment period, Rawson 2004 (5–7) reported a greater reduction in the number of days of self-reported methamphetamine use in the treatment as usual arm as compared to the Matrix model intervention. Results were consistent for the long-term follow-up period (up to 26 weeks).

**Blended imaginal desensitization plus motivational interviewing**

Compared to those receiving treatment as usual, participants receiving the combined psychosocial intervention experienced a greater reduction in the number of self-reported days of methamphetamine use (38).

**Pharmacotherapies**

Ling 2012 (17) reported a greater reduction in self-reported days of methamphetamine use in the placebo group as compared to the PROMETA ^TM^ protocol. Results favoured placebo over N-acetylcysteine (39) and methylphenidate (34) in two separate studies.

##### Change in proportion of methamphetamine-negative urine samples, gbMSM

Compared to participants receiving CBT, those receiving voucher-based reinforcement alone, gay and bisexual men-specific CBT alone, and combination CBT plus voucher-based reinforcement were more likely to submit methamphetamine-negative urine samples during the 16-week treatment period (24–26). Another study reported results favouring gay-specific CBT over gay social support therapy (29).

##### Change in proportion of methamphetamine-positive urine samples, gbMSM

Two studies (27,40) reported that the rate of methamphetamine-positive urine samples decreased among those receiving mirtazapine as compared to placebo. Coffin 2018 (28) reported results favouring naltrexone over placebo. Results were inadequately reported in one study of bupropion (41).

##### Change in number of self-reported days of methamphetamine use, gbMSM

Reback 2019 (42) reported that during the 39-week trial, the comparison condition consisting of weekly self-monitoring text-based assessments was associated with a greater reduction in the number of days of self-reported methamphetamine use when compared to automated gay-specific text messages plus weekly self-monitoring text-based assessments with or without interactive text-messaging conversations with a peer health educator. A second study by the same author (30) reported results favouring counselling plus mobile app-based ecological momentary assessments and a web-based visualization dashboard compared to a matched historical control group. However, results favoured the matched historical control group as compared to the intervention condition without the counselling component (**S1 Data, Figure A7**). A final study (29) reported a greater reduction in days of use among those receiving gay-specific CBT compared to gay social support therapy.

##### Change in methamphetamine use, participants with mental health comorbidities

One study (19,20) examined the effect of bupropion on the change in negative use weeks amongst those with higher severity of depressive symptoms while another examined citicoline (43) for change in self-reported days of use in those with bipolar depression or major depressive disorder. The direction of effect for each is unclear as results were inadequately reported.

Amongst those with lower severity of depressive symptoms (19,20), the bupropion group had a greater rate of increase in methamphetamine-free study weeks over the 12-week treatment period as compared to the placebo group.

##### Change in proportion of methamphetamine-negative urine samples, by HIV status

One study (16) reported data stratified by HIV status; the direction of effect is unclear due to reporting.

##### Change in proportion of participants with a negative methamphetamine use week, by severity of methamphetamine dependence

One study (36,37) reported a greater rate of increase in methamphetamine-negative use weeks amongst participants with lower severity of methamphetamine dependence receiving bupropion compared to placebo. Results were inadequately reported for the higher severity of dependence subgroup.

##### Change in methamphetamine positive urine samples, participants with heavy baseline methamphetamine use

Participants with heavy baseline methamphetamine use receiving bupropion were less likely to submit methamphetamine positive urine samples during the 12-week treatment period as compared to placebo (15).

##### Change in proportion of participants with a negative methamphetamine use week, participants with heavy baseline use

Two studies reported on the effect of bupropion on change in methamphetamine negative use week. One study reported no difference between groups (19,20) while the other favoured placebo amongst those with three or more methamphetamine-positive urine drug screens at baseline (12,13).

##### Change in proportion of participants with a negative methamphetamine use week, participants with light baseline use

Both studies (12,13,19,20) reported results favouring bupropion over placebo amongst participants with light baseline methamphetamine use.

#### Relapse

##### Methamphetamine relapse, unrestricted sample of methamphetamine users

**Contingency management**

One study (2) reported on the effect of contingency management on methamphetamine relapse following 4 weeks of abstinence. As compared to all other schedules, results favoured the schedule comprised of low initial magnitude of reinforcement, moderate escalation, moderate bonuses for continuous abstinence and resets in voucher.

##### Illegal substance relapse, unrestricted sample of methamphetamine users

**Inpatient residential rehabilitation (FAST model)**

One study (44) reported that a lower proportion of participants receiving inpatient residential rehabilitation relapsed on illegal substances as compared to those receiving outpatient Matrix model treatment.

##### Methamphetamine relapse, participants with mental health comorbidities

**Pharmacotherapies**

In a study of participants with a history of methamphetamine-associated psychosis but without current psychotic symptoms, fewer participants receiving paliperidone experienced methamphetamine relapse as compared to placebo (31).

#### Other substance use outcomes

Data on other substance use was sparsely reported. Two studies reported on methamphetamine and other substance use or abstinence. One study (45) of stimulant (i.e., cocaine, amphetamine, methamphetamine) use reported results favouring contingency management over treatment as usual for all abstinence measures. The second study (46,47) reported results favouring modafinil over placebo for all measures of illicit psychostimulant use (i.e., cocaine, methamphetamine or 3,4-methylenedioxymethamphetamine).

Regarding change in methamphetamine and other substance use measures, one study (45) reported that participants receiving contingency management were more likely to submit stimulant-negative urine samples as compared to those receiving treatment as usual. Another study (48,49) reported that the direction of effect favoured standard motivational interviewing over intensive motivational interviewing for change in self-reported drug use according to the Addiction Severity Index. A third study (50–52) found that more sessions of CBT were more effective in reducing self-reported daily occasions of poly drug use as compared to fewer sessions.

Other substance use (i.e., excluding methamphetamine) was reported in a small number of studies. Ling 2014 (14) reported that at 14 weeks, participants receiving placebo had a lower proportion of cocaine- and opioid-positive urine samples as compared to the methylphenidate group. Regarding change in other substance use measures, one study (48,49) reported results favouring intensive motivational interviewing over the standard condition for self-reported alcohol use. Results favoured methylphenidate over placebo for reduction of opium and sedative use but not for other substances (i.e., alcohol, marijuana, polysubstance, heroin) (34). Contingency management outperformed treatment as usual regarding change in proportion of marijuana-negative urine drug screens (45). Generally, results favoured CBT over ACT for measures of change in other substance use as reported by Smout 2010 (53).

#### Other outcomes

##### Risk behaviours

Among studies that did not restrict inclusion to a specific subpopulation of methamphetamine users, results favoured aripiprazole over placebo for reducing sexual risk behaviours in one study (10) and outpatient Matrix model treatment over inpatient residential rehabilitation for reducing risk taking behaviours in a second study (44). Results did not favour methylphenidate over placebo for a majority of risk behaviours reported by Noroozi 2020 (34). The direction of effects was unclear for several other studies due to inadequate reporting of findings.

The direction of effects for risk behaviour outcome measures in gbMSM was adequately reported in five studies. In one study (42), results consistently favoured automated gay-specific text messages plus weekly self-monitoring text-based assessments over weekly self-monitoring text-based assessments alone. The intervention condition also including interactive text-messaging conversations with a peer health educator outperformed weekly self-monitoring text-based assessments alone for some (but not all) sexual risk behaviours. Reback 2018 (30) reported greater reductions in sexual risk behaviours among participants receiving counselling plus mobile app-based ecological momentary assessments and web-based visualization dashboard as compared to matched historical controls. Results were inconsistent across outcome measures for the intervention arm without the counselling component. The effect of mirtazapine was examined in two studies (27,40). One study (40) consistently reported results favouring mirtazapine over placebo for reducing sexual risk behaviours. The second study (27) reported that mirtazapine was effective for reducing some sexual risk behaviours but not others. Finally, Coffin 2018 (28) reported results favouring naltrexone over placebo for reducing three of the six reported sexual risk behaviours.

##### Mental health outcomes

Studies reported on a variety of mental health outcomes including depressive symptoms, anxiety, and suicidal behaviours. Regarding reduction in depressive symptoms, the direction of effect favoured acceptance and commitment therapy over CBT at short-term follow-up; however, from weeks 12 to 24, reduction in depressive symptoms was observed only in the CBT group (53). Separate studies reported that baclofen (11), modafinil 400 mg (9), riluzole (54), and N-acetylcysteine (39) were associated with a greater reduction in depressive symptoms as compared to placebo. Other studies reporting on changes in depressive symptoms reported results favouring lower dose imipramine HCl (10 mg) over higher dose (150 mg) (55), placebo over aripiprazole (10), outpatient Matrix model treatment over inpatient residential rehabilitation (44),conventional group therapy alone over conventional group therapy plus 10 hours of group therapy focusing on stimulant use (56), and blended imaginal desensitization plus motivational interviewing over treatment as usual (38). Other measures of depression were sparsely reported (**S1 Data 19, Figure A24**). Regarding anxiety measures, one study each reported results favouring PROMETA ^TM^ protocol (17), sertraline (22,23), outpatient Matrix model treatment (44), and blended imaginal desensitization plus motivational interviewing (38). Remaining studies reported that intensive motivational interviewing did not decrease the number of days in which participants experienced anxiety (48,49) and two studies reported that anxiety occurred more frequently in participants receiving modafinil as compared to placebo (8,46,47). Results for other infrequently reported mental health outcome measures as well as data for specific subpopulations is reported in **S1 Data (Figures A24-A28)**.

##### Harms

Among studies that did not restrict inclusion to a specific subpopulation of methamphetamine users, separate trials reported that, compared to placebo, the rate of adverse events was higher in participants assigned to buprenorphine (57), bupropion (15), ibudilast (16), modafinil (9), topiramate (36,37), and varenicline (58). Remaining studies reported a lower frequency of adverse events in those receiving dexamphetamine (59), methylphenidate (14), and PROMETA ^TM^ protocol (17), as compared to placebo.

Regarding study withdrawal due to adverse events, one study reported that more participants assigned to baclofen withdrew from the study as compared to those receiving gabapentin or placebo (11). Compared to placebo, one study each reported that fewer participants assigned to dexamphetamine (59) and modafinil (46,47) withdrew due to adverse events. There was no difference in the rate of withdrawals between placebo and gabapentin (11), bupropion (12,13), or topiramate (36,37).

Adverse event data for specific subpopulations of methamphetamine users was sparsely reported. Among gbMSM, one study reported that one participant assigned to mirtazapine died by unintentional opioid overdose (27) and another study reported that one bupropion participant was diagnosed with HIV and rectal gonorrhea (41); these events were not experienced by placebo participants. A study of methamphetamine users with bipolar depression or major depressive disorder (43) reported more withdrawals due to adverse events in participants receiving citicoline as compared to those receiving placebo. In a study of participants with a history of methamphetamine-associated psychosis (31), as compared to placebo, more participants receiving paliperidone withdrew due to adverse events; however, fewer participants in the paliperidone group withdrew due to psychotic recurrence after methamphetamine relapse. Finally, a study (32) of participants with high baseline methamphetamine use (i.e., use on 18 or more days of last 30) reported that more participants assigned to combination naltrexone plus bupropion experience adverse events as compared to placebo.

##### Retention

Study and/or treatment retention was reported in nearly all studies (n=45). Regarding study retention, at least one study reported results favouring CBT (50–52), contingency management (3,45), and intensive motivational interviewing (48,49) as compared to treatment as usual, self-help booklet, or a standard psychosocial treatment. Compared to ACT, more participants receiving CBT were retained in the study reported by Smout (53). Results favoured treatment as usual over a blended imaginal desensitization plus motivational interviewing intervention (38). There was no difference in study retention between participants receiving an educational intervention and those receiving treatment as usual (60). Of the pharmacotherapies examined, results favoured placebo over aripiprazole (10), sertraline with or without contingency management (22,23), and PROMETA ^TM^ (17). Compared to placebo or CBT, two studies reported results favouring modafinil (33,46,47), and one study each reported results favouring dexamphetamine (59), riluzole (54), valproate (61), and varenicline (58). Results varied across the two studies reporting on bupropion; one reported no difference between groups (21) while the second reported a higher proportion of placebo participants retained in the study (12,13). However, in the latter study (12,13), participants receiving bupropion remained in the study longer than those receiving placebo. Of the two studies (14,34) comparing methylphenidate to placebo, only one (34) reported results favouring methylphenidate. In a four-arm trial, a greater proportion of participants receiving no treatment were retained in the study compared to those receiving Matrix model treatment or a combination of Matrix model treatment and methylphenidate (35). Of the remaining studies, one reported results favouring no treatment over community-based residential rehabilitation and detoxification (62–64), one reported results favouring inpatient residential rehabilitation over outpatient Matrix model treatment (44), and one reported results favouring conventional group therapy plus 10 hours of group therapy over treatment as usual (i.e., conventional group therapy only) (56,65).

For studies reporting data on both treatment and study retention, the direction of effect was generally similar for both outcomes (**S1 Data, Figure A33**). Of the studies only reporting data for treatment retention, one study each reported results favouring Matrix model (5–7), baclofen (11), ibudilast (16), and topiramate (36,37) as compared to treatment as usual or placebo. Both studies of modafinil 400 mg reported a higher proportion of modafinil participants retained in treatment as compared to placebo (8,9). Data for specific subpopulations is reported in **S1 Data (Figures A34-A37)**.

## References

1. Chudzynski J, Roll JM, McPherson S, Cameron JM, Howell DN. Reinforcement Schedule Effects on Long-Term Behavior Change. Psychol Rec. 2015 Jun 1;65(2):347–53.

2. Roll JM, Huber A, Sodano R, Chudzynski JE, Moynier E, Shoptaw S. A Comparison of Five Reinforcement Schedules for use in Contingency Management-Based Treatment of Methamphetamine Abuse. Psychol Rec. 2006 Jan;56(1):67–81.

3. Roll JM, Chudzynski J, Cameron JM, Howell DN, McPherson S. Duration effects in contingency management treatment of methamphetamine disorders. Addict Behav. 2013 Sep;38(9):2455–62.

4. Roll JM, Shoptaw S. Contingency management: schedule effects. Psychiatry Res. 2006 Sep 30;144(1):91–3.

5. Rawson RA, Marinelli-Casey P, Anglin MD, Dickow A, Frazier Y, Gallagher C, et al. A multi-site comparison of psychosocial approaches for the treatment of methamphetamine dependence. Addiction. 2004 Jun;99(6):708–17.

6. Rawson RA, Gonzales R, Pearce V, Ang A, Marinelli-Casey P, Brummer J, et al. Methamphetamine dependence and human immunodeficiency virus risk behavior. J Subst Abuse Treat. 2008 Oct;35(3):279–84.

7. Rawson RA, Gonzales R, Greenwell L, Chalk M. Process-of-care measures as predictors of client outcome among a methamphetamine-dependent sample at 12- and 36-month follow-ups. J Psychoactive Drugs. 2012 Oct;44(4):342–9.

8. Anderson AL, Li SH, Biswas K, McSherry F, Holmes T, Iturriaga E, et al. Modafinil for the treatment of methamphetamine dependence. Drug Alcohol Depend. 2012 Jan 1;120(1–3):135–41.

9. Heinzerling KG, Swanson AN, Kim S, Cederblom L, Moe A, Ling W, et al. Randomized, double-blind, placebo-controlled trial of modafinil for the treatment of methamphetamine dependence. Drug Alcohol Depend. 2010 Jun 1;109(1–3):20–9.

10. Coffin PO, Santos GM, Das M, Santos DM, Huffaker S, Matheson T, et al. Aripiprazole for the treatment of methamphetamine dependence: a randomized, double-blind, placebo-controlled trial. Addiction. 2013 Apr;108(4):751–61.

11. Heinzerling KG, Shoptaw S, Peck JA, Yang X, Liu J, Roll J, et al. Randomized, placebo-controlled trial of baclofen and gabapentin for the treatment of methamphetamine dependence. Drug Alcohol Depend. 2006 Dec 1;85(3):177–84.

12. Shoptaw S, Heinzerling KG, Rotheram-Fuller E, Steward T, Wang J, Swanson AN, et al. Randomized, placebo-controlled trial of bupropion for the treatment of methamphetamine dependence. Drug Alcohol Depend. 2008 Aug 1;96(3):222–32.

13. Brensilver M, Heinzerling KG, Swanson AN, Telesca D, Furst BA, Shoptaw SJ. Cigarette smoking as a target for potentiating outcomes for methamphetamine abuse treatment. Drug Alcohol Rev. 2013 Jan;32(1):96–9.

14. Ling W, Chang L, Hillhouse M, Ang A, Striebel J, Jenkins J, et al. Sustained-release methylphenidate in a randomized trial of treatment of methamphetamine use disorder. Addiction. 2014 Sep;109(9):1489–500.

15. Heinzerling KG, Swanson AN, Hall TM, Yi Y, Wu Y, Shoptaw SJ. Randomized, placebo-controlled trial of bupropion in methamphetamine-dependent participants with less than daily methamphetamine use. Addiction. 2014 Nov;109(11):1878–86.

16. Heinzerling KG, Briones M, Thames AD, Hinkin CH, Zhu T, Wu YN, et al. Randomized, Placebo-Controlled Trial of Targeting Neuroinflammation with Ibudilast to Treat Methamphetamine Use Disorder. J Neuroimmune Pharmacol. 2020 Jun;15(2):238–48.

17. Ling W, Shoptaw S, Hillhouse M, Bholat MA, Charuvastra C, Heinzerling K, et al. Double-blind placebo-controlled evaluation of the PROMETA^TM^ protocol for methamphetamine dependence. Addiction. 2012 Feb;107(2):361–9.

18. Ling W, Shoptaw S, Wesson D, Rawson RA, Compton M, Klett CJ. Treatment effectiveness score as an outcome measure in clinical trials. NIDA Res Monogr. 1997;175:208–20.

19. Elkashef AM, Rawson RA, Anderson AL, Li SH, Holmes T, Smith EV, et al. Bupropion for the treatment of methamphetamine dependence. Neuropsychopharmacology. 2008 Apr;33(5):1162–70.

20. McCann DJ, Li SH. A novel, nonbinary evaluation of success and failure reveals bupropion efficacy versus methamphetamine dependence: reanalysis of a multisite trial. CNS Neurosci Ther. 2012 May;18(5):414–8.

21. Anderson AL, Li SH, Markova D, Holmes TH, Chiang N, Kahn R, et al. Bupropion for the treatment of methamphetamine dependence in non-daily users: a randomized, double-blind, placebo-controlled trial. Drug Alcohol Depend. 2015 May 1;150:170–4.

22. Shoptaw S, Huber A, Peck J, Yang X, Liu J, Jeff Dang null, et al. Randomized, placebo-controlled trial of sertraline and contingency management for the treatment of methamphetamine dependence. Drug Alcohol Depend. 2006 Oct 15;85(1):12–8.

23. Zorick T, Sugar CA, Hellemann G, Shoptaw S, London ED. Poor response to sertraline in methamphetamine dependence is associated with sustained craving for methamphetamine. Drug Alcohol Depend. 2011 Nov 1;118(2–3):500–3.

24. Shoptaw S, Reback CJ, Peck JA, Yang X, Rotheram-Fuller E, Larkins S, et al. Behavioral treatment approaches for methamphetamine dependence and HIV-related sexual risk behaviors among urban gay and bisexual men. Drug Alcohol Depend. 2005 May 9;78(2):125–34.

25. Peck JA, Reback CJ, Yang X, Rotheram-Fuller E, Shoptaw S. Sustained reductions in drug use and depression symptoms from treatment for drug abuse in methamphetamine-dependent gay and bisexual men. J Urban Health. 2005 Mar;82(1 Suppl 1):i100-108.

26. Jaffe A, Shoptaw S, Stein J, Reback CJ, Rotheram-Fuller E. Depression ratings, reported sexual risk behaviors, and methamphetamine use: latent growth curve models of positive change among gay and bisexual men in an outpatient treatment program. Exp Clin Psychopharmacol. 2007 Jun;15(3):301–7.

27. Coffin PO, Santos GM, Hern J, Vittinghoff E, Walker JE, Matheson T, et al. Effects of Mirtazapine for Methamphetamine Use Disorder Among Cisgender Men and Transgender Women Who Have Sex With Men: A Placebo-Controlled Randomized Clinical Trial. JAMA Psychiatry. 2020 Mar 1;77(3):246–55.

28. Coffin PO, Santos GM, Hern J, Vittinghoff E, Santos D, Matheson T, et al. Extended-release naltrexone for methamphetamine dependence among men who have sex with men: a randomized placebo-controlled trial. Addiction. 2018 Feb;113(2):268–78.

29. Shoptaw S, Reback CJ, Larkins S, Wang PC, Rotheram-Fuller E, Dang J, et al. Outcomes using two tailored behavioral treatments for substance abuse in urban gay and bisexual men. J Subst Abuse Treat. 2008 Oct;35(3):285–93.

30. Reback CJ, Rünger D, Fletcher JB, Swendeman D. Ecological momentary assessments for self-monitoring and counseling to optimize methamphetamine treatment and sexual risk reduction outcomes among gay and bisexual men. J Subst Abuse Treat. 2018 Sep;92:17–26.

31. Wang G, Ma L, Liu X, Yang X, Zhang S, Yang Y, et al. Paliperidone Extended-Release Tablets for the Treatment of Methamphetamine Use Disorder in Chinese Patients After Acute Treatment: A Randomized, Double-Blind, Placebo-Controlled Exploratory Study. Front Psychiatry. 2019;10:656.

32. Trivedi MH, Walker R, Ling W, Dela Cruz A, Sharma G, Carmody T, et al. Bupropion and Naltrexone in Methamphetamine Use Disorder. N Engl J Med. 2021 Jan 14;384(2):140–53.

33. Fard MT, Mansouri SS, Jafari A, Vousooghi N. Role of modafinil in the treatment of patients with methamphetamine dependence; An update on randomized, controlled clinical trial. Trop J Pharm Res. 2020 Nov 26;19(10):2179–85.

34. Noroozi A, Motevalian SA, Zarrindast MR, Alaghband-Rad J, Akhondzadeh S. Adding extended-release methylphenidate to psychological intervention for treatment of methamphetamine dependence: A double-blind randomized controlled trial. Med J Islam Repub Iran. 2020;34:137.

35. Aryan N, Banafshe HR, Farnia V, Shakeri J, Alikhani M, Rahimi H, et al. The therapeutic effects of methylphenidate and matrix-methylphenidate on addiction severity, craving, relapse and mental health in the methamphetamine use disorder. Subst Abuse Treat Prev Policy. 2020 Sep 25;15(1):72.

36. Elkashef A, Kahn R, Yu E, Iturriaga E, Li SH, Anderson A, et al. Topiramate for the treatment of methamphetamine addiction: a multi-center placebo-controlled trial. Addiction. 2012 Jul;107(7):1297–306.

37. Ma JZ, Johnson BA, Yu E, Weiss D, McSherry F, Saadvandi J, et al. Fine-grain analysis of the treatment effect of topiramate on methamphetamine addiction with latent variable analysis. Drug Alcohol Depend. 2013 Jun 1;130(1–3):45–51.

38. Sorsdahl K, Stein DJ, Pasche S, Jacobs Y, Kader R, Odlaug B, et al. A novel brief treatment for methamphetamine use disorders in South Africa: a randomised feasibility trial. Addict Sci Clin Pract. 2021 Jan 7;16(1):3.

39. McKetin R, Dean OM, Turner A, Kelly PJ, Quinn B, Lubman DI, et al. N-acetylcysteine (NAC) for methamphetamine dependence: A randomised controlled trial. EClinicalMedicine. 2021 Aug;38:101005.

40. Colfax GN, Santos GM, Das M, Santos DM, Matheson T, Gasper J, et al. Mirtazapine to reduce methamphetamine use: a randomized controlled trial. Arch Gen Psychiatry. 2011 Nov;68(11):1168–75.

41. Das M, Santos D, Matheson T, Santos GM, Chu P, Vittinghoff E, et al. Feasibility and acceptability of a phase II randomized pharmacologic intervention for methamphetamine dependence in high-risk men who have sex with men. AIDS. 2010 Apr 24;24(7):991–1000.

42. Reback CJ, Fletcher JB, Swendeman DA, Metzner M. Theory-Based Text-Messaging to Reduce Methamphetamine Use and HIV Sexual Risk Behaviors Among Men Who Have Sex with Men: Automated Unidirectional Delivery Outperforms Bidirectional Peer Interactive Delivery. AIDS Behav. 2019 Jan;23(1):37–47.

43. Brown ES, Gabrielson B. A randomized, double-blind, placebo-controlled trial of citicoline for bipolar and unipolar depression and methamphetamine dependence. J Affect Disord. 2012 Dec 20;143(1–3):257–60.

44. Perngparn U, Limanonda B, Aramrattana A, Pilley C, Areesantichai C, Taneepanichskul S. Methamphetamine dependence treatment rehabilitation in Thailand: a model assessment. J Med Assoc Thai. 2011 Jan;94(1):110–7.

45. Roll JM, Petry NM, Stitzer ML, Brecht ML, Peirce JM, McCann MJ, et al. Contingency management for the treatment of methamphetamine use disorders. Am J Psychiatry. 2006 Nov;163(11):1993–9.

46. Shearer J, Darke S, Rodgers C, Slade T, van Beek I, Lewis J, et al. A double-blind, placebo-controlled trial of modafinil (200 mg/day) for methamphetamine dependence. Addiction. 2009 Feb;104(2):224–33.

47. Shearer J, Shanahan M, Darke S, Rodgers C, van Beek I, McKetin R, et al. A cost-effectiveness analysis of modafinil therapy for psychostimulant dependence. Drug Alcohol Rev. 2010 May;29(3):235–42.

48. Polcin DL, Bond J, Korcha R, Nayak MB, Galloway GP, Evans K. Randomized trial of intensive motivational interviewing for methamphetamine dependence. J Addict Dis. 2014;33(3):253–65.

49. Korcha RA, Polcin DL, Evans K, Bond JC, Galloway GP. Intensive motivational interviewing for women with concurrent alcohol problems and methamphetamine dependence. J Subst Abuse Treat. 2014 Feb;46(2):113–9.

50. Baker A, Lee NK, Claire M, Lewin TJ, Grant T, Pohlman S, et al. Brief cognitive behavioural interventions for regular amphetamine users: a step in the right direction. Addiction. 2005 Mar;100(3):367–78.

51. Kay-Lambkin FJ, Baker AL, Lee NM, Jenner L, Lewin TJ. The influence of depression on treatment for methamphetamine use. Med J Aust. 2011 Aug 1;195(3):S38-43.

52. Lee NK, Pohlman S, Baker A, Ferris J, Kay-Lambkin F. It’s the thought that counts: craving metacognitions and their role in abstinence from methamphetamine use. J Subst Abuse Treat. 2010 Apr;38(3):245–50.

53. Smout MF, Longo M, Harrison S, Minniti R, Wickes W, White JM. Psychosocial treatment for methamphetamine use disorders: a preliminary randomized controlled trial of cognitive behavior therapy and Acceptance and Commitment Therapy. Subst Abus. 2010 Apr;31(2):98–107.

54. Farahzadi MH, Moazen-Zadeh E, Razaghi E, Zarrindast MR, Bidaki R, Akhondzadeh S. Riluzole for treatment of men with methamphetamine dependence: A randomized, double-blind, placebo-controlled clinical trial. J Psychopharmacol. 2019 Mar;33(3):305–15.

55. Galloway GP, Newmeyer J, Knapp T, Stalcup SA, Smith D. A controlled trial of imipramine for the treatment of methamphetamine dependence. J Subst Abuse Treat. 1996 Dec;13(6):493–7.

56. Kamp F, Proebstl L, Hager L, Schreiber A, Riebschläger M, Neumann S, et al. Effectiveness of methamphetamine abuse treatment: Predictors of treatment completion and comparison of two residential treatment programs. Drug Alcohol Depend. 2019 Aug 1;201:8–15.

57. Salehi M, Emadossadat A, Kheirabadi GR, Maracy MR, Sharbafchi MR. The Effect of Buprenorphine on Methamphetamine Cravings. J Clin Psychopharmacol. 2015 Dec;35(6):724–7.

58. Briones M, Shoptaw S, Cook R, Worley M, Swanson AN, Moody DE, et al. Varenicline treatment for methamphetamine dependence: A randomized, double-blind phase II clinical trial. Drug Alcohol Depend. 2018 Aug 1;189:30–6.

59. Longo M, Wickes W, Smout M, Harrison S, Cahill S, White JM. Randomized controlled trial of dexamphetamine maintenance for the treatment of methamphetamine dependence. Addiction. 2010 Jan;105(1):146–54.

60. Ghasemi A, Estebsari F, Bastaminia A, Jamshidi E, Dastoorpoor M. Effects of Educational Intervention on Health-Promoting Lifestyle and Health-Related Life quality of Methamphetamine Users and Their Families: a Randomized Clinical Trial. Iran Red Crescent Med J. 2014 Nov;16(11):e20024.

61. Kheirabadi GR, Ghavami M, Maracy MR, Salehi M, Sharbafchi MR. Effect of add-on valproate on craving in methamphetamine depended patients: A randomized trial. Adv Biomed Res. 2016;5:149.

62. Ciketic S, McKetin R, Doran CM, Najman JM, Veerman JL, Hayatbakhsh RM. Health-related quality of life (HRQL) among methamphetamine users in treatment. Mental Health and Substance Use. 2013 Aug;6(3):250–61.

63. McKetin R, Najman JM, Baker AL, Lubman DI, Dawe S, Ali R, et al. Evaluating the impact of community-based treatment options on methamphetamine use: findings from the Methamphetamine Treatment Evaluation Study (MATES). Addiction. 2012 Nov;107(11):1998–2008.

64. McKetin R, Kothe A, Baker AL, Lee NK, Ross J, Lubman DI. Predicting abstinence from methamphetamine use after residential rehabilitation: Findings from the Methamphetamine Treatment Evaluation Study. Drug Alcohol Rev. 2018 Jan;37(1):70–8.

65. Kamp F, Hager L, Proebstl L, Schreiber A, Riebschläger M, Neumann S, et al. 12- and 18-month follow-up after residential treatment of methamphetamine dependence: Influence of treatment drop-out and different treatment concepts. J Psychiatr Res. 2020 Oct;129:103–10.
